# Comparative genomic and transcriptomic analyses of chemosensory genes in the citrus fruit fly *Bactrocera* (*Tetradacus*) *minax*

**DOI:** 10.1038/s41598-020-74803-5

**Published:** 2020-10-22

**Authors:** Jun-Feng Cheng, Ting Yu, Zhong-Jian Chen, Shicheng Chen, Yu-Peng Chen, Lei Gao, Wen-Hu Zhang, Bo Jiang, Xue Bai, Edward D. Walker, Jun Liu, Yong-Yue Lu

**Affiliations:** 1 College of Agriculture, South China Agricultural University, Guangzhou, Guangdong China; 2 Agro-Biological Gene Research Center, Guangdong Academy of Agricultural Sciences, Guangzhou, Guangdong China; 3 Department of Microbiology and Molecular Genetics, Michigan State University, East Lansing, USA; 4 Crop Research Institute, Guangdong Academy of Agricultural Sciences, Guangdong Provincial Key Laboratory of Crop Genetics and Improvement, Guangzhou, Guangdong China; 5 Fruit Tree Research Institute, Guangdong Academy of Agricultural Sciences, Guangzhou, Guangdong China

**Keywords:** Transcriptional regulatory elements, Transcriptomics

## Abstract

The citrus fruit fly *Bactrocera* (*Tetradacus*) *minax* is a major and devastating agricultural pest in Asian subtropical countries. Previous studies have shown that *B. minax* interacts with plant hosts via the efficient chemosensory system. However, the molecular components of the *B. minax* chemosensory system have not been well characterized. Herein, we identified a total of 25 putative odorant-binding receptors (OBPs), 4 single-copy chemosensory proteins (CSPs) and 53 candidate odorant receptors (ORs) using a newly generated whole-genome dataset for *B. minax*. This study significantly extended the chemosensation-related gene profiles (particularly, OBPs and ORs) in six other tephritid species. Comparative transcriptome analysis of adult *B. minax* and *Bactrocera dorsalis* showed that there were 14 highly expressed OBPs (FPKM > 100) in *B. dorsalis* and 7 highly expressed ones in *B. minax*. The expression level of *CSP*3 gene and *CSP*4 gene was higher in *B. dorsalis* than that in *B. minax*. Comparative genomic and transcriptomic analyses of chemosensory genes in the citrus fruit fly *B. minax* provided new insights for preventive control of this agriculture important pest and closely related species.

## Introduction

*Bactrocera* (*Tetradacus*) *minax* (Diptera: Tephritidiae) is a destructive pest that damages primarily citrus tree fruits and diminishes fruit production^1^. Once severe infestations occur in citrus-producing areas, severe economic losses typically follow^1^. Therefore, new prevention and control methods specifically targeting *B. minax* are urgently needed.

Insects recognize their plant hosts mainly via perception of chemical signals emanating from the plants^2^. Odorant-binding proteins (OBPs), chemosensory proteins (CSPs) and odorant receptors (ORs) are involved in insect chemosensation^3–7^. The current model for insect chemosensation and recognition is as follows^2,6–8^: some lipophilic odorant molecules in the environment reach the hydrophilic lymph of the insect through micropores on the olfactory sensilla surface and form a complex with the OBPs or CSPs in the sensillar lymph. Then, the complex passes through the sensillar lymph and binds to the ORs on the dendritic membranes. The membrane permeability changes when ORs are stimulated with the bindings. The above process results in the formation of an action potential and triggering cascade reactions, with the complex eventually entering the insect’s central nervous system. Therefore, insects can sense exogenous odorant molecules and react accordingly with physiological and behavioral responses, such as foraging for food and oviposition sites.

The sizes of OBPs are approximately 15–17 kDa, consisting of 120–150 amino acids^9^. Specifically, the complete amino acid sequences of OBPs from *B. dorsalis* have a length ranging from 134 to 274 amino acids^10^. OBPs on the surface of insect antennae play an important role in binding odorants such as volatile substances^7^. Similar to OBPs, CSPs are also small, highly water-soluble, and acidic proteins with hydrophobic binding sites^11^. CSPs contain about 120 amino acids (12–14 kDa). CSPs have been reported to participate in the chemosensory process by delivering hydrophobic sensory molecules to trigger neuronal responses in other insects (such as locusts, ants and *Bemisia tabaci*^12–14^).

Insect ORs are a group of G-protein-coupled receptors with seven transmembrane domains. Different from those in vertebrate ORs^5^, the N terminal peptides of the ORs of insects are located within the cell while the C terminal domains are located outside. Insect ORs include both the conventional ORs and the atypical OR Or83b^15^. *Drosophila* olfactory conventional ORs and Or83b encode 370–400 amino acids and 486 amino acids, respectively^16^. The insect Or83b protein genes were coexpressed with conventional ORs in most olfactory neurons^17,18^, which possibly impact on the olfactory behavior of insects^19^.

Application of sex pheromones to trap and kill adults is an important technique for managing *B. minax* infestation, which effectively reduces the number of pests and the rate of fruit damage^1^. Thus, the identification of olfaction-related genes provides insights into the alternative strategies for specifically targeting these agriculturally important pests. However, the molecular components of olfaction system in *B. minax* have not been well studied. In this study, we focused on identification of the new chemosensory genes in *B. minax* by comparison to those from other tephritid species. Moreover, the expression patterns at three different developmental stages (larva, pupa and adult) were compared between *B. dorsalis* and *B. minax*.

## Results

### Identification and gene expression of OBPs in *B. minax*

A total of 25, 37, 33, 35, 30, 29, 34 OBP genes were identified in *B. minax*, *B. dorsalis*, *B. cucurbitae*, *B. latifrons*, *B. oleae*, *R. zephyria*, and *C. capitata*, respectively (Table 1). 37 of 52 OBP sequences in *D. melanogaster* showed high homology to those in the seven selected tephritid species (Table 1). Among them, the sequences of 8 OBPs (19a, 19d, 50e, 56a, 56d, 56h, 84a and 99c) were conserved (Table 1).

**Table 1:** Identification of OBP genes in Tephritidae*.

OBPs	Tephritidae									
	Dmel	Bmin	Bdor	Bcuc	Blat	Bole	Rzep	Ccap	Tephritidae	Total
8a	1	1	1	1	1	1	1	1	+	8
18a	1	0	0	0	0	0	0	0	–	1
19a	1	2	2	1	2	2	2	2	+	14
19b	1	1	1	1	1	1	1	1	+	8
19c	1	1	1	1	1	1	1	1	+	8
19d	1	3	3	3	2	3	3	3	+	21
22a	1	0	0	0	0	0	0	0	−	1
28a	1	1	1	1	1	1	1	1	+	8
44a	1	0	1	1	1	1	1	1	+	7
46a	1	0	1	0	0	0	1	1	+	4
47a	1	1	1	1	1	1	0	1	+	7
47b	1	0	0	1	1	1	0	1	+	5
49a	1	0	0	0	0	0	0	0	−	1
50a	1	0	1	1	1	0	0	0	+	4
50b	1	0	0	0	0	0	0	0	−	1
50c	1	1	1	1	1	0	2	1	+	8
50d	1	0	0	0	0	0	0	0	−	1
50e	1	0	1	**7**	2	2	**5**	2	+	20
51a	1	0	0	0	0	0	0	0	−	1
56a	1	1	1	1	2	2	3	2	+	13
56b	1	1	1	1	1	1	1	1	+	8
56c	1	1	1	1	1	1	1	1	+	8
56d	1	1	3	1	2	1	3	1	+	13
56e	1	0	1	1	1	1	0	1	+	6
56f	1	0	0	0	0	0	0	0	−	1
56g	1	1	1	1	1	0	2	2	+	9
56h	1	2	3	3	3	2	2	2	+	18
56i	1	0	0	0	0	0	0	0	−	1
57a	1	0	0	0	0	0	0	0	−	1
57b	1	0	0	0	0	0	0	0	−	1
57c	1	0	1	1	1	1	1	1	+	7
57d	1	0	0	0	0	0	0	0	−	1
57e	1	0	0	0	0	0	0	0	−	1
58b	1	0	0	0	0	0	0	0	−	1
58c	1	1	1	1	1	0	0	1	+	6
58d	1	0	1	1	1	0	0	1	+	5
59a	1	0	1	0	1	0	0	1	+	4
69a	1	0	1	0	0	1	1	1	+	5
73a	1	1	1	1	1	1	1	1	+	8
83a	1	1	1	1	1	1	1	1	+	8
83b	1	1	1	1	1	1	2	1	+	9
83cd	1	1	1	0	1	1	1	1	+	7
83ef	1	0	1	1	1	1	1	0	+	6
83g	1	1	1	1	1	1	0	1	+	7
84a	1	1	2	2	2	2	2	2	+	14
85a	1	0	0	0	0	0	0	0	−	1
93a	1	0	0	0	0	0	0	0	−	1
99a	1	0	1	1	1	1	1	0	+	6
99b	1	1	1	1	1	1	1	1	+	8
99c	1	2	**8**	2	**4**	3	1	**6**	+	27
99d	1	1	1	1	1	1	1	1	+	8
lush	1	1	1	1	1	1	1	1	+	8
Sequences	52	30	51	45	46	39	45	47		355
Families	52	25	37	33	35	30	29	34	37	

*The abbreviation used for the species are: *B. minax* (Bmin), *B. dorsalis* (Bdor), *B. cucurbitae* (Bcuc), *B. latifrons* (Blat), *B. oleae* (Bole), *C. capitata* (Ccap), *R. zephyria* (Rzep), *D. melanogaster* (Dmel).

**Figure 1:**
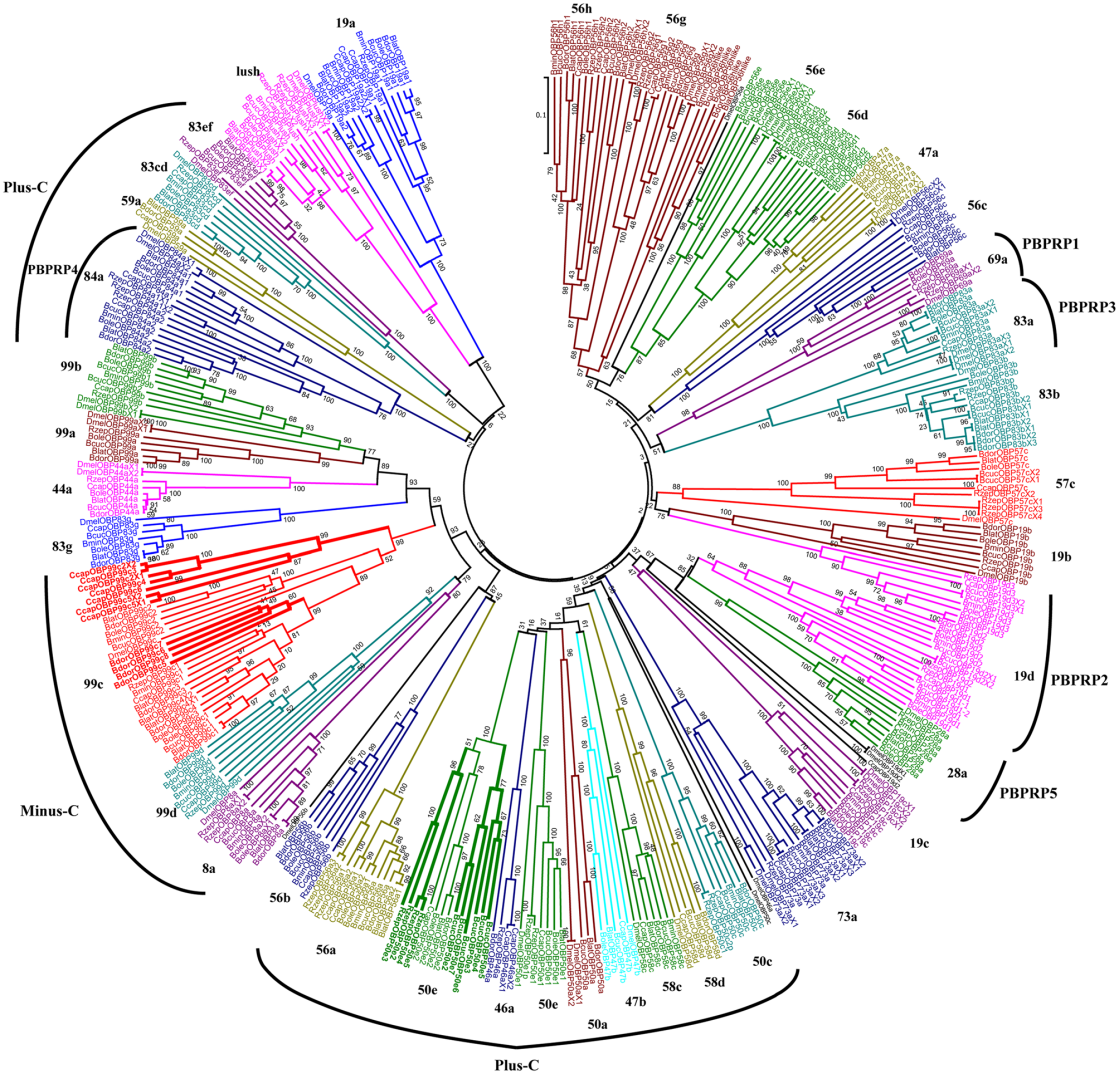
Phylogenetic relationships of OBP proteins in the selected Tephritid (NJ method). Bootstrap values greater than 50% (1000 replications) were displayed. The numbers of OBP genes present in *D. melanogaster* were previously reported^45^.

A phylogenetic tree revealed the OBPs’ evolution relationships among the 7 tephritid species and *D. melanogaster* (Fig. 1). OBP19a showed two orthologs (OBP19a1 and OBP19a2) in Tephritidae, which were clustered with DmelOBP19a (Fig. 1). OBP19d1, d2 and d3 are pheromone binding protein related proteins (PBPRPs) in the tephritids. OBP19d1 and d2 were clustered with DmelOBP19d; instead, OBP19d3 formed a separate branch near them on the phylogenetic tree. The sequence of OBP50e showed good homology to DmelOBP50e. OBP50e proteins were encoded by multi-copy genes in *B. cucurbitae* (7 copies) and in *R. zephyria* (5 copies). OBP84a (classified as PBPRP4) had two orthologs in the tephritids, which formed a branch with DmelOBP84a (Fig. 1). Similarly, OBP99c with two orthologs in the tephritids processed the species-specific multiple copies in *B. dorsalis* (8) and *C. capitata* (6) (see Table 1 and Fig. 1).

OBPs can be classified into three types (Classic, Plus-C and Minus-C) based on the their conserved cysteines^20^. Approximately 20 classic OBPs (with 6 conserved cysteines) were found in the tephritids, including 19a, 19b, 19c, 19d, 28a, 44a, 47a, 56b, 56c, 56d, 56e, 56g, 56h, 57c, 69a, 73a, 83a, 83b, 83g, 99a, 99b, and lush. Further, at least 12 Plus-C OBPs (more than 6 conserved cysteine residues) were identified in the tephritids, which were clustered in two clades on the phylogenetic tree: one cluster contained eight OBPs (46a, 47b, 50a, 50c, 50e, 56a, 58c, 58d) and another one had four (59a, 83cd, 83ef, 84a). Three Minus-C OBPs (less than 6 cysteines) were identified in the tephritids (8a, 99c, 99d), which formed an independent cluster on the phylogenetic tree. The above observations suggested that these OBPs possibly evolved in different routes in the tephritids.

**Figure 2:**
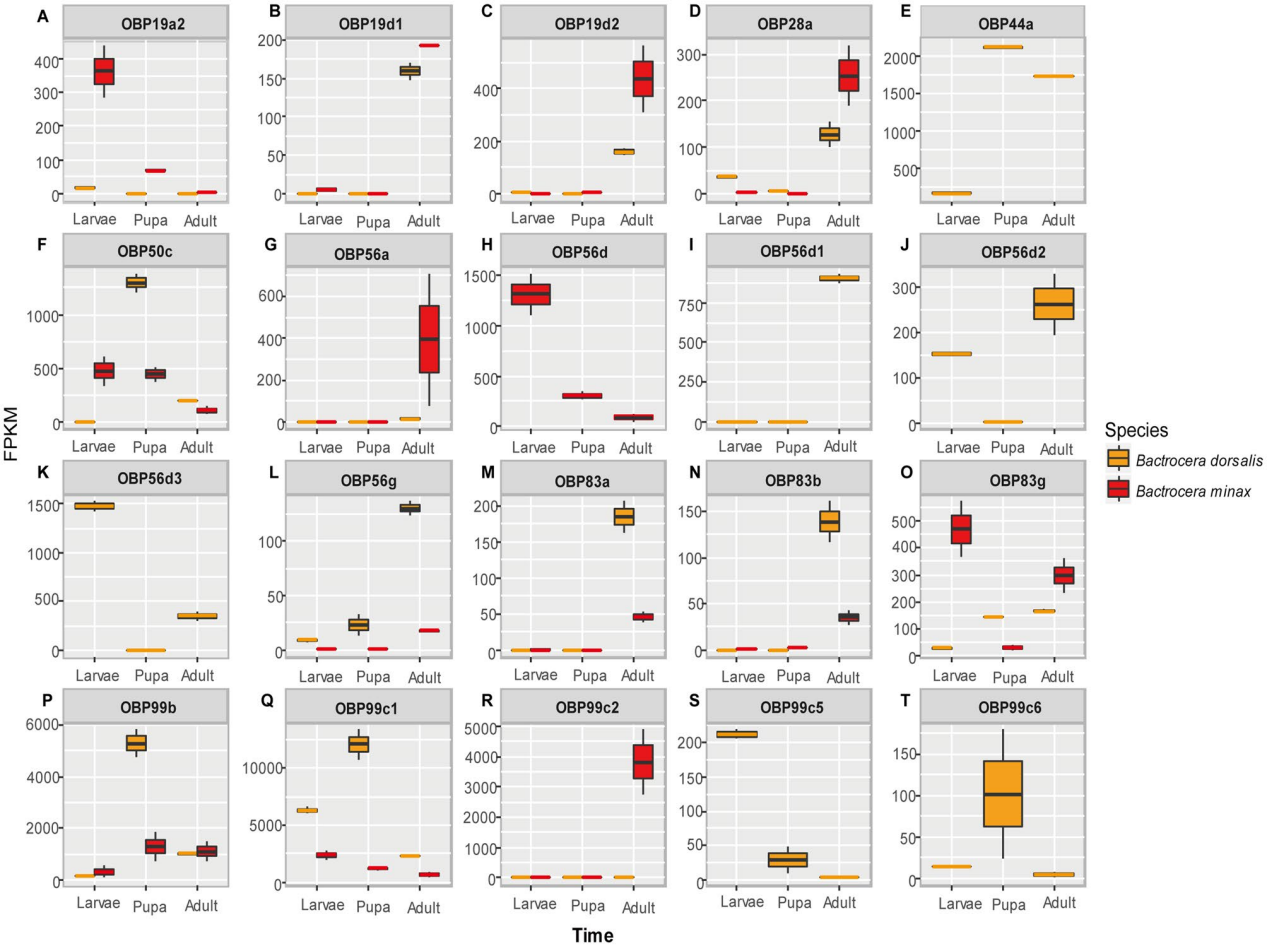
Highly expressed OBPs in *B. minax* and *B. dorsalis*. Twenty selected OBPs were shown in Fig A-T. These OBPs were chosen because of their highly expressed level (FPKM ≥ 100) in *B. minax* or *B. dorsalis*.

In the larval stage, both *B. minax* and *B. dorsalis* had six OBP genes showing a high transcription level (i.e., 99c1, 56d, 50c, 83g, 19a2 and 99b in *B. minax* as well as 99c1, 56d3, 99c5, 44a, 99b and 56d2 in *B. dorsalis,* respectively). Four (99c1, 99b, 50c and 56d) and five (99c1, 99b, 44a, 50c and 83g) OBP genes with high expression levels were found in *B. minax* and *B. dorsalis* in the pupal stage, respectively. At least seven (99c2, 99b, 99c1, 19d2, 83g, 28a, and 19d1) and fourteen (99c1, 44a, 99b, 56d1, 56d3, 56d2, 50c, 83a, 83g, 19d1, 19d2, 83b, 56g, 28a) OBP genes demonstrated high transcription levels in adult *B. minax* and *B. dorsalis*, respectively (Fig. 2). Notably, OBP99c1 was expressed at the highest levels in all developmental stages (with the exception of adult *B. minax*) for both species. The expression level of OBP99c1 peaked (FPKM = 12,091) in the pupal stage of *B. dorsalis*, suggesting its important role in the metamorphosis and/or detoxification. It is very interesting that the transcriptional level of OBP99c2 was higher (FPKM = 3823) in *B. minax* adults while it was relatively lower (FPKM = 6) in *B. dorsalis* adults, indicating that the regulation of OBP99c2 was species-specific*.* However, more studies are warranted to elucidate its physiological functions.

### Identification and gene expression of CSPs in *B. minax*

**Figure 3:**
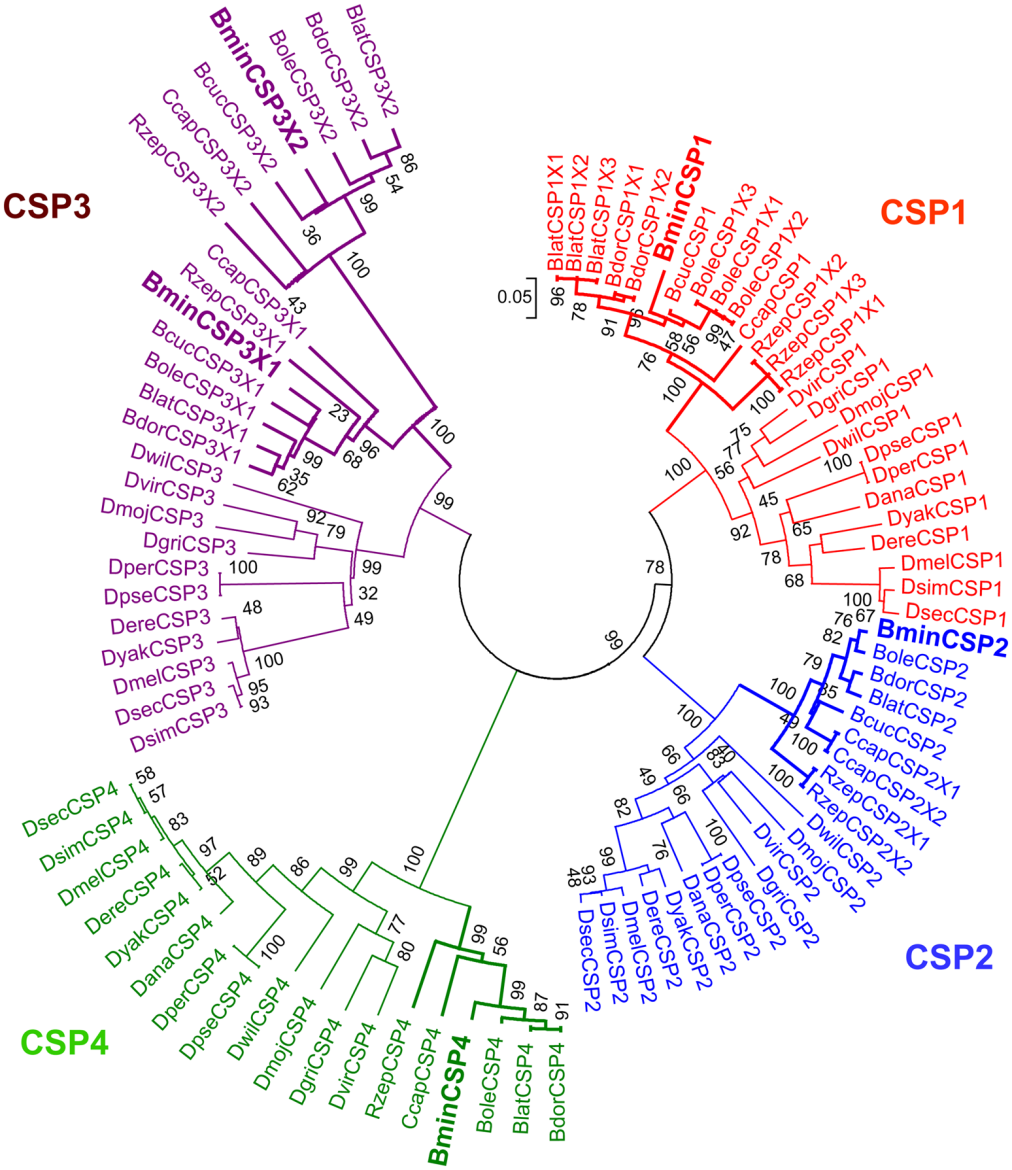
Neighbor joining (NJ) phylogenetic tree of the CSP family. The abbreviation used for the species are: The abbreviation used for the species are: *B. minax* (Bmin), *B. dorsalis* (Bdor), *B. cucurbitae* (Bcuc), *B. latifrons* (Blat), *B. oleae* (Bole), *C. capitata* (Ccap), *R. zephyria* (Rzep), *D. melanogaster* (Dmel), *D. simulans* (Dsim), *D. sechellia* (Dsec), *D. erecta* (Dere), *D. yakuba* (Dyak), *D. ananassae* (Dana), *D. pseudoobscura* (Dpse), *D. persimilis* (Dper), *D. willistoni* (Dwil), *D. mojavensis* (Dmoj), *D. virilis* (Dvir) and *D. grimshawi* (Dgri). The number of CSP genes present in *Drosophila* has been previously reported^6^.

**Figure 4:**
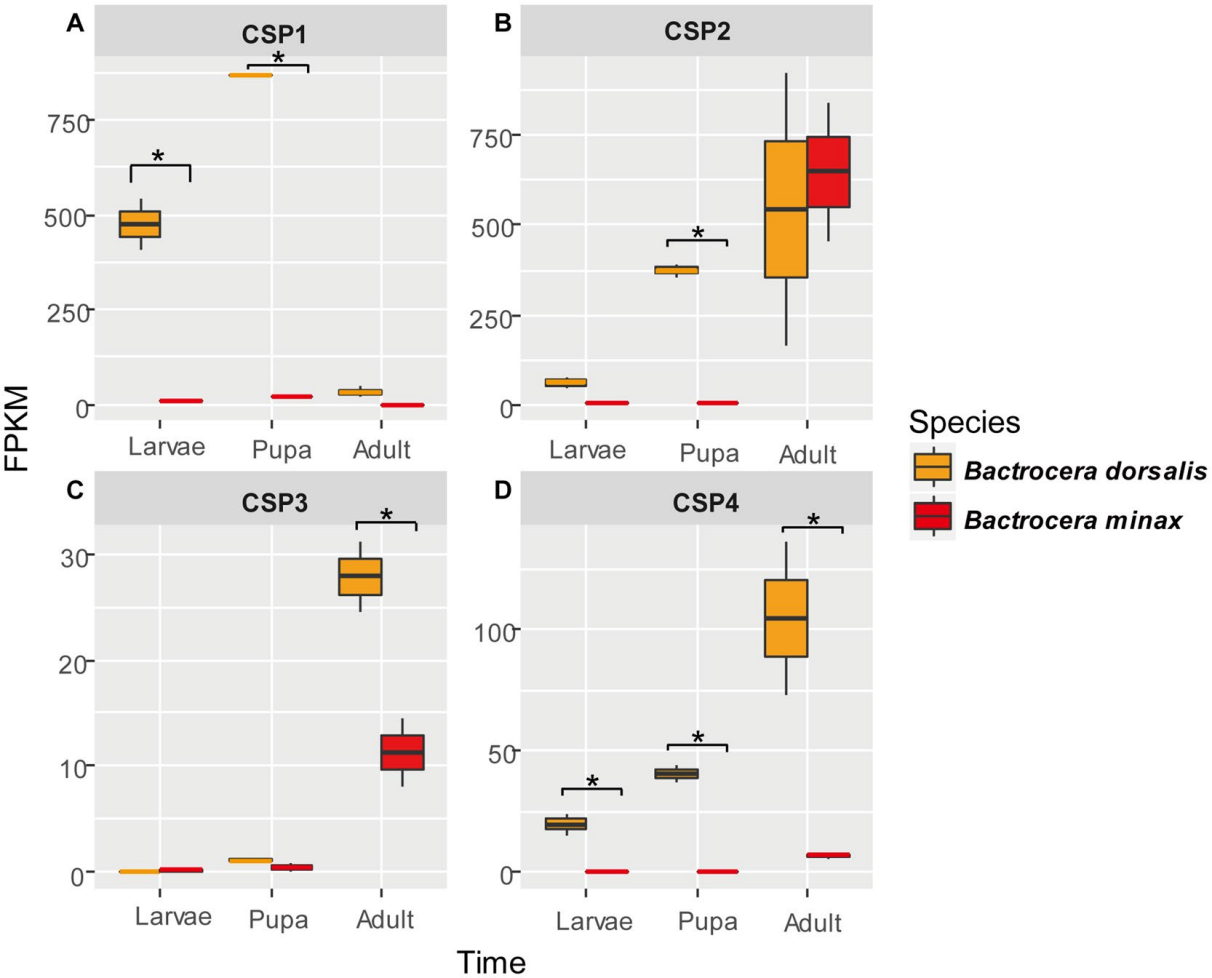
Gene expression of CSPs in *B. minax* and *B. dorsalis*. The transcriptional expression values of CSP1-4 at the three developmental stages in *B. minax* and *B. dorsalis* were shown in (**A**–**D**).

There were at least 4 genes encoding the CSP family proteins (CSP1, CSP2, CSP3, and CSP4) among all tested species in tephritids. The CSPs in *B. dorsalis* were closely related to those in *B. latifrons*, followed by *B. oleae*, *B. minax*, *B. cucurbitae* , *C. capitata* , *R.* zephyria and *D. melanogaster* (Fig. 3). There were four conserved cysteines in CSP1-4. The interspecies sequence similarity of CSPs among the tephritids was higher than that in *D. melanogaster*. All CSP3 genes of the 7 tephritid species had two splice variants, namely, CSP3X1 and CSP3X2. The average identities of CSP1, CSP2, CSP3X1, CSP3X2 and CSP4 among the tephritids were 91%, 91%, 82%, 80%, and 89%, respectively (Table 2; Supplementary file 7). However, the average degrees of similarity of CSP1, CSP2, CSP3X1, CSP3X2, and CSP4 between the tephritids and *D. melanogaster* were 64%, 73%, 50%, 28%, and 66%, respectively (Table 2). The gene encoding CSP1 was expressed at the highest transcriptional level in the pupae stage for both *B. minax* and *B. dorsalis*. However, the gene encoding CSP2-4 showed a higher transcriptional level than CSP1 in the adult stages. Further, the transcript level of CSP3 and CSP4 was lower in adult *B. minax* than that in *B. dorsalis* (Fig. 4).

**Table 2:** Sequence similarity of CSPs in Tephritidae.

	CSP1 (%)	CSP2 (%)	CSP3X1 (%)	CSP3X2 (%)	CSP4 (%)
Intra-Tephritidae	91	91	82	80	89
Tephritidae and *D. melanogaster*	64	73	50	28	66

### Identification and gene expression of ORs in *B. minax*

**Figure 5:**
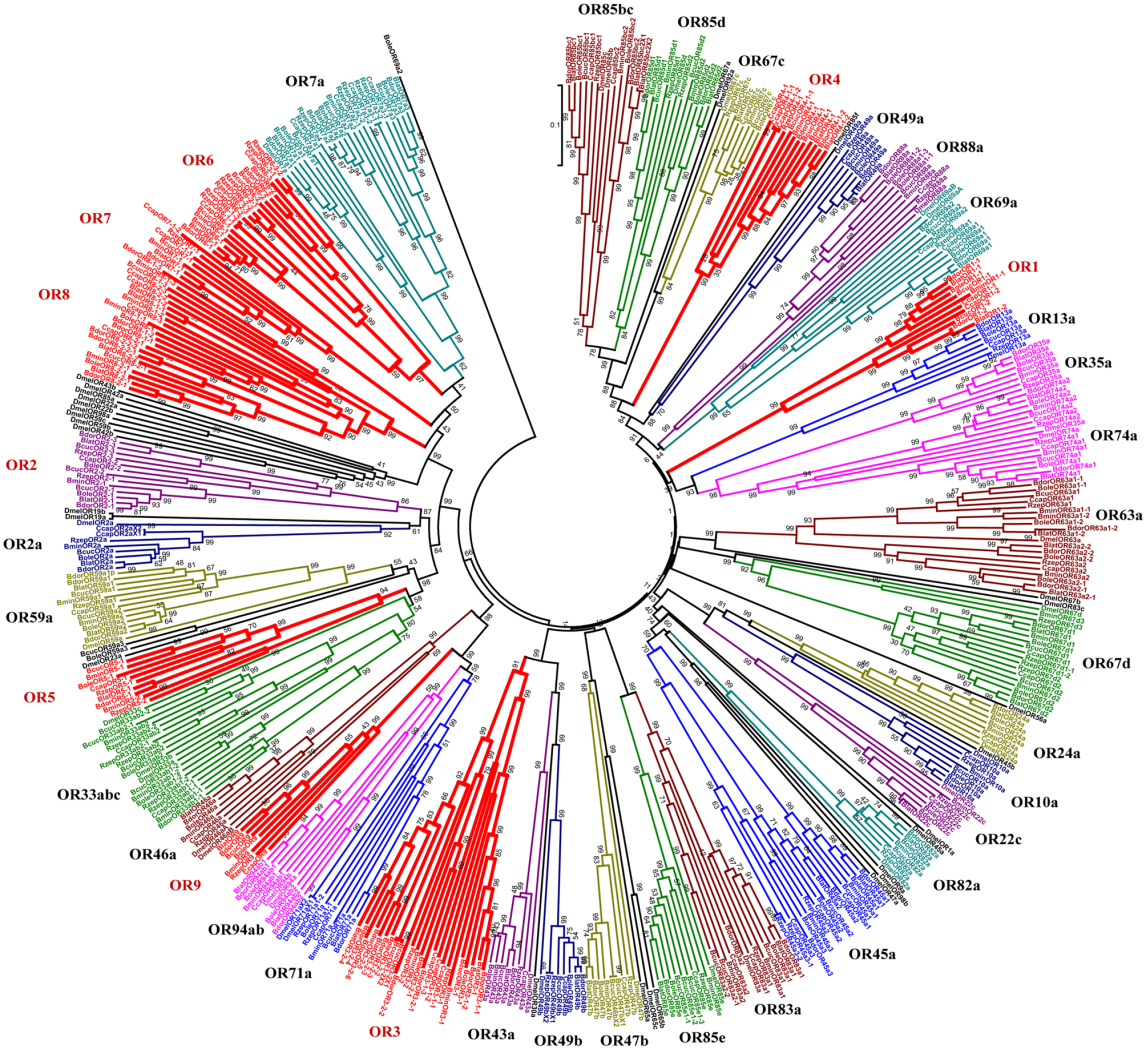
NJ tree of the OR family in Tephritidae. NOTE: The number of OR genes present in *D. melanogaster* has been previously reported^20^.

To search for candidate OR genes in *B. minax*, the sequence similarity analysis and the phylogenetic tree construction were performed. As shown in Table 3, 53, 70, 61, 58, 59, 64, and 61 OR genes were identified in *B. minax*, *B. dorsalis*, *B. cucurbitae*, *B. latifrons*, *B. oleae*, *R. zephyria*, and *C. capitata*, respectively. 35 of 62 ORs from *D. melanogaster* were present in these tephritids (Table 3). 14 ORs (7a, 33abc, 45a, 59a, 63a, 67d, 69aA/B, 74a, 83a, 85bc, 85d, 85e, 88a and 94ab) showed gene duplications in the tephritids (Table 3). For example, OR85e and OR88a had 3 copies in Bcuc and in Bole, respectively (Fig. 5). In addition, nine OR genes specific to the tephritids were identified, which were individually named OR1, OR2, OR3, OR4, OR5, OR6, OR7, OR8 and OR9. Except OR9, these ORs all presented gene duplications; for example, OR3 had 8 copies in *B. dorsalis*.

**Table 3:** Identification of OR genes in Tephritidae*.

	Dmel	Bmin	Bdor	Bcuc	Blat	Bole	Rzep	Ccap	Total
**Nonclassical olfactory receptor**									
83b (ORCO)	1	1	1	1	1	1	1	1	8
**Typical olfactory receptor ORs**									
2a	1	1	1	1	1	1	1	1	8
7a	1	2	3	3	3	3	6	4	25
10a	1	1	1	1	1	1	1	1	8
13a	1	0	1	1	1	1	1	1	7
22c	1	1	1	1	1	1	1	1	8
24a	1	1	1	1	1	1	1	1	8
33abc	3	3	1	4	0	2	5	2	20
35a	1	1	1	1	1	1	1	1	8
43a	1	1	1	1	1	1	1	1	8
45a	1	2	3	2	2	3	3	3	19
46aA/B	2	1	1	0	1	1	1	1	8
47b	1	1	1	1	1	1	1	1	6
49a	1	1	1	1	1	1	1	1	8
49b	1	0	1	1	1	1	1	1	7
59a	1	2	2	3	2	2	1	1	14
63a	1	3	4	1	3	4	2	2	20
67c	1	1	1	1	1	1	1	1	8
67d	1	3	2	2	2	2	4	2	18
69aA/B	2	0	2	2	1	2	2	2	13
71a	1	1	1	1	1	1	2	1	9
74a	1	2	2	2	2	2	2	2	15
82a	1	1	1	1	1	1	1	1	8
83a	1	2	2	3	1	1	1	2	14
85bc	2	1	2	1	2	2	1	2	13
85d	1	2	2	2	2	1	2	0	12
85e	1	1	1	3	1	1	1	1	12
88a	1	1	1	1	1	3	1	1	10
94a/b	2	2	3	1	3	3	1	2	17
Common Diptera ORs	35	39	45	44	40	46	48	41	339
**Tephritidae-specific ORs**	0	14	25	17	18	13	16	20	87
OR1	0	1	2	2	2	1	0	2	10
OR2	0	1	2	3	2	2	2	1	13
OR3	0	2	8	3	3	3	2	3	24
OR4	0	1	2	2	2	1	1	3	12
OR5	0	2	1	1	1	1	2	1	9
OR6	0	1	2	2	2	2	7	3	19
OR7	0	1	1	1	1	0	1	2	7
OR8	0	4	6	2	4	2	0	4	22
OR9	0	1	1	1	1	1	1	1	7
Total Diptera ORs	62	53	70	61	58	59	64	61	426

*The abbreviation used for the species are: *B. minax* (Bmin), *B. dorsalis* (Bdor), *B. cucurbitae* (Bcuc), *B. latifrons* (Blat), *B. oleae* (Bole), *C. capitata* (Ccap), *R. zephyria* (Rzep), *D. melanogaster* (Dmel).

The copy numbers of OR families were much higher than those of OBP and CSP families^7^. We further identified 21 putative ORs in the tephritids with their ancestral nodes (here named as 2a, 7a, 10a, 13a, 22c, 24a, 33abc, 43a, 45a, 46a, 47b, 49a, 49b, 59a, 67c, 67d, 69a, 71a, 82a, 85e, and 88a). Six genes in the tephritids were identified with two orthologs, which were distributed in two distinct clades together with *D. melanogaster* OR genes. This observation suggested that these ORs maybe have appeared earlier in the tephritids than those in *D. melanogaster* (63a, 74a, 83a, 85bc, 85d and 94ab). Nine OR genes (OR1-9) were conserved in *D. melanogaster* and in the tephritids. These results showed that the OR gene family possibly underwent rapid evolution with a large amount of variations. The exception of the OR46a1 gene exhibited moderate expression between FPKM 8.1 and 13.4 in the larval stage of *B. minax*. However, most OR genes showed a relative lower level at the three developmental stages in both species (Supplementary Table S5).

### Validation of RNA-Seq data by qRT-PCR analysis

**Figure 6:**
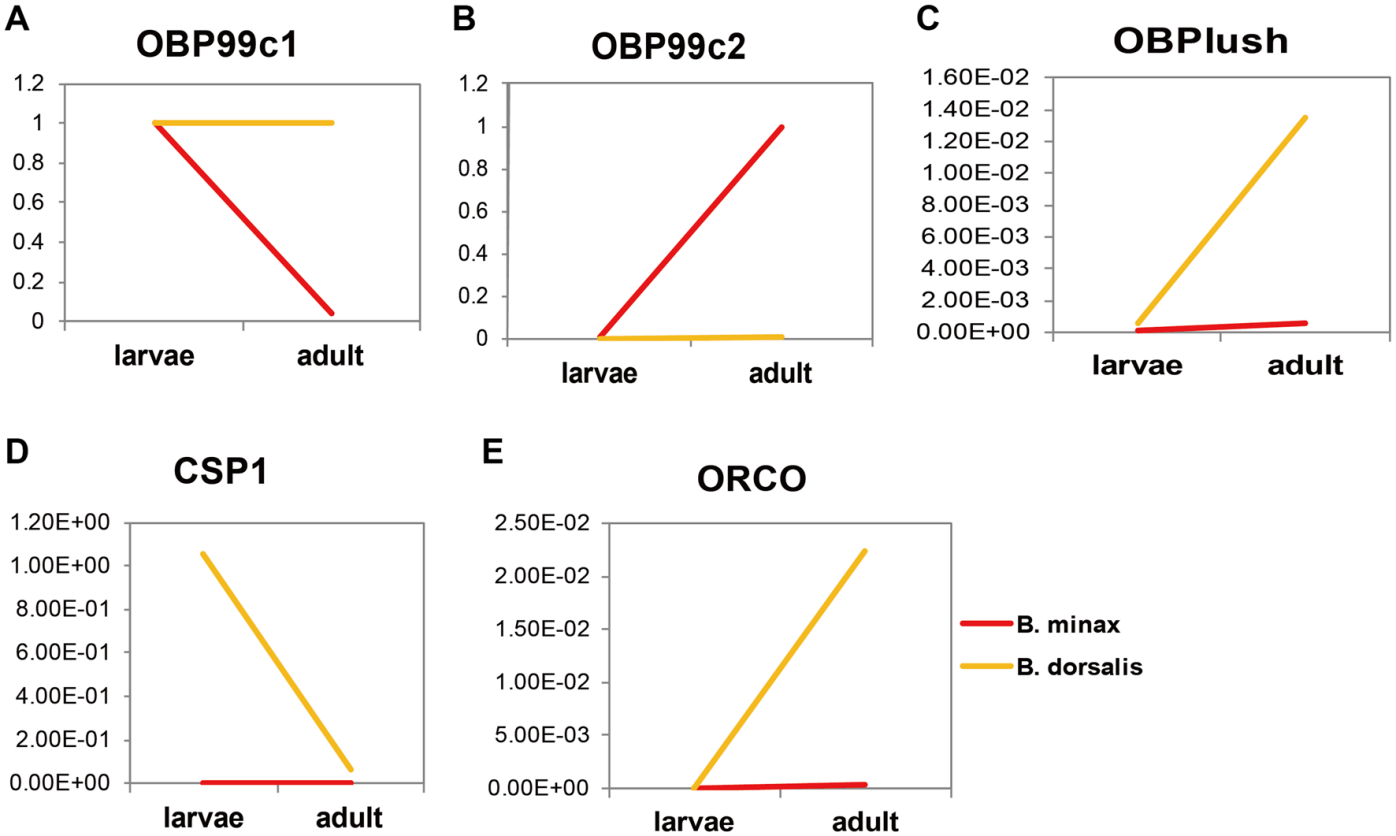
The qRT-PCR verification of gene expression. Five genes, including OBP99c1, OBP99c2, OBPlush, CSP1 and ORCO, were selected and their gene expressions were validated using qRT-PCR from *B. minax* and i in the larvae and adult stages.

The expression of five selected genes including *OBP99c1*, *OBP99c2*, *OBPlush*, *CSP1*, and *ORCO* were validated using qRT-PCR from *B. minax* and *B. dorsalis* in the larvae and adult stages (Fig. 6). Coefficient analysis showed that qRT-PCR data of the 5 selected genes were significantly correlated with the RNA-Seq results (r = 0.91 in *B. minax* and r = 0.95 in *B. dorsalis*; Supplementary file 8), which indicated that the RNA-Seq data in the present study were reliable and could support the transcriptomic analysis presented above. For example, the results from qRT-PCR also showed that the transcriptional level of *OBP99c1* was high at the larvae stage but relatively low in the adult stage in *B. minax. OBP99c2* was highly expressed in *B. minax* adults, indicating that *OBP99c2* may be an important gene for sensing chemicals in adult *B. minax.*

## Discussion

Insects recognize their hosts mainly via the insect chemosensory systems (i.e. OBPs, CSPs and ORs)^6^. For example, *B. minax* Enderlein has a restricted host range and almost always lays eggs in *Citrus* fruits^1^; however, *B. dorsalis* Hendel infests over 200 different fruits and vegetables^21^. Identification and characterization of the insect chemosensory system will contribute to the development of novel biocontrol methods for targeting the specific agricultural pests such as *B. minax*. Wu et al.^10^ investigated the transcriptome profiles in *B. dorsalis*. At least 31 OBPs, 4 CSPs and 23 ORs were found in *B. dorsalis*^10^. However, Liu et al. (2016) reported that there were 20 OBPs, 5 CSPs and 35 ORs in male and female adults of *B. dorsalis*^13^. Both studies likely underestimate the insect chemosensory systems due to the limitation of the RNA-seq technology (transcriptome coverage, low abundance of the genes, or the chosen studied sites).

We first identified up to 25 OBP, 4 CSP and 53 OR genes in the genome of *B. minax* using comparative genomic approaches. Several chemosensory proteins identified in this study were not previously reported. Remarkably, we discovered at least 37 OBP, 4 CSP, and 70 OR genes in the genome of *B. dorsalis*. Moreover, the comparative genomic analyses allowed us to identify at least 33 OBPs, 4 CSPs and 61 ORs in the genome of *B. cucurbitae* (Supplementary files 3–6). Only 17 OBPs were previously reported in the Mediterranean fruit fly, *Ceratitis capitata*, from the EST libraries of adult heads, embryos, male accessory glands and testes^22^. Instead, we found 34 OBP, 4 CSP and 61 OR genes in the genome of *C. capitata* (Supplementary files 3–6). Collectively, our discoveries significantly extend the chemosensing-related gene profiles (particularly, OBPs and ORs) in these agriculture important pests.

Compared to those in *B. dorsalis*, the less OBPs and ORs in *B. minax* possibly related to its narrower plant host ranges^23^. However, this hypothesis needs to be further tested within the context of the generalized or specific binding properties of OBPs^7^. At the adult developmental stage, 14 OBP genes were highly expressed (FPKM > 100) in *B. dorsalis* while only 7 OBP genes were highly expressed in *B. minax*. *B. dorsalis* may utilize more OBPs to perform the odorant and pheromone binding functions than those in *B. minax.* Among them, it is worthy of highlighting that gene *OBP99c1* was highly expressed in all the developmental stages in both species. However, *OBP99c1* exhibited a gradually declining trend during *B*. *minax* development, suggesting that it may play a crucial role in the odorant-binding in the citrus fruit fly. The gene *OBP99c1* in *B. dorsalis* (named *BdorOBP10*) was highly expressed in females, especially in the abdomen, where the reproductive organs are located^24^. Interestingly, the OBP10 protein of two sibling Lepidopteran species, *Helicoverpa armigera* and *Helicoverpa assulta*, was detected first in the male reproductive system, then in females during mating, and eventually in eggs^25^. In addition, OBP10 exhibited binding to an insect repellent, indicating that this protein may be a carrier for some semiochemicals^25^. Therefore, *OBP99c1* may be a good target for the design of novel pest control agents. On the other hand, *OBP99c2* was found to be highly expressed in *B. minax* adults*,* but it was barely expressed in *B. dorsalis*. *OBP99c2* may play a species-specific role in the development of odorant-binding function. However, further study is warranted to elucidate the physiological functions of *OBP99c2*.

The number of conventional ORs varies dramatically among different insect species. For example, the genome of honey bee *Apis mellisfera* contains 170 OR genes. However, *D. melanogaster* and *Anopheles gambiae* only carry 62 and 79 OR genes, respectively^26^. Our study showed that the numbers of ORs in 6 selected Tephritidae ranged from 53 to 70. Unlike the relatively high expression levels of OBPs and CSPs in *B. minax* and *B. dorsalis*, the expression level in OR genes was considerably low during the development progress, which was consistent with the previous observations^10^. However, Liu et al. (2016) reported that two OR genes (*Bdoror 13* and *Bdoror 14*) in *B. dorsalis* were specifically expressed at a high level in the male antennae^27^. The discrepancy may be caused by the different selection of study sites (whole body vs tissues) or different investigation approaches (qPCR vs RNAseq)^10^.

The protein sequence similarity among CSPs among the selected insects was high. For example, CSPs of *Schistocerca gregaria* and *Locusta migratoria* share 50–60% sequence identity^28^. We found that the sequence similarities of CSPs among Tephritidae were higher than those between Tephritidae and *D. melanogaster*. CSPs were known to play a key role in olfactory perception. Therefore, CSPs have been used to screen potential bioactive compounds for pest management^29^. Notably, insecticides have been shown to significantly up-regulated adult-specific CSP1 gene expression in the sweet potato whitefly *Bemisia tabaci*^13^ (Liu et al., 2016). Additionally, in whitefly, due to the ligand binding specificity, CSP1 may be responsible for regulation of the insect immune response mediated by fatty acids, while CSP2 and CSP3 facilitate insect communication with the surrounding environment via favorable or unpleasant odors^13^. A recent study indicated that, in *Bradysia odoriphaga* (Diptera: Sciaridae), tissue-specific enrichment of CSP4 (in both the antennae and heads) and CSP1/CSP2 (in the legs and heads) may be involved in other crucial physiological functions of this insect^30^. In fact, the honeybee (*Apis mellifera*) exhibited abnormal head development upon loss of CSP5 function and could not turn into the larval stage^31^. Therefore, further investigation of the tissue distribution of the CSPs identified in this study may facilitate the functional analysis of these genes.

In conclusion, we identified the chemosensing-related genes of the citrus fruit fly *B. minax* based on genome data and identified 82 candidate chemosensing-related genes, including 25 OBPs, 4 CSPs and 53 ORs. Our study compared the genetic relationships of candidate genes among 7 species and showed that *B. minax* had the least numbers of OBPs and ORs. Based on the transcriptomes of three developmental stages (larvae, pupae and adults) of *B. minax* and *B. dorsalis*, the expression profiles of candidate OBPs, CSPs and ORs were compared and demonstrated that almost all the OBPs and the CSPs presented more highly transcriptional expression values in *B. dorsalis* than in *B. minax.* These findings suggested that *B. dorsalis* may exhibit more powerful odorant and pheromone binding properties than *B. minax*, which could be related to the fact that *B. dorsalis* targets a wider range of host species than *B. minax*.

## Materials and methods

### Insect rearing and ethics statement

#### B. minax

*B. minax* insects were collected and identified by personnel of the Hunan Academy of Agricultural Sciences in Jishou City, Hunan Province, China. Larvae were reared in tangerines and pupae were buried in wet soil. Larvae were collected at the 3^rd^ instar, and pupae at 20 days after pupation. The adults were collected 12 h after emergence without any feeding or sexually matured.

#### B. dorsalis

*B. dorsalis* insects were reared in the laboratory at 25 ± 1 °C under a 16:8 h light:dark photoperiod and 70–80% relative humidity (RH). Artificial diets for the larvae were provided by the Institute of Insect Ecology of South China Agricultural University, and consisted of banana, corn flour, sucrose, yeast extract, paper, sodium benzoate, hydrochloric acid and water in appropriate proportions^32,33^. Larvae developed into pupae in wet sand. Artificial diet for adults was a mixture (1:1) of yeast extract and sucrose^34^. Larvae were collected 6 days after incubation at the 3rd instar. Pupae were collected 6 days after pupation. Adults were collected 12 h after emergence without any feeding and before sexual maturation.

### Sample preparation and genome sequencing

Body parts of single *B. minax* males were dissected to obtain DNA for whole-genome sequencing. Total DNA was extracted using the Magen HiPure Insect DNA Kits (D3129-02) (Guangzhou, China) according to the manufacturer’s instructions. Samples were sent to the GeneDenovo company (Guangzhou, China) (https://www.genedenovo.com/) for library construction and genome sequencing. Genome sequencing was carried out with the combination of Next-generation sequencing (NGS) using the Hiseq 2500 and third-generation sequencing adopting the Pacbio RSII. Briefly, five libraries were constructed, making two duplicated short fragment libraries (450 bp + 800 bp) and three long mate-pair libraries (2 kb + 5 kb + 10 kb), which produced in total 102G nucleotide bases (Supplementary file 1, Table S1-1) and covered an estimated 300 × of the genome size (Supplementary file 1, Figure S1-1). The genome size was estimated at about 331 Mb based on Kmer (k = 17) short fragmentary libraries analysis (Supplementary file 1, Figure S1-2). For third-generation sequencing, two libraries were constructed, five SMRT cells were sequenced, total 5G raw data were obtained, and the genome coverage reached 15 × (Supplementary file 1, Table S1-2); and SMRT analysis software (version 2.3.0) (https://www.pacb.com/products-and-services/analytical-software/smrt-analysis/) provided from Pacbio was used for the sequencing quality control. The genome assembly was divided into two steps: (1) Platanus (version1.2.1)^35^ was used to assemble Illumina data and GapCloser (v1.10)^36^ was utilized to extend the contig length; (2) PBjelly (PBSuite_15.8.24)^37^ was used to extend the scaffolds and fill the gaps by combining the third-generation sequencing data. Totally, we obtained a genome size of 340 Mb, which is close to the estimated genome size. The numbers of contigs/scaffolds were 38,509/8019 and the contig/scaffold N50 reached 23 kb/1.6 Mb (Supplementary file 1, Table S1-3). The genome sequences have been submitted to NCBI (SAYV00000000). The repetitive sequences account for 20.97% of the genome with using de novo prediction (RepeatModeler^38^ and LTR-FINDER^39^), RepBase^40^-based homology prediction (RepeatMasker^41^ and RepeatProteinMask^42^), and tandem repeats finder (TRF^43^) (Supplementary file 1, Table S1-4 and Table S1-5). Several methods of de novo prediction, homology-based gene prediction, and cDNA/EST prediction were used to predict gene structure after excluding the repetitive sequences (Supplementary file 1, Figure S1-3). Softwares from three systems (Augustus 2.7^44^, Genscan 1.0^45^, and Glimmer HMM 3.0.1^46^) were used for de novo prediction of gene models. MAKER 2.28 software^47^ was applied to integrate all gene sets into a non-redundant and more complete one. Lastly, the BUSCO method^48^ was used to estimate the reliable degree of gene models, and the complete single-copy BUSCO scores reached 98.5% in the assembled gene set and 94.9% in the annotated gene set, respectively (Supplementary file 1, Table S1-6). After all these analyses, a set of 12,533 gene models was obtained and used for identification of chemosensory genes.

### Identification of chemosensory genes

The protein sequences from *B. dorsalis* (Bdor), *Bactrocera cucurbitae* (Bcuc), *Bactrocera latifrons* (Blat), *Bactrocera oleae* (Bole), *Ceratitis capitata* (Ccap), and *Rhagoletis zephyria* (Rzep), all Diptera, family Tephritidae, were downloaded from NCBI (ftp://ftp.ncbi.nlm.nih.gov/genome). Protein sequences from the above species that were annotated as chemosensation-related genes were extracted. The protein sequences from *B. minax* were aligned to the protein sequences annotated as chemosensation-related genes from other 6 species by using BLASTP with an e-value setting of 1e-5. From this analysis, the possible chemosensation-related genes of *B. minax* were obtained. The sequences of fifty-one OBP genes and one lush gene from *Drosophila melanogaster*^49^ and those of 31 OBP genes from *B. dorsalis* were obtained. The sequences of forty-seven CSP genes from 12 *Drosophila* species^6^ and those of four CSPs (CSP1-4) from *B. dorsalis* were acquired. The sequences of sixty-two OR genes from *D. melanogaster*^17^ and those of 23 OR genes from *B. dorsalis*^12^ were obtained. Then the potential chemosensation-related genes of *B. minax* and the protein sequences annotated as chemosensation-related genes from the other 6 tephritid species were aligned to the collected gene sets (83 OBPs, 51 CSPs and 85 ORs) by using BLASTP with an e-value setting of 1e-5. According to sequence similarity of more than 40% and manual inspection: (1) multiple consistent hits to directly classify this one group; (2) multiple inconsistent hits to take the one with high similarity, the potential or annotated chemosensation-related genes were assigned to each known chemosensory gene. Other genes that were difficult to assign were classified as indeterminate genes, including the genes annotated as chemosensation-related genes but their sequence similarity lower than 40% to the collected gene sets.

### Sequence comparison and phylogenetic analysis

The tool ClustalX^50^ was used to execute multiple sequence alignment with default parameters, and GeneDoc^51^ was used to visualize the alignment. Highly divergent sequences which by visual inspection had no common sites with others were moved to the indeterminate gene category. Then, the sequence alignment was input into MEGA6^52^; the NJ (neighbor-joining) method with the pairwise deletion option and the maximum likelihood method with partial deletion option were selected to construct phylogenetic trees (to see supplementary file 2). Robustness of the branches was assessed with 1000 bootstrap pseudo-replicates. In this manner, the determinate chemosensory genes in the 7 species were obtained. Lastly, all identified chemosensory genes and the indeterminate genes were combined to carry out sequence comparison and phylogenetic analysis. Some highly divergent or short sequences, reported by MEGA6 to affect the phylogenetic tree construction, were deleted. The gene names for *B. minax* and the six other species were modified according to the phylogenetic clusters.

### Sequence information

The OBP, CSP and OR family sequences from *B. minax* have been submitted to GenBank with accession numbers MH937211–MH937240, MH937207–MH937210, MH937241–MH937290. The NCBI reference sequence and GenBank accessions of OBPs, CSPs and ORs in all 7 tephritid species are provided in the supplementary file 3. Protein sequences and alignments of each OBP/CSP/OR member with those from other species are presented in supplementary files 4, 5 and 6, respectively.

### RNA sequencing and transcript analysis

Total RNA was extracted from two *B. minax* individuals and four *B. dorsalis* individuals at the development of larvae, pupae and adult using TRIzol reagent (Invitrogen, California, USA). The adult samples were equal numbers of females and males. Two biological replicates per developmental stage were used to construct the cDNA libraries using the Illumina TruSeq RNA Sample Preparation kit and the mixed libraries were sequenced on one lane of Illumina Hiseq 4000 platform with paired-end 150-bp reads. Raw paired-end reads from all samples were submitted to the Sequence Read Archive of the NCBI (*B. minax*: SRP193917 and *B. dorsalis*: SRP193924). Clean reads were obtained by Perl scripts: (1) the adapters were removed and the reads, which were shorter than 50 nt and including adapters, were also removed; (2) the reads with a percentage of low-quality bases (lower than Q20) more than 40% were filtered; (3) the reads containing more than 10% N were excluded. The number of clean reads in all samples ranged from 22 to 32 million (Supplementary Table S1). Clean reads from different species were mapped to the genome sequences of the corresponding species using Bowtie2^53^ and Tophat2^54^ programs with default parameter values, which generated an average of 72% mapped ratio in all samples (Supplementary Table S1). The Cufflinks program^55^ was used to calculate the FPKM values (fragments per kilobase of exon model per million mapped reads) for determination of gene expression levels. The expression dataset including 12,533 predicted gene from *B. minax* and 13,121 predicted gene from *B. dorsalis* was listed in Supplementary Table S2. Among them, the transcript information of these identified OBPs, CSPs and ORs was specifically extracted to evaluate their gene expression level. The Student’s *t* test method without having to rely on the total input genes was suitable for cross-species comparisons of expression of OBPs, CSPs and ORs. Compared *B. minax* to *B. dorsalis* at the same development stage, genes were deemed significantly differentially expressed after setting corrected p-value ≤ 0.05 and the relative change threshold ≥ 2-fold (to see Supplementary Table S3–S5). The genes with FPKM values greater than 100 were considered to be highly expressed.

### Validation of gene expression by qRT-PCR

The expression of 5 genes from *B. minax* and *B. dorsalis* at the development of larvae and adult were validated using qRT-PCR, including *OBP99c1* (Bmi011415 and gene11468/BdorOBP10_hz2013/AKI29023.1/AGC82131.1/AGS08192.1/XP_011210420.1), *OBP99c2* (Bmi011416 and gene11474/AKI29026.1/AGC82130.1/XP_011210427.1), *OBPlush* (Bmi007112 and gene9962/AKI28996.1/XP_011208040.1), *CSP1* (Bmi004355 and gene876 /AKI28975.1/ XP_011204433.1), *ORCO* (Bmi011214 and gene7268/AKI29027.1/JAC55447.1/XP_011203778.1). Two genes, GAPDH and A-tub, were selected from non-DEGs in the RNA-Seq dataset respectively as candidate internal reference genes of *B. minax*^56^ and *B. dorsalis*^57^. Primers were designed on the online website NCBI Primer-BLAST (https://www.ncbi.nlm.nih.gov/tools/primer-blast/) according to the coding sequences (CDS) of each gene and their corresponding transcriptome. Two pairs of primers per gene recommended by NCBI were selected and synthesized. To be consistent with RNA-Seq information, two *B. minax* individuals and four *B. dorsalis* individuals at the development stage of larvae and adult (male:female = 1:1) were respectively used to extract total RNA as a biological replicate. Three biological replicates per sample were performed. The ages of the samples from *B. minax* and *B. dorsalis* were the same as that of the samples used for RNA-Seq, except the samples from pupa stage were not picked due to seasonal reasons. Complementary DNA was synthesized using the FirstKing cDNA Strand Synthesis Kit (KR180123) (Beijing TianGen biological co., LTD., China). Through gel imaging observation, one pair of better primers were selected for each target gene. Information on the primers is listed in Supplementary Table S6. qRT-PCR was performed with three technical replicates on a CFX-96_CFX Connect Real-Time PCR System (Bio-Rad, American). The relative expression levels of the 4 selected DEGs normalized to the expression level of the internal reference control were calculated using the 2^−ΔΔCt^ method^58^, and coefficient analysis was carried out to evaluate the correlation between the qRT-PCR and the RNA-Seq results.

## Supplementary information

Supplementary Information 1.

Supplementary Information 2.

Supplementary Information 3.

Supplementary Information 4.

Supplementary Information 5.

Supplementary Information 6.

Supplementary Information 7.

Supplementary Information 8.

Supplementary Information 9.

## Data Availability

All data generated or analysed during this study are included in this published article (and its Supplementary Information files).
