# Supplementary material for: Comparative genomic and transcriptomic analyses of chemosensory genes in the citrus fruit fly Bactrocera (Tetradacus) minax
Source: Sci Rep. 2020 Oct 22;10:18068. doi: 10.1038/s41598-020-74803-5 (PMC7583261; doi:10.1038/s41598-020-74803-5)
Supplement: Supplementary file 2 — Supplementary Information 2. [file 41598_2020_74803_MOESM2_ESM.pdf]

Supplementary file 1

Comparative genomic and transcriptomic analyses of chemosensory genes in the citrus fruit fly *Bactrocera* (*Tetradacus*) *minax*

Jun-Feng Cheng[1,2#], Ting Yu[2#], Zhong-Jian Chen[2#], Shicheng Chen[3#], Yu-Peng Chen[1#], Lei Gao[4],

Wen-Hu Zhang[2], Bo Jiang[5], Xue Bai[1], Edward D. Walker[3], Jun Liu[2*], and Yong-Yue Lu[1*]

Table S1-1 The output statistics of raw data sourcing from the next-generation sequencing.

Insert size	Reads length (nt)	Reads number	Total Base	Coverage (X)
**450**	250	35,324,047	17,662,023,500	53.5
**450**	250	34,859,811	17,429,905,500	52.8
**800**	250	34,942,335	17,471,167,500	52.9
**800**	250	40,213,945	20,106,972,500	60.9
**2000**	48	125,837,121	12,080,363,616	36.6
**5000**	48	118,601,140	11,385,709,440	34.5
**10000**	48	61,267,928	5,881,721,088	17.8
**Total**		451,046,327	102,017,863,144	309

Table S1-2 The statistics of the raw and filtered data sourcing from the third-generation sequencing.

Metrics	Pre-Filter	Post-Filter
**Polymerase Read Bases**	5,047,245,749	4,043,252,637
**Polymerase Reads Number**	751,460	337,083
**Polymerase Read N50**	16,083	16,938
**Polymerase Read Mean Length**	6,716	11,994
**Polymerase Read Quality**	0.46	0.847

Table S1-3 The statistics of the genome assembly.

	Scaffold	Contig
Total Number (#):	8,019	38,509
Total length (bp):	341,841,136	333,856,021
Gap(N)(bp):	7,985,115	0
Average Length (bp):	42,628.90	8,669.56
N50 Length (bp):	1,636,139	22,655
N90 Length (bp):	23,412	4251
Maximum Length (bp):	7,301,009	640,561
Minimum Length (bp):	1,000	25
GC content :	34.59%	34.59%

Table S1-4 Statistical results of repetitive sequences.

Type	Repeat Size(bp)	% of genome
TRF	5,550,077	1.45
RepeatMasker	17,752,458	4.64
RepeatProteinMask	11,055,979	2.89
De novo	62,612,105	16.35
Total	80,279,226	20.97

Table S1-5 Statistics of classification results of repetitive sequences.

	**RepBase TEs**		**TE Proteins**		***De novo***		**Combined TEs**	
	**Length (bp)**	**%in Genome**	**Length (bp)**	**% in Genome**	**Length (bp)**	**% in Genome**	**Length (bp)**	**% in Genome**
DNA	12,526,609	3.27	6,354,499	1.66	31,301,419	8.18	40,805,167	10.66
LINE	2,816,805	0.74	2,199,454	0.57	7,075,663	1.85	9,677,563	2.53
SINE	10,093	0.00	0	0.00	0	0.00	10,093	0.00
LTR	2,821,927	0.74	2,520,332	0.66	18,792,559	4.91	22,125,854	5.78
Other	1,082	0.00	0	0.00	0	0.00	1,082	0.00
Unknown	92,578	0.02	0	0.00	14,501,388	3.79	14,593,660	3.81
Total	17,752,458	4.64	11,055,979	2.89	61,879,122	16.16	76,603,768	20.01

Table S1-6 The estimation of BUSCO.

	Assembly		Annotation	
	Proteins	Percentage	Proteins	Percentage
Complete Single-Copy BUSCOs	1050	98.50	1012	94.93
Complete Duplicated BUSCOs	5	0.47	9	0.84
Fragmented BUSCOs	2	0.19	28	2.63
Missing BUSCOs	9	0.84	17	1.59
Total BUSCO groups searched	1066	100.00	1066	100.00

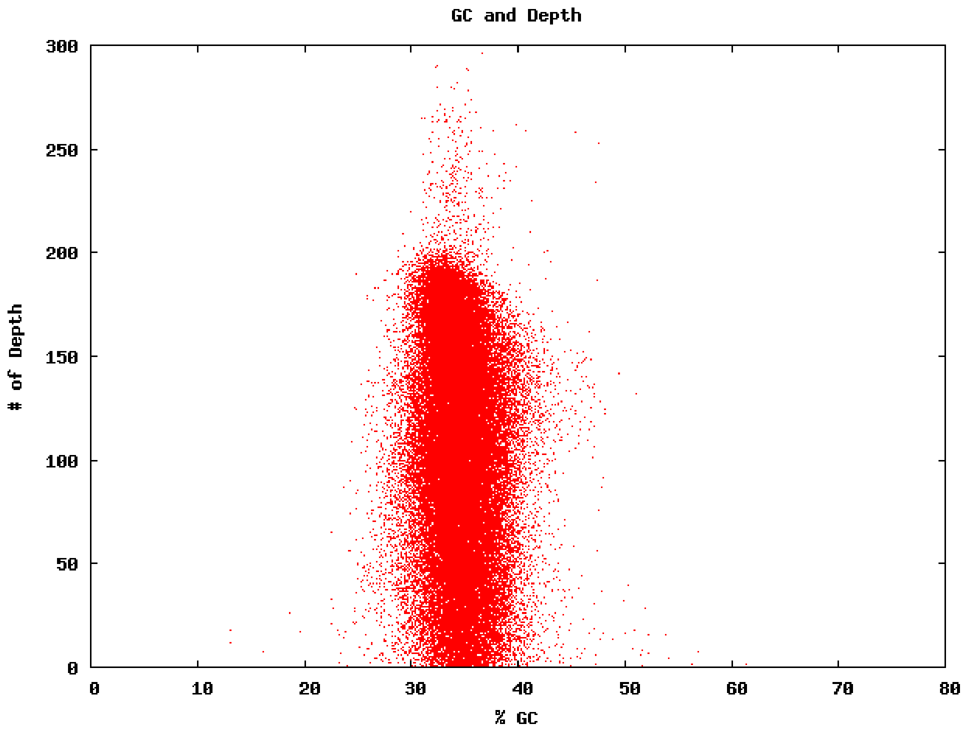

Figure S1-1 The GC content and the distribution of reads depth.

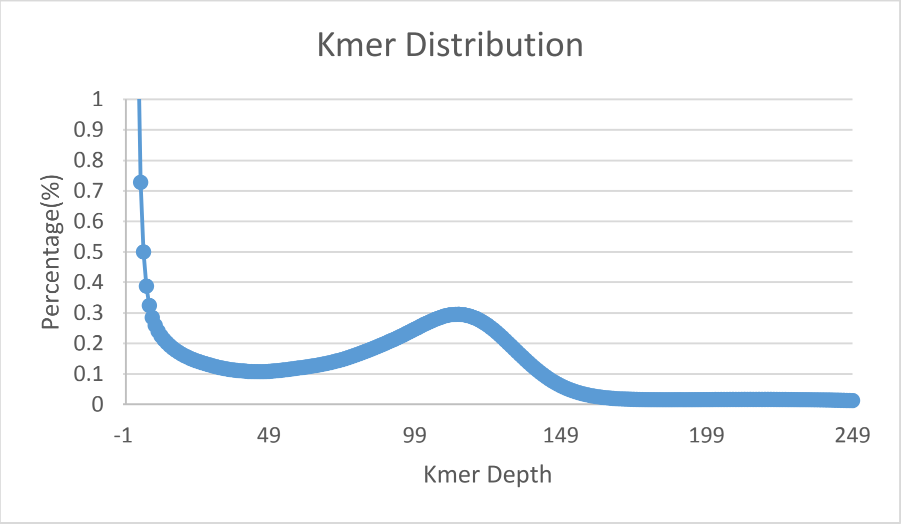

Figure S1-2 The distribution of Kmer depth.

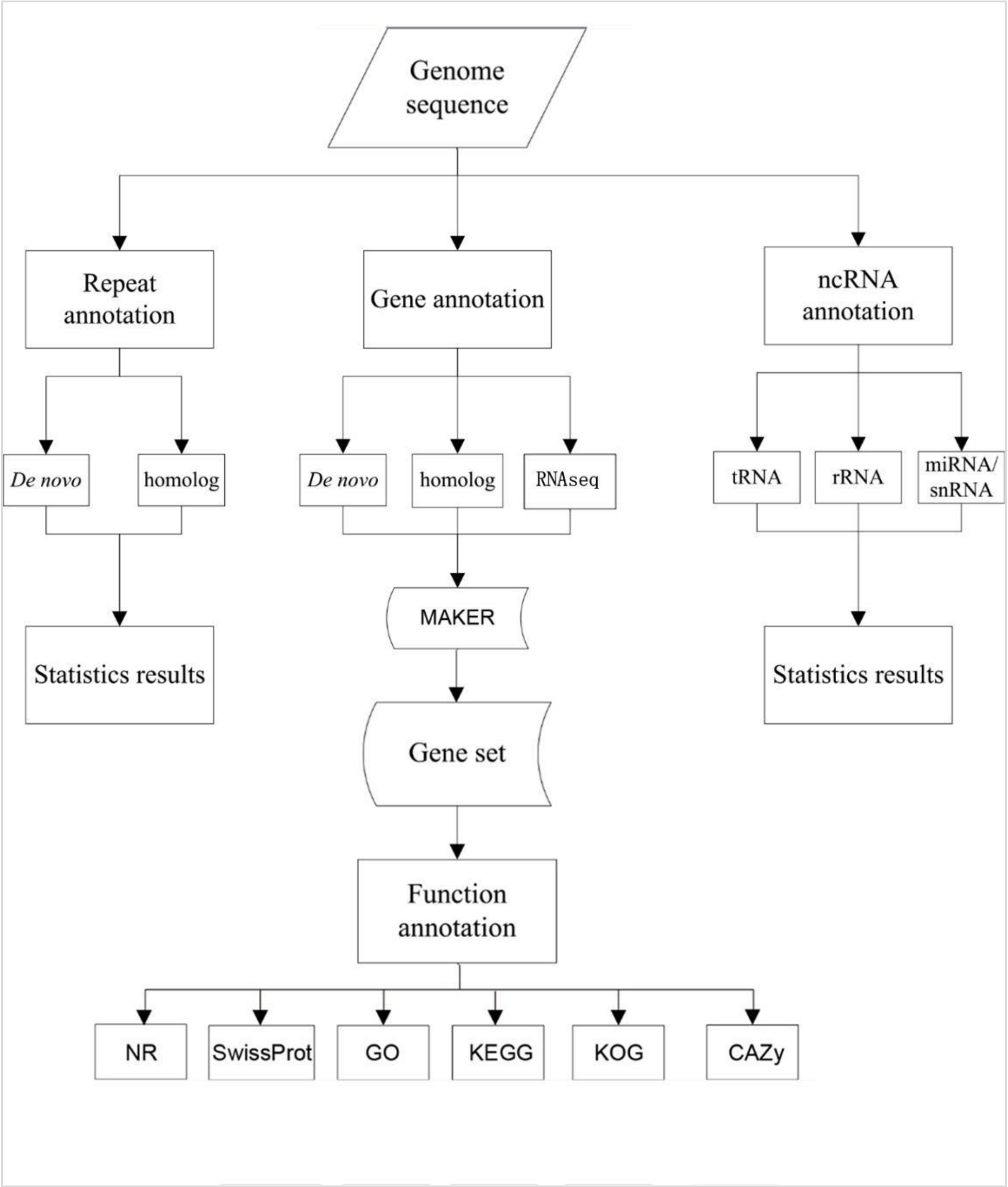

Figure S1-3 The genome annotation flow chart.
