# Supplementary material for: Comparative genomic and transcriptomic analyses of chemosensory genes in the citrus fruit fly Bactrocera (Tetradacus) minax
Source: Sci Rep. 2020 Oct 22;10:18068. doi: 10.1038/s41598-020-74803-5 (PMC7583261; doi:10.1038/s41598-020-74803-5)
Supplement: Supplementary file 3 — Supplementary Information 3. [file 41598_2020_74803_MOESM3_ESM.pdf]

Supplementary file 2

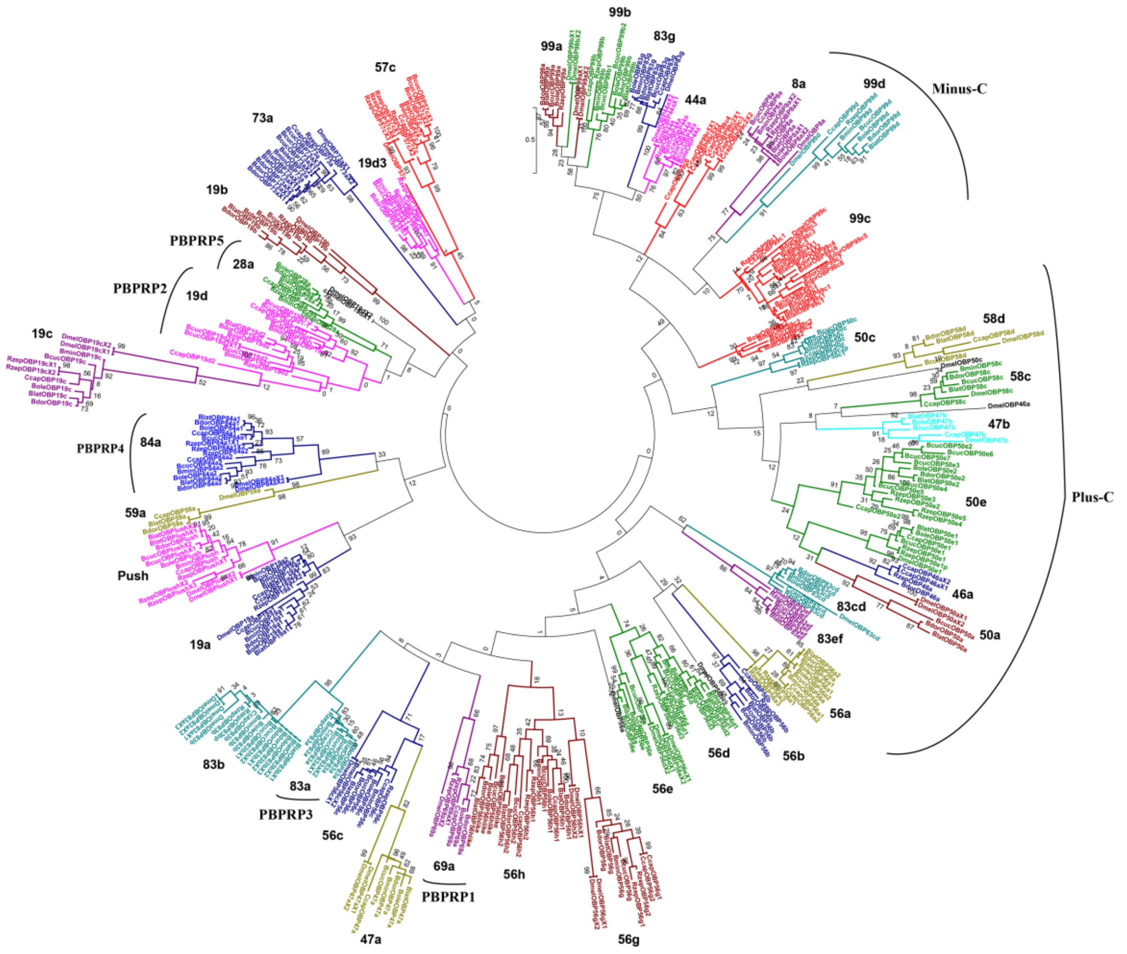

Figure S2-1 Phylogenetic relationships of OBP proteins in the selected Tephritid by the maximum likelihood method. Bootstrap values greater than 50% (1000 replications) were displayed. The numbers of OBP genes present in *D. melanogaster* were previously reported[45].

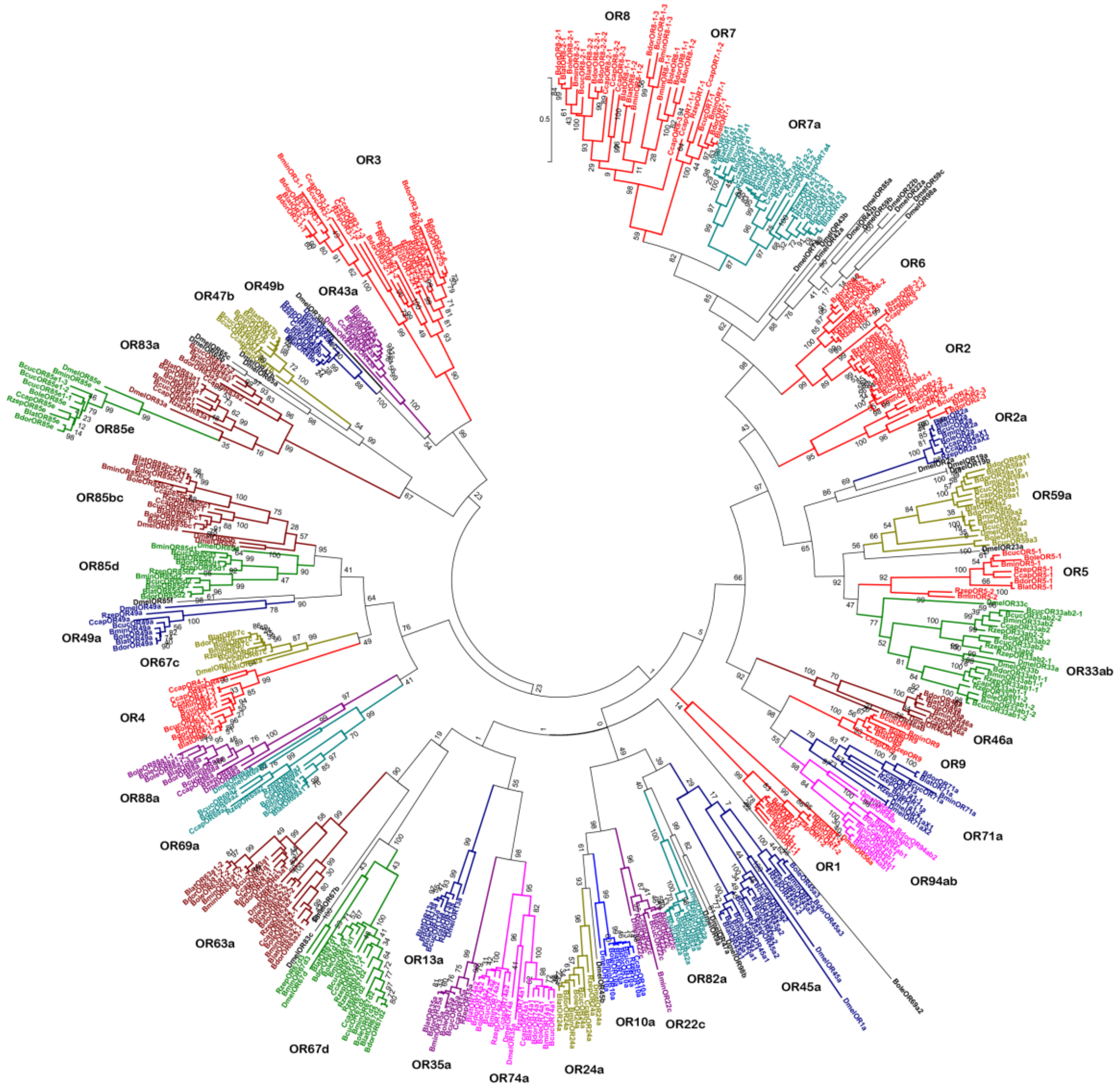

Figure S2-2 **ML tree of the OR family in Tephritidae.** NOTE: The number of OR genes present in *D. melanogaster* has been previously reported[20].
