# Supplementary material for: Comparative genomic and transcriptomic analyses of chemosensory genes in the citrus fruit fly Bactrocera (Tetradacus) minax
Source: Sci Rep. 2020 Oct 22;10:18068. doi: 10.1038/s41598-020-74803-5 (PMC7583261; doi:10.1038/s41598-020-74803-5)
Supplement: Supplementary file 4 — Supplementary Information 4. [file 41598_2020_74803_MOESM4_ESM.pdf]

Supplementary file 3

Table S3-1 The NCBI reference sequences and genbank accessions of OBPs in 7 trypetid species.

<table>
<tr><td>OBP8a</td><td>DmelOBP8a:NP_727322.1;BminOBP8a:Bmi011412;BdorOBP8a:BdorOBP1_hz2013,AKI28997.1,AGS08183.1;BcucOBP8a:gi|751459925|ref|XP_011184701.1|;BlatOBP8a:gi|1098665652|ref|XP_018795287.1|;BoleOBP8aX1:gi|929362373|ref|XP_014090680.1|;BoleOBP8aX2:gi|929362375|ref|XP_014090681.1|;RzepOBP8aX1:gi|1048024647|ref|XP_017472958.1|;RzepOBP8aX2:gi|1048024649|ref|XP_017472959.1|;CcapOBP8a:ref|XP_004521185.1|.</td></tr>
<tr><td>OBP19a</td><td>DmelOBP19a:NP_728338.2;BminOBP19a1:Bmi003171;BdorOBP19a1:AKI28998.1,gi|751779455|ref|XP_011198814.1|;BcucOBP19a1:gi|751464532|ref|XP_011187213.1|;BlatOBP19a1:gi|1098641893|ref|XP_018787238.1|;BoleOBP19a1:gi|929373371|ref|XP_014096691.1|;RzepOBP19a1:gi|1048012126|ref|XP_017466961.1|;RzepOBP19a1p:gi|1048057718|ref|XP_017462172.1|;CcapOBP19a1:ref|XP_004525026.1|;BminOBP19a2:Bmi003172;BdorOBP19a2:gi|1132378836|ref|XP_019844742.1|;BcucOBP19a2:gi|751464530|ref|XP_011187212.1|;BlatOBP19a2:gi|1098641958|ref|XP_018787259.1|;RzepOBP19a2:gi|1048034170|ref|XP_017491138.1|;CcapOBP19a2X1:ref|XP_004525133.2|;CcapOBP19a2X2:ref|XP_020714398.1|.</td></tr>
<tr><td>OBP19b</td><td>DmelOBP19b:NP_608391.2;BminOBP19b:Bmi003173;BdorOBP19b:AKI28999.1,gi|751779453|ref|XP_011198813.1|;BcucOBP19b:gi|751464540|ref|XP_011187218.1|;BlatOBP19b:gi|1098641976|ref|XP_018787266.1|;BoleOBP19b:gi|929373377|ref|XP_014096694.1|;RzepOBP19b:gi|1048028230|ref|XP_017474842.1|;CcapOBP19b:ref|XP_004525027.1|.</td></tr>
<tr><td>OBP19c</td><td>DmelOBP19cX1:NP_608392.1;DmelOBP19cX2:NP_728340.2;BminOBP19c:Bmi003174;BdorOBP19c:AKI29000.1,gi|751779451|ref|XP_011198811.1|;BcucOBP19c:gi|751464542|ref|XP_011187219.1|;BlatOBP19c:gi|1098641970|ref|XP_018787264.1|;BoleOBP19c:gi|929380127|ref|XP_014100435.1|;RzepOBP19cX1:gi|1048028232|ref|XP_017474843.1|;RzepOBP19cX2:gi|1048028234|ref|XP_017474844.1|;CcapOBP19c:ref|XP_004525028.1|.</td></tr>
<tr><td>OBP19d</td><td>DmelOBP19dX1:NP_523421.2;DmelOBP19dX2:NP_788940.1;BminOBP19d1:Bmi003187;BdorOBP19d1:AKI29001.1,gi|751772528|ref|XP_011211361.1|;BcucOBP19d1-1:gi|751464585|ref|XP_011187241.1|;BcucOBP19d1-2:gi|751464591|ref|XP_011187244.1|;BoleOBP19d1:gi|929361375|ref|XP_014090131.1|;RzepOBP19d1:gi|1048015610|ref|XP_017468801.1|;CcapOBP19d1:ref|XP_004525035.1|;BminOBP19d2:Bmi003188;BdorOBP19d2:AKI29002.1,gi|751772526|ref|XP_011211346.1|;BlatOBP19d2:gi|1098641765|ref|XP_018787194.1|;BcucOBP19d2:gi|751464593|ref|XP_011187246.1|;BcucOBP19d2X1:gi|751464587|ref|XP_011187242.1|;BcucOBP19d2X2:gi|751464589|ref|XP_011187243.1|;BoleOBP19d2:gi|929361377|ref|XP_014090132.1|;RzepOBP19d2:gi|1048015608|ref|XP_017468800.1|;CcapOBP19d2:ref|XP_012157066.1|;BminOBP19d3:Bmi003186;BdorOBP19d3:AKI29003.1,gi|751772530|ref|XP_011211373.1|;BlatOBP19d3:gi|1098641966|ref|XP_018787263.1|;BcucOBP19d3X1:gi|751464580|ref|XP_011187239.1|;BcucOBP19d3X2:gi|751464582|ref|XP_011187240.1|;BoleOBP19d3:gi|929361422|ref|XP_014090156.1|;RzepOBP19d3:gi|1048015606|ref|XP_017468799.1|;CcapOBP19d3:ref|XP_004525139.2|.</td></tr>
<tr><td>OBP28a</td><td>DmelOBP28a:NP_523505.1;BminOBP28a:Bmi003166;BdorOBP28a:AKI29004.1,gi|751779467|ref|XP_011198820.1|;BcucOBP28a:gi|751475415|ref|XP_011193147.1|;BlatOBP28a:gi|1098641952|ref|XP_018787257.1|;BoleOBP28a:gi|929370837|ref|XP_014095310.1|;RzepOBP28a:gi|1048015997|ref|XP_017469008.1|;CcapOBP28a:ref|XP_004525016.1|.</td></tr>
<tr><td>OBP44a</td><td>DmelOBP44aX1:NP_610358.1;DmelOBP44aX2:NP_001286186.1;BdorOBP44a:AKI29005.1,gi|751790596|ref|XP_011204856.1|;BcucOBP44a:gi|751456661|ref|XP_011182910.1|;BlatOBP44a:gi|1098651864|ref|XP_018790487.1|;BoleOBP44a:gi|929370029|ref|XP_014094882.1|;RzepOBP44a:gi|1048001217|ref|XP_017469504.1|;CcapOBP44a:ref|XP_004535942.1|.</td></tr>
<tr><td>OBP46a</td><td>DmelOBP46a:NP_610574.1;BdorOBP46a:gi|751799817|ref|XP_011209887.1|;RzepOBP46a:gi|1048001507|ref|XP_017471013.1|;CcapOBP46aX1:ref|XP_012155810.1|;CcapOBP46aX2:ref|XP_012155811.1|.</td></tr>
<tr><td>OBP47a</td><td>DmelOBP47aX1:NP_610632.1;DmelOBP47aX2:NP_995810.1;BminOBP47a:Bmi004519;BdorOBP47a:gi|751779757|ref|XP_011198976.1|;BcucOBP47a:gi|751464715|ref|XP_011187314.1|;BlatOBP47a:gi|1098626193|ref|XP_018805032.1|;BoleOBP47a:gi|929356598|ref|XP_014087515.1|;CcapOBP47a:ref|XP_012154866.1|.</td></tr>
<tr><td>OBP47b</td><td>DmelOBP47b:NP_610669.1;BcucOBP47b:gi|751447460|ref|XP_011177869.1|;BlatOBP47b:gi|1098622130|ref|XP_018803955.1|;BoleOBP47b:gi|929351763|ref|XP_014103556.1|;CcapOBP47b:ref|XP_012162233.1|.</td></tr>
<tr><td>OBP50a</td><td>DmelOBP50aX1:NP_725385.1;DmelOBP50aX2:NP_995832.1;BdorOBP50a:gi|751799975|ref|XP_011209971.1|;BcucOBP50a:gi|751444190|ref|XP_011196826.1|;BlatOBP50a:gi|1098675124|ref|XP_018797907.1|.</td></tr>
<tr><td>OBP50c</td><td>DmelOBP50c:NP_725387.3;BminOBP50c:Bmi005981;BdorOBP50c:AKI29006.1,gi|1132380618|ref|XP_011200390.2|;BcucOBP50c:gi|751440376|ref|XP_011189341.1|;BlatOBP50c:gi|1098664619|ref|XP_018794965.1|;RzepOBP50c1:gi|1048070291|ref|XP_017464462.1|;RzepOBP50c2p:gi|1048058810|ref|XP_017482802.1|;CcapOBP50c:ref|XP_004522983.1|.</td></tr>
<tr><td>OBP50e</td><td>DmelOBP50e1:NP_610959.2;BcucOBP50e1:gi|751443449|ref|XP_011196419.1|;BlatOBP50e1:gi|1098675110|ref|XP_018797902.1|;BoleOBP50e1:gi|929345206|ref|XP_014096496.1|;RzepOBP50e1:gi|1048028013|ref|XP_017474729.1|;RzepOBP50e1p:gi|1048044947|ref|XP_017493931.1|;CcapOBP50e1:ref|XP_012157857.1|;BdorOBP50e2:AKI29007.1;BlatOBP50e2:gi|1098645661|ref|XP_018788552.1|;BoleOBP50e2:gi|929371053|ref|XP_014095428.1|;BcucOBP50e2:gi|751469797|ref|XP_011190101.1|;BcucOBP50e3:gi|751469739|ref|XP_011190069.1|;BcucOBP50e4:gi|751469801|ref|XP_011190103.1|;BcucOBP50e5:gi|751469737|ref|XP_011190068.1|;BcucOBP50e6:gi|751479390|ref|XP_011195271.1|;BcucOBP50e7:gi|751469741|ref|XP_011190070.1|;RzepOBP50e2:gi|1048059581|ref|XP_017483225.1|;RzepOBP50e3:gi|1048027767|ref|XP_017474599.1|;RzepOBP50e4:gi|1048052290|ref|XP_017460912.1|;RzepOBP50e5:gi|1048027775|ref|XP_017474604.1|;CcapOBP50e2:ref|XP_004533835.1|.</td></tr>
<tr><td>OBP56a</td><td>DmelOBP56a:NP_611442.1;BminOBP56a:Bmi006859;BdorOBP56a:AKI29008.1,gi|1132377112|ref|XP_019844410.1|;BcucOBP56a:gi|751452896|ref|XP_011180838.1|;BlatOBP56a1:gi|1098633108|ref|XP_018784475.1|;BlatOBP56a2:gi|1098633116|ref|XP_018784478.1|;BoleOBP56a1:gi|929349145|ref|XP_014102105.1|;BoleOBP56a2:gi|929349151|ref|XP_014102109.1|;RzepOBP56a1:gi|1048049278|ref|XP_017479697.1|;RzepOBP56a2:gi|1048049280|ref|XP_017479700.1|;RzepOBP56a3:gi|1048049282|ref|XP_017479701.1|;CcapOBP56a1:ref|XP_020718148.1|;CcapOBP56a2:ref|XP_020718149.1|.</td></tr>
</table>

OBP56b	DmelOBP56b:NP_611443.1;BminOBP56b:Bmi006857;BdorOBP56b:gi|751776657|ref|XP_011197298.1|;BcucOBP56b:gi|751453034|ref|XP_011180914.1|;BlatOBP56b:gi|1098633099|ref|XP_018784471.1|;BoleOBP56b:gi|929349117|ref|XP_014102090.1|;RzepOBP56b:gi|1048068744|ref|XP_017486529.1|;CcapOBP56b:ref|XP_004517903.1|.
OBP56c:	DmelOBP56cX1:NP_995902.1;DmelOBP56cX2:NP_725925.3;BminOBP56c:Bmi006858;BdorOBP56c:gi|1132377106|ref|XP_011197306.2|;:BcucOBP56c:gi|751452892|ref|XP_011180836.1|;BlatOBP56c:gi|1098633094|ref|XP_018784469.1|;BoleOBP56c:gi|929349115|ref|XP_014102088.1|;RzepOBP56c:gi|1048068738|ref|XP_017486526.1|;CcapOBP56c:ref|XP_012155938.2|.
OBP56d	DmelOBP56dX1:NP_611444.2;DmelOBP56dX2:NP_001286619.1;BminOBP56d:Bmi006856;BdorOBP56d1:BdorOBP4_hz2013,AGS08186.1,gi|751776663|ref|XP_011197302.1|;BdorOBP56d2:BdorOBP2_hz2013,AKI29010.1,AGS08184.1,gi|751776661|ref|XP_011197300.1|;BdorOBP56d3:AKI29009.1,gi|751776659|ref|XP_011197299.1|;BcucOBP56d:gi|751452890|ref|XP_011180835.1|;BlatOBP56d1:gi|1098633078|ref|XP_018784464.1|;BlatOBP56d2:gi|1098633084|ref|XP_018784466.1|;BoleOBP56d:gi|929349029|ref|XP_014102039.1|;RzepOBP56d1:gi|1048068740|ref|XP_017486527.1|;RzepOBP56d2:gi|1048068742|ref|XP_017486528.1|;RzepOBP56d3:gi|1048044784|ref|XP_017493842.1|;CcapOBP56d:ref|XP_004517803.1|.
OBP56e	DmelOBP56eX1:NP_611445.1;DmelOBP56eX2:NP_001286620.1;BdorOBP56e:AKI29011.1,gi|751776655|ref|XP_011197297.1|;BcucOBP56e:gi|751452894|ref|XP_011180837.1|;BlatOBP56e:gi|1098633096|ref|XP_018784470.1|;BoleOBP56e:gi|929348975|ref|XP_014102008.1|;CcapOBP56e:ref|XP_004517904.1|.
OBP56g	DmelOBP56gX1:NP_611447.1;DmelOBP56gX2:NP_995903.1;BminOBP56g:Bmi006852;BdorOBP56g:BdorOBP5_hz2013,:AGS08187.1,gi|751776669|ref|XP_011197305.1|;BcucOBP56g:gi|751452880|ref|XP_011180830.1|;BlatOBP56g:gi|1098663783|ref|XP_018794675.1|;RzepOBP56g1:gi|1048051220|ref|XP_017480715.1|;RzepOBP56g2:gi|1048066219|ref|XP_017485615.1|;CcapOBP56g1:ref|XP_004518466.1|;CcapOBP56g2:ref|XP_012155720.1|.
OBP56h	DmelOBP56hX1:NP_611448.2;DmelOBP56hX2:NP_001188979.1;BminOBP56h1:Bmi006853;BdorOBP56h1:BdorOBP6_hz2013,AKI29012.1,gi|751776667|ref|XP_011197304.1|;BcucOBP56h1:gi|751452882|ref|XP_011180831.1|;BlatOBP56h1:gi|1098633113|ref|XP_018784477.1|;BoleOBP56h1:gi|929349131|ref|XP_014102098.1|;RzepOBP56h1:gi|1048067246|ref|XP_017486157.1|;CcapOBP56h1:ref|XP_004517804.1|;BdorOBP56h2:gi|751798262|ref|XP_011209038.1|;BcucOBP56h2:gi|751453028|ref|XP_011180911.1|;BlatOBP56h2:gi|1098663737|ref|XP_018794661.1|;RzepOBP56h2:gi|1048067244|ref|XP_017486156.1|;CcapOBP56h2:ref|XP_004517905.1|;BminOBP56hlike:Bmi008922;BdorOBP56hlike::gi|751798264|ref|XP_011209039.1|;BlatOBP56hlike:gi|1098663741|ref|XP_018794662.1|;BcucOBP56hlike:gi|751452878|ref|XP_011180828.1|;BoleOBP56hlike:gi|929348971|ref|XP_014102006.1|.
OBP57c	DmelOBP57c:NP_611481.1;BdorOBP57c:AKI29013.1,gi|1132378253|ref|XP_011198265.2|;BcucOBP57cX1:gi|751461171|ref|XP_011185379.1|;BcucOBP57cX2:gi|751461169|ref|XP_011185378.1|;BlatOBP57c:gi|1098634981|ref|XP_018785093.1|;BoleOBP57c:gi|929363540|ref|XP_014091324.1|;RzepOBP57cX1:gi|1048032745|ref|XP_017490358.1|;RzepOBP57cX2:gi|1048032751|ref|XP_017490361.1|;RzepOBP57cX3:gi|1048032747|ref|XP_017490359.1|;RzepOBP57cX4:gi|1048032749|ref|XP_017490360.1|;CcapOBP57c:ref|XP_004522856.1|.
OBP58c	DmelOBP58c:NP_611710.1;BminOBP58c:Bmi002479;BdorOBP58c:gi|751797852|ref|XP_011208814.1|;BcucOBP58c:gi|751447464|ref|XP_011177871.1|;BlatOBP58c:gi|1098622243|ref|XP_018803987.1|;CcapOBP58c:ref|XP_004537654.1|.:
OBP58d	DmelOBP58d:NP_611711.1;BdorOBP58d:gi|751798003|ref|XP_011208895.1|;BcucOBP58d:gi|751447836|ref|XP_011178073.1|;BlatOBP58d:gi|1098622002|ref|XP_018803917.1|;CcapOBP58d:ref|XP_004537653.1|.
OBP59a	DmelOBP59a:NP_788429.1;BdorOBP59a:gi|1132390486|ref|XP_011208813.2|;BlatOBP59a:gi|1098622140|ref|XP_018803957.1|;CcapOBP59a:ref|XP_020717722.1|.
OBP69a	DmelOBP69a:NP_524039.2;BdorOBP69a:AKI29014.1,gi|751784296|ref|XP_011201434.1|;BoleOBP69a:gi|929359012|ref|XP_014088831.1|;RzepOBP69aX1:gi|1048040848|ref|XP_017477316.1|;RzepOBP69aX2:gi|1048040850|ref|XP_017477317.1|;CcapOBP69a:ref|NP_001295335.1|.
OBP73a	DmelOBP73aX1:NP_001097628.1;DmelOBP73aX2:NP_001334711.1;BminOBP73a:Bmi005271;BdorOBP73aX1::gi|751805444|ref|XP_011212952.1|;BdorOBP73aX2:gi|1132395267|ref|XP_019848181.1|;BdorOBP73aX3::gi|1132395269|ref|XP_019848182.1|;BcucOBP73aX1:gi|751458328|ref|XP_011183827.1|;BcucOBP73aX2:gi|751458330|ref|XP_011183828.1|;BcucOBP73aX3:gi|751458332|ref|XP_011183829.1|;BlatOBP73aX1:gi|1098660105|ref|XP_018793310.1|;BlatOBP73aX2:gi|1098660110|ref|XP_018793311.1|;BoleOBP73a:gi|929366884|ref|XP_014093157.1|;RzepOBP73a:gi|1048028435|ref|XP_017474953.1|;RzepOBP73ap:gi|1048052313|ref|XP_017460926.1|;CcapOBP73a:ref|XP_012156332.1|.
OBP83a	DmelOBP83aX1:NP_524241.1;DmelOBP83aX2:NP_001287189.1;DmelOBP83aX3:NP_001287190.1;BdorOBP83a:AKI29016.1,ACB56577.1,AGO28153.1,AGS42237.1,gi|751804561|ref|XP_011212472.1|;BcucOBP83aX1:gi|751440806|ref|XP_011191671.1|;BcucOBP83aX2:gi|751440804|ref|XP_011191662.1|;BminOBP83a:Bmi002228;BlatOBP83a:gi|1098617602|ref|XP_018794248.1|;BoleOBP83a:gi|929362148|ref|XP_014090557.1|;RzepOBP83a:gi|1048044723|ref|XP_017493809.1|;CcapOBP83a:ref|NP_001295333.1|.
OBP83b	DmelOBP83b:NP_524242.2;BdorOBP83bX1:AKI29015.1,gi|751804559|ref|XP_011212470.1|;BdorOBP83bX2::gi|751804557|ref|XP_011212469.1|;BdorOBP83bX3:gi|1132395332|ref|XP_019848198.1|;BminOBP83b:Bmi002229;BcucOBP83bX1:gi|751440802|ref|XP_011191650.1|;BcucOBP83bX2:gi|751440800|ref|XP_011191644.1|;BlatOBP83bX1:gi|1098617535|ref|XP_018794048.1|;BlatOBP83bX2:gi|1098617539|ref|XP_018794058.1|;BoleOBP83b:gi|929362150|ref|XP_014090558.1|;RzepOBP83b:gi|1048019882|ref|XP_017488099.1|;RzepOBP83bp:gi|1048045461|ref|XP_017494211.1|;CcapOBP83b:ref|XP_020713726.1|.
OBP83cd	DmelOBP83cd:NP_649612.1;BdorOBP83cd:AKI29017.1;BminOBP83cd:Bmi002221;BlatOBP83cd:gi|1098618149|ref|XP_018795816.1|;BoleOBP83cd:gi|929362154|ref|XP_014090560.1|;RzepOBP83cd:gi|1048044711|ref|XP_017493802.1|;CcapOBP83cd:ref|XP_020713668.1|.
OBP83ef	DmelOBP83ef:NP_731042.1;BdorOBP83ef:BdorOBP8_hz2013,AKI29018.1,AGS08190.1,gi|1132394775|ref|XP_019848076.1|;BlatOBP83ef:gi|1098618146|ref|XP_018795806.1|;BcucOBP83ef:gi|751441754|ref|XP_011195484.1|;BoleOBP83ef:gi|929362144|ref|XP_014090554.1|;RzepOBP83ef:gi|1048061121|ref|XP_017462537.1|.
OBP83g	DmelOBP83g:NP_731043.1;BminOBP83g:Bmi002220;BdorOBP83g:BdorOBP7_hz2013,AKI29019.1,AGS08189.1,gi|751804578|ref|XP_011212480.1|;BcucOBP83g:gi|751440786|ref|XP_011191566.1|;BlatOBP83g:gi|1098618152|ref|XP_018795827.1|;BoleOBP83g:gi|929362146|ref|XP_014090556.1|;CcapOBP83g:ref|XP_004523508.1|.

OBP84a	DmelOBP84aX1:NP_476990.1;DmelOBP84aX2:NP_001097700.2;BdorOBP84a1:AKI29020.1,gi|751793934|ref|XP_011206667.1|;BcucOBP84a1:gi|751460114|ref|XP_011184805.1|:;BlatOBP84a1:gi|1098615499|ref|XP_018787917.1|;BoleOBP84a1:gi|929348031|ref|XP_014097710.1|;RzepOBP84a1X1:gi|1048069032|ref|XP_017486683.1|;RzepOBP84a1X2:gi|1048069034|ref|XP_017486684.1|;CcapOBP84a1:ref|XP_012158643.1|.BminOBP84a2:Bmi011107;BdorOBP84a2:AKI29021.1,AKM45827.1,gi|751778725|ref|XP_011198416.1|;BcucOBP84a2:gi|751460100|ref|XP_011184797.1|;BlatOBP84a2:gi|1098685020|ref|XP_018801147.1|;BoleOBP84a2:gi|929348033|ref|XP_014097722.1|;RzepOBP84a2:gi|1048050452|ref|XP_017480316.1|;CcapOBP84a2:ref|XP_004529369.1|.
OBP99a	DmelOBP99aX1:NP_651707.1;DmelOBP99aX2:NP_001287586.1;BdorOBP99a:BdorOBP9_hz2013,AGS08191.1,gi|751791234|ref|XP_011205204.1|;BcucOBP99a:gi|751447118|ref|XP_011177680.1|;BlatOBP99a:gi|1098653297|ref|XP_018790942.1|;BoleOBP99a:gi|929367138|ref|XP_014093299.1|;RzepOBP99a:gi|1048023721|ref|XP_017472452.1|.
OBP99b	DmelOBP99bX1:NP_651713.1;DmelOBP99bX2:NP_001263078.1;BminOBP99b:Bmi011411;BdorOBP99b:AKI29022.1,gi|751800809|ref|XP_011210417.1|;BcucOBP99b1:gi|751459929|ref|XP_011184703.1|;BcucOBP99b2:gi|751459927|ref|XP_011184702.1|;BlatOBP99b:gi|1098665662|ref|XP_018795290.1|;BoleOBP99b:gi|929362389|ref|XP_014090689.1|;RzepOBP99b:gi|1048024645|ref|XP_017472956.1|;CcapOBP99b:ref|XP_004521183.1|.
OBP99c	DmelOBP99c:NP_651711.1;BminOBP99c1:Bmi011415;BdorOBP99c1:BdorOBP10_hz2013,AKI29023.1,AGC82131.1,AGS08192.1,gi|751800815|ref|XP_011210420.1|;BcucOBP99c1:gi|751459919|ref|XP_011184698.1|;BlatOBP99c1:gi|1098678699|ref|XP_018799091.1|;BoleOBP99c1:gi|929362371|ref|XP_014090679.1|;RzepOBP99c1:gi|1048007580|ref|XP_017464529.1|;CcapOBP99c1:ref|XP_004521186.1|;BminOBP99c2:Bmi011416;BdorOBP99c2:AKI29026.1,AGC82130.1,gi|751800827|ref|XP_011210427.1|;BcucOBP99c2:gi|751459917|ref|XP_011184697.1|;BlatOBP99c2:gi|1098678712|ref|XP_018799095.1|;BoleOBP99c2:gi|929362343|ref|XP_014090663.1|;BdorOBP99c3:AKI29024.1,gi|751800825|ref|XP_011210426.1|;BlatOBP99c3:gi|1098678708|ref|XP_018799094.1|;BoleOBP99c3:gi|929362393|ref|XP_014090691.1|;BdorOBP99c4:gi|751800823|ref|XP_011210425.1|;BlatOBP99c4X1:gi|1098678702|ref|XP_018799092.1|;BlatOBP99c4X2:gi|1098678705|ref|XP_018799093.1|;BdorOBP99c5:Bdor64_gi|751800819|ref|XP_011210422.1|;BdorOBP99c6:AKI29025.1,AGC82132.1,gi|751800817|ref|XP_011210421.1|;BdorOBP99c7:Bdor64_gi|751800859|ref|XP_011210445.1|;BdorOBP99c8:Bdor64_gi|751800821|ref|XP_011210423.1|;CcapOBP99c2X1:ref|XP_004536904.1|;CcapOBP99c2X2:ref|XP_004536905.1|;CcapOBP99c3:ref|NP_001295338.1|;CcapOBP99c4:ref|NP_001266316.1|;CcapOBP99c5X1:ref|XP_004536902.1|;CcapOBP99c5X2:ref|XP_020717485.1|;CcapOBP99c6:ref|XP_020717484.1|.
OBP99d	DmelOBP99d:NP_651712.1;BminOBP99d:Bmi011413;BdorOBP99d:gi|1132392371|ref|XP_019847572.1|;BcucOBP99d:gi|751459923|ref|XP_011184700.1|;BlatOBP99d:gi|1098665649|ref|XP_018795286.1|;BoleOBP99d:gi|929362377|ref|XP_014090682.1|;RzepOBP99d:gi|1048024651|ref|XP_017472960.1|;CcapOBP99d:ref|XP_004521184.1|.
OBPlush	DmelOBPlushX1:NP_524162.1;DmelOBPlushX2:NP_001163468.1;BminOBPlush:Bmi007112;BdorOBPlush:AKI28996.1,gi|751796449|ref|XP_011208040.1|;BcucOBPlushX1:gi|751438043|ref|XP_011176687.1|;BcucOBPlushX2:gi|751438045|ref|XP_011176696.1|;BlatOBPlushX1:gi|1098650774|ref|XP_018790185.1|;BlatOBPlushX2:gi|1098650769|ref|XP_018790184.1|;BoleOBPlush:gi|929359810|ref|XP_014089270.1|;RzepOBPlushX1:gi|1048040981|ref|XP_017477379.1|;RzepOBPlushX2:gi|1048040983|ref|XP_017477380.1|;RzepOBPlushX3:gi|1048040987|ref|XP_017477382.1|;CcapOBPlush:ref|XP_004522281.1|.

Table S3-2 The NCBI reference sequences and genbank accessions of CSPs in 7 trypetid species.

CSP1	DmelCSP1:FBgn0035089,NP611990;BminCSP1:Bmi004355;BdorCSP1X1:AKI28975.1,gi|751771303|ref|XP011204433.1|;BdorCSP1X2:AKI28975.1,gi|751771305|ref|XP011204439.1|;BcucCSP1:gi|751479098|ref|XP011195115.1|;BlatCSP1X1:gi|1098660662|ref|XP018793511.1|;BlatCSP1X2:gi|1098660665|ref|XP018793513.1|;BlatCSP1X3:gi|1098660668|ref|XP018793514.1|;BoleCSP1X1:gi|929374974|ref|XP014097577.1|;BoleCSP1X2:gi|929374976|ref|XP014097578.1|;BoleCSP1X3:gi|929374978|ref|XP014097579.1|;RzepCSP1X1:gi|1048023278|ref|XP017472213.1|;RzepCSP1X2:gi|1048023280|ref|XP017472214.1|;RzepCSP1X3:gi|1048023282|ref|XP017472216.1|;CcapCSP1:ref|XP012158387.1|;
CSP2	DmelCSP2:FBgn0011695,NP524966;BminCSP2:Bmi004346;BdorCSP2:AKI28976.1,gi|751770936|ref|XP011202324.1|;BcucCSP2:gi|751477596|ref|XP011194329.1|;BlatCSP2:gi|1098643949|ref|XP018787923.1|;BoleCSP2:gi|929375975|ref|XP014098127.1|;RzepCSP2X1:gi|1048058965|ref|XP017482883.1|;RzepCSP2X2:gi|1048058967|ref|XP017482884.1|;CcapCSP2X1:ref|XP004536818.1|;CcapCSP2X2:ref|XP004536819.1|.
CSP3	DmelCSP3:FBgn0011293,AAF49381;BminCSP3X1:Bmi010858.1;BminCSP3X2:Bmi010858.2;BdorCSP3X1:AKI28977.1,AGS42236.1,gi|751797401|ref|XP011208559.1|;BdorCSP3X2:ACB56576.1,gi|751797399|ref|XP011208558.1|;BcucCSP3X1:gi|751446282|ref|XP011177223.1|;BcucCSP3X2:gi|751446280|ref|XP011177222.1|;BlatCSP3X1:gi|1098634183|ref|XP018784825.1|;BlatCSP3X2:gi|1098634180|ref|XP018784824.1|;BoleCSP3X1:gi|929355737|ref|XP014087038.1|;BoleCSP3X2:gi|929355735|ref|XP014087037.1|;RzepCSP3X1:gi|1048059474|ref|XP017483166.1|;RzepCSP3X2:gi|1048059476|ref|XP017483167.1|;CcapCSP3X1:ref|XP004529710.1|;CcapCSP3X2:ref|XP004529711.1|.
CSP4	DmelCSP4:FBgn0050172,NP726402;BminCSP4:Bmi004554;BdorCSP4:AKI28978.1,gi|1132373093|ref|XP019845325.1|;BlatCSP4:gi|1098669275|ref|XP018796406.1|;BoleCSP4:gi|929374364|ref|XP014097241.1|;RzepCSP4:gi|1048026429|ref|XP017473899.1|;CcapCSP4:ref|XP004536817.1|.

Table S3-3 The NCBI reference sequences and genbank accessions of ORs in 7 trypetid species.

<table>
<tr><td>OR2a</td><td>DmelOR2a:NP_525046.1;BminOR2a:Bmi006479;BdorOR2a:gi|751778677|ref|XP011198390.1|,XP011198390.1,XM_011200088.1;BcucOR2a:gi|751465288|ref|XP_01118</td></tr>
<tr><td>OR7a</td><td>DmelOR7a:NP_511081.1;BminOR7a1:Bmi005185;BdorOR7a1:AKI29028.1,gi|1132390315|ref|XP019847175.1|;BcucOR7a1:gi|751454947|ref|XP_011181972.1|;BlatOR7a
R7a2:gi|1098677130|ref|XP_018798558.1|;BoleOR7a2:gi|929364851|ref|XP_014092042.1|;RzepOR7a2-1:gi|1048068258|ref|XP_017486266.1|;RzepOR7a2-2:gi|104802738
1:gi|1048063022|ref|XP_017484414.1|;RzepOR7a3-2:gi|1048068821|ref|XP_017486569.1|;CcapOR7a3-1:ref|XP_004520908.2|;CcapOR7a3-2:ref|XP_012161119.1|;RzepOR</td></tr>
<tr><td>OR10a</td><td>DmelOR10a:NP_511122.1;BminOR10a:Bmi009555;BdorOR10a:XP011206229.1,XM_011207927.1,gi|751793114|ref|XP011206229.1|;BcucOR10a:gi|751459863|ref|XP_01</td></tr>
<tr><td>OR13a</td><td>DmelOR13a:NP_523359.2;BdorOR13a:AKI29033.1,XP011204429.1,XM_011206127.2,gi|751789805|ref|XP011204429.1|;BcucOR13a:gi|751446550|ref|XP_011177369.1|;</td></tr>
<tr><td>OR22c</td><td>DmelOR22c:NP_523454.2;BminOR22c:Bmi009742;BdorOR22c:gi|751800230|ref|XP011210110.1|;BcucOR22c:gi|751476046|ref|XP_011193492.1|;BlatOR22c:gi|109864</td></tr>
<tr><td>OR24a</td><td>DmelOR24a:NP_523470.3;BminOR24a:Bmi010522;BdorOR24a:XP011199522.1,XM_011201220.1,gi|751780772|ref|XP011199522.1|;BcucOR24a:gi|751470546|ref|XP_01</td></tr>
<tr><td>OR33ab</td><td>DmelOR33a:NP_523553.1;DmelOR33b:NP_523554.1;DmelOR33c:NP_523555.1;BminOR33ab1-1:Bmi000272;BdorOR33ab1-1:gi|751796266:XP_011207940.1|;BoleOR3
2:Bmi000270;BcucOR33ab2:gi|751448977|ref|XP_011178698.1|;BoleOR33ab2:gi|929374155|ref|XP_014097126.1|;BminOR33ab2:Bmi000271;BcucOR33ab1-2:gi|751448</td></tr>
<tr><td>OR35a</td><td>DmelOR35a:NP_723916.1;BminOR35a1:Bmi002525;BdorOR35a:gi|1132377223|ref|XP_019844437.1|;BcucOR35a:gi|751461147|ref|XP_011185366.1|;BoleOR35a:gi|9293</td></tr>
<tr><td>OR43a</td><td>DmelOR43a:NP_523647.2;BminOR43a:Bmi005218;BdorOR43a:AKI29035.1,XP_011214112.1,XM_011215810.1,gi|751774946|ref|XP011214112.1|;BcucOR43a:gi|75144</td></tr>
<tr><td>OR45a</td><td>DmelOR45a:NP_523666.3;BminOR45a1:Bmi003431;BdorOR45a1:AKI29038.1,XP011212447.2,XM_011214145.2,gi|1132394498|ref|XP011212447.2|;BcucOR45a1:gi|75
XP_014098071.1|;CcapOR45a2:ref|XP_004534466.1|;RzepOR45a2:gi|1048052069|ref|XP_017460790.1|;BminOR45a3:Bmi003430;BdorOR45a3:XP011212445.1,XM_011</td></tr>
<tr><td>OR46a</td><td>DmelOR46aB:NP_995793.1;DmelOR46aA:995794.1;BminOR46a:Bmi004385;BdorOR46a:XP011200513.1,XM_011202211.1,gi|751770615|ref|XP011200513.1|;BlatOR4</td></tr>
<tr><td>OR47b</td><td>DmelOR47b:NP_523690.3;BminOR47b:Bmi004946;BdorOR47b:XP019847427.1,XM_019991868.1,gi|1132391553|ref|XP019847427.1|;BcucOR47bX1:gi|751443942|ref|</td></tr>
<tr><td>OR49a</td><td>DmelOR49a:NP_523711.3;BminOR49a:Bmi002639;BdorOR49a:XP011212431.1,XM_011214129.1,gi|751804491|ref|XP011212431.1|;BcucOR49a:gi|751453665|ref|XP_01</td></tr>
<tr><td>OR49b</td><td>DmelOR49b:NP_523721.1;BdorOR49b:AKI29039.1,XP019845516.1,XM_019989957.1,gi|1132382716|ref|XP019845516.1|;BcucOR49b:gi|751443238|ref|XP_011196302.1</td></tr>
<tr><td>OR59a</td><td>DmelOR59a:NP_523821.1;BminOR59a1:Bmi009042;BdorOR59a1:AKI29041.1;BdorOR59a1p:XP019844588.1,XM_019989029.1,gi|1132372707|ref|XP019844588.1|;Bla
ucOR59a2:gi|751444182|ref|XP_011196822.1|;BcucOR59a1:gi|751467191|ref|XP_011188666.1|;BoleOR59a3:gi|929344964|ref|XP_014096236.1|;BcucOR59a3:gi|7514441</td></tr>
<tr><td>OR63a</td><td>DmelOR63a:NP_523895.2;BminOR63a1-1:Bmi004869;BminOR63a1-2:Bmi004870;BminOR63a2:Bmi004730;BdorOR63a1-1:XP011201816.1,XM_011203514.1,gi|7517
2:gi|929368811|ref|XP_014094223.1|;BdorOR63a2-1:AKI29042.1;BlatOR63a2-1:gi|1098643899|ref|XP_018787905.1|;BoleOR63a2-1:gi|929376191|ref|XP_014098250.1|;</td></tr>
<tr><td>OR67c</td><td>DmelOR67c:NP_524018.2;BminOR67c:Bmi008170;BdorOR67c:XP011199152.2,XM_011200850.2,gi|1132379161|ref|XP_011199152.2|;BcucOR67c:gi|751451442|ref|XP_</td></tr>
<tr><td>OR67d</td><td>DmelOR67d:NP_648390.2;BminOR67d1:Bmi000893;BdorOR67d1:AKI29045.1,XP011203703.1,XM_011205401.1,gi|751788481|ref|XP011203703.1|;BcucOR67d1:gi|751
2:gi|1048015337|ref|XP_017468655.1|;CcapOR67d1:ref|XP_004533437.2|;BminOR67d2:Bmi000894;BdorOR67d2:XP011203704.2,:XM_011205402.2,gi|1132384382|ref|</td></tr>
<tr><td>OR69a</td><td>DmelOR69aB:NP_996069.1;DmelOR69aA:996070.1;BdorOR69a1:AKI29046.1,XP011209369.1,XM_011211067.2,gi|751772204|ref|XP_011209369.1|;BcucOR69a1:gi|751
13.1|;BoleOR69a2:gi|929358429|ref|XP_014088513.1|;RzepOR69a2:gi|1048009089|ref|XP_017465320.1|;CcapOR69a2:ref|XP_020715762.1|.</td></tr>
<tr><td>OR71a</td><td>DmelOR71aX1:NP_524078.2;DmelOR71aX2:NP_001246763.1;BminOR71a:Bmi004512;BdorOR71a:XP011205499.1,XM_011207197.2,gi|751791772|ref|XP011205499.1</td></tr>
<tr><td>OR74a</td><td>DmelOR74a:NP_524123.1;BminOR74a1:Bmi008540;BdorOR74a1:XP011200119.1,XM_011201817.1,gi|751781873|ref|XP_011200119.1|;BcucOR74a1:gi|751457865|ref|
1379.1;BoleOR74a2:gi|929353423|ref|XP_014085775.1|;BcucOR74a2:gi|751451750|ref|XP_011180216.1|;RzepOR74a2:gi|1048032172|ref|XP_017490047.1|;CcapOR74a2:</td></tr>
<tr><td>OR82a</td><td>DmelOR82a:NP_730794.1;BminOR82a:Bmi006911;BdorOR82a:XP011208732.1,XM_011210430.2,gi|751797704|ref|XP011208732.1|;BcucOR82a:gi|751454951|ref|XP_01</td></tr>
<tr><td>OR83a</td><td>DmelOR83a:NP_524234.2;BminOR83a1:Bmi011217;BdorOR83a1:XP011203872.1,XM_011205570.1,gi|751788791|ref|XP011203872.1|;BcucOR83a1:gi|751458907|ref|X
1:gi|751458909|ref|XP_011184142.1|;BcucOR83a2-2:gi|751458911|ref|XP_011184143.1|;CcapOR83a2:ref|XP_004518314.1|.</td></tr>
<tr><td>OR85bc</td><td>DmelOR85b:NP_524279.2;DmelOR85c:524280.2;BdorOR85bc1:XP_011209575.1:XM_011211273.2,gi|751799245|ref|XP011209575.1|;BcucOR85bc1:gi|751474275|ref|X
997|ref|XP_014093774.1|;BlatOR85bc2X1:JAI32382.1,gi|1098686968|ref|XP_018801757.1|;BlatOR85bc2X2:gi|1098686972|ref|XP_018801758.1|;Ccap85bc2:ref|XP_0121</td></tr>
<tr><td>OR85d</td><td>DmelOR85d:NP_524281.1;BminOR85d1:Bmi001692;BdorOR85d1:XP011209577.2,XM_011211275.2,gi|1132391151|ref|XP011209577.2|;BcucOR85d1:gi|751474277|ref|
929367993|ref|XP_014093772.1|;RzepOR85d2:gi|1048024056|ref|XP_017472634.1|.</td></tr>
<tr><td>OR85e</td><td>DmelOR85e:NP_001262374.1;BminOR85e:Bmi007558;BdorOR85e:XP011198148.1,XM_011199846.1,gi|751778226|ref|XP011198148.1|;BcucOR85e1-1:gi|751466515|ref</td></tr>
<tr><td>OR88a</td><td>DmelOR88a:NP_524348.2;BdorOR88a:AKI29048.1;BcucOR88a:gi|751456895|ref|XP_011183038.1|;BminOR88a:Bmi007186;BlatOR88a:gi|1098651616|ref|XP_0187904</td></tr>
<tr><td>OR94ab</td><td>DmelOR94a:NP_524455.1;DmelOR94b:NP_524456.1;BminOR94ab1:Bmi011849;BdorOR94ab1:XP011211752.1,XM_011213450.1,gi|751803245|ref|XP011211752.1|;Bla</td></tr>
<tr><td>ORCO</td><td>DmelORCOX1:NP_524235.2;DmelORCOX2:NP_001097687.1;BminORCO:Bmi011214;BdorORCO:AKI29027.1,JAC55447.1,gi|751788618|ref|XP_011203778.1|;BcucO</td></tr>
<tr><td>OR1</td><td>BminOR1-1:Bmi010399;BdorOR1-1:XP019845303.1,XM_019989744.1,gi|1132381746|ref|XP019845303.1|;BcucOR1-1:gi|751450861|ref|XP_011179733.1|;BlatOR1-1:gi|</td></tr>
<tr><td>OR2</td><td>BminOR2-1:Bmi012274;BdorOR2-1:XP011208898.1,XM_011210596.1,gi|751798009|ref|XP011208898.1|;BcucOR2-1:gi|751447842|ref|XP_011178076.1|;BoleOR2-1:gi|9
3:gi|1048000289|ref|XP_017465327.1|;BlatOR2-3:gi|1098621988|ref|XP_018803914.1|;BdorOR2-3:XP011208900.1,XM_011210598.1,gi|751798013|ref|XP011208900.1|;</td></tr>
<tr><td>OR3</td><td>BminOR3-1:Bmi001029;BcucOR3-1:gi|751439955|ref|XP_011187028.1|;BoleOR3-1:gi|929379425|ref|XP_014100035.1|;RzepOR3-1:gi|1048050793|ref|XP_017480493.1|;
2:gi|1048060106|ref|XP_017483505.1|;BdorOR3-2-1:gi|1132392534|ref|XP019847596.1|;BdorOR3-2-2:XP019847598.1,XM_019992039.1,gi|1132392538|ref|XP019847598
2:gi|929374815|ref|XP_014097486.1|;BcucOR3-2X1:gi|751478530|ref|XP_011194820.1|;BcucOR3-2X2:gi|751478532|ref|XP_011194821.1|;BlatOR3-2-3:gi|1098676554|ref</td></tr>
<tr><td>OR4</td><td>BminOR4-1:Bmi006901;BdorOR4-1:AKI29044.1,XP011200400.1,XM_011202098.1,gi|751782391|ref|XP_011200400.1|;BcucOR4-1:gi|751440693|ref|XP_011191024.1|;B
1:gi|751440691|ref|XP_011191013.1|;CcapOR4-1-1:ref|XP_020716968.1|;CcapOR4-1-2:ref|XP_004535567.2|.</td></tr>
</table>

<table>
<tr><td>OR5</td><td>BminOR5-1:Bmi005394;BdorOR5-1:XP011199471.1,XM_011201169.1,gi|751780677|ref|XP011199471.1|;BcucOR5-1:gi|751439923|ref|XP_011186833.1|;BlatOR5-1:gi|10</td></tr>
<tr><td>OR6</td><td>BminOR6-1:Bmi007806;BdorOR6-1:XP011209441.1,XM_011211139.2,gi|751798998|ref|XP011209441.1|;BcucOR6-1:gi|751457300|ref|XP_011183260.1|;BlatOR6-1:gi|10
2:gi|751457302|ref|XP_011183261.1|;BoleOR6-2:gi|929380937|ref|XP_014100884.1|;BlatOR6-2:gi|1098623000|ref|XP_018804184.1|;CcapOR6-2:ref|XP_020715917.1|;Rze</td></tr>
<tr><td>OR7</td><td>BminOR7-1:Bmi011393;BdorOR7-1:XP011210512.1,XM_011212210.1,gi|751800979|ref|XP011210512.1|;BcucOR7-1:gi|751460080|ref|XP_011184786.1|;BlatOR7-1:gi|10</td></tr>
<tr><td>OR8</td><td>BminOR8-1-1:Bmi002251;BminOR8-1-2:Bmi001974;BminOR8-1-3:Bmi012276:;BdorOR8-1-:AKI29037.1,XP011212981.2,XM_011214679.2,gi|1132395203|ref|XP01121
3:gi|751447848|ref|XP_011178079.1|;BminOR8-2-1:Bmi009485;BdorOR8-2-1:AKI29032.1,gi|1132380798|ref|XP019845111.1|;BcucOR8-2-1:gi|751465622|ref|XP_011187
2:ref|XP_004518300.1|;CcapOR8-2-3:ref|XP_020717560.1|;CcapOR8-3:ref|XP_004535438.2|.</td></tr>
<tr><td>OR9</td><td>BminOR9:Bmi007416;BdorOR9:XP011201756.1,XM_011203454.1,gi|751784886|ref|XP011201756.1|;BcucOR9:gi|751452095|ref|XP_011180402.1|;BoleOR9:gi|92936562</td></tr>
</table>
