# Supplementary material for: Comparative genomic and transcriptomic analyses of chemosensory genes in the citrus fruit fly Bactrocera (Tetradacus) minax
Source: Sci Rep. 2020 Oct 22;10:18068. doi: 10.1038/s41598-020-74803-5 (PMC7583261; doi:10.1038/s41598-020-74803-5)
Supplement: Supplementary file 5 — Supplementary Information 5. [file 41598_2020_74803_MOESM5_ESM.pdf]

Supplementary file 4

>BminOBP8a [gene=OBP8a] [protein=odorant-binding protein 8a] [organism=Bactrocera minax] [moltype=protein] [country=China] [ID=Bmi011412] [locus=Contig914:542161:543131:+ len:468nt odorant-binding protein 8a]
MKGTALVFIIPFSALFANINADYEEKTEDDFLSAGERCFQRERLAASYQHRFDNFDYPDEQ
PVHRYVHCIWTELKLWNDRTGFNVEHIAALYRDKANTEVLVPILSDCNRNAQNDPILNWC
YKAFKCVLNSRVGQWFKEDVGRKLQERHVGNHVA*
>BminOBPlush [gene=OBPlush] [protein=odorant-binding protein lush] [organism=Bactrocera minax] [moltype=protein] [country=China] [ID=Bmi007112] [locus=Contig2134:556799:557867:- len:330nt odorant-binding protein lush]
MQQFETSLDMMRNGCAPKFKVSTEILDNLRAGEFAENNSDLKLTKKGDFSVQKALAQIPI
ILPPEMQDAAKEALNACKDVQKNYKDSCDRVFYTTKCVRDYDPSTFKFP*
>BminOBP19a1 [gene=OBP19a1] [protein=odorant-binding protein 19a1] [organism=Bactrocera minax] [moltype=protein] [country=China] [ID=Bmi003171] [locus=Contig1349:2158519:2162429:- len:348nt odorant-binding protein 19a1]
MLNKINPFILTAVFIALVLHSDQVSGGATEDQMISAGKLMRDVCLPKFNKMKKGKFLYESS
LKQVDLLMPDSYKDDYRNGLGKCKDVANGIKNNCDASYAVLICLRDNISKFVFP*
>BminOBP19a2 [gene=OBP19a2] [protein=odorant-binding protein 19a2] [organism=Bactrocera minax] [moltype=protein] [country=China] [ID=Bmi003172] [locus=Contig1349:2167677:2170122:+ len:444nt odorant-binding protein 19a2]
MNFCKVLFTTSVLAFLFATSISAGVTEEQMWATAKLMRDVCLPRFPKISIELANQLRDGNIP
DNNKDVKCYINCVLEMMQTMKKGKFLYEASLKQVDLVLPDNYKDDYRAGLLKCKDAN
GGIKRDNCEAAYRILKCLCGEIKKFIFP*
>BminOBP19b [gene=OBP19b] [protein=odorant-binding protein 19b] [organism=Bactrocera minax] [moltype=protein] [country=China] [ID=Bmi003173] [locus=Contig1349:2174610:2175352:- len:465nt odorant-binding protein 19b]
MFKVFTVFTGTILIIVTREVLAEEMMTLPMSLLLETIEPYAMECEPNPERVHAEELFLNKED
AHPITKCLRRCLMDQFELFAEDSTDVNTEKLVSWMVLLYSEKMDELKELSNGCNEKTIEM
GIIDKCEVAHSFAMCMLKEMREREYEIPEVEQ*
>BminOBP19c [gene=OBP19c] [protein=odorant-binding protein 19c] [organism=Bactrocera minax] [moltype=protein] [country=China] [ID=Bmi003174] [locus=Contig1349:2177516:2178547:- len:555nt odorant-binding protein 19c]
MANSSSVLLFAGFICLMAVQSLSALNEDSHKLFGKRKPLMTREDPSTLEDYKRTKRQLSQ
PLQDFQDFIITSKTQCAREMDINPNELQKALLYEDQPTPKEKCLMECILKRMEVMDKDDT
LSTSAIGRIADIIGENNALITSIAMATAENCKKFITAKDSCERAYQINKCIAAEMKMRKIKLI
Y*
>BminOBP19d1 [gene=OBP19d1] [protein=odorant-binding protein 19d1] [organism=Bactrocera minax] [moltype=protein] [country=China] [ID=Bmi003187] [locus=Contig1349:2323057:2325125:- len:426nt odorant-binding protein 19d1]
MKFLNIGLIVCVTLISNAMCDYEEAKAAAELCKEEVGATDDDVETILKFEPAGTMEAKCL
HACIIKRFGVMNGDGKIDRDKAMEILTIIASGNGEQQALGVEVLEACADIDVNEDHCEAA
EEYRTCMHAKAKENGFIMGRV*
>BminOBP19d2 [gene=OBP19d2] [protein=odorant-binding protein 19d2]

[organism=Bactrocera minax] [moltype=protein] [country=China] [ID=Bmi003188] [locus=Contig1349:2327243:2328975:- len:432nt odorant-binding protein 19d2]
MKYFVVFLAICSSAICFSEAGELLEKVKNIAEECKNQVGASDDDVAGLLKYEPAANDKAK
CLRACTMKKFGIMDENNKLVEAAAIDYIKAVAAGDAEFEKLSTEVFNECKNTPASSDECE
FAETFSSCIIENSKSKGIKILPQ*
>BminOBP19d3 [gene=OBP19d3] [protein=odorant-binding protein 19d3] [organism=Bactrocera minax] [moltype=protein] [country=China] [ID=Bmi003186] [locus=Contig1349:2317823:2320040:- len:477nt odorant-binding protein 19d3]
MEVFKVLPIFALLSLVFAIIAADTGSNPPHYSSLRVMAEAAIEDCYEDAAESVKVQITDESF
DEIVKGSRTNLSRNAKCLRYCIMRKNGLLNEDNSIDRENILQIFQIIHPQIEKDFPLDVIQKC
SREMDKQADNCECAFVASSCILRELQVDGVTDI*
>BminOBP28a [gene=OBP28a] [protein=odorant-binding protein 28a] [organism=Bactrocera minax] [moltype=protein] [country=China] [ID=Bmi003166] [locus=Contig1349:2007997:2008688:- len:444nt odorant-binding protein 28a]
MAKFILIAALCILSAVVSKAAFNKEEVIKTFMTRAEECRGEVGAADSDVQDILSKLPAAGK
EGKCLRNCLMKKYSAVDNNGKFVKSVAEEHAQIYTDGDADKMKIAHEIIDACAGIPVPD
DPCEAAEVYGKCFLEQAKAHGIEKFDF*
>BminOBP47a [gene=OBP47a] [protein=odorant-binding protein 47a] [organism=Bactrocera minax] [moltype=protein] [country=China] [ID=Bmi004519] [locus=Contig1553:5809231:5809976:- len:666nt odorant-binding protein 47a]
MDSKLIVLTATMLAAIVELTPFLSVEARSASVKMSLDLSVSEDQHRISADMVSLCALETDL
SMGELRRFSENDFRNATKAIECFTHCLYEHMGLLNNGVFVERDIISLLGDVTDLKRMLER
ECLGQYSDNKCERAFLIHQCYRAGQRVMMAPNRQPESIDQLNEERDGEEEKSVANLPKS
DMAMPSVHRMATDSERQLLIKNILAKRLPKKQTINQGLVEE*
>BminOBP50c [gene=OBP50c] [protein=odorant-binding protein 50c] [organism=Bactrocera minax] [moltype=protein] [country=China] [ID=Bmi005981] [locus=Contig1765:1515036:1516529:+ len:765nt odorant-binding protein 50c]
MKYFVGSLLLILLLGVNAYDFDDSVFNEYLFKELQSHYEEDQVYLHRARREATDAADAK
ECSKRNWKKDMQCCKGGNVIGDQLELFKSVKKQCIADLKGEPADDAFDPFDCEKMQQV
KEQMICITECIAKKFKSLDENGELQRDAILEGLRAQIGTVQWKVDAIEGYVDKCLAEVKE
KLDQKKKAGELKEGGCSRSPLAFHSCMWRQFWNGCPADLRVDSPKCNKLRERVANGDT
RFFGKHFLNKYYPSPRDEE*
>BminOBP56a [gene=OBP56a] [protein=odorant-binding protein 56a] [organism=Bactrocera minax] [moltype=protein] [country=China] [ID=Bmi006859] [locus=Contig2076:264209:264928:- len:501nt odorant-binding protein 56a]
MKSSIICCILATVLLSLCTFGTDAALGRKPKKLTPELESKFEVLTAWIAYRLNLKHAKEACV
GEYGFSDELATNLVKIRVANPSDNEKCYVNCLYNKLVFYKDNAINKQAMKESLYEIVGEQ
RLMNIVNGCLNAGGTNACDKVYKFHACASPEFDKVRSDIFLPDE*
>BminOBP56b [gene=OBP56b] [protein=odorant-binding protein 56b] [organism=Bactrocera minax] [moltype=protein] [country=China] [ID=Bmi006857] [locus=Contig2076:216452:217185:- len:417nt odorant-binding protein 56b]
MKSLGFLYLFVAVFICGPITQLYAADDKAAREICIKETKLTPADANLVRGAAVISKLIQNEPE
ALKCFRLCYYKELGLIDAAGKTNAPKILEYMMQVSGVSDKTRLASALGACESVKGTSNC
DKWYQFEKCALAKLGA*

>BminOBP56c [gene=OBP56c] [protein=odorant-binding protein 56c] [organism=Bactrocera minax] [moltype=protein] [country=China] [ID=Bmi006858] [locus=Contig2076:218158:219269:- len:852nt odorant-binding protein 56c]
MYYIISLFAILLCAAVQHAKSRTITLSVNMSLTMGVERHKELLEMRHQQRQQEQTRPAFTA
TLLRSCMKQTELSMAELQHFRLSLFCRDPELDCSDEPSTELPNFYEMEEDNLVSGETQIDG
IIYEPASDINEQNNNALEYEDPLTIKSDGKTDESLQCFVYCLYEQLGLIAKGVYMENELFAK
LYALVGRERHLVKECMNLNTNNKCESSYKMHLCYASLKILEEENRIRKILENAKDDQERE
EILEAETAQETHAMEETEPTQSIFAYAEGAKTEEVGVEQ*
>BminOBP56d [gene=OBP56d] [protein=odorant-binding protein 56d] [organism=Bactrocera minax] [moltype=protein] [country=China] [ID=Bmi006856] [locus=Contig2076:208209:208978:- len:414nt odorant-binding protein 56d]
MKFFTIVAVFAFVAVAAAQEGIKLSEEQKQKVHTLGAECLTETGASEAAVRAVGKGDLSQV
DDKVKCFAKCLQGKLGYFENGQVNEAVVQSSLGKVVGEEKIKAIQAKCNGLKGTDDCD
TAFLLHKCYVAENASTLV*
>BminOBP56g [gene=OBP56g] [protein=odorant-binding protein 56g] [organism=Bactrocera minax] [moltype=protein] [country=China] [ID=Bmi006852] [locus=Contig2076:13872:14887:+ len:516nt odorant-binding protein 56g]
MKSFITIALFVVSCAVVLCNANDPEMRKQIEECNKEHNVTPKDYHDFMEGKLTTIPENLK
CSSHCVMMKQGIMDDSGKFKAEVAKAKMNDDKFSATVDECKDLSGSTPCDTAMKITEC
LLSHK*
>BminOBP56h1 [gene=OBP56h1] [protein=odorant-binding protein 56h1] [organism=Bactrocera minax] [moltype=protein] [country=China] [ID=Bmi006853] [locus=Contig2076:112291:113005:+ len:408nt odorant-binding protein 56h1]
MHTLYVLTIIVALVTLAVCQSPADLEKFHKACMDEAKVTNEELKKFFQNGMKYDAAKENI
KCHTKCLMQKHGVWKNGAFDAEAKAKELMQIPKLKEHEVQIKQALSNCKNEKGANEC
DTAFKITMCLKEFKAQID*
>BminOBP56hlike [gene=OBP56hlike] [protein=odorant-binding protein 56h-like] [organism=Bactrocera minax] [moltype=protein] [country=China] [ID=Bmi008922] [locus=Contig3340:32457:32990:- len:402nt odorant-binding protein 56h-like]
MKVVLLCFVSALLVAAQAEVPLKELIETCSKETGVPSEELQQFLDSEMDPAEATNGIKCH
MKCVLEKIGLYKNNMLDEALTIKFFKDNNKDQPVDVEALKQSVQNCNQKKGVDPCDTA
FQIAKCFMSQHPIFN*
>BminOBP58c [gene=OBP58c] [protein=odorant-binding protein 58c] [organism=Bactrocera minax] [moltype=protein] [country=China] [ID=Bmi002479] [locus=Contig1300:1346081:1386904:- len:606nt odorant-binding protein 58c]
MKNSTLIIFSFAWLLKFGNSLKIDCDNPEFIREDRIHYCCKHPDGYQEFVDSCAKETGFNYI
QSNEESIVDITADHATMGTCFAKCVFNKLQFMKGDELDMSAVRQHFESKYKADPEYAKE
MINAFDHCHGKSVENTAKFLSNPIFHNIGAEFCDPKPGVIMACVIREFFHNCPADRWAKTE
ECNTVLEFSKKCKDALTTI*
>BminOBP73a [gene=OBP73a] [protein=odorant-binding protein 73a] [organism=Bactrocera minax] [moltype=protein] [country=China] [ID=Bmi005271] [locus=Contig1697:132056:133593:+ len:795nt odorant-binding protein 73a]
MISNVVYRAYIALILLDRAGILAKDLHGNSFTKVLRSKGSQLSRCLNPPRTARRVESFIQDC
QEDVKNKLFSEAYYILKSEVNKEYTELDITVDNIAEIDSTPASPAAPSLIFHTIEGNSNLASS

HNSYQFQSDRQQVTSLMHRIGRISYDPRSAIYHPTLVPAEEKRLAGCLLHCVYAKNNAIDK
LGWPTLDGLVNFYSEGVNEHGFFMATLRSVNLCLHAITIKYNINRRKLPKLGESCDLAFD
VFDCISDQLTGYCLNQYE*

>BminOBP83a [gene=OBP83a] [protein=odorant-binding protein 83a] [organism=Bactrocera minax] [moltype=protein] [country=China] [ID=Bmi002228] [locus=Contig1291:2252022:2252987:+ len:447nt odorant-binding protein 83a]

MYSIKSILGTLLWFGFLTTVIWAQKELRRDETYPPPELLKALRPVHDSCVSKTGVTEEAIKE
FSDGDVHEDELLKCYMYCVFEETDVLHEDGEVHLEKILDSLPQSMHDIALHMGKKCLYP
KGDNKCERAFWLHRCWKESDPKHYFLI*

>BminOBP83b [gene=OBP83b] [protein=pheromone-binding protein-related protein 83b] [organism=Bactrocera minax] [moltype=protein] [country=China] [ID=Bmi002229] [locus=Contig1291:2258164:2261909:+ len:531nt pheromone-binding protein-related protein 83b]

MVLTGIWRCQAFNAFLIVALSSSLTLMHVQAQEPRRDDKWPPPAVLKMAKIFHDICVEKT
GVTEEAIKEFSDGQIHEDEALKCYMNCLFHEIDVVDDNGDVHLETLYNTVPGTVRDKLIN
MARGCEHPEGDTLCHKAWWFHQCWKKADPVIAEVRGGRPATRTPLSSPAALVTPAF*

>BminOBP83cd [gene=OBP83cd] [protein=odorant-binding protein 83cd] [organism=Bactrocera minax] [moltype=protein] [country=China] [ID=Bmi002221] [locus=Contig1291:2006182:2011265:- len:1599nt odorant-binding protein 83cd]

MKLILIFLFAAAIISPSVAVVQPNAEEVRIISECLQSYGGLTEDNSQRLVRFKDWSEKYEEIPC
FTKCYIKNMFDMFDESVGFKDEQVIKQFGQPLYIACEHRMKPSTDNCQQAYNGFHCLVN
LEDDPFVIIESMKNVSTEAKTAMKDCLHRFDQYEWERIKDYAKNPVREPIPCFTKCFIDRL
QLYSQQTRQWNIPALTAKLGVPAAGANIQHCLKQRRNRNACVNSAATTKAANQDVE

>BminOBP83g [gene=OBP83g] [protein=odorant-binding protein 83g] [organism=Bactrocera minax] [moltype=protein] [country=China] [ID=Bmi002220] [locus=Contig1291:2004665:2005886:+ len:429nt odorant-binding protein 83g]

MQTQLILLMACVALVAGKFHVRTAQDALDAHEACHEEYRVPEDIYQKFLNYEFAPHKRTN
CYVKCFVERMGLFTEEKGFDEKAIIAQFTAKSSKNLAKISHGLEKCLDHNEHDSDTCTWA
NRVFSCWISVNRPIVRRTYIEN*

>BminOBP84a2 [gene=OBP84a2] [protein=odorant-binding protein 84a2] [organism=Bactrocera minax] [moltype=protein] [country=China] [ID=Bmi011107] [locus=Contig900:3020516:3021247:- len:513nt odorant-binding protein 84a2]

MSNSNVLVVCPFMIMLLYCSIMISIQDRAKDNGDIFVQHKEQQECVRPIIVQANDSISSEGT
DVVLMCNSSFSIPSDYIAQFNMNGALSDTLDKTGMCFIRCYFEKAGLIKNWQLNKDMIM
QTMRLIKANSIEFCEPEAKQETNACIRTYAIAKCLMKRGFEDTCIQTVA*

>BminOBP99b [gene=OBP99b] [protein=odorant-binding receptor 99b] [organism=Bactrocera minax] [moltype=protein] [country=China] [ID=Bmi011411] [locus=Contig914:535616:536235:+ len:465nt odorant-binding receptor 99b]

MKFFLALLSLIFAVVLADEHDHAAHSDYVVKTNADLLRYRDECVSKLSIPADLVEKYKQ
WNFPDDEKTPCYLKCILEKFGLYDEEKGFDVHKIHHQLDGDKVDHSDATHGAIENCAKE
AAAAGDDACVRAYRGFSCFLKDNIQLVQAGVDKGSK*

>BminOBP99d [gene=OBP99d] [protein=odorant-binding protein 99d] [organism=Bactrocera minax] [moltype=protein] [country=China] [ID=Bmi011413] [locus=Contig914:542542:560860:- len:672nt odorant-binding protein 99d]

TEELFNPATAVPISEYEEASRRLYKAQGICIQKISSPTTRDNVASTEQKAGAYVRCIASNMGL

WTDATGYNSKRVAKFFIKEHNENEVMTVVDYCNQKHQQADLDLWAFEAYRCATAGRMGIWLREYVLSAKL*

>BminOBP99c1 [gene=OBP99c1] [protein=odorant-binding protein 99c1] [organism=Bactrocera minax] [moltype=protein] [country=China] [ID=Bmi011415] [locus=Contig914:665977:666592:+ len:450nt odorant-binding protein 99c1]

MKFFIVILAVVALACAEDEWVPKNVAEIKVIRQECIKEFSLSEEHIQKLKNLEYPDEEPVRKYLLCTAEKLGVFCEHEGFHANRIAKQFKMDLDEAEVLAIAEGCVDKNEEGSSPDVWAYRGHKCLIDSKIGESVKAYIKKSADEAKKQ*

>BminOBP99c2 [gene=OBP99c2] [protein=odorant-binding protein 99c2] [organism=Bactrocera minax] [moltype=protein] [country=China] [ID=Bmi011416] [locus=Contig914:669005:669795:- len:426nt odorant-binding protein 99c2]

MKYFIVILAIIALVQADDWSPKTTDEIKSIRENCVKQVPSSDEEFKNRKKDEYPDIESVRNYVLCTSKAWGLYEDGKGFKGEHVAQQFKGDLSEDEIKSIVHDCDEKTKEDSDDERAYHVLMCILSSKLGGDVKELLKRSE*

```
                    10        20        30        40        50        60        70        80        90       100       110
BdorOBP8a   : MPQAMKCTAFVF---IILFAALFAH---INADYEEKTEDDFLSAGERCFQRERLAASYQRRFDNFDYPDEEPVQRYVHCIWTELKLWNDRTGFNVEHIAALYR--DKANTE : 103
BlatOBP8a   : MXQAMKCTAFVF---ILLFAALFAH---INADYEEKTEDDFLSAGERCFQRERLAASYQRRFDNFDYPDEEPVQRYVHCIWTELKLWNDRTGFNVEHIAALYR--DKANTE : 103
BoleOBP8aX1 : MPQAMKCTVFVH---IILFAALFAH---INADYEEKTEDDFLSAGERCFQRERLAAPYQRRFDTFDYPDEEPVHRYVHCIWTELKLWNDRTGFNVEHIAALYR--DKANTE : 103
BoleOBP8aX2 : MPQAMKCTVFVH---IILFAALFAH---INADYEEKTEDDFLSAGERCFQRERLAAPYQRRFDTFDYPDEEPVHRYVHCIWTELKLWNDRTGFNVEHIAALYR--DKANTE : 103
BminOBP8a   : ----MKGTALVF---IIPFSALFAN---INADYEEKTEDDFLSAGERCFQRERLAASYQHRFDNFDYPDEQPVHRYVHCIWTELKLWNDRTGFNVEHIAALYR--DKANTE :  99
BcucOBP8a   : MPQAMKYTAFII---IILFSTLIAL---INADHEETTEDDFLSAGQRCFQRERLAASYQRRFDNFEYPDEEPVHRYVHCIWTELKLWNDRTGFNVEHIAALYR--DKANTE : 103
CcapOBP8a   : MTEAMKCSTIAFTWIIILLFNFCNF---IGADFEEKTEEDFLTASERCFQRERLAASYQRQFDNFVYPDEAPVHRYVHCIWNELKLWNDRTGFNVEHIAALYR--DKANTE : 106
RzepOBP8aX2 : ----MKRTTLAY---IIFLFTFCMP---ISADFEEKTEDDFLTASEHCYERERLPASYQRRFDNFDYPNEEPVHRYVHCIWTELKLWNDRTGFNVEHIAALYR--DKANTE :  99
RzepOBP8aX1 : ----MKRTTLAY---IIFLFTFCMPYGQISADFEEKTEDDFLTASEHCYERERLPASYQRRFDNFDYPNEEPVHRYVHCIWTELKLWNDRTGFNVEHIAALYR--DKANTE : 102
DmelOBP8a   : ---MMRRSQIGLLSRLLLLLLVVELTPPAIPVPMRSSPQSLALLRARDQCGRELTAAQRLQLDRMQFEDAAHVRHYLHCFWSRLQLWLDETGFQAQRIVQSFGGERRLNVE : 108
                  Mk t       ii           i ad eekteddfl  a erc  rerL A yqrrfD f ypde pV rYvHCiWteLkLWnDrTGFnvehIaalyr  dkaNtE

                 120       130       140       150       160       170
BdorOBP8a   : VLVPILSDCNRNAQN-----EPTLKWCYRAFKCVLNSRVGQWFKEDVGRKLEERRVGNHVA : 159
BlatOBP8a   : VLVPILSDCNRNAQN-----EPTLKWCYRAFKCVLNSRVGQWFKEDVGRKLEERRXGNHVA : 159
BoleOBP8aX1 : VLVPILSDCNRNAQN-----EPSLKWCYRAFKCVLNSQVGQWFKEDVERKLQERRVGNHVA : 159
BoleOBP8aX2 : VLVPILSDCNRNAQN-----EPSLKWCYRAFKCVLNSQVGQWFKEDVERKLQERRVGNHVA : 159
BminOBP8a   : VLVPILSDCNRNAQN-----DPILNWCYKAFKCVLNSRVGQWFKEDVGRKLQERHVGNHVA : 155
BcucOBP8a   : VLVPILSECNRNTQN-----EPTLRWCYKAFKCVLNSRVGQWFKEDVGRKLHERRVGNHVG : 159
CcapOBP8a   : VLVPILSDCNRNTNN-----APTLKWCYNAFKCVLNSRVGQWFKEDVERKLHERRTGNHVA : 162
RzepOBP8aX2 : VLVPILSDCNRNAQN-----EPTLKWCFNAFKCVLNSRVGQWFKEDVGRKLQDTKTTNHLG : 155
RzepOBP8aX1 : VLVPILSDCNRNAQN-----EPTLKWCFNAFKCVLNSRVGQWFKEDVGRKLQDTKTTNHLG : 158
DmelOBP8a   : QALPAINGCNAKTSSRGSGAQTVVDWCFRAFVCVLATPVGEWYKRHMSDVIN----GNA-- : 163
              vlvPilsdCNrn qn      p l WC  AFkCVLns VGqWfKedv rkl     gNh
```

Figure S4-1 The alignment of OBP8a

```
                      10        20        30        40        50        60        70        80        90       100       110
BdorOBP19a2   : --------------------------------MWATAKLMRDVCLPRFPKISIELANQLRDGNIPDNNKDVKCYINCVLEMMQTMKKGKFLYEASLKQVDLVLPDSYKDD :  78
BlatOBP19a2   : MMYFCKAIFTTSILAFLFLTP--ISAGITEEQMWATAKLMRDVCLPRFPKINIELANQLRDGNIPDDNKDVKCYINCVLEMMQTMKKGKFLYEASLKQVDIVLPDSYKDD : 108
BcucOBP19a2   : -MHFCKVFLTTSVLAFLFATP--ISAGVTEEQMWATAKLMRDVCLPRFPKISIELANQLRDGNIPDDNKDVKCYINCVLEMMQTMKKGKFLYDASLKQVDLVLPDSYKDD : 107
BminOBP19a2   : -MNFCKVLFTTSVLAFLFATS--ISAGVTEEQMWATAKLMRDVCLPRFPKISIELANQLRDGNIPDNNKDVKCYINCVLEMMQTMKKGKFLYEASLKQVDLVLPDNYKDD : 107
RzepOBP19a2   : --------------------------------MWATAKLMRDVCLPRFPKISFELADQLRDGNIPDNNKDVKCYINCVLEMMQTMKKGKFLFDASL-------------- :  64
CcapOBP19a2X2 : --------------------------------MWATAKLMRDVCLPRFPKIATELADQLRDGNIPDNNKDVKCYINCVLEMMQTMKKGKFLYEASLKQVDLILPDSYKDD :  78
CcapOBP19a2X1 : -MKYFKVLLTSCIMVLFFIAP--TFAGVTEEQMWATAKLMRDVCLPRFPKIATELADQLRDGNIPDNNKDVKCYINCVLEMMQTMKKGKFLYEASLKQVDLILPDSYKDD : 107
BdorOBP19a1   : MLNKINSFVLATVFIALVLHSDQVSGGATEEQMISAGKLMRDVCLPKFSKISPEVADGIKEGNVPD-TKDVKCYINCIMEMMQTMKKGKFLYESALKQIDLLMPDDYKDD : 109
BlatOBP19a1   : MLNKINSFVLATVFIALVLYSDQVSGGATEEQMISAGKLMRDVCLPKFNKISPEVADGIKEGNVPD-TKDVKCYINCIMEMMQTMKKGKFLYESALKQIDLLMPDNYKDD : 109
BoleOBP19a1   : MLKKINPFVLATVFIALVLHSDQVSGGATEEQMISAGKLMRDVCLPKFNKVSPEVADGIKDGNVPD-TKDVKCYINCIMEMMQTMKKGKFLYESALKQIDLLMPDNYKED : 109
BminOBP19a1   : MLNKINPFILTAVFIALVLHSDQVSGGATEDQMISAGKLMRDVCLPKFNK----------------------------------MKKGKFLYESSLKQVDLLMPDSYKDD :  76
BcucOBP19a1   : MSNKINLFVLVAVFIALVLRSDQVFGGATEEQMIAAGKLMRDVCLPKFNKITPEVADGIKEGKVPD-TKDVKCYINCVMEMMQTMKKGKFLYESSLKQVDLLMPDSYKDE : 109
CcapOBP19a1   : MLPRMNLFVLPAVLIVLTLNTDRVYGGATEEQMIAAGKLMRDVCLPKFKVPT-DVADGIKDGIVPE-TKEVKCYINCILEMMQTMKKGKFLYESSLKQVDLLMPDDYKDQ : 108
RzepOBP19a1p  : -------------------------------------DLY--TFIFSFP----EVADGIKLGNIPD-TKDVKCYINCVLEMMQTMKKGKFLLESSLKQIDLLMPDDYKDD :  66
RzepOBP19a1   : --------------------------------MFAAGKLMRDVCLPKFPKVSTEVADGIKLGNIPD-TKDVKCYINCVLEMMQTMKKGKFLLESSLKQIDLLMPDDYKDD :  77
DmelOBP19a    : -MKFHLLLVCVAISLGPIPQS---EAGVTEEQMWSAGKLMRDVCLPKYPKVSVEVADNIRNGDIPN-SKDTNCYINCILEMMQAIKKGKFQLESTLKQMDIMLPDSYKDE : 105
                                                m    kLmrdvclp f k    e a      g  pd  kdvkcyinc  emmqtmKKGKFl e  Lkq dl  pd ykd

                       120       130       140       150
BdorOBP19a2   : YRAGLLKCKDASAGIKKD-NCEAAYTILKCLRGEIKKFIFP : 118
BlatOBP19a2   : YRAGLLKCKDASAGIKKD-NCEAAYTILKCLRGEIKKFIFP : 148
BcucOBP19a2   : YRGGLLKCKDAAVGIKKD-NCEAAYTILKCLRGEIKKFIFP : 147
BminOBP19a2   : YRAGLLKCKDANGGIKRD-NCEAAYRILKCLCGEIKKFIFP : 147
RzepOBP19a2   : ----------------------------------------- :   -
CcapOBP19a2X2 : YRAGLLKCKDASAHIKKD-NCESAYTVLKCLRGEIKKFIFP : 118
CcapOBP19a2X1 : YRAGLLKCKDASAHIKKD-NCESAYTVLKCLRGEIKKFIFP : 147
BdorOBP19a1   : YRNGLAKCKDVTSGIKN--NCDASYALLICMRDNISKFLFP : 148
BlatOBP19a1   : YRNGLAKCKDVTSGIKN--NCDASYALLICMRDNISKFLFP : 148
BoleOBP19a1   : YRNGLTKCKDVTSGIKN--NCDASYALLICMRDNISMFLFP : 148
BminOBP19a1   : YRNGLGKCKDVANGIKN--NCDASYAVLICLRDNISKFVFP : 115
BcucOBP19a1   : YRNGLGKCKDAAVGIKN--NCDASYALLICMRDNITKFVFP : 148
CcapOBP19a1   : YKSGFAACKDSPNGIKN--NCDASYALLICMRDKITKFVFP : 147
RzepOBP19a1p  : YRAGLQTCKDAVNGIKN--NCDAAYALLICIRGEIKRFIFP : 105
RzepOBP19a1   : YRAGLQTCKDAVNGIKN--NCDAAYALLICIRGEIKRFIFP : 116
DmelOBP19a    : YRKGINLCKDSTVGLKNAPNCDPAHALLSCLKNNIKVFVFP : 146
                yr gl  ckd   gik   nc   y  l c r  i  f fp
```

Figure S4-2 The alignment of OBP19a

```
                            10        20        30        40        50        60        70        80        90       100       110
BdorOBP19b : -----------------MNKVLTVFAVVVLMIATRKVLAEKIMKAPMGLALETLEPFAMNCKLKPEKAHMEELILNKEDADLITKCLRGCLMEQFQLFVEDSTDVNTEKLLSWM :  97
BlatOBP19b : -----------------MNKVLTVFSVVVLMIATKEVLAEEIMKVPMGLAVETLEPFAMNCEHKPEKVHMEELILNKEDADVLTRCLRSCLMEQFELFVEDSTDVNTEKLLSWM :  97
BoleOBP19b : -----------------MYKVFTVFAVIILMIATRKVLAEEIMKIPMGLVLEAVEPYAMNCEPKPEKAHMEELILNKEDAHLITKCLRGCLMEQFGLFVEDRTDVNSEKLVSWM :  97
BminOBP19b : -----------------MFKVFTVFTGTILIIVTREVLAEEMMTLPMSLLLETIEPYAMECEPNPERVHAEELFLNKEDAHPITKCLRRCLMDQFELFAEDSTDVNTEKLVSWM :  97
BcucOBP19b : ------------------MKGFIVFAAIILVMATKEVLAEEMMTLPMGLLLETIEPYARECEPKPERVHAEELFLNKEDAHPITKCLRRCLMDQFDLFVEDSTDVKTEKLVSWL :  96
RzepOBP19b : MEVFSVFLASSCKYSKKMLKYFIIFTVTTFISLTQNVQANDIMKLPMGLLLEGVAPYANKCDPKPELEHMEELFLFKEDAQHATKCFRRCLMDQFELFVEGGTQVHTEKLAYGM : 114
CcapOBP19b : --------------------MFHLCVAAFLISMMGDVLADEMMTLPIGMLIEGVEPFVQDCDPKPELEHMEELFLNKEDAQQTTKCLRHCLMAQFELFKEGDTAVESEKLVGFM :  94
DmelOBP19b : ------MMQCSRMTTTLKMTNLLLAVACAAVLMGSATADEEEGSMTVDEVVELIEPFGDACTPKPSRENIVEMVLNKEDAKHETKCFRHCMLEQFELMPEDQLQYNEDKTVDMI : 108
                               k    f    l   t  vlaee m  pmgl  E  eP a  C pkPe  h eEl LnKEDA   TkClR Clm QF Lf Ed t v  eKl   m

                  120       130       140       150       160       170
BdorOBP19b : VLLYSEKIDELRAISNGCNAKNVEMGITDKCEVAHSYAMCMLKEMKEREYEIPEVEQ : 154
BlatOBP19b : VLLYSEKIDELRAISNGCNAKNVEMGITDKCEVAHSFAMCMLKEMKEREYEIPEVEQ : 154
BoleOBP19b : VLLYVEKMDELIDISNGCNEKNVEMGITDKCEVAHSFAMCMLKEMKEREYEIPEVE- : 153
BminOBP19b : VLLYSEKMDELKELSNGCNEKTIEMGIIDKCEVAHSFAMCMLKEMREREYEIPEVEQ : 154
BcucOBP19b : VLLYTDKMDELNEISNGCNEKNVEMGITDKCEVAHSYAMCMLKEMQEREYEIPEVEQ : 153
RzepOBP19b : LLLYPDKMDELSEISDGCNEQNNQMGIEEKCEVAHSYAMCMLKEMQSRNYEIPEVEQ : 171
CcapOBP19b : ALLYPEKADELNEIVDGCNQRNKDMGLNEMCEVAHSFGMCMLKEMQSREYEIPIVEQ : 151
DmelOBP19b : NMMFPDREDDGRRIVKTCNEELKAE--QDKCEAAHGIAMCMLREMRSSGFKIPEIKE : 163
              lly  k Del  is gCN  n  mgi dkCEvAHs aMCMLkEM  reyeIPeveq
```

Figure S4-3 The alignment of OBP19b

```
                           10        20        30        40        50        60        70        80        90       100       110
BdorOBP19c   : MVKSSSALLIAGFICLMSLQSLTALSEDADKLSEKRKPLMTREDPSTLEDYKRTKRQLPQPLQEFQDFVTTSKTQCAKEMNINPNELQKSLLYEDQPTSMEKCMMECVLKRIE : 113
BlatOBP19c   : MVKSSSALII---ICLMSLQSLTALSEDADKLSGKRKPLMTREDPSTLEDYKRTKRQLPQPLQEFQDFVTTSKTECAKEMNINPNELQKSLLYEDQPTSIEKCMMECILKRIE : 110
BoleOBP19c   : MVNSSSALLIAGFICLMTLQSLNALIEDAYKLSGKRKPLMTREDPSTLEDYKRTKRQLPGPLQEFQDFITLSKAQCAKDLNINPNELQKSLLYEDQPTSTEKCMMECILKRME : 113
BcucOBP19c   : MANSSSALLIAGFICMMVVQSLDALNADVYKLSGKRKPLVTREDPTALEDYKRTKRQLPQPLQEFQDFITTSKTECAKEMNINPNELQKALLYEDQPTPKEKCMMECILKRME : 113
BminOBP19c   : MANSSSVLLFAGFICLMAVQSLSALNEDSHKLFGKRKPLMTREDPSTLEDYKRTKRQLSQPLQDFQDFIITSKTQCAREMDINPNELQKALLYEDQPTPKEKCLMECILKRME : 113
CcapOBP19c   : -MYSNKTLLFASLICLLAVESINALNANSYKLSGKRKPLATREDATIIEDYKRTKRQLSQPMQEFQAFITTSKGQCASEMGFKANEMEKSLLYEELPTPKEKCMMECILKRME : 112
RzepOBP19cX2 : ---------------------------------------MTRQNSLELGDYKRTKRQLTQPMQEFQDFITNSKTQCANGMGFSADELQKSLLYEDQPTRKEKCLIECILKRME :  74
RzepOBP19cX1 : MISLRNKMLCTVILCLAAFQSLNALDTNPYKLSGKRKPLMTRQNSLELGDYKRTKRQLTQPMQEFQDFITNSKTQCANGMGFSADELQKSLLYEDQPTRKEKCLIECILKRME : 113
DmelOBP19cX1 : -MKPSTPVAAIPLMTIVVAVLLQTHCVRGQTQAFDLAKLLPKTG------TEPIWAVIDRNLPQVQELVTAARMECIQKLQLPRD--QRPLGKVTNPSEKEKCLVECVLKKIK : 104
DmelOBP19cX2 : -MKPSTPVAAIPLMTIVVAVLLQTHCVRGQTQAFDLAKLLPKTG------TEPIWAVIDRNLPQVQELVTAARMECIQKLQLPRD--QRPLGKVTNPSEKEKCLVECVLKKIK : 104
                                    l                l tr       dykrtkrql  p q fQ f t sk  Ca         e qk Llye  Pt  EKC  EC LKr e

                   120       130       140       150       160       170       180
BdorOBP19c   : VMSKDDTLSTTTIGHIADIIGDNNALITSIAMASAENCKKFITAEDSCERAFQINKCIAAEMKMRKIKLIY : 184
BlatOBP19c   : VMSKDDTLSTTAIGYIADIIGDNNALITSIAVASAENCKKFITAEDSCERAFQINKCIATEMKMRKIKLIY : 181
BoleOBP19c   : VMDKNDSLSTTAIGHIADIIGENNALLTSIAMASAENCKKFITAEDSCERAFQINKCIGAEMKMRKIKLIY : 184
BcucOBP19c   : VMNQDDTLSTSAVGRIADIIGDNNALITSIAMASAENCKKFITAENSCERAYQINKCIANEMKMRKIKLIY : 184
BminOBP19c   : VMDKDDTLSTSAIGRIADIIGENNALITSIAMATAENCKKFITAKDSCERAYQINKCIAAEMKMRKIKLIY : 184
CcapOBP19c   : VMDRDNMLSTPAIGRIADIIGNNNALITSIAMASADNCKKFITAEDPCERAYQINKCIATEMKMRKIKLIY : 183
RzepOBP19cX2 : VMDKDNTLSTAAIGRIANIVGNNNPLITSIAMATADNCKKFINAEDSCERAHQINICIATEMKMRKIKLIY : 145
RzepOBP19cX1 : VMDKDNTLSTAAIGRIANIVGNNNPLITSIAMATADNCKKFINAEDSCERAHQINICIATEMKMRKIKLIY : 184
DmelOBP19cX1 : LMDADNKLNVGQVEKLTSLVTQDNKMAIAVSSSMAQACSRGISSKNPCEVAHLFNQCISRQLERNNVKLVW : 175
DmelOBP19cX2 : LMDADNKLNVGQVEKLTSLVTQDNKMAIAVSSSMAQACSRGISSKNPCEVAHLFNQCISRQLERNNVKLVW : 175
                vM  d  Lst   g ia i g nN l tsia a A nCkkfI a   CErA qiN CI  emkmrkiKLiy
```

Figure S4-4 The alignment of OBP19c

```
                            10        20        30        40        50        60        70        80        90       100       110
DmelOBP19dX1  : ----------------------------------MSHLVHLTVLLLVGILCLGATSAKPHEEINRDHAAELANECKAETG------ATDEDVEQLMSHDLPERHE-AKCLRA :  71
DmelOBP19dX2  : ----------------------------------MSHLVHLTVLLLVGILCLGATSAKPHEEINRDHAAELANECKAETG------ATDEDVEQLMSHDLPERHE-AKCLRA :  71
BdorOBP19d2   : -------------------------------------MKYFVVFLAICSFAISFS---EADEF-GEKVKKIAEECKGQVG------ASDDDVARLFKYEPAANDK-AKCLTA :  64
BlatOBP19d2   : -------------------------------------MKYFVLFLAICSLAISFS---EADEF-GEKVKKMAGECKEQVG------ASDDDVARLFKYEPAANDK-AKCLNA :  64
BoleOBP19d2   : -------------------------------------MKYFLVFLAICSLVISFS---EAEEF-LEKVKKIAEECKVQVG------ASDEDVAGVFKYEPAANDK-AKCLNA :  64
BminOBP19d2   : -------------------------------------MKYFVVFLAICSSAICFS---EAGEL-LEKVKNIAEECKNQVG------ASDDDVAGLLKYEPAANDK-AKCLRA :  64
BcucOBP19d2   : -------------------------------------MKYFVVCLAICSLVISHT---EAQEL-LEKIKQIAEECKGQVG------ASDDDVTRMLKYEPATNDK-AKCLQA :  64
BcucOBP19d2X1 : -------------------------------------MKYFVVSLVICSLVMSYS---EAQEL-PMRLKKIVEECKARLG------AGDDDVAAMFKYEPATNKQ-VKCLHA :  64
BcucOBP19d2X2 : -----------------------------------------------------------------MRLKKIVEECKARLG------AGDDDVAAMFKYEPATNKQ-VKCLHA :  40
RzepOBP19d2   : -------------------------------------MKYFVVFLFICGLATSYA---QLEEV-VKKAQKIAGDCKEEVG------ATDADVEAMFKHEPAGNDK-AKCLNA :  64
CcapOBP19d2   : MEVFKDSSDLYYDVYYPYSCDGLYLSMQLIAIPLNVYLKVIIAEILIRNVIINFVIYFQANDL-VDKVTKLAQECKGSTG------ASDADYENLLNYQAPNTDK-GKCLTE : 104
BminOBP19d1   : -------------------------------------MKFLNIGLIVCVTLISNA----MCD--YEEAKAAAELCKEEVG------ATDDDVETILKFEPAGTME-AKCLHA :  62
BoleOBP19d1   : -------------------------------------MKLFNICLIVCVALISNA----KCG--HEEAKAAAEECKDEVG------ATDDDVETILKFEPAETME-AKCLHA :  62
BdorOBP19d1   : -------------------------------------MKILNICLIVCVALISNA----KCN--YEEAKAVANECKEEVG------ATDDELETILKMEAAETTT-EKCLGA :  62
BcucOBP19d1-1 : -------------------------------------MKLLKICLILCVALISNA----KCG--SEEAKAAAEECKEEVG------ATDDDVEAMFKFKSAGSME-AKCLHA :  62
BcucOBP19d1-2 : -------------------------------------MKLLKIFLIVCVALISNA----KCS--FEEVKAAAEECKEEVG------ATDDDVAAMFNFESAGSME-AKCLHA :  62
CcapOBP19d1   : -------------------------------------MKFLNICLIVCAALIANT----KCDD-FEGVKLTAQECKDEVG------ATDEDVETMFKHEPAGSAE-AKCLHA :  63
RzepOBP19d1   : -------------------------------------MKSYFICLVVCAVFTSYV----ECD--LEKFKAVAEECKEEVG------ATDEDVETMINHEPAGTPE-AKCLHA :  62
BdorOBP19d3   : -----------------MEILIVLP---------ILAVSS-VFTITIA----AAESEPPHYSSLRVMAEAAIEDCYEDSAQSVKVQITDESFDEILKGSRTNLSHNAKCLRY :  81
BlatOBP19d3   : -----------------MEILIVLP---------ILAVSS-VFTITIA----AAESEPPHSSSLRVMAEAAIEDCYEDSAQSVKVQITDESFDEILKGSRTNLSHNAKCLRY :  81
BoleOBP19d3   : -----------------MEILIILP---------ILAVSS-VFIIKMA----AAESKPPHYSSLLVMAEAAIEDCYKDAAQSVKVQLTHESFDEILKGSRTNLSHNAKCLRY :  81
BminOBP19d3   : -----------------MEVFKVLP---------IFALLSLVFAIIAA----DTGSNPPHYSSLRVMAEAAIEDCYEDAAESVKVQITDESFDEIVKGSRTNLSRNAKCLRY :  82
BcucOBP19d3X2 : ----------------------------------------------MA----SADAKPPHYSALRVVAEAAIADCYEDSAQSVKVQITDEGFDEIVKGSRTNLTHNAKCIRY :  62
BcucOBP19d3X1 : -----------------MHTFKILP---------IFAVLSSIYTITMA----SADAKPPHYSALRVVAEAAIADCYEDSAQSVKVQITDEGFDEIVKGSRTNLTHNAKCIRY :  82
CcapOBP19d3   : -----------------MEIPKFFF---------IFAIIITGSKMTLV----VADSKPPHYSSLRLMAEDAIGDCYEDAARSVKVEITDEGFDELLKGSRDNLSRNAKCLRY :  82
RzepOBP19d3   : -------------MNFTVEVRLLFINLVRICGMRVLSVLTICLAISLLPVSMLADPPPPHYDSLRTMAEAAVEDCYEDADQNVKVEITDEGFDELLRGSRGNLLRNTKCLRY :  99
                                                                                            C              d                 aKCl

                      120       130       140       150       160       170       180       190
DmelOBP19dX1  : CVMKKLQIMDESGKLNKEHAIELVKVMSKHDAEKEDAPAEVVAKCEAIETPEDHCDAAFAYEECIYEQMKEHGLELEEH- : 150
DmelOBP19dX2  : CVMKKLQIMDESGKLNKEHAIELVKVMSKHDAEKEDAPAEVVAKCEAIETPEDHCDAAFAYEECIYEQMKEHGLELEEH- : 150
BdorOBP19d2   : CTMKKLGTMDENNKVVEAGAIAYIKQLSGGDAEFEKLSLETYNECKSTPESSNECEYAEAFRQCVLESAKSKGLKILPQV : 144
BlatOBP19d2   : CLMKKIGILDENNKVIEAAAIAYVKQVSGGDAEFEKLSMETYNECKSTPESSDECEYAEAFRQCVIESAKSKDIKILPR- : 143
BoleOBP19d2   : CCMQKLGILDENHKLVEAGAIEYIKQVAAGDAEYEKQSMEVLNECKNTPESSNECEYAEAFRVCVIESSKSKGIKILPQ- : 143
BminOBP19d2   : CTMKKFGIMDENNKLVEAAAIDYIKAVAAGDAEFEKLSTEVFNECKNTPASSDECEFAETFSSCIIENSKSKGIKILPQ- : 143
BcucOBP19d2   : CIMKQFGILDDNNKLVEAGAMAYIKSLAAGDAELEKLSTEVYNECKNTPANSNECEYAEAVRACTIENSKSKGIKILPQ- : 143
BcucOBP19d2X1 : CTMRLLGILDKNNKPFEAGAMAYIKSVTASNAELEKLLTEVYNECKYFPASKEGCEFSEAFRVCIIERSRAKGIHMLLH- : 143
BcucOBP19d2X2 : CTMRLLGILDKNNKPFEAGAMAYIKSVTASNAELEKLLTEVYNECKYFPASKEGCEFSEAFRVCIIERSRAKGIHMLLH- : 119
RzepOBP19d2   : CVLKNFGFLDADNKLVKDVGVDFLKSISGGDADYEKLGTEILDACKDTPAGGNNCETSEALRTCLIEKSQEKGFKLPF-- : 142
CcapOBP19d2   : CVLKKSGALGENNKINEAVMVEFIKAGAAGDASIEKIGMEGLQACLKVPQHPNACENAAALNACFTKNLRAKGFKGLPA- : 183
BminOBP19d1   : CIIKRFGVMNGDGKIDRDKAMEILTIIASGNGEQQALGVEVLEACADIDVNEDHCEAAEEYRTCMHAKAKENGFIMGRV- : 141
BoleOBP19d1   : CVIKRFGVMNGDGKIDRDKAMEVLTIITSGNEEQHALGVEVLEACADIDVNDDHCEAAEEYRTCMHAKAKENGFVMGRV- : 141
BdorOBP19d1   : CVMKRFGAMNGDGKFDREKAMEILAIIADGNDEQHALGVEVLDACADIDVNEDHCEAAEEYRTCMHAKAKEIGFVVGRV- : 141
BcucOBP19d1-1 : CVMKRFGLINGDGKMDRDKAIEILENIASGDNEQQALGVEIVEACEGIEVDEDHCEAAEEYRSCMHDKGKENGFKMGRV- : 141
BcucOBP19d1-2 : CVMKRFGLMNGDGKMDRDIAIEILENIASGDEAQQALGVEVVEACEGIEVDEDHCEAAEEYRSCMHDKGKENGFKMGRV- : 141
CcapOBP19d1   : CVMKRFGLMNDDGKMDKEKATDILKIIASGNEEQESLGLEVLETCVDIDVDEDHCEAAEDYRVCMHTKAAENGFTMARV- : 142
RzepOBP19d1   : CAMKKFGLMDDDGKLDKERAIEILKIIKSDDEEQQNLGVEIVDACADIDVNEDHCEAAEEYRTCMHAKAAENGFTLGRV- : 141
BdorOBP19d3   : CIMRKNGLLSMDNSIDEENILQIFGIIHP-QIKKDSLLDVLHKCARETDKQTDNCERAFVATGCILRELQADGVTDI--- : 157
BlatOBP19d3   : CIMRKNGLLSVDNSIDEENILQIFEIIHP-QIKKDSLLDVLHKCSRETDKQTDNCERAFVATSCILRELQADGVTDI--- : 157
BoleOBP19d3   : CIMRKNGLLNIDNSIDEENILQIFQIIHP-QVEKDSLLDVLQKCSRETNKQADNCERAFMASSCILRELQEDGVTDI--- : 157
BminOBP19d3   : CIMRKNGLLNEDNSIDRENILQIFQIIHP-QIEKDFPLDVIQKCSREMDKQADNCECAFVASSCILRELQVDGVTDI--- : 158
BcucOBP19d3X2 : CIMRKNGLLNNDNSIDKENILQIFQIIHP-QIEKDLLLNVLQKCSQEADKQANNCERAFVTSSCILQELSEDGVTDI--- : 138
BcucOBP19d3X1 : CIMRKNGLLNNDNSIDKENILQIFQIIHP-QIEKDLLLNVLQKCSQEADKQANNCERAFVTSSCILQELSEDGVTDI--- : 158
CcapOBP19d3   : CIMRKNGLLNELNSVDEEKIIQIFQIIHP-QIEREKLLSVIRKCSEETEKQTDNCERAFLAAKCILLELKEDGVTDI--- : 158
RzepOBP19d3   : CIMRKNGLINAGNSIDEDRVVEIFEIIHP-QIEKEKLIKVLQKCSDETEKQIDNCERAFAAATCVLGELKGQGVTDI--- : 175
                C m   g                         e   l               Ce a      C        g
```

Figure S4-5 The alignment of OBP19d

```
                     10        20        30        40        50        60        70        80        90       100       110
BdorOBP28a : -MAKLILFAALCILSAAVSNAAFNKEEAIKNFMTKAEECRGEVGAADSDIQNIVAKLPAASKEGKCLHSCLMKKYGAMDSNGKFVKSVADQHAQDFTDGDADKLKTAREII : 110
BlatOBP28a : -MAKFILFAALCILSAAVSKAAFNKEEAMKNFMTKAEECRGEVGAADSDIQDIIAKVPAASKEGKCLRSCLMKKYGAMDSNGKFVKSVADQHAQDFTDGDADKLKTAREII : 110
BoleOBP28a : -MAKFILFAAFCILSAAVSKAAFNKEEAIKTFMTRAEECRGEVGAADSDIQDIVAKVPAAGKEGKCLRSCLMKKYGAMDNNGKFVKSVADEHAQTYTDGDADKLKIAREII : 110
BcucOBP28a : -MAKFILIAALCILSAAVTKAAFNKEEAIKTFMTRAEECRGEVGAADSDIQDLIAKVPAAGKEGKCLRSCLMKKYGAMDGNGKFVKSVADEHAQTYTDGDADKLKIAREII : 110
BminOBP28a : -MAKFILIAALCILSAVVSKAAFNKEEVIKTFMTRAEECRGEVGAADSDVQDILSKLPAAGKEGKCLRNCLMKKYSAVDNNGKFVKSVAEEHAQIYTDGDADKMKIAHEII : 110
CcapOBP28a : -MAKFILIAALCILSAALTKAAFNKEEAIKTFMTRAEECRGEAGAADSDIQDLITKSPAAGKEGKCMRSCIMKKYGVMDSNGKFVKSVAMEHTKTYTDGDADKLKIANEII : 110
RzepOBP28a : -MAKLVLIGAFCILNAAVSKAAFNKEEAIKNFMTNVEECRGEVGAAASDIEDLIKKAPAASKEGKCLRSCMMKKYGVMDDGGKFVKSVALEHASTFTDGDAEKMKTANEIT : 110
DmelOBP28a : MQSTPIILVAIVLLGAALVR-AFDEKEALAKLMESAESCMPEVGATDADLQEMVKKQPASTYAGKCLRACVMKNIGILDANGKLDTEAGHEKAKQYTGNDPAKLKIALEIG : 110
              mak il aA ciLsAav kaAFnkeEaik fMt aEeCrgEvGAadsDiqd   K PAa keGKClrsC MKkyg mD nGKfvksva eha   TdgDadKlK A EIi

                   120       130       140
BdorOBP28a : DACADIAVPDDHCEATEVYGKCFMDQAKAHGIQKFDF : 147
BlatOBP28a : DACADIAVPDDHCEATEVYGKCFMEQAKAHGIQKFDF : 147
BoleOBP28a : DACADIAVPDDHCEASELYGKCFMDQAKARGIQKFDF : 147
BcucOBP28a : DACADIAVPADHCEATEVYGKCFMEQAKAHGIEKFEF : 147
BminOBP28a : DACAGIPVPDDPCEAAEVYGKCFLEQAKAHGIEKFDF : 147
CcapOBP28a : DACADIAVPDDHCEATELYGKCFMEQTKAHGIDKFEF : 147
RzepOBP28a : DACAGIAVPDDHCEAAEIYGKCFMEQAKAHGLDKFEF : 147
DmelOBP28a : DTCAAITVPDDHCEAAEAYGTCFRGEAKKHGLL---- : 143
             DaCA IaVPdDhCEA E YGkCFm qaKahGi kf f
```

Figure S4-6 The alignment of OBP28a

```
                      10        20        30        40        50        60        70        80        90       100       110
BdorOBP44a    : MKYIVAVLLAALVAMAAAEEYKIRNQDDLLKARKECMEAKKVPTEHIEKFKKFEFPDDEVTRCYIECIFNKFQLFSPTEGFKTQNLIAQLGQNKENKDAVKADIEKCADK : 110
BcucOBP44a    : MKYIVAVLLAALVAMAAAEEYKIRNQDDLLKARKECMEAKKVPTEHIEKFKKFEFPDDEVTRCYIECIFNKFQLFSPTEGFKTQNLIAQLGQNKENKDAVKADIEKCADK : 110
BoleOBP44a    : MKYIIAVLLAALVAMAAAEEYKIRNQDDLLKARKECMEAKKVPTEHIEKFKKFEFPDDEVTRCYIECIFNKFQLFSPTEGFKTQNLIAQLGQNKENKDAVKADIEKCADK : 110
BlatOBP44a    : MKYIVAVLLAALVAMAAAEEYKIRNQDDLLKARKECMEAKKVPTEHIEKFKKFEFPDDEVTRCYIECIFNKFQLFSPTEGFKTQNLIAQLGHNKENKDAVKADIEKCADK : 110
CcapOBP44a    : MKYIVAVLLA-LFALAAAEEYKLRNQDDLLKARKECMEAKKVPAEHIEKYKKFEFPDDEVTRCYIECIFNKFQLFSPTEGFKTQNLIAQLGHNKENKDAVKADIEKCADK : 109
RzepOBP44a    : MKYIAAVLLA-LFALAAAEEYKLRNQDDLLKARKECVEAKKVPVDHIEKYKKFEFPDDEVTRCYIECIFNKFQLFSPTEGFKTHNLIAQLGQSKENKDAVKADIEKCADK : 109
DmelOBP44aX2  : MKNAVAILLCALLGLASASDYKLRTAEDLQSARKECAASSKVTEALIAKYKTFDYPDDDITRNYIQCIFVKFDLFDEAKGFKVENLVAQLGQGKEDKAALKADIEKCADK : 110
DmelOBP44aX1  : MKNAVAILLCALLGLASASDYKLRTAEDLQSARKECAASSKVTEALIAKYKTFDYPDDDITRNYIQCIFVKFDLFDEAKGFKVENLVAQLGQGKEDKAALKADIEKCADK : 110
                MKyivAvLLaaL a AaAeeYK RnqdDLlkARKEC eakKVp  hIeK KkFefPDDevTRcYIeCIFnKFqLFspteGFKt NLiAQLGq KEnKdAvKADIEKCADK

                    120       130       140
BdorOBP44a    : NEQKSDSCTWAYRGFKCFISKNLPLVQESLKKN : 143
BcucOBP44a    : NEQKSDSCTWAYRGFKCFISKNLPLVQESLKKN : 143
BoleOBP44a    : NEQKSDSCTWAYRGFKCFISKNLPLVQESLKKN : 143
BlatOBP44a    : NEQKSDSCTWAYRGFKCFISKNLPLVQESLKKN : 143
CcapOBP44a    : NEQKSDSCTWAYRGFKCFISKNLPLVQESLKKN : 142
RzepOBP44a    : NEQKSDSCTWAYRGFKCFISKNLPLVQESLKKN : 142
DmelOBP44aX2  : NEQKSPANEWAFRGFKCFLGKNLPLVQAAVQKN : 143
DmelOBP44aX1  : NEQKSPANEWAFRGFKCFLGKNLPLVQAAVQKN : 143
                NEQKSdsctWAyRGFKCFisKNLPLVQeslkKN
```

Figure S4-7 The alignment of OBP44a

```
                      10        20        30        40        50        60        70        80        90       100       110
CcapOBP46aX2  : MFRTVIPIVIWLLLLQYQCHAEQ-KTTTDCSKLPKSI---------------------------------------CFVECLFNRSGICHQGKCSYQR--AIDYLDSEFA- :  68
CcapOBP46aX1  : MFRTVIPIVIWLLLLQYQCHAEQ-KTTTDCSKLPKSIAPQSCCRFPEP----------FQNPILSECYTLYNDIGQCFVECLFNRSGICHQGKCSYQR--AIDYLDSEFA- :  97
RzepOBP46a    : MIRAIIFFAIWVLAA--ICRAEQ-KTTTDCSKLPKSIAPQSCCRFPEP----------FQNPILDECYNLHNDIGQCFVECLFNRSGICRQGKCSYQR--AIDYLDREFA- :  95
BdorOBP46a    : MIQLFILLSTWLFTE--KCYAEQ-NNQTDCSRLPKSIAPHICCRFPEL----------FEGQILDECHRLHNEFGQCFVECLFGRADICRHGKCSYQR--ANKYLDNKFV- :  95
DmelOBP46a    : MCSQLFAFLLLLTAFVTGRSTPPALDEDCELNSVDTMHDFCCDLHDESPQFSDCQMEWHEKIPYETDEEEQTYMFCTAECSFNSTNFLGRDRRSLNLNEVKEHLESDLVN : 111
                M    i    wll      c aeq     tDCs lpksi     cc                       i  e          CfvEClFnr   ic  gkcSyqr   a  yLd   f

                    120       130       140       150       160       170       180       190
CcapOBP46aX2  : -LQQSAFKDIYKRAFKKCIAKA-NDVLGNMVKRFSRHGCHPLPEIIRFCIRNEMFTSCPNGYWNERVPGCSKKRDFIRNCIKDN---- : 150
CcapOBP46aX1  : -LQQSAFKDIYKRAFKKCIAKA-NDVLGNMVKRFSRHGCHPLPEIIRFCIRNEMFTSCPNGYWNERVPGCSKKRDFIRNCIKDN---- : 179
RzepOBP46a    : -LQQSAFKEIYKRAFKKCIAKA-NDVLGNMVKRFSRHGCHPLPEIIRFCVRNEMFTSCPNGYWNNRVAGCSKKRDFIRNCIKENL--- : 178
BdorOBP46a    : -LQQTIFKDIYKSAFKKCIEKG-NGLLTPLVERFRRNGCHPVPELIRFCVRNEMFISCPNEYWNEKLMRCTKKQQFIRHCTKEN---- : 177
DmelOBP46a    : DADIKLLYDTYVKCDKHALSLMPHKGVKQLSKRLSRLGCHPYPGLVLECVANEMILHCPTKRFRQTAQ-CEETRNHLKQCMQYLKYKS : 198
                 lqq   fkdiYk afKkci  k   n   l    vkRfsR GCHP  Pe  irfC rNEMf sCPn ywn     C kkr fir C k n
```

Figure S4-8 The alignment of OBP46a

```
                      10        20        30        40        50        60        70        80        90       100       110
BdorOBP47a    : ----MDGKWVVLAGVILASIGNLPPFSGVEARSASVKMSLDLSVSEDQ-HRITADMVRLCALETDLSMDELRRFSENDFANATKATQCFTHCLFEHMGLVSNGIFVERDIISFLG : 110
BlatOBP47a    : ----MDSKWIVLAGAILASIGNLPPFPAVEARSASVKMSLDLSVSEDQ-HRKTADMVRLCALETDLSMDDLRRFSENDFSNVTKATQCFTHCLFEHMGLVSNGIFVERDIISFLG : 110
BoleOBP47a    : MQSAINKALIVLTAVILATIGGLPSFPGVKARSASV-------------------------------DELRRFSEKDFSNATKATQCFTHSLCEHMGLVSNGVFVERYIISFLG :  83
BminOBP47a    : ----MDSKLIVLTATMLAAIVELTPFLSVEARSASVKMSLDLSVSEDQ-HRISADMVSLCALETDLSMGELRRFSENDFRNATKAIECFTHCLYEHMGLLNNGVFVERDIISLLG : 110
BcucOBP47a    : ----MDSKLIVFTALVLAAMVELPTFPGVEARSASIKMSFDMSVSEDQ-HRINPEMVRLCALETDLSMDELRKFNENDFSNATKNTQCFTHCLYEHMGVVRNGVFVERDLISLLG : 110
CcapOBP47a    : ----MNN-------------------PTLTARVASIKMSIDLSISEDQ-HRINSDMVHLCALETDLFMEELRKFSENDFNDTVKVTQCFTHCLYEHMGLVRDGVFVQRDVISLLG :  91
DmelOBP47aX1  : ----MNR--------VLVLLLVLKMFALSESRFAKININLGLTVADESPKTITEEMIRLCGDQTDISLRELNKLQREDFSDPSESVQCFTHCLYEQMGLMHDGVFVERDLFGLLS : 103
DmelOBP47aX2  : ----MNR--------VLVLLLVLKMFALSE-----ININLGLTVADESPKTITEEMIRLCGDQTDISLRELNKLQREDFSDPSESVQCFTHCLYEQMGLMHDGVFVERDLFGLLS :  98
                    m          l     l  f    ear as        l v        i    m   lc    td s   eLr  f  e DF      k   qCFTHcL EhMGl     GvFVeRd is Lg

                  120       130       140       150       160       170       180       190       200       210       220
BdorOBP47a    : DVTDPKRMLERECLGQFSDNKCERAFLIHQCYRA--GQRLMKPPNNQPEPVDQLIEERNREEETPAVDLPTTDMAMASAHRMPPDDERQLLIKNILAKRLPRKQNIKQELEEE : 221
BlatOBP47a    : DVTDPKRMLERECLGHFSDNKCETAFLIHQCYQS--GQRLMRPPYNQPEPVDQLNEEPNRKEETRAMDLSTTDMAMPSAHRMSPDAEHQLLIKNILAKRLPRKQNIKQGLEEE : 221
BoleOBP47a    : DVTDPKRMLERECLGQFSDNKCERALLIHQCYRA--DQRLMK------DNIYLF---------------------------------------------------------- : 129
BminOBP47a    : DVTDLKRMLERECLGQYSDNKCERAFLIHQCYRA--GQRVMMAPNRQPESIDQLNEERDGEEEKSVANLPKSDMAMPSVHRMATDSERQLLIKNILAKRLPKKQTINQGLVEE : 221
BcucOBP47a    : DVTDPKRMLERECLGQYSDNKCERAFLIHQCYRA--GQRMMMPSNHQLEPVDQINEEDNAEEE-STANLSKSDMAMTPAHRMAPNSERQLMIKKNLTKRLPKKQTINQGLEEE : 220
CcapOBP47a    : DTIDSKHIIEHECFNQCSENKCKRAFLIHQCYHGDGGKGIISSAQQFEAAEDKLNEDEKEDDSEAASDVSLPRTLIESTMPVKAGTERQIILKKLLAKRQPKIH--------- : 195
DmelOBP47aX1  : DVSNTDYWPERQCHAIRGNNKCETAYRIHQCQQQLKQQQQNLLATKEVEVTTTPAGSDETKP-------------------------------------------------- : 165
DmelOBP47aX2  : DVSNTDYWPERQCHAIRGNNKCETAYRIHQCQQQLKQQQQNLLATKEVEVTTTPAGSDETKP-------------------------------------------------- : 160
                Dv d k   EreC     s NKCe A lIHQCy      q          e
```

Figure S4-9 The alignment of OBP47a

```
                    10        20        30        40        50        60        70        80        90       100       110
BlatOBP47b : -----MQKIELFVFCAFLVYS---CVKAVEIDCRRPPQLVQPSLCCKNEERAAVMETCAQR-LGLSF-EKQKGATALEDVTCFAECIVKELQHLSAPEKINFEAVQKYLQTKS : 103
BoleOBP47b : -----MARLR-FLCCFSLLYTVK-YTDAVEIDCSRPPQLVQPSFCCKNDERAAVMETCAQR-LGISF-EKQNGATPLEDVTCFAECIVKELQHLSAPEEINFEAVQNYLQTKS : 104
BcucOBP47b : --MKIMLKFKLFVLFACLAYS---CAETVEIDCRRPPQLVQPNLCCKGGDSVPSLKTCAQR-LGVNL-EDQTGAIPLQTAACFAECIVKESQYITEPEKVNFEALQTYLQTKS : 106
CcapOBP47b : MKVRIMLQWKFCVFFLLLAVFGFGRSKAVEIDCQRPPRLVDPAQCCKDGGRDQIVEACAQR-MGLAS-QRGNGPPTVEAATCFAECILKQLQYMQEPEKINYEAMRKHLQMKF : 111
DmelOBP47b : -----MSPSQLLVIFASLALNTRLVFGQATIDCQRPPQLVDPALCCKDGGRDQVAEQCAQRILGTANGQKAGGPPSLDTAACLAECILTSSKYIDEPQKLNLANIRSDLSAKF : 108
                   M      v    L          veIDC RPPqLV P  CCK    r     e CAQR lG          G    l      CfAECI k  q     Pek N ea     Lq K

                 120       130       140       150       160       170       180       190       200
BlatOBP47b : GNDTTYVDTMLTAYRKCVSVAQQRMQVIKQHS---LGGGVFALRS-CSPFSGILLSCVHMEYFMNCPANRWTDNAECALAKQFVTQCAIGTV : 191
BoleOBP47b : GNDTTYVNTMMTAYRKCESVAQQRMQSIKQMS---FGGGALSQRR-CSLFSGILLSCVHMEYFMNCPSNRWTDNEECALAKQFVTQCAIGSI : 192
BcucOBP47b : DNDTTYVDTMMTAYKKCELAAQQRMQMFQQIP---YGGGAIAQRRICSPFYGILLSCVHMEYFMNCPAQRWTTNEECALAKQFVKQCTVGAS : 195
CcapOBP47b : SNDTIYVNTMLQAYQKCEPSVQQKMQILKQTP---LGGGAAALRRGCSPFSGMLLGCTYMEYFKNCPAHRWNENEQCALAKQFVTKCSFNA- : 199
DmelOBP47b : SNDTLYVETMTMAFSKCEPQSQRRLAMIMQQQQQVQQQKTQQQQPRCSPFSAIVLGCTYMEYFKNCPDHRWTPNAQCTLAKAYVTQCGLGA- : 199
              NDT YV TM   Ay KCe    Qqrmq    Q       ggg     r  CSpFsgilL C  MEYF NCP  RWt N  CaLAKqfVtqC  g
```

Figure S4-10 The alignment of OBP47b

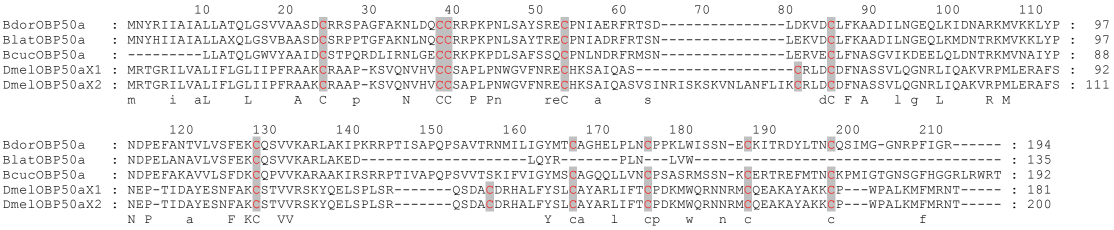

Figure S4-11 The alignment of OBP50a

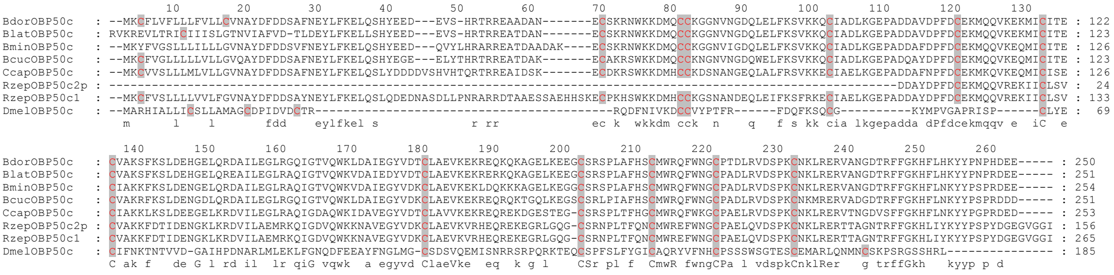

Figure S4-12 The alignment of OBP50c

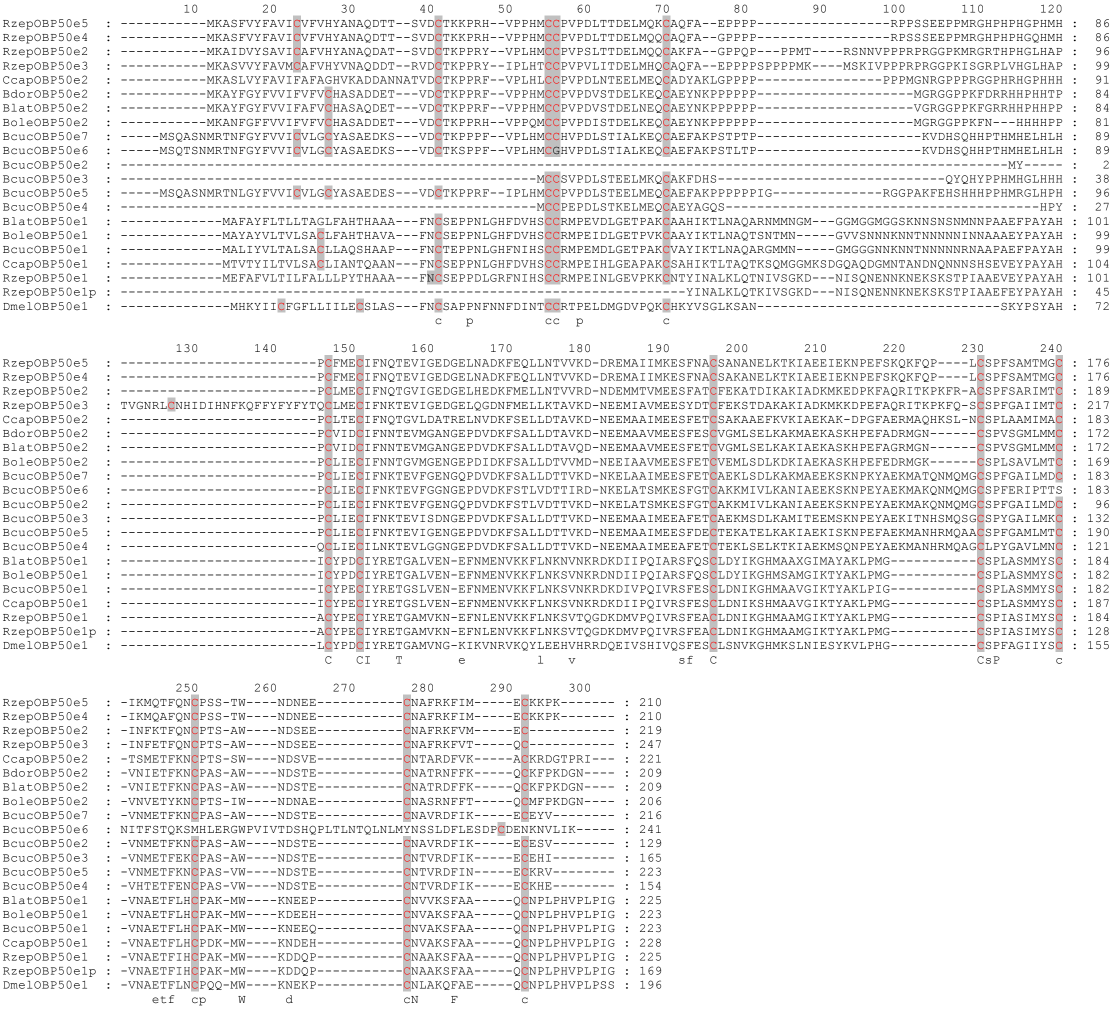

Figure S4-13 The alignment of OBP50e

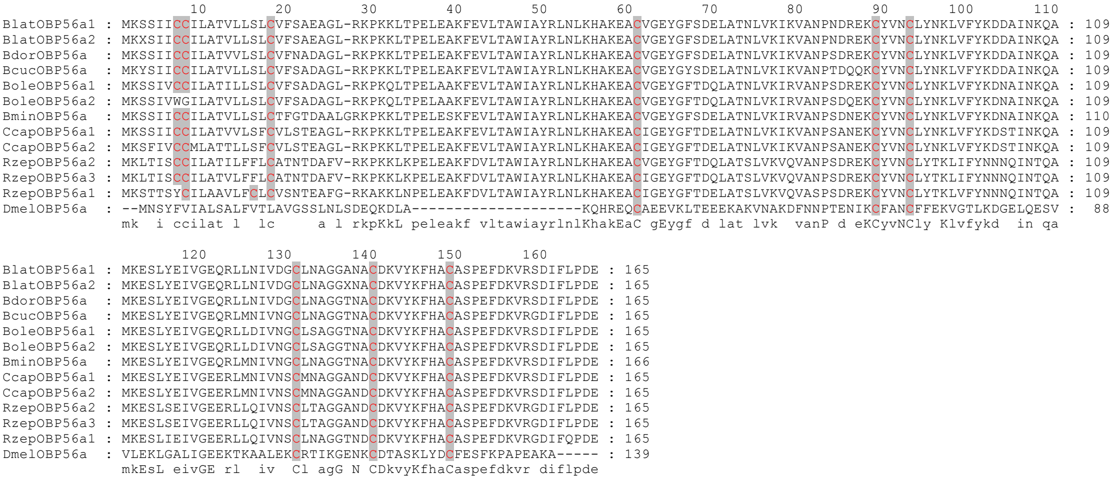

Figure S4-14 The alignment of OBP56a

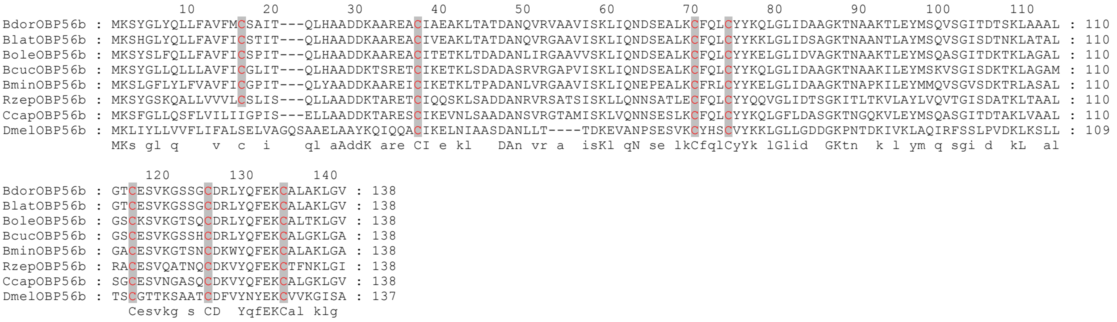

Figure S4-15 The alignment of OBP56b

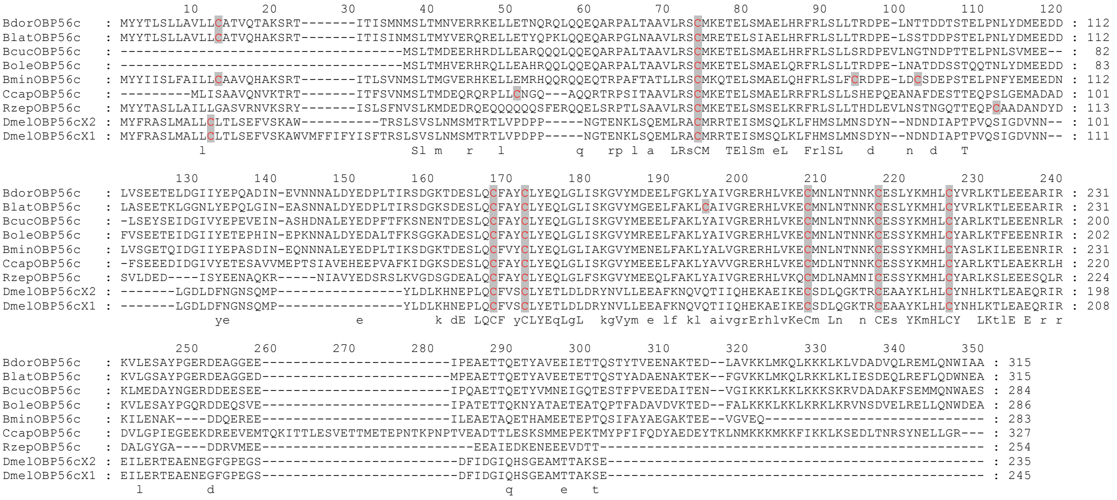

Figure S4-16 The alignment of OBP56c

```
                      10        20        30        40        50        60        70        80        90       100       110
BdorOBP56d2  : MKFFAVAVLLAFVAVAAAQEGVGKLTEEQKQKARALGTECLKETGASEEAIRALIKGDDSQVDGKVKCFSKCMQEKLGFVENGKVNEEKVQNFLGKLIGEENAKATQAKCND : 112
BlatOBP56d2  : MKFFAVAILLAVVAISAAQEGVGKLTEEQKQKAHAVGTDCLKETGASEEAIHALIKGDDSQVDSKVKCFTXCMLEKLGFIENSKVNEEKVQNFLGKLIGEEKAKATQDKCNG : 112
BdorOBP56d1  : MNFFAVAVLIVFVAVAAAQEG--MLTPEQIQKVHTLSNECLKETGASEDAIRALIKGDDSQVDGKVKCYAQCMLVKLGYVENGKVNEEKVQNILGKLIGEEKAKATQAKCNG : 110
BlatOBP56d1  : MKFFAVAVLLAFVAVAAAQEGAGMLTQEQIQKVHTLSTECLKETGASEEAIRALIKGDDSQVDGKVKCFTKCMLDKLGYVENGKVNEEKVQNILGKLIGMEKAKATQAKCNG : 112
BdorOBP56d3  : MKFFAVAVLLAFVAVAVAQEGVGKLTEEQKQKVHAAAAECFKETGASEDAVRALLKGDDSQVDGKVKCFAKCTLGKLDLLQNGKVNEEKVQNILGKLIGEEKAKAAQAKCNG : 112
BminOBP56d   : MKFFTIVAVFAFVAVAAAQEG-IKLSEEQKQKVHTLGAECLTETGASEAAVRAVGKGDLSQVDDKVKCFAKCLQGKLGYFENGQVNEAVVQSSLGKVVGEEKIKAIQAKCNG : 111
BcucOBP56d   : MKFFTVAVVLAFVAVAAAQDGGLKLSEEQKQKVHTLGAECLKETGASEEAVRAVGKGDFSQVDGNVKCFAKCLQGKLGYFVDGKVNEAAVQSSLGKLVGEEKIKAIQAKCNG : 112
CcapOBP56d   : MKFFTVAIVLAFVAAAAAQEL-IKLTDEQKQKVHALGAECIKETGAAEEAVRAAGKGDYSQVDGKVKCFAKCLQGKLGYVEDGKLNEAAIQASLGKIVGDEQIKAIQAKCNG : 111
BoleOBP56d   : MKFFAVVVVLAFVAVAAAQEAGLKLTEEQKQKVHTLGAECLKETGAAEEAVRAVVKGDNSQVDDKVKCFAKCMLGKLGYVENGKVNEEVVQNSLGKLIGDEKVKAIQAKCNG : 112
RzepOBP56d1  : MKLFAAAIVLAFVAVAVAQNA-PRLSLDQMLKVSALTSECAKETGTSMESVLPLRQGDFSQVDDKVKCFSKCFQERLGFLVNGVVNEEAVQKSLGPLAGEEKVKAIQAKCNG : 111
RzepOBP56d3  : MKLFAAAIVLAFVAVAVAQNA-PRLILDQMLKVSALTSECAKETGTSMESVLPLRQGDFSQVDDKVKCFSKCFQERLGFLVNGVVNEEAVQKSLGPLAGEEKVKAIQAKCNG : 111
RzepOBP56d2  : MKYFATVIVLTFVAVAVGQDG-LKLSAEQKEKVHLLVGECAKETGVSKEAILALRQGDFSQVDDKVKCFSKCYQERLGYLVNGVVNEEAVQKSLGPLAGEEQIKAVQAKCNG : 111
DmelOBP56dX1 : MKFLIVLSVILAISAAE-----LQLSDEQKAVAHANGALCAQQEGITKDQAIALRNGNFDDSDPKVKCFANCFLEKIGFLINGEVQPDVVLAKLGPLAGEDAVKAVQAKCDA : 107
DmelOBP56dX2 : MKFLIVLSVILAISAAE-----LQLSDEQKAVAHANGALCAQQEGITKDQAIALRNGNFDDSDPKVKCFANCFLEKIGFLINGEVQPDVVLAKLGPLAGEDAVKAVQAKCDA : 107
               Mkff       fva a aq     L  eQ  k h     eC ketG       a    Gd sqvD kVKCf  C    klg   ng vne   vq  LG l Gee   KA QaKCng

                   120       130
BdorOBP56d2  : LKGTDECDTAFQIRQCYAAGHEGLDF : 138
BlatOBP56d2  : LKGTDECDFAYQIRQCYSSGYSGFAF : 138
BdorOBP56d1  : LKGTDECDTAYQIRQCYSAGYNGFAF : 136
BlatOBP56d1  : LKGADECDTAYQIRKCYSAGYNGFVF : 138
BdorOBP56d3  : LKGTDECDTAYQIRQCYAAGHESFVF : 138
BminOBP56d   : LKGTDDCDTAFLLHKCYVAENASTLV : 137
BcucOBP56d   : LKGTDDCDTAFQLHKCYATENASILV : 138
CcapOBP56d   : VKGADDCDTAFELHKCYLAENANIQI : 137
BoleOBP56d   : LKGTDDCDTAFQLRQCYVAEHGAIII : 138
RzepOBP56d1  : VKGADDCDTAFEWYKCYYEEKG--LA : 135
RzepOBP56d3  : VKGADDCDTAFEWYKCYYEEKG--LA : 135
RzepOBP56d2  : VKGANDCETAFELYKCYYGEKATVLA : 137
DmelOBP56dX1 : TKGADKCDTAYQLFECYYKNRAHI-- : 131
DmelOBP56dX2 : TKGADKCDTAYQLFECYYKNRAHI-- : 131
                KG d CdtA     CY
```

Figure S4-17 The alignment of OBP56d

```
                      10        20        30        40        50        60        70        80        90       100       110
BcucOBP56e   : MKSNILLAILIALSAVVAAHKS--HKPSQAHQFYEECLKESGASPALLDPLKKGDFNAVGDKAKCFLKCLENKKGILENGAPNEADI-RKVRK--VGNEEPKNILAKCNGLMGAN : 110
BoleOBP56e   : MKLNIFVATLISLSAVVAAHES--NKPTQAHQYYEHCLKESGASAAQLDALKRGDFNAIDDKAKCFLKCLENKKELLKNGVPNEAGK-GKLPMPAIGNSAPRNVLAKCNGLQGAN : 112
BdorOBP56e   : MKSYNFASRFNFTQRLSRLYELKPNKSTQVHQFYEDCLKESGASAAQLDALKKGDFNAVDDKAKCFLKCLQNKKGILENGVPNEAAI-HKVMTPAVGNSPPKNTLAKCNGLKGAN : 114
BlatOBP56e   : MKSFILVAALIALSTVA-AHELKSNKSTQVHQFYDDCLKQSGASAAQLDALKKGDFNAVDDKVKCFLKCVQNKKGILANGIPNEIAI-HKVLKPAVGNSAPKDILAKCNGLNGAN : 113
CcapOBP56e   : MNSNIFLITFLVLFALAAANELKSHKPARNHKIYEDCMKESGASVAQLEALKKGDFNSIDNKAKCFLKCLEDNKGILTNGMPNEAGIRKKVHAPPAGNGVSKDLLAKCNGLKGAD : 115
DmelOBP56eX1 : -MKVFFVFAALAALSLASAVGLTDSQKAEAKQRAKACVKQEGITKEQAIALRSGNFADSDPKVKCFANCFLEQTGLVANGQIKPDVVLAKLGP-IAGEANVKEVQAKCDSTKGAD : 113
DmelOBP56eX2 : -MKVFFVFAALAALSLASA-GLTDSQKAEAKQRAKACVKQEGITKEQAIALRSGNFADSDPKVKCFANCFLEQTGLVANGQIKPDVVLAKLGP-IAGEANVKEVQAKCDSTKGAD : 112
                                 a            q    C K  G    q  aL  G F   d K KCF   C      g   NG          K       G     k   AKC    GA

                 120       130
BcucOBP56e   : ECDTAFLLYKCYWQEHAALI : 130
BoleOBP56e   : ECDTAFQLYKCYLQEHVILI : 132
BdorOBP56e   : ECDTAFQIYKCYRQEHVGLI : 134
BlatOBP56e   : ECDTAFQIYKCYWQEHVILI : 133
CcapOBP56e   : ECDTAYQIYKCFMQEKVPLI : 135
DmelOBP56eX1 : KCDTSYLLYKCYYENHAQF- : 132
DmelOBP56eX2 : KCDTSYLLYKCYYENHAQF- : 131
                CDT    YKCy   h
```

Figure S4-18 The alignment of OBP56e

```
                      10        20        30        40        50        60        70        80        90       100
BdorOBP56g   : MKSFVTIALLV--VGSAVVLCNPH-DPEMRGYIEDCNKEHNVSPKDFHDFIEG--KLTTVPENMKCSSQCIMVKQGIMDESGNFKPDAAKAKMK----EDKLV :  94
BlatOBP56g   : MKSFVTIALLV--VGSAVVLCNPN-DPEMRGYIEDCNKEHNVSLKDFHDFMEG--KLTTVPDNLKCSSQCIMVKQGIMDESGNFKPDVAKAKVK----DDKLV :  94
BcucOBP56g   : MKSFITIALLV--VYSVVVLCHPN-DPEMRKFIEDCNKEHNVSPKDFHDFMEG--KLTTVPESLKCSSHCIMVKQGVMDESGNFKADVAKAKIT----EEKFA :  94
BminOBP56g   : MKSFITIALFV--VSCAVVLCNAN-DPEMRKQIEECNKEHNVTPKDYHDFMEG--KLTTIPENLKCSSHCVMMKQGIMDDSGKFKAEVAKAKMN----DDKFS :  94
RzepOBP56g2  : MKSFITIAFVL--VSSVVVMCEPP-NPQIRQYIDECNKEHNVSPKDFHEFNEG--KLSSPSEDLKCAIHCFMVKQGSMDEAGTFKPDVAKTGMPN---DDKFA :  95
RzepOBP56g1  : MKSFITIAFVL--VSSVVVMCEPP-NPQIRQYIDECNKEHNVSPKDFQEFNEG--KLSSPSEDLKCSMHCFLIKQGSMDEAGTFKPDVAKTSMPN---DDKFA :  95
CcapOBP56g1  : --------------------CHPPPDPEMRKYIDECTKDHNVTPKEFHEFMEG--KAASPSENLKCSSHCVMLKQGIIDEAGNFKPDVAKSKMP----DDKLA :  77
CcapOBP56g2  : MKSYFALTLFV--ISCAVVMCHPPPDPEMRKYIDECTKDHNVTPKEFHEFMEG--KAASPSENLKCSSHCVMLKQGIIDEAGNFKPDVAKSKMP----DDKLA :  95
DmelOBP56gX1 : MRATFALTLLLGCLSGILAQ-ANIDSSVSKELVTDCLKENGVTPQDLADLQSGKVKAEDAKDNVKCSSQCILVKSGFMDSTGKLLTDKIKSYYANSNFKDVIE : 102
DmelOBP56gX2 : MRATFALTLLLGCLSGILAQQANIDSSVSKELVTDCLKENGVTPQDLADLQSGKVKAEDAKDNVKCSSQCILVKSGFMDSTGKLLTDKIKSYYANSNFKDVIE : 103
               m                   c     p  r  i  C KehnV pkd   f eG  K         KCss C    KqG mD  G fk d aK          dk

                   110       120       130
BdorOBP56g   : AAVDECKDLSGSTPCDTAFKITSCMLSKKEFLKRWL : 130
BlatOBP56g   : AAVDGCKDLSGSTPCDTAFKITACMLSKK------- : 123
BcucOBP56g   : AAIDECKDLTGSTPCDTAMKITECMITHK------- : 123
BminOBP56g   : ATVDECKDLSGSTPCDTAMKITECLLSHK------- : 123
RzepOBP56g2  : AAVDACKDKTGSSACETAFRITQCVISHK------- : 124
RzepOBP56g1  : AAVDACKDKTGSSACETAFEIAQCIMSHK------- : 124
CcapOBP56g1  : AAIDDCKDLSGSSACDTAFKITECVLSHK------- : 106
CcapOBP56g2  : AAIDDCKDLSGSSACDTAFKITECVLSHK------- : 124
DmelOBP56gX1 : KDLDRCSAVKGANACDTAFKILSCFQAAN------- : 131
DmelOBP56gX2 : KDLDRCSAVKGANACDTAFKILSCFQAAN------- : 132
               a   D Ckd   Gs   CdTAfkI  C     k
```

Figure S4-19 The alignment of OBP56g

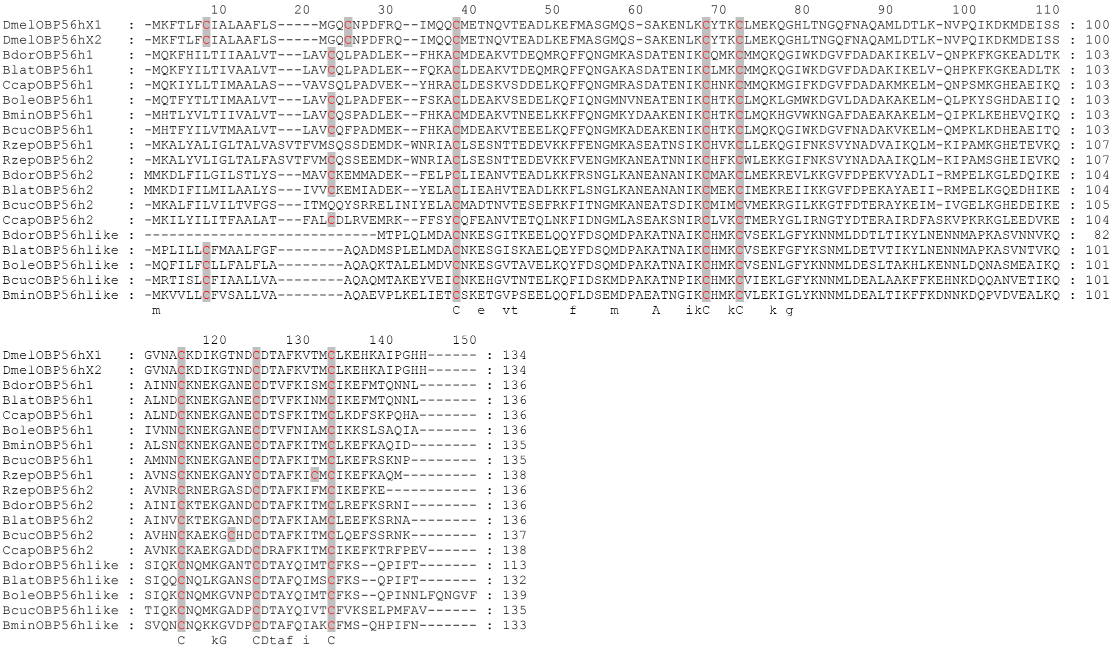

Figure S4-20 The alignment of OBP56h

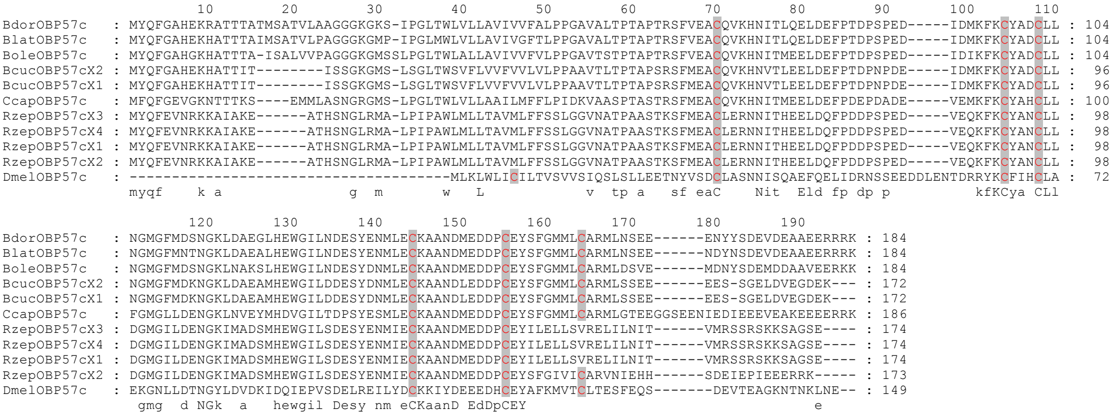

Figure S4-21 The alignment of OBP57c

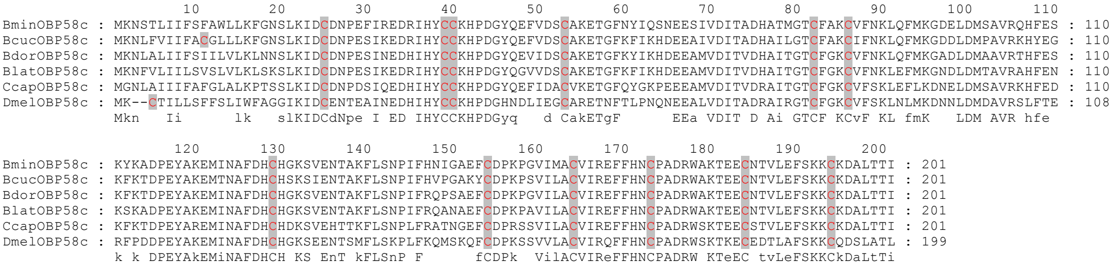

Figure S4-22 The alignment of OBP58c

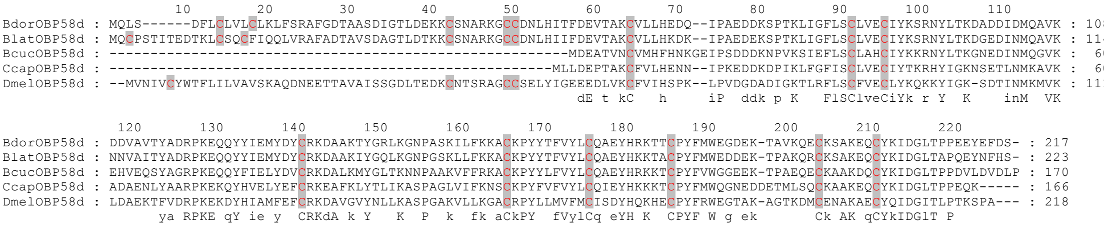

Figure S4-23 The alignment of OBP58d

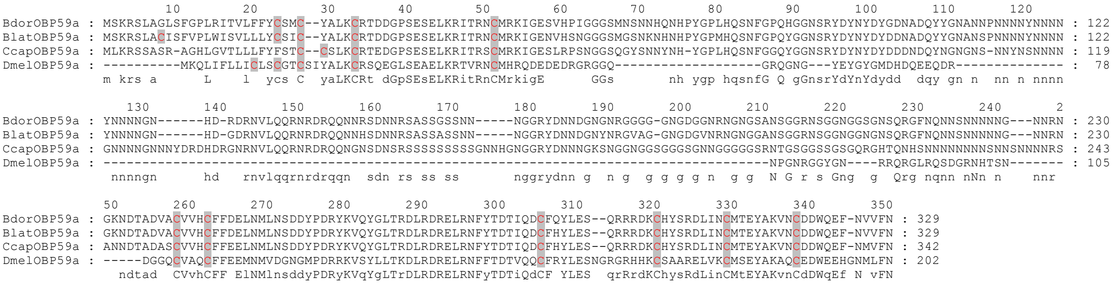

Figure S4-24 The alignment of OBP59a

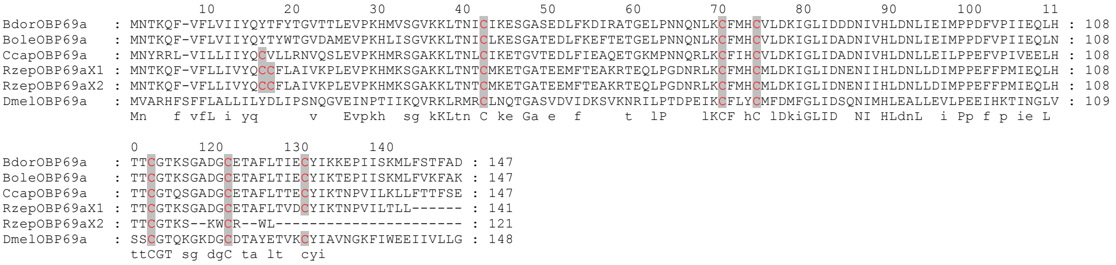

Figure S4-25 The alignment of OBP69a

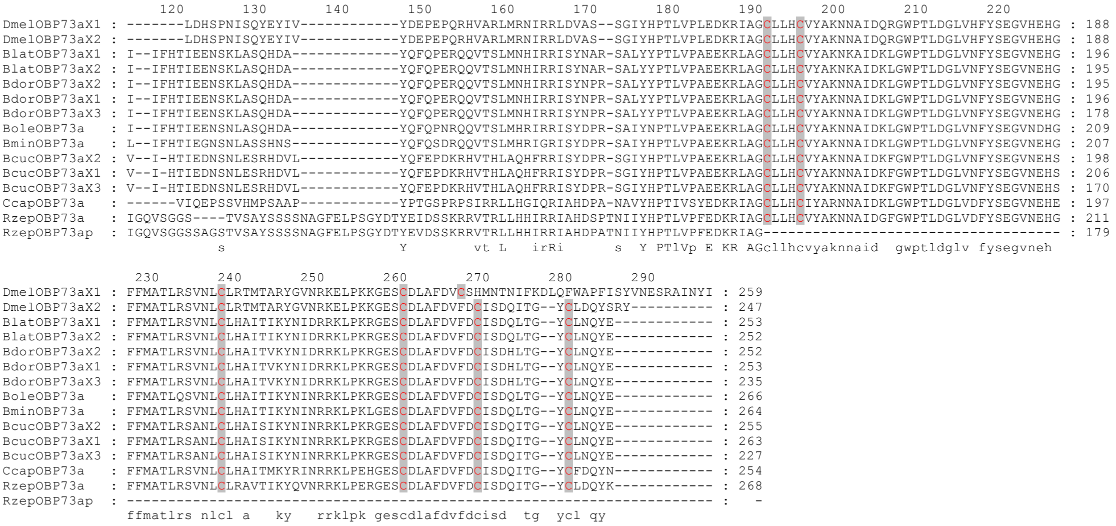

Figure S4-26 The alignment of OBP73a

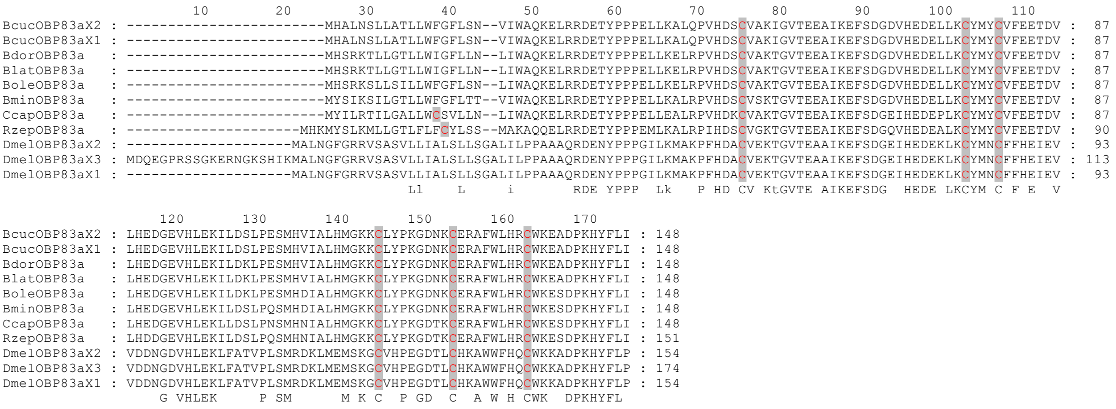

Figure S4-27 The alignment of OBP83a

```
                        10        20        30        40        50        60        70        80        90       100       110
BdorOBP83bX2 : MVLTGTRRGQAFHAFLIVALS-SSLTLMH-VQAQEPRRDDKWPPPAVLKMAKIFHDICVEKTGVTEEAIKEFSDGQIHEDEALKCYMNCLFHEIDVVDDNGDVHLETLFNTV : 110
BdorOBP83bX3 : MVLTGTRRGQAFHAFLIVALS-SSLTLMH-VQAQEPRRDDKWPPPAVLKMAKIFHDICVEKTGVTEEAIKEFSDGQIHEDEALKCYMNCLFHEIDVVDDNGDVHLETLFNTV : 110
BdorOBP83bX1 : MVLTGTRRGQAFHAFLIVALS-SSLTLMH-VQAQEPRRDDKWPPPAVLKMAKIFHDICVEKTGVTEEAIKEFSDGQIHEDEALKCYMNCLFHEIDVVDDNGDVHLETLFNTV : 110
BlatOBP83bX1 : MVVTGIRRGQAFHAFLIVALS-SSLILMH-VQAQEPRRDDKWPPPAVLKMAKIFHDICVEKTGVTEEAIKEFSDGQIHEDEALKCYMNCLFHEIDVVDDNGDVHLETLFNTV : 110
BlatOBP83bX2 : MVVTGIRRGQAFHAFLIVALS-SSLILMH-VQAQEPRRDDKWPPPAVLKMAKIFHDICVEKTGVTEEAIKEFSDGQIHEDEALKCYMNCLFHEIDVVDDNGDVHLETLFNTV : 110
BoleOBP83b   : MALIGIWRCQAFHAFLIVALS-SSLTLMH-VQAQEPRRDDKVRNIKHLKNISTFX----------SEAIKEFSDGQIHEDEALKCYMNCLFHEIDVVDDNGDVHLETLFNTV : 100
BcucOBP83bX2 : MVLNGILRGHPLSAFLIVALA-SSLTLVH-VRAQEPRRDDKWPPPAVLKMAKIFHDICVEKTGVSEEAIKEFSDGQIHEDEALKCYMNCLFHEIDVVDDNGDVHLETLFNTV : 110
BcucOBP83bX1 : MVLNGILRGHPLSAFLIVALA-SSLTLVH-VRAQEPRRDDKWPPPAVLKMAKIFHDICVEKTGVSEEAIKEFSDGQIHEDEALKCYMNCLFHEIDVVDDNGDVHLETLFNTV : 110
BminOBP83b   : MVLTGIWRCQAFNAFLIVALS-SSLTLMH-VQAQEPRRDDKWPPPAVLKMAKIFHDICVEKTGVTEEAIKEFSDGQIHEDEALKCYMNCLFHEIDVVDDNGDVHLETLYNTV : 110
CcapOBP83b   : MALNGSRRSQAFNAFLIVALALSSTLLMHVVQAQEPRRDDKWPPPAVLKMAKIFHDICVEKTGVSEEAIKEFSDGQIHDDEALKCYMNCLFHEIDVVDDNGDVHLETLYNTV : 112
RzepOBP83bp  : -----------------------------------------WPPPALLKMAKIFHDICVEKTGVTEEAIKEFSDGQIHEDEALKCYMNCLFHEIDVVDDNGDVHLETLYNTV :  71
RzepOBP83b   : MILNGILREQAFSAFLMVVLLSLSLRENY-VQAQEPRRDNKWPPPAVLKMAKIFHDTCVEKTGVTEEAIKEFSDGQIHEDEALKCYMNCLFHEIDVVDDNGDVHLETLYNTV : 111
DmelOBP83b   : ----------------MVKYP-LILLLIGCAAAQEPRRDGEWPPPAILKLGKHFHDICAPKTGVTDEAIKEFSDGQIHEDEALKCYMNCLFHEFEVVDDNGDVHMEKVLNAI :  95
               m   g  r     aflival  ssl l h v aqeprrddkwpppavLKmakiFhdicvektgv eEAIKEFSDGQIHeDEALKCYMNCLFHEidVVDDNGDVHlEtl Ntv

                      120       130       140       150       160       170
BdorOBP83bX2 : PG-TVRNQLINMAKECEHPEGDTLCHKAWWFHQCWKKADPVHYFLP--------------------- : 155
BdorOBP83bX3 : PG-TVRNQLINMAKECEHPEGDTLCHKAWWFHQCWKKADPVHYFLP--------------------- : 155
BdorOBP83bX1 : PG-TVRNQLINMAKECEHPEGDTLCHKAWWFHQCWKKADPVHYFLP--------------------- : 155
BlatOBP83bX1 : PG-TVRNQLINMAKECEHPEGDTLCHKAWWFHQCWKKADPVHYFLP--------------------- : 155
BlatOBP83bX2 : PG-TVRNQLINMAKECEHPEGDTLCHKAWWFHQCWKKADPVHYFLP--------------------- : 155
BoleOBP83b   : PG-TVRNQLINMAKDCEHPEGDTLCHKAWWFHQCWKKADPVHYFLP--------------------- : 145
BcucOBP83bX2 : PG-TVRNQLINMAKECEHPEGDTLCHKAWWFHQCWKKADPVHYFLP--------------------- : 155
BcucOBP83bX1 : PG-TVRNQLINMAKECEHPEGDTLCHKAWWFHQCWKKADPVHYFLP--------------------- : 155
BminOBP83b   : PG-TVRDKLINMARGCEHPEGDTLCHKAWWFHQCWKKADPVIAEVRGGRPATRTPLSSPAALVTPAF : 176
CcapOBP83b   : PG-TVRDKLINMAKGCEHPEGDTLCHKAWWFHQCWKKADPVHYFLP--------------------- : 157
RzepOBP83bp  : PG-TVRDKLINMAKDCVHPQGDTLCHKAWWFHQCWKKADPVHYFLP--------------------- : 116
RzepOBP83b   : PG-TVRDKLINMAKDCVHPQGDTLCHKAWWFHQCWKKADPVHYFLP--------------------- : 156
DmelOBP83b   : PGEKLRNIMMEASKGCIHPEGDTLCHKAWWFHQCWKKADPVHYFLV--------------------- : 141
               PG tvR  linmak CeHPeGDTLCHKAWWFHQCWKKADPVhyflp
```

Figure S4-28 The alignment of OBP83b

```
                       10        20        30        40        50        60        70        80        90       100       110       120
BdorOBP83cd : MELILIFLFAATIISP-AVAVVQPNAEEIRTLSDCLQSYGGITEENSKRLARFKDRSETYEEIPCFTKCYIKNMFNMFDESVGFNDEQVIKQFGQPLHKACKHRMEP----AADSCQQAYNGFHCL : 121
BlatOBP83cd : MESISIFLFAAAIISP-AVAVIQPNXVEIRILSDCLQSYGGITEENSKRLVRFKDWSETYEEIPCFTKCYIKNMFNMFDESVGFNDEQVIKQFGQPLHKACKHRMEP----AADSCQQAYNGFHCL : 121
BoleOBP83cd : MELILIFLFAAAIITP-SVAVVEPNAQEVRIISECLRSYGGLTEENAQRLVRFKGWSETYEEIPCFTKCYIKNMFDMFDESVGFKDEQVIKQFGQPLYKACKHRMEP----SANSCQQAYNGFHCL : 121
BminOBP83cd : MKLILIFLFAAAIISP-SVAVVQPNAEEVRIISECLQSYGGLTEDNSQRLVRFKDWSEKYEEIPCFTKCYIKNMFDMFDESVGFKDEQVIKQFGQPLYIACEHRMKP----STDNCQQAYNGFHCL : 121
RzepOBP83cd : MELFFISLLAIVIIAP-ITAVVQPNAEESRIINECLKSFGGLTEENARRLVRFKEWSEKYEEIPCFSKCYIKNMFDIFDESSGFKEEQVVRQFGQPLYNACQHRMVP----LADACEQAYHGFHCL : 121
CcapOBP83cd : MEYICIFLLAGALLATLAAAVVQPNVQEGLIIADCLKNYGGLTEDKAQRLVRFKDWSDHYEEIPCFTKCYIQNMFEMFDESEGFKEEQVIKQFGQPLYKACKHRMVP----AADTCQQAYNGFHCI : 122
DmelOBP83cd : MQMKSGILIALCLCLS-LNEGLALLEHEGETINRCIQNYGGLTAENAERLERFKEWSDSYEEIPCFTRCYLSEMFDFYNNLTGFNKDGIVGVFGRPVYEACRKKLELPFESGESSCKHAYEGFHCI : 125
              M    i L A          av  pn  E       Cl  yGG Te n  RL RFK wS  YEEIPCFtkCYi nMF  fdes GF  eqv  qFGqPl  AC hrm p        C qAY GFHC

                    130       140       150       160       170       180       190       200       210       220       230       240
BdorOBP83cd : VNLEDDPFVIIESMKNVSTEAKTAMKDCLHRFDQYEWERVKDYSKNPVREPIPCFTKCFIDRLQLYSQQTRQWNIPALTAKLGVPAAGANIQHCLKQRRNRNACVWMYQEFTCFVLAHD : 240
BlatOBP83cd : VNLEDDPFVIIESMKNVSTEAKTAMKDCLHRFDQYEWERVKDYSKNPVREPIPCFTKCFIEXLQLYSQQTRQWNIPALTAKLGVPAAGANIQHCLKQRRNRNACVWMYQEFTCFVLAHD : 240
BoleOBP83cd : VNLEDDPFVLIESMKNVSIEAKTAMKDCLHRFDQYEWERVKDYSKNPVREPIPCFTKCFIDRLQLYGQQSRQWNIPALTAKLGVPATGANIQHCLKQRRKRNACVWMYQEFTCFVLAHD : 240
BminOBP83cd : VNLEDDPFVIIESMKNVSTEAKTAMKDCLHRFDQYEWERIKDYAKNPVREPIPCFTKCFIDRLQLYSQQTRQWNIPALTAKLGVPAAGANIQHCLKQRRNRNACVNSAATTKAANQDVE : 240
RzepOBP83cd : VNLEHDPFVLIESMQNISTEAKTVMKECLHRFDQYEWEHLKDYSKNPVREPIDCFTKCFIERLQVYKASTHRWDIQALRTKLGVPAAEANIQHCLRRRHRGNACVWMYQDFICFALAH- : 239
CcapOBP83cd : VSLEDDPFVLIESMKNVSTEAKTAMKDCLHRYDRYEWEHMKDYAANPVREPIPCFTKCFVEHLQVFNQKTRQWNIPLLRAKLGVPAVGADIKHCLERRRNRNVCGWMYQDFTCFGLPV- : 240
DmelOBP83cd : TNMESHPFTVIDNMPNISPSAKDAMKDCLQDVHQDEWKSFDAFAYYPVNEPIPCFTRCFVDKLHIFEEKTRLWKLEAMKQNLGIPAKGARIRTCHRHR-GRDRCATYYKQFTCYAMAV- : 242
              vnlE dPFv IesM N S eAKtaMKdCLhr dqyEWe  kdy  nPVrEPIpCFTkCF   Lq      tr W i al  kLGvPA gA I hCl  R  rn C   y  f c
```

Figure S4-29 The alignment of OBP83cd

```
                       10        20        30        40        50        60        70        80        90       100       110
BcucOBP83ef : MIFRVASQAVLLIAAAYCIQMVSSAATTKVEERDVESDAEILRKCLHEVGSKD-VVGELQKVARYSKWTSEEIPCFTRCLASMKRWFDADESKWNKQQIADDLGADMFNYC : 110
RzepOBP83ef : --------------------------------------------------------------------------------------------------------------- :   -
BdorOBP83ef : MNFREASQVVLLIAVAYCIQMVSSAATTKAAEQDVDSDTEILRKCLREVGSKD-LVGELQKVARYSKWTSEEVPCFTRCLASMKHWFDADESKWNKQQIADDLGADMYNYC : 110
BlatOBP83ef : MNFHEASKVVLLIAVAYCIQLVSSAVTTKAAEQDVDSDTEILRKCLREVGSKD-LVGELQKVARYSKWTSEEVPCFTRCLASXKHWFDXDESKWNKQQIADDLGADMYNYC : 110
BoleOBP83ef : MNFREASQVILLIA--YCIQMVSSAATTKAAERDVDSDTEILRKCLREVGSKD-LVGELQKVARYSKWTLEEVPCFTRCLASMKHWFDANESKWNKQQIADDLGADMFNYC : 108
DmelOBP83ef : ---MSSPRAVLVSLFLICSQALA----------DLSGDAQTLEKCLRQLSSPESIAGDLRKLERYSSWTREEVPCLMRCLAREKGWFDVEENKWRLKQLTEDLGADVYNYC :  98
                     l       c q              d   d   l kcl     s      g l k   rys wt ee pc   rcla   k wfd   e kw    q    dlgad   nyc

                     120       130       140       150       160       170       180       190       200       210       220
BcucOBP83ef : RYELDRYNEDSCEFAYTGLRCLKQAELYTLETYKNILSCATELNVTMKELQKYAAFPTKEVVPCLFQCLAEKMDFYTPTYEWNFDKWVKAFGPTRQDPTAS----NVCKVS : 217
RzepOBP83ef : -------------------------------------------MEELQKYAAFPSKEVVPCLFQCLAKKMNFYTPTYEWNFDNWVKAFGPMRQNRKAS----DVCKVS :  61
BdorOBP83ef : RYELDRYNEDSCEFAYTGLRCLKQAELYTLETYKNIVSCASELNVTMKELQKYAAFPTKEVVPCLFQCLAEKMNFYTPTYEWNLDNWVQAFGPMRQDRTAS----NVCKVS : 217
BlatOBP83ef : RYELDRYNEDSCEFAYTGLRCLKQAELYTLETYKNIVSCASKLNVTMKELQKYAAFPTKEVVPCLFQCLAEKMNFYTPTYEWNFDNWVQAFGPMRQDRTAS----NVCKVS : 217
BoleOBP83ef : RYELDRYNEDSCEFAYTGLRCLKQAELYTLETYKNIVSCASELNVTMKELQKYAAFPTKEVVPCLFQCLAEKLNFYTPSYEWNFDNWVKAFGPMRQDRTAS----NVCKVS : 215
DmelOBP83ef : RFELRRMGSDGCSFAYRGLRCLKQAEMHAGTSLSTLLQCSRQLNATNVELLQYSKLKSKEPIPCLFQCFADAMGFYDPDGNWRLENWKQAFGPSGNEDQSSGADYSGCRLS : 209
              r el r   d c fay glrclkqae             c   ln tm ELqkYaafp KEvvPCLFQClA km FYtP yeWn dnWv AFGP rq    aS     vCkvS

                  230       240       250       260       270       280
BcucOBP83ef : SEQ-INKRDKCEWMYEEYNCLERLNYNTDGSYPMETTTLSAVITSKKPDAVEAAVKEAS----- : 275
RzepOBP83ef : AEQ-MQNRDKCEWMYEEYNCLERLNYNTDGSYPLETTTLSAVIASKKTKEVQVQQKTEEADANS : 124
BdorOBP83ef : AEQ-MKTRDKCEWMYEEYNCLERLNYNTDGSYPLESTTLSSVIARKTAASVEDKTKAS------ : 274
BlatOBP83ef : XEQ-MKTRDKCEWMYEEYNCLERLNYNTDGSYPLESTTLSAVITRNTAAAVEDLAKTS------ : 274
BoleOBP83ef : AEQ-TKMRDKCEWMYEEYNCLERLNYNTDGSYPLESTTLSAVITSKTTETVEDKTKAS------ : 272
DmelOBP83ef : GTQREVALSKCSWMYHEYKCWERVNGN---------------------KLVEDNEEQ------- : 245
               eQ    rdKCeWMYeEYnClERlNyNtdgsyp e ttls vi       Ve   k
```

Figure S4-30 The alignment of OBP83ef

```
                      10        20        30        40        50        60        70        80        90       100       110
BdorOBP83g : MNTQ-LILLLACVA--LVAG---KFQIRTAQDALDAHEACHEEYRVPEDIYQKFLNYEFPAHKRTNCYVKCFVERMGLFTEEKGFDEKAIIAQFTAKSSKNLAKVSHGLEKCL : 107
BlatOBP83g : MNTQ-LILLLACVA--LVAG---KFQIRTAQDALDAHEACHEEYRVPEDIYQKFLNYEFPAHKRTNCYVKCFVERMGLFTEEKGFDEKAIIAQFTAKSSKNLAKVSHGLEKCL : 107
BoleOBP83g : MKTQ-LILLLAFVA--LVAG---KFQIRTAQDALDAHEACHEEYRVPEDIYQKFLNYEFPAHKRTNCYVKCFVERMGLFTEEKGFDQKAIIAQFTAKSSKNLAKISHGLEKCL : 107
BminOBP83g : MQTQ-LILLMACVA--LVAG---KFHVRTAQDALDAHEACHEEYRVPEDIYQKFLNYEFAPHKRTNCYVKCFVERMGLFTEEKGFDEKAIIAQFTAKSSKNLAKISHGLEKCL : 107
BcucOBP83g : MKTQ-LILLLACVA--LVAG---KFQIRTAQDALAAHEACHDEFRIPEDIYEKYLNYEFPPHKRTNCYVKCFVERMGLFTEEKGFDEKAIIAQFTAKSSKNLAKVSHGLEKCI : 107
CcapOBP83g : MKIQ-LILLLACVA--LVAG---KFELRTAQDALAAHEACRDEFRIPDDIYEKYLNYEFPPHKRTNCYVKCFVERMGLFTEEKGFDEKAIIAQFTAKNSKNLAKVSHGLEKCL : 107
DmelOBP83g : MQSQSLLLIVAAVATFLVAQTTAKFLLKDHADAEKAFEECREDYYVPDDIYEKYLNYEFPAHRRTSCFVKCFLEKLELFSEKKGFDERAMIAQFTSKSSKDLSTVQHGLEKCI : 113
             M  Q LiLl A VA  LVAg   KF  rtaqDAl AhEaC  e r P DIY K LNYEFp HkRTnCyVKCFvErmgLFtEeKGFDekAiIAQFTaKsSKnLak sHGLEKC

                 120       130       140
BdorOBP83g : DHNEHDSDTCTWANRVFSCWISVNRPIVRRTYIEN : 142
BlatOBP83g : DHNEHDSDTCTWANRVFSCWISVNRPIVRRTYIEN : 142
BoleOBP83g : DYNEHDSDTCTWANRVFSCWISVNRPIVRRTYIEN : 142
BminOBP83g : DHNEHDSDTCTWANRVFSCWISVNRPIVRRTYIEN : 142
BcucOBP83g : DHNEHDSDTCTWANRVFSCWISVNRPIVRKTYIEN : 142
CcapOBP83g : DHNEHDSDTCTWANRVFSCWISVNRPIVRKTYIAN : 142
DmelOBP83g : DHNEAESDVCTWANRVFSCWLPINRHVVRKVFA-- : 146
             DhNEhdSDtCTWANRVFSCWisvNRpiVR tyi n
```

Figure S4-31 The alignment of OBP83g

```
                    10        20        30        40        50        60        70        80        90       100       110       120
BdorOBP84a2   : ---------------------------------------------MSNGNVFVMLPLTIILLYCGIMVSAQ-DRAKDNGDIFVQHKE--------QRECVAPIMVQANGSVSSEGMDVAH :  66
BlatOBP84a2   : ---------------------------------------------MSNRNVFVMIPFTIILLYCDIMVSVE-DRAKDNGDIFVQHRE--------QRECVAPIMVHANGSNSSEDMDVAH :  66
BoleOBP84a2   : ---------------------------------------------MSNCNVLVTLPFTIMLLYCSIMMSVQ-DRAKDNGDIFVQHKE--------QRECVAPIIVQSNGSFSSEGTDVMH :  66
BminOBP84a2   : ---------------------------------------------MSNSNVLVVCPFMIMLLYCSIMISIQ-DRAKDNGDIFVQHKE--------QQECVRPIIVQANDSISSEGTDVVL :  66
BcucOBP84a2   : ---------------------------------------------MSNCNAFVALPYMIMLLYCSVRMSMQ-DRAKNNGDIFVQHKE--------QRECVAPILVQTNDSISNEGADVLD :  66
CcapOBP84a2   : ---------------------------------------------MFRKAVCIKLPLLLILVYY-IVASIQ-DHAKDNGDIYVQHKQDSKCFNNLEREANATIPLQISNSTGNELEDVVR :  73
RzepOBP84a2   : ---------------------------------------------MTSINACIQLTLTFLLIYSCNMVRTQ-SRPKDNGDIYVQHKTQSVMAGITADQTDIPKAAQTYGSNIFNVEEVMR :  74
DmelOBP84aX2  : MFHSLYLIGILSLIWVAAQDIVPDDPEVQMQMHAMFYTARVACADENLIPYVRACAVIAFLILSPNCARALQDHAKDNGDIFIINYDS---FDGDVDDISTTTSAPRE-ADYVDFDEVNR : 116
DmelOBP84aX1  : -----------------------------------MYSA-----------LVRACAVIAFLILSPNCARALQDHAKDNGDIFIINYDS---FDGDVDDISTTTSAPRE-ADYVDFDEVNR :  70
BdorOBP84a1   : --------------------------------------------MINHRLLSLALSLVLLGFLAGTRAHPETDATDNKLDKQQS------------MEPTTPTAGAAN-ETGFDFEEVVR :  63
BlatOBP84a1   : --------------------------------------------MINHRLLSLALSLLLLGFLAGTWAYPEIDATDNKLDKQQP------------METTTPNAGAAN-ETGFDFLEVVR :  63
BoleOBP84a1   : --------------------------------------------MINHRQLSLALPLLLLRVLAGTKANPQTDATNNKLDKQQS------------METTTPSVRAAN-VSGFDFEEVVR :  63
BcucOBP84a1   : --------------------------------------MSELLEMVNYRKVELALSLLLLGVLAGTTADAQSDATDNKLDKQQS------------METTTPMAEAAN-ASGFDFQDVVR :  69
CcapOBP84a1   : ---------------------------------MGITCKLLKKTMLDHRHLNIILLLLQFGIMSSGAADAQTNLTNNASDQLQA------------VENTTPMVEEEH-EMGFDFDAVVR :  74
RzepOBP84a1X2 : --------------------------------------------MVNH-QLGMTLSVLLF--LATSLANAQTNSMNNRLDVQPS------------METKTTMAENPS-AMGFDFEAAVR :  60
RzepOBP84a1X1 : --------------------------------------------MVNH-QLGMTLSVLLF--LATSLANAQTNSMNNRLDVQPS------------METKTTMAENPS-AMGFDFEAAVR :  60
                                                                                          D                 e                   v

                   130       140       150       160       170       180       190       200       210       220       230
BdorOBP84a2   : ICNNSFSIPSDYIVQFNRNGDLPEIVDKTG-MCFIRCYFEKAGLIKNWQLNKDLIMQTMWPIKADSIAICEPEAKQ-EMNACVRSYAIAKCLMKRGFQDTCNDTVA------- : 170
BlatOBP84a2   : ICNNSFSIPSDYIVQFNRNGDLPETVDKTG-MCFIRCYFEKAGLIKNWQLNKDLIMQTMWPIKADSIAFCEPEEKQ-EMNACVRSYAIAKCLMKRGFQDTCNDTVA------- : 170
BoleOBP84a2   : ICNNSFSIPNDYIVQFNTNGALSDTVDKTG-MCFIRCYFEKASLIKNWQLNRDLIMQTMWPIKADSIEFCELESKQ-EMNACVRSYAIAKCLMKRGFEDTCNHTVA------- : 170
BminOBP84a2   : MCNSSFSIPSDYIAQFNMNGALSDTLDKTG-MCFIRCYFEKAGLIKNWQLNKDMIMQTMRLIKANSIEFCEPEAKQ-ETNACIRTYAIAKCLMKRGFEDTCIQTVA------- : 170
BcucOBP84a2   : MCNSSFSIPMDYIVQFNTNGALSETADKTG-MCFLRCYFEKMGLIKAWQLNKDLIMQTMRPIKADSIEFCEPTAKQ-EVNACVRTYGIAKCLMKRGFDDTCNQTVAQNSFRAY : 177
CcapOBP84a2   : LCNSSFSIPTDHIMQFNTDGALADTVDKTG-MCFIRCYFEKSGLIQNWKLNKDQIVQKMWPLAADSVDFCESEAKQ-EMNACVRTYAIAKCLMQRRFEGTCNPSAV------- : 177
RzepOBP84a2   : TCNASFSIPMDYIVQFNTTGELSDTTDKTG-MCFIRCFLEKSGLIKNWHLNKDLIMQTMWSIIADSVELCESESES-EVNACVRTYAIAKCLMKRTLEAAGNQTLT------- : 178
DmelOBP84aX2  : NCNASFITSMTNVLQFNNTGDLPDDKDKVTSMCYFHCFFEKSGLMTDYKLNTDLVRKYVWPATGDSVEACEAEGKD-ETNACMRGYAIVKCVFTRALTDARNKPTV------- : 221
DmelOBP84aX1  : NCNASFITSMTNVLQFNNTGDLPDDKDKVTSMCYFHCFFEKSGLMTDYKLNTDLVRKYVWPATGDSVEACEAEGKD-ETNACMRGYAIVKCVFTRALTDARNKPTV------- : 175
BdorOBP84a1   : TCNASYTIPLEYIQQFNETAELPNITDKTG-MCFLKCYMEKTGLLRDWQLNPTLIRQTMWPATGDSLPVCQNEGSR-ETCPCKRTYAIAKCLTLRALVDARNKPLV------- : 167
BlatOBP84a1   : TCNASYTIPLEYIQQFNETAELPNITDKTG-MCFLKCYMEKSGLLRDWQLNPTLIRQTMWPATGDSLPVCQNEGSR-ETCPCKRTYAIAKCLMLRALVDARNKPLV------- : 167
BoleOBP84a1   : TCNASFTVPLEYIQQFNETAELPNITDKTG-MCFLKCYMENTGLLRNWQLNPTLIRQTMWPATGDSIPVCQNEGSR-ETCPCKRTYAIAKCLMLRALVDARNKPIV------- : 167
BcucOBP84a1   : TCNASFTIPLEYIQRFNETAELPNTTDKTG-MCFLKCYMEKTGLLRNWQLNSTLIRQTMWPATGDSIPVCQNEGSR-ETCPCKRTYAIAKCLMLRALVDARNKPIV------- : 173
CcapOBP84a1   : TCNASFAIPLELIQRFNETAELPNTTDKTG-MCFLKCYMEGTGLLRNWQLNRSLIRQTMWPATGDSIPVCQEEGSR-ESCPCKRTYAIAKCLMIRALVDARNKPIV------- : 178
RzepOBP84a1X2 : TCNASFPTPIEYIQQFNETAELPNTADKTS-MCFMHCYMEKTGLMRNWQLNRALIMQTMWPATGDSIPVCQNEGCNTETCACKRTYAIAKCLMIRSLVDARNKPVV------- : 165
RzepOBP84a1X1 : TCNASFPTPIEYIQQFNETAELPNTADKTS-MCFMHCYMEKTGLMRNWQLNRALIMQTMWPATGDSIPVCQNEGSRKLVLVSVRTPSLNV----------------------- : 149
                 CN Sf  p   i qFN    L    DKt  MCf  Cy Ek gL   w LN  li qtmwp   dS   C  e    e   c R yaiakcl  r   d  n
```

Figure S4-32 The alignment of OBP84a

```
                     10        20        30        40        50        60        70        80        90       100       110
BdorOBP99a    : MKTIIALCLLLAVTSAEYVVKNEENLQQYRRECATELKVPAEHIEQFRKWQFPNDAVTQCYLKCVFEKFGLFDAVTGFNVEHIHQQLQGAEVAPPGDADHDDVVHDKIAAC : 111
BlatOBP99a    : MKTVIA-CLLLAVTSAEYVVKNEENLQQFRRECATELKVPAEHIEQFRKWQFPNDSXTQCYLKCVFEKFG-FDAETGFNVEHIHQQLQGAEVAPPGDADHDDVIHDKIAAC : 109
BcucOBP99a    : MKNFIAFCLILAVTSAEYVVKNEENLQQYRRECATKLKVPEDHIQQFRKWQFPNDSVTQCYLKCVFEKFGLFNAETGFNVEYIHQQLQGAQVAPPGDADHDDVVHDKIAAC : 111
BoleOBP99a    : MKGVIVFCLFVALTSAEYVVKTEENLLQFRRECVNELKVPEEHIQQFGKWQFPNDSVTQCYLKCVFEKFGFFDALTGFNVEHIHQQLQGAEVAPPGDADHDHVVHDKIAAC : 111
RzepOBP99a    : MKFFAAFLLFAAMVSAEYVIKTEENLRQYRQECVAELQVPDEHVQQFRNRQFPNDSITQCYLKCIFNKFQLFDDETGFNVESIHQQLQGAQVAPPGNADHDDALHDKIAAC : 111
DmelOBP99aX1  : MKVFVAICVLIGLASADYVVKNRHDMLAYRDECVKELAVPVDLVEKYQKWEYPNDAKTQCYIKCVFTKWGLFDVQSGFNVENIHQQLVG------NHADHNEAFHASLAAC : 105
DmelOBP99aX2  : MKVFVAICVLIGLASADYVVKNRHDMLAYRDECVKELAVPVDLVEKYQKWEYPNDAKTQCYIKCVFTKWGLFDVQSGFNVENIHQQLVG------NHADHNEAFHASLAAC : 105
                MK   a c      SA YVvK         R EC  eL VP        kw  PND  TQCY KCvF K g Fd   GFNVE IHQQL G         ADH    H   AAC

                      120       130       140
BdorOBP99a    : VDTNEQGSNACEWAYRGGVCFIKENLQLVKHSVKPQA : 148
BlatOBP99a    : VDTNEQGSNACEWAYRGGVCFIKENLQLVKHSVKPQA : 146
BcucOBP99a    : VDSNEQGSNACEWAYRGGVCFIKENLQLVKHSVKQQA : 148
BoleOBP99a    : VDNNEQGSNACEWAYRGGVCFIKKNLRLVKHSVKPQA : 148
RzepOBP99a    : VDSNEQGSSACEWAYRGGVCGMKANLQFIKTN----- : 143
DmelOBP99aX1  : VDKNEQGSNACEWAYRGATCLLKENLAQIQKSLAPKA : 142
DmelOBP99aX2  : VDKNEQGSNACEWAYRGATCLLKENLAQIQKSLAPKA : 142
                VD NEQGSnACEWAYRG  C  K NL     s    a
```

Figure S4-33 The alignment of OBP99a

```
                     10        20        30        40        50        60        70        80        90       100       110
BdorOBP99b    : MKFCLALLSLLMVVVFAVAD-HASHSDYVVKTNEDLIRYRDECVSKLSIPSDLVDKYKEWSFPDDEKTHCYLKCVLEKFELFDAAKGFDVHKIHHQLVGA--NADHSDATH : 108
BlatOBP99b    : MKFCLALLSLLLAVVFAVAD-HAGHSDYVVKTKEDLIRYRDECVSKLSIPSDLVDKYKEGSFPDDEKTHCYLKCVLEKFELFDESKGFDVHKIHHQLEGA--NADHSDATH : 108
BoleOBP99b    : MKFCLALLSLLIAVVLAVADHHADHSDYVVKTNADLVRYRDECVSQLSIPSDLVDKYKQWSFPDDEKTHCYLKCVMEKFDVFDAEKGFDVHKIHHQLEGS--NADHSDATH : 109
BminOBP99b    : MKFFLALLSLIFAVVLADEHDHAAHSDYVVKTNADLLRYRDECVSKLSIPADLVEKYKQWNFPDDEKTPCYLKCILEKFGLYDEEKGFDVHKIHHQLDGD--KVDHSDATH : 109
BcucOBP99b1   : MKFFVALLLLIFAVVLASAHDHSGHSDYVVKTNEDLLRYRDECVSQLSIPADLVEKYKQWSFPDDEKTHCYLKCILEKFELFDAAKGFDVHKIHHQLEGD--KVDHSDATH : 109
BcucOBP99b2   : MKFFVALLSFIFAVVIADGVDHAGHSGYVVKTNVDLLRYREECVSQLNIPLDLVMQYQEWNYPNDEKTHCYLKCVLEKFELYDAEKGFDVYKVHHQLEGD--KADHTNAMH : 109
CcapOBP99b    : MKFCLALLSLFFAVVVADHG---DHSDYVVKTGEDLARYRDQCVAKLSIPADLVEKYKKWEYPDDEKSRCYLKCVLESFGLFDDAKGFDVHKVHHQLGGG--DVDHSNELH : 106
RzepOBP99b    : MKYFIAFLS-LIVVVLAYHDHSEHHADYVVKSKEDLAHARDHCVSKLTIADDLVEKYKKWEYPDDEKTHCYLKCIFEELGLYDDEKGFDVHKVHHQVAGD--KVDHFDDLH : 108
DmelOBP99bX1  : MK---VLIVLLLGLAFVLADHHHHHHDYVVKTHEDLTNYRTQCVEKVHASEELVEKYKKWQYPDDAVTHCYLECIFQKFGFYDTEHGFDVHKIHIQLAGPGVEVHESDEVH : 108
DmelOBP99bX2  : MK---VLIVLLLGLAFVLADHHHHHHDYVVKTHEDLTNYRTQCVEKVHASEELVEKYKKWQYPDDAVTHCYLECIFQKFGFYDTEHGFDVHKIHIQLAGPGVEVHESDEVH : 108
                MK   all l   vv a    h  H dYVVKt  DL  yR  CV  l i  dLV kYk w  PdDekthCYLkC  ekf   D  kGFDVhK HhQl G      dhsd  H

                      120       130       140       150
BdorOBP99b    : GAIENCAKKAA--GDDACVRAYNGFTCFLKNNAQLVQAGVEKSSK : 151
BlatOBP99b    : GAIENCAKEAA--GDDACVRAYRGFTCFLKNNAQLVQDGVKKSSK : 151
BoleOBP99b    : GAIENCAKEAAAAGDDACVRAYRGFTCFLKDNIKLVQAGVEKSSK : 154
BminOBP99b    : GAIENCAKEAAAAGDDACVRAYRGFSCFLKDNIQLVQAGVDKGSK : 154
BcucOBP99b1   : GAIENCAKEAAAAGDDACVRAYRGFNCFLKDNIQLVQAGVEKSAK : 154
BcucOBP99b2   : VAIDKCAKEAAVASDDACVRAYRGFTCFLMDYTHLIPAGN--GSK : 152
CcapOBP99b    : GKIENCAKEGDAAGEDACTRAYRGALCFFKENLALVKQNV--ASK : 149
RzepOBP99b    : KTIESCAKEGAD-SDDSCIRAYRGGICLINNNLTLVKQSFVSD-- : 150
DmelOBP99bX1  : QKIAHCAETHSK-EGDSCSKAYHAGMCFMNSNLQLVQHSVKV--- : 149
DmelOBP99bX2  : QKIAHCAETHSK-EGDSCSKAYHAGMCFMNSNLQLVQHSVKV--- : 149
                  I  CAk       D C rAY g  Cf   n  Lv   v
```

Figure S4-34 The alignment of OBP99b

```
                          10        20        30        40        50        60        70        80        90       100       110
BdorOBP99c2   : -MKYFMFIVILAVVALVQAD-DWSPKTVDDIKKIREECMKQVPSSDEEFQKRKENDYPDVESVRKYALCNSKGWGLYKEGKGFYPDRVAEQFKDDMP-EDEIKAIVNDCD : 107
BlatOBP99c2   : -MKYFLCIVILAVIXLVQAD-DWSPKTVDDIKNIREECMKQVPSSDEEFQKRKENNYPDVESVRKYVLCNSKAWGLYKEGKGFNADRIAAQFKDEMP-EDEIKAIMQDCD : 107
BoleOBP99c2   : -----------------MSK-EFHTVSIDDIKSIREECVKQVPASDEEFEKRKENEYPDVESVRKYVLCTSKAWGLYEDGKGFNADRVAQQFKGDLT-EDEIKTIVHDCD :  91
BminOBP99c2   : -MKYF--IVILAIIALVQAD-DWSPKTTDEIKSIRENCVKQVPSSDEEFKNRKKDEYPDIESVRNYVLCTSKAWGLYEDGKGFKGEHVAQQFKGDLS-EDEIKSIVHDCD : 105
BcucOBP99c2   : -MKYF--IVILALIALVQAD-DWSPKTIDEVKSIRENCAKDIPASDEEFHKRKENDYPDVASVRNYVLCTAKEWGIYEEGKGYNADRIAQQLKGDLS-EDEIKAIVHDCD : 105
BcucOBP99c1   : -MKFF--IVILAVVALAYAEDDWVPKNAAEIKVIRQECFKDFPLSEEYIQKMKSFEYPDEEPVRKYLLCTAKKLGVFCEHQGYHADRVAKQFKMDLD-EAEVLAIAEGCV : 106
BoleOBP99c1   : -MKFF--LVILAVVALAYADDEWVPKNAAEIKVIRQECFKDFPLSEEYIQKMKNFEYPDEEPVRKYLLCTAKKLGVFCEHQGYHADRVAKQFKMDLD-EAEVVAIAEGCV : 106
BdorOBP99c1   : -MKFF--IVILAVVALAYADEEWVPKNVAQIKAIRQECIKDFPLSEEYIQKMKNFEYPDEEPVRKYLLCTAKKLGVFCEHEGYHADRVAKQFKMDLD-EAEVIAIAEGCA : 106
BlatOBP99c1   : -MKFF--IVILAVVALAYADEEWVPKNVAQIKVIRQECIKDFPLSXEYIQKMKNFEYPDEEPVRKYLLCTAKKLGVFCEHEGYHADRVAKQFKMDLD-EAEVIAIAEGCA : 106
BdorOBP99c3   : -MKFF--LVILAVVTQTYAEDEWRPKNMAELNAIRQECFKEYPLSEEQLQKIKNFEYTDEEPARKILLCTVKKLGVFCEREGYNADRVAKQFKMDLD-EAEALAIVEGCL : 106
BlatOBP99c3   : -MKFF--LLILAVISRTYAEEEWRPKNAAEVNVIRQECLKDFPLSEEELQKVKNFEYSDEEPARKVLLCTVKKLGVFCERDGYNVDRVTKQFKMDLD-EAEALSIVEGCM : 106
BoleOBP99c3   : -MKLF--IVVLAVVALVYAD-EWMPKNFAEINVIRQECFKDFPLSEEYIQKMKNFEYPDEEPVRQYLLCTVKKVGIFCEREGYLADRVAKQFKMDLD-EAEAIAIAQGCV : 105
BlatOBP99c4X1 : -MKFF--IVILAVVALAYADEEWVPKNVAQIKVIRQECIKDFPLSDEYIQKMKNFEYPDEEPVRKYLLCTVKKFGIFREDEGYNVNRVAKQFKMDLD-EAEALAIVEGCV : 106
BlatOBP99c4X2 : -MKXF--LAFLAHFALAYAEDEWRPKNVAELNAIRQECIKDFPLSDEYIQKMKNFEYPDEEPVRKYLLCTVKKFGIFREDEGYNVNRVAKQFKMDLD-EAEALAIVEGCV : 106
BdorOBP99c4   : -MKFF--IVILAHVALAYAEDEWMPKNMAELNVIRQECLKDFPLNDEYIEKMKNFEYPDEEPVRKYLLCTVKRFGIFREGQGYNIDRVAKQFKMDLD-EAEVLAIVEGCV : 106
BdorOBP99c6   : -MKFF--IVILAVIALVYAKDEWVPKTEAELKVIVKECLKDFPLNNEQLQKYTTFQQPDEEPIRKYMLCTAKRVGFFSEHEGCHVDRVAKQFKLDLD-EAEVAAITEGCA : 106
BdorOBP99c5   : -MKFF--IVILAVVALAYAKDEWEPKTEAELKVIANECIKDFPLSNEQVQKYTAYQHPDKESIRNRMLCAIKKXDFFSEHEGYHADRIAKQFQIDFH-EAEVAAIAERCA : 106
BdorOBP99c7   : -MKFF--IVILVVIALTYAEDEWIPKNDTELEVIAQECLRDFPLSKEQLQKFSSFEYPDEEPIRKYMLCTAKRVGFFTEQEGYHADRVAKQLKMDLD-EAEIVAIAEGCA : 106
BdorOBP99c8   : -MKFF--IVMLAVVTLAYAEDEWMPKNDAEVSVIRQECIKDFPLSEEQLQKFRIFEYPEEEALRKYLLCVTKAVGIFTEHEGYHADRVAKQFNINLD-EAEVTIIAEGCA : 106
BminOBP99c1   : -MKFF--IVILAVVALACAEDEWVPKNVAEIKVIRQECIKEFSLSEEHIQKLKNLEYPDEEPVRKYLLCTAEKLGVFCEHEGFHANRIAKQFKMDLD-EAEVLAIAEGCV : 106
CcapOBP99c1   : -MKFL--IVVLAVVALAYADDEWTVKQAAEIKEIRQECLKENPLGDEYIQKMKSFEYPDEEPVRKYLLCTAKKLGIFCVHEGYHVDRIAKQFKMDLD-EAEVVAIAEGCV : 106
RzepOBP99c1   : -MKYF--IAILAVVALAQAEEEWNVKTAADIKVIRQECIKEFPLSQEYIQKMKNFEYPDEEPVRKYLLCTAKKLGIFCEHQGYHADRVAKQFKMDLD-EAEVLAIAESCV : 106
DmelOBP99c    : MLKYL--IVALALCAVAHAD-DWTPKTGEEIRKIRVDCLKENPLSNDQISQLKNLIFPNEPDVRQYLTCSAIKLGIFCDQQGYHADRLAKQFKMDLS-EEEALQIAQSCV : 106
CcapOBP99c4   : -MKYF-IVILAAVVLAQAADDDWVPKTPEEFNAIRRECHKEFPFSKELQKQEDELDFSDDETVRKYEVCVFRKWGIIDADDTFHGERLVKQFEAVLDGVEGIEQKVNNCV : 108
CcapOBP99c2X1 : -MKYF-IVILAAVVLAQAADDDWVPKTPEEFNAIRRECHKEFPFSKELQKQEDNLDFSDDETVRKYEVCVFRKWGIIDAEDNFHGERLVKQFDAVLDEVENIEQKVNNCV : 108
CcapOBP99c3   : -MKYF-IVILAAVVLAQAADDDYKLKTPEEFNAMARECHREFPFSKELQNQEDNLDFSDDETVRKYEVCFYRKLGILDADNNFNGDRLVKQFEAVLD-VEGIEQKVNNCV : 107
CcapOBP99c2X2 : -MKYF-IVILAAVVLAQAADDDYKLKTPEEFNAMRRECHREFPFSKELQNQEDNLDFSDDETVRKYEVCFYRKLGILDADNNFNGERLVKQFEAVLD-VEGIEQKVNNCV : 107
CcapOBP99c5X1 : -MKYF-ILILAAVVLAQAQDD-WKIKSANEVNDIRRECHKEHPFNEELQKHEEVLRFPDEDVVRNYEVCVFTKWGVFDPETGFKKDRLVRQFEPVLK-REEIDEIIGRCA : 106
CcapOBP99c5X2 : -MKYF-FLILAAFLFAQAQDD-WKIKSANEVNDIRRECHKEHPFNEELQKHEEVLRFPDEDVVRNYEVCVFTKWGVFDPETGFKKDRLVRQFEPVLK-REEIDEIIGRCA : 106
CcapOBP99c6   : -MKYF-ILLLAAVLLAQAEGE-WKVKTVDEFNDMRRDCHKEIPFSEELQKKEEVLRFPDEEVVRKYLLCIAKTWEVFDEENGFNMDRLIVEFELLLN-RDEVLPIAKRCV : 106
                 mk f      a      a   w  k       ir eC k  p s e         pd e vR y  C     g       g    r   qf    l     e   i   C

                         120       130       140       150
BdorOBP99c2   : EKTKE-ETDDERCYHLLKCVMSTKLGDHIKDLVKRLE-------- : 143
BlatOBP99c2   : EKTKE-ETDDERCYHILKCACSTKLGDHIKDLIKRSE-------- : 143
BoleOBP99c2   : AKTKE-DTDDERAYHIMKCTCSTKLGEDIKEILKRSE-------- : 127
BminOBP99c2   : EKTKE-DSDDERAYHVLMCILSSKLGGDVKELLKRSE-------- : 141
BcucOBP99c2   : EKTKE-DADDEKAFHILKCLCTSKIGEAVKQFLHKSE-------- : 141
BcucOBP99c1   : DKNVEGSSADVWAYRGHKCLMASKIGDRVKAYIKKTVEEAKKQ-- : 149
BoleOBP99c1   : DKNVEGSSADVWAYRGHKCLMASKIGDRVKAYIQKSVEEAKKQ-- : 149
BdorOBP99c1   : DKNVEGSSADVWAYRGHKCVMASKIGERVKAYIQKSVEEAKKH-- : 149
BlatOBP99c1   : DKNVEGSSADVWAYRGHKCVMASKIGERVKAYIQKSVEEAKKH-- : 149
BdorOBP99c3   : DKNLEGSSADVWAYRGHECVVASKIGDRVKAYFLKS----KK--- : 144
BlatOBP99c3   : DKNLEGSSADVWAYRGHECVVASKIGERVKAYFLKNGEKPKK--- : 148
BoleOBP99c3   : DKNVEGSSADVWAYRGHECVLASKIGDRVKAYFKKSGENTKK--- : 147
BlatOBP99c4X1 : DKNIEGSSDDVWAYRCRKCVLASKIGDRVKAKSKEESYKQ----- : 146
BlatOBP99c4X2 : DKNIEGSSDDVWAYRCRKCVLASKIGDRVKAKSKEESYKQ----- : 146
BdorOBP99c4   : DKNTEGSSDDVWVYRCRKCVMASKIGDRVKAKSRE---------- : 141
BdorOBP99c6   : DKNAEGSSVDVWAYRGHKCAMASKIGERLRVYIQNLKKEAKKH-- : 149
BdorOBP99c5   : DKIVKGSSVDVWAYRSRKCVMTSKIGERLKARNQKI--------- : 142
BdorOBP99c7   : DKNVEGSSADVWAYRVHKCVMASKIGEHGKAYFQK---------- : 141
BdorOBP99c8   : DKNVEGSSADVWAYRIHKCVMASKLGERVKAYIQNLKKEAKKH-- : 149
BminOBP99c1   : DKNEEGSSPDVWAYRGHKCLIDSKIGESVKAYIKKSADEAKKQ-- : 149
CcapOBP99c1   : DKNEQGSSADVWAYRGHKCLMASKIGDRVKSYIKKNVEEAKKQ-- : 149
RzepOBP99c1   : DNNEQGSSPDVWAYRGHKCLMASKVGEKVKAYIKKRAEDAQKQ-- : 149
DmelOBP99c    : DDNAQKNPTDVWAFRGHQCMMASKIGDKVRAFVKAKAEEAKKKAA : 151
CcapOBP99c4   : DKNEQGSPIDVYASRIQQCIDKTDIAPKLLKVIGKL--------- : 144
CcapOBP99c2X1 : DKNEQGSPIDVYASRIQRCIDKTDIAPNLLKVIGKL--------- : 144
CcapOBP99c3   : DKNEQGRPVDEYVSRIQRCIDKTDIAPNLLKVIGKL--------- : 143
CcapOBP99c2X2 : DKNEQGRPVDEYVSRIQRCIDKTDIAPNLLKVIGKL--------- : 143
CcapOBP99c5X1 : DKNEQGSPVDVWVYRFQQCVSRSEIPPNFLKIIGKL--------- : 142
CcapOBP99c5X2 : DKNEQGSPVDVWVYRFQQCVSRSEIPPNFLKIIGKL--------- : 142
CcapOBP99c6   : DKNEQGSSPDVWVYRVQQCVGKTKLGPLFLEALGKLSK------- : 144
                dkn  g   D   yr   C    k g
```

Figure S4-35   The alignment of OBP99c

```
                      10        20        30        40        50        60        70        80        90       100       110
BdorOBP99d : MLIPRFLIIPI-TIMFSVLISFPTPSKAVPIS-EHEEASRRLYKAHGFCVQNSSAPTSIDKEYRSTKQEPAAYVRCVASNMGLWTDASGYQAKRVAKFFVKEHNENEVMTVVD : 111
BlatOBP99d : MFTSRYLIISL-AVGFCVAIISLKPAKAVPIS-EHEKASRRLYKAHGFCVQNSSTPTSMNKEYRNTEQKTGAYLRCVASNMGLWTDASGYHAKRVAKFFIKEHNENEVMTVVX : 111
BoleOBP99d : MFIPRYLIIRL-AVVLCVVISFFMSATAVPIS-EHEDASRRLYKAHGICVQNISTPKSTD-SHISTEQIVGAYVRCIASNMGLWTDATGYQAKRVAKFFTKEHSESEVMTVVD : 110
BminOBP99d : ------------------TEELFNPATAVPIS-EYEEASRRLYKAQGICIQKISSPTTRD-NVASTEQKAGAYVRCIASNMGLWTDATGYNSKRVAKFFIKEHNENEVMTVVD :  93
BcucOBP99d : --MLKTIIIKL-AVVICVLINLCTKTTAIPIS-EQEEASHRLHKAHGICIQNITSPTSTD-DDTSTRQLAGAYVRCIATNVGLWNDATGYNAKQVAKFFIKERNENEVMTLVD : 108
RzepOBP99d : MFRIRAAVIFVGVIVLNAFISSTASAIPAPLTTAYEEPVRRLHKANKFCAKEISLPLAED---MDNEHIAGAYIRCMATNMGLWNDGGGYNAKRVAKFFIKQRNENEVMTVVE : 110
CcapOBP99d : MFTTRYLLIFSLLVICAISSSFTAVANPTSIS-DYEQPSRRLYKAHELCLPIVTDVSSAE--SEQGMQRAATYIRCMVSNMGLWTDSVGYKAKRVAKFFMKQHSEHEVMALVE : 110
DmelOBP99d : MNHLRLEIICWSCLLIAMAVSTEAASVWKLPT------AQMVYEDLEKCRQ-------------ESQEEDAATLRCLVKKLGLWTDESGYNARRIAKIFAGHNQMEELMLVVE :  94
             m   r   i                    pis    e  srrlyka    C q     p            q    ay RC  a nmGLWtD   GY akrvAKfF  k    e EvMtvV

                 120       130       140       150
BdorOBP99d : YCNQKHQQA-DLDLWAYEAYRCATAGRMGIWLRE--------- : 144
BlatOBP99d : YCNQKHQQA-DLDLWAYEAYRCATAGXMGVWLKEYVVSAKL-- : 151
BoleOBP99d : YCNQKHQQM-NLDLWAYEAYRCATAGRMGIWLREYMLSAKL-- : 150
BminOBP99d : YCNQKHQQA-DLDLWAFEAYRCATAGRMGIWLREYVLSAKL-- : 133
BcucOBP99d : YCNQKHRQT-DLNLWAYEAYRCATAGRMGAWLNAYVRSAKL-- : 148
RzepOBP99d : YCNQQYQQT-DVDLWAFQAYRCATAGRMGTWLGEYMLSAKL-- : 150
CcapOBP99d : YCNQKNQQE-NLDLWAFEAYRCATAGRMGTWLGEYVLSAKL-- : 150
DmelOBP99d : HCNRMEQDTSHLDDWAFLAYRCATSGQFGHWVKDFMSQKEVER : 137
             yCNqk qq   ldlWA eAYRCATaGrmG Wl ey  sakl
```

Figure S4-36   The alignment of OBP99d

```
                        10        20        30        40        50        60        70        80        90       100       110
RzepOBPlushX1 : -------MLFGLNIIRNFLPLLACIIGVSAVTMQQFETSLDMMRNGCAPKFKIPVELLDRLRDGDF-VENNSELKCYTRCVAQLAGTLTKKGDFSVQKALAQIPIILPPEMQDT : 106
RzepOBPlushX3 : -------MLFGLNIIRNFLPLLACIIGVSAVTMQQFETSLDMMRNGCAPKFKIPVELLDRLRDGDF-VENNSELKCYTRCVAQLAGTLTKKGDFSVQKALAQIPIILPPEMQDT : 106
RzepOBPlushX2 : --------------------------------MQQFETSLDMMRNGCAPKFKIPVELLDRLRDGDF-VENNSELKCYTRCVAQLAGTLTKKGDFSVQKALAQIPIILPPEMQDT :  81
BcucOBPlushX1 : -------MLVKINAFKYFLTLLACTG-VNAITMQQFESSLDMMRNGCAPKFKVATEILDNLRAGEF-IENNGELKCYTRCVAQLAGTVTKKGEFSVQKALAQIPIILPPEMQEA : 105
BcucOBPlushX2 : -------MLVKINAFKYFLTLLACTG-VNAITMQQFESSLDMMRNGCAPKFKVATEILDNLRAGEF-IENNGELKCYTRCVAQLAGTVTKKGEFSVQKALAQIPIILPPEMQEA : 105
BoleOBPlush   : -------MCLKINALKYFLTLLACTG-VSAITMQQFETSLDMMRNGCTPKFKVPIEILDNLRAGEF-IENNGELKCYTRCIAQLAGTVTKKGEFSVQKALAQIPIILPPEMQNA : 105
BlatOBPlushX1 : --------------------------------MQQFETSLDMMRNGCAPKFKIATEILDNLRAGEF-VENNGDLKCYTRCIAQLAGTVTKKGDFSVQKALAQIPIILPPEMQGP :  81
BlatOBPlushX2 : -------MCLKINAFKYFLTLLACTG-VNAVTMQQFETSLDMMRNGCAPKFKIATEILDNLRAGEF-VENNGDLKCYTRCIAQLAGTVTKKGDFSVQKALAQIPIILPPEMQGP : 105
BdorOBPlush   : -------MYLKINALKYFLTLLACTA-VSAVTMQQFETSLDMMRNGCAPKFKIATEILDNLRAGEF-IENNGDLKCYTRCIAQLAGTVTKKGDFSVQKALAQIPIILPPEMQDP : 105
BminOBPlush   : --------------------------------MQQFETSLDMMRNGCAPKFKVSTEILDNLRAGEF-AENNSDLK------------LTKKGDFSVQKALAQIPIILPPEMQDA :  69
CcapOBPlush   : MQEKYKAMILNTNGIKYFFTLLACAS-VGAVTMQQFEASLDMMRNGCAPKFKVSVDILDKLRAGEF-TENNNDLRCYTRCIAQLAGTLTKKGDFSVQKALAQIPIILPPEMQDA : 112
DmelOBPlushX1 : -MKHWKRRSSAVFAIVLQVLVLLLPDPAVAMTMEQFLTSLDMIRSGCAPKFKLKTEDLDRLRVGDFNFPPSQDLMCYTKCVSLMAGTVNKKGEFNAPKALAQLPHLVPPEMMEM : 113
DmelOBPlushX2 : -MKHWKRRSSAVFAIVLQVLVLLLPDPAVAMTMEQFLTSLDMIRSGCAPKFKLKTEDLDRLRVGDFNFPPSQDLMCYTKCVSLMAGTVNKKGEFNAPKALAQLPHLVPPEMMEM : 113
                                       l       a tMqQFetSLDMmRnGCaPKFK   e LD LR G F  enn  Lkcytrc aqlagt tKKG FsvqKALAQiPiilPPEMq

                   120       130       140       150       160       170
RzepOBPlushX1 : AREAMNACKEVQKNY-----KESCER-VFY--------TTKCVRDYAPDTFKFP----- : 146
RzepOBPlushX3 : AREAMNACKEVRKKK-----LQGIVR-ACF--------LHNEMRA-------------- : 137
RzepOBPlushX2 : AREAMNACKEVQKTTRNRASVFSTQRNACVTMLPILSSFHNFVQLFLYLLCKYMHTYVT : 140
BcucOBPlushX1 : AKAALNACKDVQKSYK-----DSCERVFYTT---------KCVRDYDPATFKFP----- : 145
BcucOBPlushX2 : AKAALNACKDVRK-----------KKL-------------------------------- : 121
BoleOBPlush   : AKEALIACKDVQKAYK-----DSCDKVFYTT---------KCVRDYDPSTFKFP----- : 145
BlatOBPlushX1 : AKEALNACKDVQKNYK-----ESCDKVFYTT---------KCVRDFDPATFKFP----- : 121
BlatOBPlushX2 : AKEALNACKDVQKNYK-----ESCDKVFYTT---------KCVRDFDPATFKFP----- : 145
BdorOBPlush   : AKEALNACKDVQKNYK-----ESCDKVFYTT---------KCVRDFDPATFKFP----- : 145
BminOBPlush   : AKEALNACKDVQKNYK-----DSCDRVFYTT---------KCVRDYDPSTFKFP----- : 109
CcapOBPlush   : AKEALNACKDVQKNYK-----ESCDRVFYTT---------KCVRDYDPTTFKFP----- : 152
DmelOBPlushX1 : SRKSVEACRDTHKQFK-----ESCERVYQTA---------KCFSENADGQFMWP----- : 153
DmelOBPlushX2 : SRKSVEACRDTHKQFK-----ESCERVYQTA---------KCFSENADGQFMWP----- : 153
                a  a nACkdv K         sc                kc        f  p
```

Figure S4-37 The alignment of OBPlush
