# Supplementary material for: Comparative genomic and transcriptomic analyses of chemosensory genes in the citrus fruit fly Bactrocera (Tetradacus) minax
Source: Sci Rep. 2020 Oct 22;10:18068. doi: 10.1038/s41598-020-74803-5 (PMC7583261; doi:10.1038/s41598-020-74803-5)
Supplement: Supplementary file 6 — Supplementary Information 6. [file 41598_2020_74803_MOESM6_ESM.pdf]

Supplementary file 5

>BminCSP_1 Bmi004355 locus=Contig1553:751269:783937:+ 751287:751469 751567:751740 len:327 chemosensory protein 1 PREDICTED: ejaculatory bulb-specific protein 3 isoform X1 [Bactrocera oleae]
QAALIFFIVVTVQCGTAQKQYTNKFDNVDVDGVLSNNRILTNYIKCLMDKGP
CTPEGRELKELLPDALQTDCSKCTDTQKKNSQKVINFLRVSRPGEWKLLLDK
YDSKGVYRSKYEKQG
>BminCSP_2 Bmi004346 127aa Bmi004346 del_header_54aa locus=Contig1553:400140:401558:- len:546 chemosensory protein 2 PREDICTED: ejaculatory bulb-specific protein 3 [Bactrocera oleae]
MKLFIFAGILALVYLTTAEDKYTTKYDNIDVDEILKSDRLFNNYFKCLIETGKC
TPEGRELKKTLPDALKTECSKCSEKQKQNTDKVIRYVIDNKPDEWKQLQAKY
DPEGIYVAKYKKEAEKQGITI*
>BminCSP_3X1 Bmi010858.1 locus=Contig865:2012169:2012717:-2012717:2012150 len: chemosensory protein [Bactrocera dorsalis]
MLRFIAATVLICTVYYVATTSAAPHPPTTAAPLAASEAAYDTKFDNIDLDEVLG
QERLLRNYIKCLENTGPCTPDSKMLKEILPDAISTGCAKCSEKQRLGSTKVTH
FLIDNRPEDWARLEQIYDPQGNYRLAYLAEKDKGDGAEKPTEAPVTKPQA
>BminCSP_3X2 Bmi010858.2 158aa ACB56576.1 locus=Contig865:2012090:2012810:- len:477 chemosensory protein [Bactrocera dorsalis]
MLRFIAATVLICTVYYVATTSAAPHPPTTAAPLAASEAAYDTKFDNIDLDEVLG
QERLLRNYIKCLENTGPCTPDSKMLKGFANFRDTPRRYFNRLRQMLGEAEAW
LNEGDALLDRQSSGGLGAFGADIRSARQLPFGLFGGEGQRRWRGETYGSAGY
*
>BminCSP_4 Bmi004554 111aa locus=Contig1558:208612:224771:- len:336 chemosensory protein 4 chemosensory protein 4 [Bactrocera dorsalis]
MKITNVSKDIGLKCIFLLLVIYTCKTDSDDKNINKLLNNQVIVSRQIMCVLEKS
PCDQLGRQLKAALPEVILRNCRNCSPQQAQNAQKLTNFLQARYPDVWAMLL
KKYQNM*

```
                      10        20        30        40        50        60        70        80        90       100       110       120
BlatCSP1X2 : MK--AAIIFLIVVVVQYGAAQKQYTNKFDNVDVDGVLSNNRILTNYIKCLMDKGPCTPEGRELKKLLPDALQTDCSKCTDTQRKNSQKVINFLRLNRPGEWKLLLDKYDSRGVYRSKYEKQG : 120
BlatCSP1X3 : MK--AAIIFLIVVVVQYGAAQKQYTNKFDNVDVDGVLSNNRILTNYIKCLMDKGPCTPEGRELKKLLPDALQTDCSKCTDTQRKNSQKVINFLRLNRPGEWKLLLDKYDSRGVYRSKYEKQG : 120
BlatCSP1X1 : MK--AAIIFLIVVVVQYGAAQKQYTNKFDNVDVDGVLSNNRILTNYIKCLMDKGPCTPEGRELKKLLPDALQTDCSKCTDTQRKNSQKVINFLRLNRPGEWKLLLDKYDSRGVYRSKYEKQG : 120
BdorCSP1X1 : MK--AAIIFLIVVAVQYAAAQKQYTNKFDNVDVDGVLSNNRILTNYIKCLMDKGPCTPEGRELKKLLPDALQTDCSKCTDTQRKNSQKVINFLRVNRPGEWKLLLDKYDSRGVYRSKYEKQG : 120
BdorCSP1X2 : MK--AAIIFLIVVAVQYAAAQKQYTNKFDNVDVDGVLSNNRILTNYIKCLMDKGPCTPEGRELKKLLPDALQTDCSKCTDTQRKNSQKVINFLRVNRPGEWKLLLDKYDSRGVYRSKYEKQG : 120
BoleCSP1X2 : MK--AALIFFIIVAVQYGSAQKQYTNKFDNVDVDGVLSNNRILTNYIKCLMDKGPCTQEGRELKKLLPDALQTDCSKCTDTQRKNSQKVINFLRVNRPGEWKLLLDKYDSKGIYRSKYEKQG : 120
BoleCSP1X3 : MK--AALIFFIIVAVQYGSAQKQYTNKFDNVDVDGVLSNNRILTNYIKCLMDKGPCTQEGRELKKLLPDALQTDCSKCTDTQRKNSQKVINFLRVNRPGEWKLLLDKYDSKGIYRSKYEKQG : 120
BoleCSP1X1 : MK--AALIFFIIVAVQYGSAQKQYTNKFDNVDVDGVLSNNRILTNYIKCLMDKGPCTQEGRELKKLLPDALQTDCSKCTDTQRKNSQKVINFLRVNRPGEWKLLLDKYDSKGIYRSKYEKQG : 120
BcucCSP1   : MK--AALIFFIVVAVQYAAAQKQYTNKFDNVDVDGVLSNNRILTNYIKCLMDKGPCTPEGRELKKLLPDALQTDCSKCTDTQRKNSQKVINFLRVNRPGEWKLLLDKYDSKGIYRSKYEKQG : 120
BminCSP1   : MK--AALIFFIVVTVQCGTAQKQYTNKFDNVDVDGVLSNNRILTNYIKCLMDKGPCTPEGRELKELLPDALQTDCSKCTDTQKKNSQKVINFLRVSRPGEWKLLLDKYDSKGVYRSKYEKQG : 120
RzepCSP1X2 : MK--AILMLLVIIAVQHASCQKQYTNKFDNVDVDGVLSNNRILTNYIKCLMEKGPCTPEGRELKKLLPDALQTECSKCTDSQRKNSQKVINFLRVNRPGEWKLLLDKYDSKGVYRAKYEKQG : 120
RzepCSP1X3 : MK--AILMLLVIIAVQHASCQKQYTNKFDNVDVDGVLSNNRILTNYIKCLMEKGPCTPEGRELKKLLPDALQTECSKCTDSQRKNSQKVINFLRVNRPGEWKLLLDKYDSKGVYRAKYEKQG : 120
RzepCSP1X1 : MK--AILMLLVIIAVQHASCQKQYTNKFDNVDVDGVLSNNRILTNYIKCLMEKGPCTPEGRELKKLLPDALQTECSKCTDSQRKNSQKVINFLRVNRPGEWKLLLDKYDSKGVYRAKYEKQG : 120
CcapCSP1   : MKT-TFLICLITIAVQLALAQK-YTNKFDNVDVDGVLSNNRILTNYIKCLMEKGPCTPEGRELKKLLPDALQTDCSKCTETQRKNSQKVINFLRSNRPGEWKLLLDKYDSRGVYRSKYEKQG : 120
DmelCSP1   : MKASLALVFCVCVGLAAAAPEKTYTNKYDSVNVDEVLGNNRVLGNYLKCLMDKGPCTAEGRELKRLLPDALHSDCSKCTEVQRKNSQKVINYLRANKAGEWKLLLNKYDPQGIYRAKHEGH- : 121
             MK  a         vq    qKqYTNKfDnVdVDgVLsNNRiLtNYiKCLM KGPCT EGRELKkLLPDALqtdCSKCTd QrKNSQKVINfLR nrpGEWKLLLdKYDs G YR KyEkqg
```

Figure S5-1 The alignment of CSP1

```
                     10        20        30        40        50        60        70        80        90       100       110       120
CcapCSP2X1 : MKLLIFAGLLALVTLAVAEEKYTTKYDNIDVDEILKSDRLFNNYFKCLIETGKCTPEGRELKKTLPDALKTECGKCSEKQKQNTDKVIRYVIDNKPDEWKQLQAKYDPEGIYAAKYKKEAEKQGITI : 127
CcapCSP2X2 : MKLLIFAGLLALVTLAVAEEKYTTKYDNIDVDEILKSDRLFNNYFKCLIETGKCTPEGRELKKTLPDALKTECGKCSEKQKQNTDKVIRYVIDNKPDEWKQLQAKYDPEGIYAAKYKKEAEKQGITI : 127
BcucCSP2   : MKLLILASFLSLVSLAIAEDKYTTKYDNIDVDEILKSDRLFNNYFKCLIETGKCTPEGRELKKTLPDALKTECSKCSEKQKLNTDKVIRYVIDNKPDEWKQLQAKYDPEGIYAAKYKKEAEKQGITI : 127
BminCSP2   : MKLFIFAGILALVYLTTAEDKYTTKYDNIDVDEILKSDRLFNNYFKCLIETGKCTPEGRELKKTLPDALKTECSKCSEKQKQNTDKVIRYVIDNKPDEWKQLQAKYDPEGIYVAKYKKEAEKQGITI : 127
BoleCSP2   : MKLFIFAGILALVYLTTAEDKYTTKYDNIDVDEILKSDRLFNNYFKCLIEMGKCTPEGRELKKTLPDALKTECSKCSEKQKQNTDKVIRYVIDNKPDEWKQLQAKYDPEGIYAAKYKKEAEKQGIAI : 127
BdorCSP2   : MKLFILAGVLALAYFTTAEDKYTTKYDNIDVDEILKSDRLFNNYFKCLIETGKCTPEGRELKKTLPDALKTECSKCSEKQKQNTDKVIRYVIDNKPDEWKQLQAKYDPEGIYAAKYKKEAEKQGITI : 127
BlatCSP2   : MKLFILTGILALAYFTTAEDKYTTKYDNIDVDEILKSDRLFNNYFKCLIETGKCTPEGRELKKTLPDALKTECSKCSEKQKQNTDKVIRYVIDNKSDEWKKLQAKYDPEGIYAAKYKKEAEKQGITI : 127
RzepCSP2X1 : MKLLILSGILAFAALAVAEEKYTTKYDNIDVDEILKSDRLFNNYFKCLIETGKCTPEGRELKKTLPDALKTECSKCSEKQKQNTDKVIRYIIDNKSEEWAQLQAKYDPEGIYASKYKKEAEKQGIKI : 127
RzepCSP2X2 : MKLLILSGILAFAALAVAEEKYTTKYDNIDVDEILKSDRLFNNYFKCLIETGKCTPEGRELKKTLPDALKTECSKCSEKQKQNTDKVIRYIIDNKSEEWAQLQAKYDPEGIYASKYKKEAEKQGIKI : 127
DmelCSP2   : MKMILALVVLGLV-LVAAEDKYTTKYDNIDVDEILKSDRLFGNYFKCLVDNGKCTPEGRELKKSLPDALKTECSKCSEKQRQNTDKVIRYIIENKPEEWKQLQAKYDPDEIYIKRYRATAEASGIKV : 126
             MKl i  g Lal  l  AE KYTTKYDNIDVDEILKSDRLFnNYFKCLietGKCTPEGRELKKtLPDALKTECsKCSEKQkqNTDKVIRY IdNK  EWkqLQAKYDPegIYa kYkkeAEkqGI i
```

Figure S5-2 The alignment of CSP2

```
                     10        20        30        40        50        60        70        80        90       100       110
BdorCSP3X1 : -----MLRFVAASVLICAIYHVT--ITSAAPHPP-TTAAPLVA--NQAAYDTKFDNIDLDEVLNQERLLRNYIKCLENTGPCTPDSKMLKEILPDAISTDCAKCSEKQRLGSAKV : 105
BlatCSP3X1 : -----MLRFIVASVLICAVYHVA--ITSAAPHPP-TTAAPLVA--NQATYDTKFDNIDLDEVLNQERLLRNYIKCLENTGPCTPDSKMLKEILPDAISTDCAKCSEKQRLGSAKV : 105
BoleCSP3X1 : -----MLRFVAALVLICTVYHVA--TTTAAPHPP-TTAAPLLA--NQAAYDTKFDNIDLDEVLSQERLLRNYIKCLENTGPCTPDSKMLKEILPDAISTDCAKCSEKQRLGSARV : 105
BcucCSP3X1 : -----MLRFIAATALICAVYHVA--TTSAAPHPP-TTAAPLVG--NQPAYDTKFDNIDLDEVLNQERLLRNYIKCLENTGPCTPDSKMLKEILPDAISTDCAKCSEKQKLGSAKV : 105
BminCSP3X1 : -----MLRFIAATVLICTVYYVA--TTSAAPHPP-TTAAPLAA--SEAAYDTKFDNIDLDEVLGQERLLRNYIKCLENTGPCTPDSKMLKEILPDAISTGCAKCSEKQRLGSTKV : 105
CcapCSP3X1 : -----MFRLIVALALILSIYHAP--SVHGAPHPP-TTAAPLVA--NQGSYDTKFDNIDLDEVLSQERLLRNYIKCLENLGPCTPDSKMLKEILPDAVSTDCAKCSEKQKLGSAKV : 105
RzepCSP3X1 : ------MRFLVAFVLIYIAYYVA--GIGAAPHPPATTSAPLVA--NQATYDTRFDNIDLDEVLSQERLLRNYIKCLENTGPCTSDSKMLKEILPDAISTDCVKCSPKQKSGSAKV : 105
DmelCSP3   : MGQPGFRRAIGHVSLVVALMCTTCFQVEGLPHPPATSPSPMMERMVEQAYDDKFDNVDLDEILNQERLLINYIKCLEGTGPCTPDAKMLKEILPDAIQTDCTKCTEKQRYGAEKV : 115
BdorCSP3X2 : -----MLRFVAASVLICAIYHVT--ITSAAPHPP-TTAAPLVA--NQAAYDTKFDNIDLDEVLNQERLLRNYIKCLENTGPCTPDSKMLKGFANFRDTPRRYFNRLRQMLGEAEA : 105
BlatCSP3X2 : -----MLRFIVASVLICAVYHVA--ITSAAPHPP-TTAAPLVA--NQATYDTKFDNIDLDEVLNQERLLRNYIKCLENTGPCTPDSKMLKGFANFRDTPRRYFNRLRQMLGEAEA : 105
BoleCSP3X2 : -----MLRFVAALVLICTVYHVA--TTTAAPHPP-TTAAPLLA--NQAAYDTKFDNIDLDEVLSQERLLRNYIKCLENTGPCTPDSKMLKGFANFRDTPRRYINRLRQMLGEAEA : 105
BcucCSP3X2 : -----MLRFIAATALICAVYHVA--TTSAAPHPP-TTAAPLVG--NQPAYDTKFDNIDLDEVLNQERLLRNYIKCLENTGPCTPDSKMLKGFANFRDTPRRYINRLRQMLGEAEA : 105
BminCSP3X2 : -----MLRFIAATVLICTVYYVA--TTSAAPHPP-TTAAPLAA--SEAAYDTKFDNIDLDEVLGQERLLRNYIKCLENTGPCTPDSKMLKGFANFRDTPRRYFNRLRQMLGEAEA : 105
RzepCSP3X2 : ------MRFLVAFVLIYIAYYVA--GIGAAPHPPATTSAPLVA--NQATYDTRFDNIDLDEVLSQERLLRNYIKCLENTGPCTSDSKMLKGFSYFRDTPRRYINRLRQMLAEAEV : 105
CcapCSP3X2 : -----MFRLIVALALILSIYHAP--SVHGAPHPP-TTAAPLVA--NQGSYDTKFDNIDLDEVLSQERLLRNYIKCLENLGPCTPDSKMLKGFAHFRDTPRRSINRLRQMLGEAEA : 105
                  m Rf  a  Li   y v      aaPHPP TtaaPl a  nq  YDtkFDNiDLDEvL QERLLrNYIKCLEntGPCTpDsKMLK                 Q lg a

                  120       130       140       150       160       170
BdorCSP3X1 : TH----FLIDNRPED-WARLEQIYDPQGNYRLNYLAAKD----KGDGMEKTTEA-VTKTQA- : 156
BlatCSP3X1 : TH----FLIDNRPED-WARLEQIYDPPGNYRLNYLAAKD----KSDGVEKTTEA-VTKPEA- : 156
BoleCSP3X1 : TH----FLIDNRPED-WARLEQIYDPQGNYRLAYLAEKD----KSDGMEKTTEA-VTKPQA- : 156
BcucCSP3X1 : TH----FLIDNRPED-WARLEQIYDPSGNYRLAYLAAKDN---KGDGAEKPTEA-VTKALA- : 157
BminCSP3X1 : TH----FLIDNRPED-WARLEQIYDPQGNYRLAYLAEKD----KGDGAEKPTEAPVTKPQA- : 157
CcapCSP3X1 : TH----FLIDNRPED-WARLEQIYDPSGNYRLAYLAEKDKGNSQSDTGEQTPSEAVTKTNEA : 162
RzepCSP3X1 : TH----FLIDNRPED-WARLEQIYDPQGSYRLAYLAEKAK---NGDDKQNTTEA-ETKA--- : 155
DmelCSP3   : TR----HLIDNRPTD-WERLEKIYDPEGTYRIKYQEMKS------KANEEP----------- : 155
BdorCSP3X2 : WLSEGDALFDRQSSGGLGTFGADIRSARQLPFELFGGEGQ-----RRWHGENYG-SGY---- : 157
BlatCSP3X2 : WLSEGDALFDRQSPGGLGTFGADIRSARQLPFELFGGEGQ-----KRWRGENYG-SGY---- : 157
BoleCSP3X2 : WLSESDALFDRQSPGGLGAFGADIRSARQLPFGLFGGEGQ-----KRWHGENYG-SGY---- : 157
BcucCSP3X2 : WLSEGDTLFDRQSSGGLGTFGADLRSVRQLPFGLFGGEGQQ----RRWRGETYG-GGY---- : 158
BminCSP3X2 : WLNEGDALLDRQSSGGLGAFGADIRSARQLPFGLFGGEGQ-----RRWRGETYGSAGY---- : 158
RzepCSP3X2 : WLSEGDAFLDRQSAGGLGALGADLRSARQLPLSLLSGEG----------------------- : 144
CcapCSP3X2 : WLSEGDAFLDRQSSGGLGTLGADIRSIRQLPFSLFSGEGQRQQPERHWRADSFG-SGY---- : 162
                    l D
```

Figure S5-3 The alignment of CSP3

```
                  10        20        30        40        50        60        70        80        90       100       110
BdorCSP4 : MKITNVTKDIGFKCIFLLLVICT-CETDSDDKNINKLLNNQVIVSRQIMCVLEKSPCDQLGRQLKAALPEVILRNCRNCSPQQAQNAQKLTNFLQARYPDVWAMLLKKYQNI : 111
BlatCSP4 : MKITNVTKDIGFKCIFLLLVICT-CETDSDDKNINKLLNNQVIVSRQIMCVLEKSPCDQLGRQLKAALPEVILRNCRNCSPQQAQNAQKLTNFLQARYPDVWAMLLKKYQNI : 111
BoleCSP4 : MKITNVSKHIGFKCIFLLLVICT-CETDSDDKNINKLLNNQVIVSRQIMCVLEKSPCDQLGRQLKAALPEVILRNCRNCSPQQAQNAQKLTNFLQARYPDVWAMLLKKYQNI : 111
BminCSP4 : MKITNVSKDIGLKCIFLLLVIYT-CKTDSDDKNINKLLNNQVIVSRQIMCVLEKSPCDQLGRQLKAALPEVILRNCRNCSPQQAQNAQKLTNFLQARYPDVWAMLLKKYQNM : 111
RzepCSP4 : MKGTKDPQSFNFKCIIVLLVIFI-CEIESDDKNINKLLNNQVIVSRQIMCVLEKSPCDQLGRQLKAALPEVILRNCRNCSPQQAQNAQKLTNFLQTRYPDVWAMLLKKYRNV : 111
CcapCSP4 : MKVSSFLRSTGIKCIFLMLVICT-SKVNSDEKNINKLLNNQVIVSRQIMCVLEKSPCDQLGRQLKAALPEVILRNCRNCSPQQAQNAQKLTNFLQTRYPDVWAMLLKKYQNA : 111
DmelCSP4 : MLLLNKNRVISLVVNFIFLIILISSSVQADERNINKLLNNQVVVSRQIMCILGKSECDQLGLQLKAALPEVITRKCRNCSPQQAQKAQKLTTFLQTRYPDVWAMLLRKYDSA : 112
           Mk          kcif  LvI       sD kNINKLLNNQViVSRQIMCvLeKSpCDQLGrQLKAALPEVIlRnCRNCSPQQAQnAQKLTnFLQ RYPDVWAMLLkKY n
```

Figure S5-4 The alignment of CSP4
