# Supplementary material for: Comparative genomic and transcriptomic analyses of chemosensory genes in the citrus fruit fly Bactrocera (Tetradacus) minax
Source: Sci Rep. 2020 Oct 22;10:18068. doi: 10.1038/s41598-020-74803-5 (PMC7583261; doi:10.1038/s41598-020-74803-5)
Supplement: Supplementary file 7 — Supplementary Information 7. [file 41598_2020_74803_MOESM7_ESM.pdf]

Supplementary file 6

>BminOR2a [gene=OR2a] [protein=odorant receptor 2a] [organism=Bactrocera minax] [moltype=protein] [country=China] [ID=Bmi006479] [locus=Contig1933:2179906:2186009:- len:1191 odorant receptor 2a]
MSLKINSWDAFKYHWCVWDLSGFRGPQKQSTWYIPYKLYTIAITLLFPIYYPTCFTVESLL
ADNLNDFCEVIYIAMADVTLNIKFLTLFIVRQQLLELRPILKHLDARARTEEEIGVLQDGID
SAKKCFLIILRLFYSAFVTSQLMVIFSVEARLMYPAWYPFDYQASRTKFWIAYGYQTIGFLV
QCTQACSVDTYPQAYMRVLTAHIRALSLRVERIGRKNFSYASSELMCLTKDEMKRNYDEL
VSSIKDHKTIIELFSTIQKPISGTSMAQFVCTGVAQCTIGVYMLYVGFNISIMLNMAVFFVSV
TMETLILCYYGDLFCQECEGLSKAIYNCNWTVQSTEFKNALSFFLLRSQRVNILMAGNWI
PVKLPTFVMLIIIIEVMSLLIDDYNKIE*
>BminOR7a1 [gene=OR7a1] [protein=odorant receptor 7a1] [organism=Bactrocera minax] [moltype=protein] [country=China] [ID=Bmi005185] [locus=Contig1665:830963:832327:+ len:1191 odorant receptor 7a1]
MFELITGRGIGIASSKDAFIYFFKGCTIVGLSPPKNSGLLYYMWSFSVNAICIIISPITGPVGF
VIKYLQNTITTVQFLSGLQAALNLIGLPVKCSTVTHALKRLRGIEPTLTIMDARYTRPEDVA
LIRKAALMGNRLVFFFGTSYFMYMLFTVIPPLINGKAPLSVWIPFFDEHQSRIHICVQIVYD
LFTMFFVLFHQSLYDSYGSVYIYVISTHLQLLVRRVGRLGTDATKSQDDNMKELVDCVVT
HQQILELLATIEPIISTTMFTQFLIISSILCVTMVNMFFFADRSTQLASTLYFLCVLLQTSPCC
YFATELKADSEQLPLAIFHCNWVEQDQHFRKVILYFMHHAQLSIELMAMQLFPINVATNIS
LAKFSFTLFTFIKEMGIGQNAKN*
>BminOR7a2 [gene=OR7a2] [protein=odorant receptor 7a2] [organism=Bactrocera minax] [moltype=protein] [country=China] [ID=Bmi008156] [locus=Contig2615:374025:384926:- len:1194 odorant receptor 7a2]
MFDLIKGRRRTVFASRDAVIYLFNSFRYVGFNPPAKYRLPYFLYSAIITFFAVLFSPVIFNVG
WLRDRNKLSVMEILTCVQASLNVMAVPLKCIALAMAMNRLRGIEPMVTELDERYTTPED
RAKIKQCAVTGNRLVFGFAVSYFLYETLTVISALVGGHAPLSLWIPNVDWHRSTWEYWLQ
VSFDAAVLFFILYHQVLNDSYPAVYIYIIRTQIQLLTSRVEKLGYDDQKSADENYQELSECIV
IHQKILKIVKIVESVVSITVFTQFLVAAAILGVTMINIFIFADLTTKIASVTYFFCVLLQTSPTC
YHASYLLADCDELRIAIFQCNWIAQNKRFNNLLIYFLHRSQDSIPFFALKLVPINLATNLSTS
FREGY*
>BminOR10a [gene=OR10a] [protein=odorant receptor 10a] [organism=Bactrocera minax] [moltype=protein] [country=China] [ID=Bmi009555] [locus=Contig5068:234314:237294:- len:1065 odorant receptor 10a]
MNFRFLSRTFSLHDYYFYVPKLCLGALGFWPLDTSEPNASNVWAWMNLIILTIGVFTEIHA
GCSALRTDLELALDTLCPAGTSAVTLLKMALIYYYRKDLAWVLKRMRGFMYTRDVSINT
VKEHIVRAHAVMAARLNFIPFVMGFITCTSYNLKPLLMTLILYVQGQEPMWKLPFNMTAS
ALFHYSMPSFLLQAPYFPLTYIFTSYTGYITIFMYGGCDAFYFEFCSNIAALLELLQNDLKSI
ISFGEGLIKTFKFPSQSVQLATVAFGCDWLACNPRLRRYVLMIILRSHRAINMAVPFFAPSLV
TFTSILQTSVDHDPLGSKKSERCSNEQVEAKDGEEEVEKEKKRKKVEN*
>BminOR22c [gene=OR22c] [protein=odorant receptor 22c] [organism=Bactrocera minax] [moltype=protein] [country=China] [ID=Bmi009742] [locus=Contig5835:1170:16990:+ len:1236 odorant receptor 22c]

MRRLLGSEVPIERSFFRIPRFSARIAGFWPLSFDRPLSWLTALRFCVNTFAVAVGGFGEVSY
GFVYLHDLFGALEAFCPGITKVISLLKMTVFFVRRKRWIHVINSMRQLLLLDISAEKRRIVE
PLASFASLLSFVLLLSGSVTNTFFNTLPLLKMGYYKWQSLEAELLLPFNVILPEMLPIAILPS
HIPGADALRRHDCFSLLVRSMVSSSALARLSRLAVCFFLVVFCCQWSTQRCAKYADTASL
WRWLVERHNKIIDLCSDFALEFYSYSDAFLFRHRWCCASQHFGLMLNSASVGVLTYIFYSI
AALTQLFLYCIGGTYVSESSLKVAEVIYDIDWYKCDVRTRRMLLMMICRAQKAKTIQVPF
FTPSLPAFRSARKSIIDQISPKRCERNQSTLIRAKHEEEEAVE*
>BminOR24a [gene=OR24a] [protein=odorant receptor 24a] [organism=Bactrocera minax] [moltype=protein] [country=China] [ID=Bmi010522] [locus=Contig7932:8746:10806:+ len:1179 odorant receptor 24a]
MLLKFLTQSYPTEKNIFLIPRLALRIIGFYPEESSSRRRQAFLIFNWVILVYGCYAEFMFGIQN
LSIDVVRALDALCPVASSIMSVVKVTFLWYHRAELNRLIKRVTELNAAQDSPLKSAYKHR
YFATATRLSASLLFFGFITATLYGTRAGMINYLSYLRKDEIPFETPFKMIIPKSLLSMPLFPLTF
ILSLWHGYITIAGFAGTDGLFLCFCMYIGTLLKALQYDMKDLLSDVHSGVCKSSAEYEIKE
SLKMIIARHNEIIDLVKRFSAVMSGVILGHFVTSSAIIGTGVLDMLLFSDYGVFVYLLHTMT
VTTELYLYCIGGTVVIECSSQLATAAYDSHWYTHSVEVQKMVLLIILRAQRSLVIKVPFFAP
SLSVLTSVSKQLRLCTVF*
>BminOR33ab1-1 [gene=OR33ab1-1] [protein=odorant receptor 33ab1-1] [organism=Bactrocera minax] [moltype=protein] [country=China] [ID=Bmi000272] [locus=Contig1018:631231:632461:- len:1170 odorant receptor 33ab1-1]
MGKKLLVVKSSVNTPVDTVACFDIFWMCWKLMGISINSNKWYVTLYDIVVNIFVTIFYPIH
LTVGLFLVPVLSDVFKNLAIIITDVACSTKHYLFRYKLSKIREIQRLLKELDDRVVAQDERD
YFNKSIRSAVRRMMLIFCVSYAGDTLASALEVLAKKDRELMYPAWFPFDWSANRYTYYA
AVIYQIVGVSLQITQNLAHDTFAPVSLCVMSGQVRLLAMRVSKVGYDESKTLAEHEEELN
ECIEDHKKLLRIFDLLQDVFWYTQLVQFSSVGLNICLTVVFLLQFADNIFAYLYYTAYFISM
ALELLPACYYGSKMQEEFQDLPYAIFKSNWIAQRKSFQQNLRIFTELSKKQLTPTAGGIINI
HLTSFVATCKMAYSLYTVLMNMK*
>BminOR33ab1-2 [gene=OR33ab1-2] [protein=odorant receptor 33ab1-2] [organism=Bactrocera minax] [moltype=protein] [country=China] [ID=Bmi000270] [locus=Contig1018:624095:625322:- len:1155 odorant receptor 33ab1-2]
MAGSNQVLQSVDSVVLYRAFWLCWHAVGISTAYNKYFCGLYNLLINVLVTIFYPIHLFLGL
FLNPTPADLFQNLSITITCFICSIKHYLLRRKLPQIHTVQVLLAELDKRVEGADEHAYFQQQ
LVVGAKNVVKLFSIAYGGANMAAISATLLSKERRLLYPAWLPFRWEASTFNYCAAVIYQIA
GVTIQIVQNLANDIYPPMSLCIIAGHVHLLALRVAKVGEDAKKSMKQHNQALIRCIEDHK
KLVRIFELTQDTLSQAELAQFISSGLNMCIVLFYLIFYVDNVFAYIYYAVYFVSMAIELLPSC
FYGSMLIYEFQQLPSAIFKCSWLGQSREFYQNQRIFVQVSLKKIVPLAGGVIGIQLNSFLGT
CKMAYSLYTVCNRMK*
>BminOR33ab2 [gene=OR33ab2] [protein=odorant receptor 33ab2] [organism=Bactrocera minax] [moltype=protein] [country=China] [ID=Bmi000271] [locus=Contig1018:628126:630717:+ len:1959 odorant receptor 33ab2]
MAIGISVVACTAKHLMLRPKLHKIIFVNDILHKLDRRVQTDEDMRYYLKKMRDNCIFMIH
FFTVVYFSVAIMAVLSALWTGNILYPAYVIVDWHGSTWKYLLVMLFQIYGLNMQIVQNLT
NDTYGPMILCLLSGHVHLLSRRILRIGHDSETDLDRNYEELVLCIDDYKVLMRIVERIISCS
YVVQFTAVGVNVVVGLIYLLFFADNLFAYCYYVFHILAIMIEIFPCCYYGSMVQAEFHALS

YAIFRSNWLSQSRTFRRTAVTFTELSLKDVTVWAGGMMKIHLDSFFKTCKMGYSIFTGKH
TKHLRLIYDILMNTYITFGFPAHLVLGIIFSTNQEQFFINLVIGIASVSCVFKHLLLRFRMRE
MQSVNEIFAQLDDRVRTNEDYDYYKRLIERPCNFMVNFFTRCYFAVSITALTMALVTGELL
YPAYIPLQWRTSVFKYAVGILFQFAGVTLQIVQNIANDAYGPVVICMLSGHVHLLANRVSR
VGHNNAESVEDSYRELSMCIEDHKLLMSTTTKVERIVSASYLVQFVGVGINICIGLIYLLFF
ADNYFAYVYYTIHIIAIMIELFPCCYYGSMLECEFHDLSYAIFSSNWPTQPRLFRRNIVNFTE
FTLREVTMYAGGMIRINLDSFFATCKMGYSFFTVIQSMK*

>BminOR35a [gene=OR35a] [protein=odorant receptor 35a] [organism=Bactrocera minax] [moltype=protein] [country=China] [ID=Bmi002525] [locus=Contig1301:1915483:1918074:- len:1254 odorant receptor 35a]

MDYFVPLQFDNRPVKLPIQVTGYKFNFLWPLKEDANILSRLVHNICLSVSVLCYIGTILGES
TFISENISNIPAVAECLCTSFMGVQYIIRIFVLLRRQRPLRKLLQNFYRDIYFTYADDAALCK
EINTVIRFMKMFTQFYYVPMMLILILYVYDVASIGLASPHKPFIYRMSFRWYDAQAPQQFII
TAIYSGWLTISTVTLWTAEDYTLCLVLCHASFRYKKLRLDLQQLLEMARADLKSGETRCT
NRNLHTAFRRRLCEIFQRQQLLNGFVAEAKAHFTYQIFYVMFFGVLLLCVVSFQFQNAPIT
VDSSKYISWLISQTAQFLLIGYFGQMLMDETTELRNSFYCCRWEDLLVHGDPHSNKLLLS
DVKFAIMNSQEPIVLDGMKFFPLTYSTVSVALRSAVSYFMFLNTMNGEN*

>BminOR43a [gene=OR43a] [protein=odorant receptor 43a] [organism=Bactrocera minax] [moltype=protein] [country=China] [ID=Bmi005218] [locus=Contig1679:27013:31484:+ len:1146 odorant receptor 43a]

MSTAVEDNPLLSVNVRLWKFLSVLFARDWRRCVAFVAPVGLMNAMQFVYLYQQWGDLA
TFILNTFFAVSVFNALLRTCFIIKNRDKFEALMRELVTLYDIIQDSGDDYAKSVLAAATKSAR
NISIFNLSASFSDLVVAMAYPLFRQQRVHPFGVALPGIDVTHSPLYEIIYIGQLSFPFTLSSMY
MPYVSLFATFAMFGKATLQILQNDLKNLCDNMKSKTEEELFELLRTNIAYHARIARYVSDF
NELVTYLVLIEFLLFSCVICSLLFCINIVSKTTSTAEKISIVMYIGTMLYVLFTYYWQANGVL
EMSLLVSDAAYEMQWYKCSRRFKRTLLIFIGRTQKPLQIRVGQMYPMTMEVFQSLLNTSY
SYFTLLHNLYND*

>BminOR45a1 [gene=OR45a1] [protein=odorant receptor 45a1] [organism=Bactrocera minax] [moltype=protein] [country=China] [ID=Bmi003431] [locus=Contig1360:740860:742322:+ len:1173 odorant receptor 45a1]

MFKSGLNIKGYFHLQKFTFHRLGIDITTRSAHVTRIYLLILQIVALATILISMASYSWQHIQDI
GEVTNAMTPFMQATITLWKIWLVIYRRREMAEIVENIYLISAKASTKELTHLIQENNRERL
MNTAYYYSVLNTGMLALTAPVLVSFIQYLRLGEFSYILVLKATYPIDYARPLNYFLIWLWS
AVSIYGVIYGSVSVDSLYSWYIHNLVGNFKILQSKLVSAESTADLSERRTHIYYCIAYHQRII
AMTEQLNLIYQPIVFVQFSLNALQICFLAYQIGSGVVATVDLPFLFLFMISVGIQLMIYSYG
GQHLQNESVNVSKSIYQTINSSAWPIDLRKVLLISMMRAQKPSKLTGIFFDVDLPLFLWVW
RTAGSYVTLLRSVDQKNL*

>BminOR45a3 [gene=OR45a3] [protein=odorant receptor 45a3] [organism=Bactrocera minax] [moltype=protein] [country=China] [ID=Bmi003430] [locus=Contig1360:734108:735637:- len:1167 odorant receptor 45a3]

MYKYLRIPYFSFRVIGINLWAQQDQRITSAPCRYYSWTLATAIIIILMGFYIYTSDQDKAIQV
LTVFLQGVLCIIKSGMFVAKGRRFIKLIRNLDALADAATVKEWSEWRHENDWQQCIVRVY
YICCTSTGTLYCTVPAVILLYSQCFREHTVFILPFDAAFPLDTGHLFFYTVSYLWCISFIIYAIH
AITAMDSLFCWFIFNISAHFRTLQHELQNVAKTSADVEDSACLHSKITETLHYHRRIIELSA

EFDELYAPIVFIEISVSYLKLCFSAYNLINLGDISGLPVIAVGLVTITFQLCNYCFSGEKLKSVS
KVVSDRIYLAFSWERVPPPLRRLLILPLMRAQRATNLTGFLFVVDHSLLVWIFKTTGSIIGFL
SATKSENTTS*
>BminOR46a [gene=OR46a] [protein=odorant receptor 46a] [organism=Bactrocera minax] [moltype=protein] [country=China] [ID=Bmi004385] [locus=Contig1553:1964416:1965652:+ len:1236 odorant receptor 46a]
MVSPQQSTTPEIDEYLLGLFGLPKHYSSFWHYMYKLYFWHVAIFWMLIFDISMWIKIIGNIS
NLNEIIKVFYLCSMAIAVMAKFMRIHLKNNSYVEIFARMHDDDLLPVNVSELEKLAQSSH
LSCRVRNYYTYLSLTSLTLIFVTKLISEPDELPLSIYIPISVESFWRYLIAYLFQFIGLSLCCLL
NISFDSLSASFFIYLKGQLDILAYRLENIGTNLDVDDNMINLQLRYCIQHYVKVRKITENME
DLLSIPMFVQMISSVLVLVANFYAMTFLTDTSDYVTFIKFLVYQLSMLSQIFMLCYLANEVS
LRSAELSYSLYSSEWTRCSKMNQRLMLLMMAQFGVPIRIKTVNRCYSFNLPAFTSKKKPK
ESSSKEQEDSTEHQSRNVINSTELETRKICSSDLTGIEFVK
>BminOR47b [gene=OR47b] [protein=odorant receptor 47b] [organism=Bactrocera minax] [moltype=protein] [country=China] [ID=Bmi004946] [locus=Contig1639:505520:508387:- len:1281 odorant receptor 47b]
MISLSSKATIDNSISSHNSYLTNKSYTHLKHTASKLQTILAPYRVLKEMLRYGEAVHPPHTC
LFYFRAYIRLLGLWPTERTAENPLYYFYNVLIMILFSFFMLYLIFFKMALFRMRNGETDIIISE
FDALHVQHANLLRDGPRNQRILQWQRRFFFGEICFFSGFYILSLFLFAAMSLQPLLSQQTLP
FRCKFPFGLDDPDEHPMAFACVYFFQCFCTLYMLVAIVVMDSLGGNSFNQTTLNLRILCEN
IRHLGESAAGTAGACSITSELVVWRELREAVEFHQKIIGLMNRINQTFYWNYVSQMGASTF
MICLTAFEALLAQDKPMVAMKFQTYMFSAFMQLLYWCWMGNRTYYDSMEVATAAYDV
HAWYEHSPLLQRQIMFIIKRAQKPLEFRAKPLFGFTFASFTSILSTSYSYFALLRTMSD*
>BminOR49a [gene=OR49a] [protein=odorant receptor 49a] [organism=Bactrocera minax] [moltype=protein] [country=China] [ID=Bmi002639] [locus=Contig1310:104546:106156:- len:1185 odorant receptor 49a]
MDFVEFFWLPNALYRVVGYDFQQLARAHWRQTIMKAFLIFTTISGICTRIYMLFQLRELIL
SGDILNSFRLGVYISYAIDSNVKFFVFLLNAQRLRVIYQSLSNEYPMTSIKRKVYQVDKYTF
KRAHLMIVAYLSVTNSILLGPMLQSIFVYIIDLSHYGYTVAAFSYLHPTPMSYNFNYCTPHY
YILIYISEYLNGHFCTTTNLGTDLYVCTFAGQFCMQLEYLGYSLEIYEPSVKNSKTDCEFLI
EWIRKHQLMLNLCSELNEVFGTTLLFKLISNCAVFCIIVVQLKLEGFGFGFLNFLSFFFVAVS
QFFMVCQYGQKLITISENLALCAYKNRWYNGSQTYKTLLFNIIARAQKPARLTAKGFQPIS
LATFQIVMTMTYRVFAVLQRALD*
>BminOR63a1-1 [gene=OR63a1-1] [protein=odorant receptor 63a1-1] [organism=Bactrocera minax] [moltype=protein] [country=China] [ID=Bmi004869] [locus=Contig1630:134511:137029:- len:1248 odorant receptor 63a1-1]
MMMENAEEIYKRNYNYIKVLIRVSFSLGVNLTAPSKIKDALKLISVILSVTSLLSMYGHCC
YLMRHLDRMPLIAEAVFTALQILMAAIKLIYFFFTHRTFYRLLDQTLTHEIIRKMEILQQDFP
INRQLKKEIDDIMNAVWRNIRRILLFYFFCCVGIIANYFFTALFQNLYHHLKQTPNYEFILPV
PSQYPFWEKKGMAFPYYHLQMYVTGSALYVSGLGAVSFEGVFMVLCQHAVGLVKVHNL
LVLRATSAQIPVERRLEYLRYTIFNYQRINKYMHEIQTIFRHISLSQFILSLIVIGFVLFEINYG
LGSNIIIFIRLIMYISASITQITIYCYHGQALTTVNEKIPLAYYNCNWYGENKTFKQLIMMMI
MRTNKEFYLEVSWFTLMNLATLISLIKASVSYYLLLQHFQEN*
>BminOR63a1-2 [gene=OR63a1-2] [protein=odorant receptor 63a1-2] [organism=Bactrocera

minax] [moltype=protein] [country=China] [ID=Bmi004870] [locus=Contig1630:137870:141751:- len:1260 odorant receptor 63a1-2]
MILENEEEIYKRNYNSSKVLFRVSFALGVNLTAPSKAKNTLKIVHVILIVSSILSLYAHWCYL
MRHFDSIPLIAEAVFTALQILMAAIKLIYFFFTHRTFYRLLDQTLTHEIIRKIEILQQDFPINRQ
LKKEIDDIMNAVWRNIRRIFLFYFFCCVGIIANYFFTALFQNLYHHLKQTPNYEFILPVPALY
PFWEKKGMAFPYYHLQMYMTGSALYVSGLGAVSFEGVFMVLCQHAVGLVKVHNLLVLR
ATSAQIPVERRLEYLRYTIFTYQRINKYMQEIQTIFRHISLSQFLLSLIVLGFVLFEINYGLQIF
KGSNIIIFIRLIMYISASITQITIYCYHGQALTTVNEKIPLAYYNCNWYGENKTFKQLIMMMI
MRTNKEFYLEVSWFTLMNLATLISLIRASVSYYLLLQNFQEN*
>BminOR63a2 [gene=OR63a2] [protein=odorant receptor 63a2] [organism=Bactrocera minax] [moltype=protein] [country=China] [ID=Bmi004730] [locus=Contig1608:3254925:3260579:+ len:1200 odorant receptor 63a2]
MYSIIERKELRVHNIWRIRELKRISYIIGINLNAQTKLKRSLRIINVLIIIFSCIALYPHWLMIK
QAQGDIPLIAETSTTALQTTTALIKMAYMLFNQHKFHKLLQKVETHDLLQRIEIFLTGMPIK
PNLKREINAIMETNWKQTRRQLLFTIGTCICIMCNYFFYALFKNLYNHLQGTPNYVYILPFT
GYPMFLDKGMASPYYAMDMFFGACSLFAAGMSALSFQGSFMVLCKHSCGLVQVVCLLL
LRSTSAMVPKPQRVEYLRYCIVQHQRTLEFINDVNRLYRHICLSQFLHSLAIYGFVLFEMNF
GLESNKVTFIRMIMYLCAALTCDCLQHVNGQFLANELEKIPLACYNCEWYHETDDFKKT
LKMIIMRSNKKFCFQISWFTVMSLATLMGVS*
>BminOR67c [gene=OR67c] [protein=odorant receptor 67c] [organism=Bactrocera minax] [moltype=protein] [country=China] [ID=Bmi008170] [locus=Contig2615:487416:491445:+ len:1215 odorant receptor 67c]
MLPEARTFSEFIYIPIKVYQTIGEDIYEHRSPGRVRRLLLKSLLYIGFFNFNLHVLGEIVYFVK
ALNSFATILEATGVAPCIGFSFVADFKQIALTIHRGTLRKHLDQMEELFPKTAMQQVEYKLR
EHERAMRRVMYIFTLLCLAYTTTFSLYPALKASVQYWLLGAPVFERNFGFAVWYPYNATE
KTWVYWLTYMGQVHGAYLAGVAFLSADLILVASVTQLCMHFDNISRCLEEFSGASQKTS
AQEDLQYLQALVVKHAKCLELSEHVNSIFSFSLLLNFLTASLTICFIGFQVTASSIEDIVKYII
FLTASLVQVFVVCYYGDELMTASMRIGDAAYNQNWFGCDRQYKWLLTIMIMRSQKPACI
RAPTFPPISFRTYMKVISMSYQFFALLRTTYSGKGN*
>BminOR67d1 [gene=OR67d1] [protein=odorant receptor 67d1] [organism=Bactrocera minax] [moltype=protein] [country=China] [ID=Bmi000893] [locus=Contig1067:243913:245328:+ len:1167 odorant receptor 67d1]
MTVKQIRPSDSFAKLIKMARLVSSLVGADVSTENYRVNIVTIIVIFCIIIYFIFTATTVASVFSE
DWTYLLEASCMVGSVLQGITKLISGIGRTTDVSGMRLELEDLYRRYESKGETYCRVLNER
CDRVWQVIKMVGHIYGASVVGILLLTTILVIKTNEKIYVMHFFIPGVDVETSFGYLLTTAVH
TVVFLAGAFGLFAGDLFFLIYLGQPELFRDILILKIKELNEAAAQKDNKTEHLLINIIQWHQ
YYTDYNERCNEVFYYIITMQIMTSGVSIICTMYIILMGDWPGAYLYILIALCGLYLYCIIGTN
IQTCNAAFFDELYNINWYELDVKRQKMMILILMKSQNPSEIKIGGVLPLSVQTALQITKTIY
GIFTMMLGFLDEEQ*
>BminOR67d2 [gene=OR67d2] [protein=odorant receptor 67d2] [organism=Bactrocera minax] [moltype=protein] [country=China] [ID=Bmi000894] [locus=Contig1067:247755:249223:- len:1167 odorant receptor 67d2]
MAVKRVRPSESFAKIIKFFHLISSLVGADVSDENYRVNIVTIILIFCIVIYFIFTGTTVASVFSEN
WTYLLEASCMVGSVLQGITKLISAFAFAKEICGTRMELENLYREYEAKGDDYSEVLNKSC

ERVWQVIKMVGQMYLIAGAGIILITVVLIFASTEKVFLMHFLIPGIDVHTQAGYLITLAVHT
VCFLFGAFGLFAGDLFFLLFLGQPMLFLDLLALKVKSLNEAAAQKSKTAKRLLIDIIEWHQ
YYTDYNLRCNHMFYYINSMQIVTSGISIICTLYILLMGDWPGAYLYILIAFSGLYLYCIMGT
KIQTCNTAFCQELCNINFYDLEVKNQKMIVPILMKAQNPSEIKIGGFLPLSVQTALQITKTIY
GIFTMMLGFLEENQ*

>BminOR67d3 [gene=OR67d3] [protein=odorant receptor 67d3] [organism=Bactrocera minax] [moltype=protein] [country=China] [ID=Bmi001522] [locus=Contig1141:1873563:1874918:+ len:1161 odorant receptor 67d3]

MQTETRPSDNFHKLLRIIRLSSSLIGVDIIDENYKFNYVVAFVLLAIAWNYMCSIYTICKDLT
TDWTVLLDVFSPISCATQGVVKMCSLILYPKLYRELALDLLEIYKKYQVVGPNYEAKLFE
WNKSMKKILIIGGLVYFVSALLALVTPLFLYIYKGERHLIIMCQMPYVDVATDHGYFITIGY
NVLCVFVAAFGLYGADLYVFLFLTHSIFFYDIFALKVGDLHKILHENNKDKRIKSLINDITE
WHQYYLEFNDKCNVIFFWSITAHVLCTTLGILSTLLIIMLKYWPGAYPYIFVCFVWLYMYC
ILGTRVEICIDQFCDGIYDINWYDLDVSDQKTVSLMLMESQVPRVITIAGIEPLSVNTALKIT
RSIYSLVMMVMQFNE*

>BminOR71a [gene=OR71a] [protein=odorant receptor 71a] [organism=Bactrocera minax] [moltype=protein] [country=China] [ID=Bmi004512] [locus=Contig1553:5022464:5028175:- len:1047 odorant receptor 71a]

MAFDSMANFRTLTRLLIILGLWHGGNGSWFERHYRYYQLFLHTTLTFTFTLLMCLEVIYSE
SLNYAIDVLKYMLCEMALVFKVLNAWYYARKISELMNEWQVSEIFALRTSDEKQMWQKT
QKNFHKLTMTYICTGFNSAMCALIGVLLMGASELPYALWMPKNWRETYFWGMYCYEFL
AMPFTCVCNITIDLFQAYLLLHLTLCYRVISMRLERLDNAGTDEAITKEFLNNIKMQQRVK
DENPVHCLAMLQYVLIVSLQMFLPCYYANELTVESEKLGIHLYSCDWTGFLVDFSPAAPN
RSTAPSQRRSTFAIQRSAFEVQHAVGQIVHTTYNHMNFGMQFDLWSLQY*

>BminOR74a1 [gene=OR74a1] [protein=odorant receptor 74a1] [organism=Bactrocera minax] [moltype=protein] [country=China] [ID=Bmi008540] [locus=Contig3074:406405:440169:+ len:1461 odorant receptor 74a1]

MLYRPRLENGKLISLSWPIAAYRLLNNICWPLQDDATQLERLFDRFLWAFGFLIFMQHNDA
ELRYIILNNNNLDEMLICGPTYLILVEIHLRAFQLGLKKNAFKRFLQKFYAEIYIDEVSHPKL
HGNMRKRLRPIWFYSFLYFGTLSSYVIMPLINYLKNVKAPLYKMYYPIDIMPNPIYVAVVL
SNIWVGFTVITMVSGEDNVLSEVMIHLNGRFLLLQQKLRQDAERLLHAADDRNIADELRR
RIVEAIKENVRLYKFAEDFKLEFSFRLFVNLSFSAGLICVLGFKVYTTNEMSSTFYESNWEL
VILRSRDTKANVRLMKTLLLAISTNQKPFVLTGFNYFSVSLTAVLKQQLASTNNNIPMADL
VMAFSCDSFGNKCYIFSRELGLSTAIFNKVIARVANEVSIAEYNIRMEISNNIVRGINTFLIEE
SPHFADKFRPPYTHTHVHTVHSVQGTCHSFGAMKLSWRFTRSDASSGIAAALAC*

>BminOR74a2 [gene=OR74a2] [protein=odorant receptor 74a2] [organism=Bactrocera minax] [moltype=protein] [country=China] [ID=Bmi006486] [locus=Contig1934:70696:73794:- len:1107 odorant receptor 74a2]

MRYLPISYHKPLLPNGRHPPIDWQLYGFFCSNGWPLAAHITKTRYIVDILVTMMQFMSES
MVLIGEAVVMYDNLDNISFVCTVLAPNLILFEMMLRAYNIIYKRNSFRTHVEEFYKKIYIQ
RTWNPELFEKIRRQHLPTKYSTFTYIITLVTYVYVPISGLVKNERLLPFPIKFGFDYTVPWPR
YVVFLAMSIWTGFAVVGPLIGEANMLAMQILHLNGRYSLLLQDLRKISRDSIAEHERCKD
KDNMLVTQRFRCRLFEIIRRNVELNDFAKSLQEQYSFRVFVMMALSATLLCVLGFLTATTD
ELSTSYYCCDWEEVIFHSRDAEENKKIMKLIALAIHLNSKPFRLTGLNFSVVNYETVVSLA

FC*
>BminOR82a [gene=OR82a] [protein=odorant receptor 82a] [organism=Bactrocera minax] [moltype=protein] [country=China] [ID=Bmi006911] [locus=Contig2103:127264:129844:- len:879 odorant receptor 82a]
MMDYIIYHIDDLALATASLSIVFTNVLTVIKTSTFLTYKREFKSLMAEFERMYDESGAKRCL
VTLNVGAKRFVKLYFYSVSCTGLYFTIKPLVSMIWAKFQNKPLLLELPMPMRFPFDFESTP
GYQFAYVYTIFITIMVVMHAASVDGLFVLFTTNLRGHFVALQYFIETNTFDKSDALLQREL
RFYVQYHVRLLDLSQSVQHIFKPIIFGQFLMTSLQVIWALSCSAVGTAVQMSQWYNLPPRH
RHVLRLMMLRSQREIIISAGFYEASLANFMSILKAAMSYITFIQSIE*
>BminOR83a1 [gene=OR83a1] [protein=odorant receptor 83a1] [organism=Bactrocera minax] [moltype=protein] [country=China] [ID=Bmi011217] [locus=Contig900:6114563:6116706:+ len:1233 odorant receptor 83a1]
MSSNEEKKKSDYLTTVQQGCPLYGQRPDMFRLMRWNLCFAAMYRLPLERYFPACLRLLA
ITLDWTYELCLYSTMLHIDILFMYTLYLNKDKDDLELIVSCMIQTVIYTWAIVIKVFFKRVK
PKRVEELMRYLNEECQTRSAPGFTYVTMKESADLANKCSAVFLIWCYAGVICWLFVPIFN
QDRSLPLACWYPIDYKVPVVYEIVYFLQTVGQLQVASAFGCSSAFYLQVSVLFSGQFDVL
NCSLKNILATTYLILRKPESELILLREKQNIANYELNQYYNAKEHNSDFDCIAHLVDVKTPK
PKKFYEAFKIAFSPCVTQHLYILNGLKMLEELYSFLWFLKTIQVTMLICLVAFACVKRSPWY
LHSSEIKQDVLFFILNAQRPFRLTGGKMYNLNVEKFRSMDLRQPQ*
>BminOR83a2 [gene=OR83a2] [protein=odorant receptor 83a2] [organism=Bactrocera minax] [moltype=protein] [country=China] [ID=Bmi011218] [locus=Contig900:6118081:6121777:+ len:999 odorant receptor 83a2]
MIPPSKRNIDTTERCANPNGCITNEKRCDLFVYVRWLLFSFAVRPIPFDKVLPPSMHGYSAL
INVVLEIFLHFSVIHIFVLFICTIYFNYDNGDLEFLISSGIQVLLYFWAIVIKVAFRHIYPELVD
GILDFVNEEYVLHSAVELRDKERIPDEYTNEFFVAKELPFDLDCLPHLLNTADTMRTRSFRE
AFKYAIEPCVKHHIFILNALRKLESLYNKIWLLKTIEVTFAMCMCAFHLVKSSNDTQFLQV
LCLGQYTVLGLLEIFMICYAGEIIYVNSQRCDEALLRSSWYPHLREVRADFLLFLIHAQRSF
ELTAGKFNPLRLDKFRGVSS*
>BminOR85bc2 [gene=OR85bc2] [protein=odorant receptor 85bc2] [organism=Bactrocera minax] [moltype=protein] [country=China] [ID=Bmi001695] [locus=Contig1207:1634762:1636675:+ len:993 odorant receptor 85bc2]
MENLEKFVRLANFFGNHIGMKPWGRLDGFQRRFLFYFSSINLLITFIAESSYIIVTITSDFILA
VMTLSYVTFIVVAYAKWYYLYTYQTERTAFFQRLETLFPCTKSQQEAIKLSEYIRMNKLSTI
SYTISFLMCISTYTFYTIARQFIYTNLLHVPGTERNLPYQAVYPWDWRDNWTYYVCYVSQ
GFAGWHSTCAQMAFDLMLCTLSTHLIMHYDHISRSLEGYQTKFAEIHGKNGLTQQARAA
MELKAVKDDIKFISSIVAYHTELLRFDWSHADIRFQKMTILVAERAQDPAALKATKLITISR
DTMTVIMQMSYKFFTVLRTMYGD*
>BminOR85d1 [gene=OR85d1] [protein=odorant receptor 85d1] [organism=Bactrocera minax] [moltype=protein] [country=China] [ID=Bmi001692] [locus=Contig1207:1504148:1505546:- len:975 odorant receptor 85d1]
MSNQPIHFESFNYLANIFYTSIGLYAYEQAGERTKDRAAYIRRQLLSIFSIIVIVNMNIALLSQ
LMYIFLAFANNNNFVETTMLSSFVVFVIVSDFKIYNICRQRARISAMMQALHALYPQTLAE
QTKYKVQWQLQRYNRLAYAFAVFYEMLVWGYNLFPLLNYLIYEVWLALRVVEKTLPYN
CWTPFDWHNNDWIYYHMYLMLSASGQACISGQLANDLLLSALAVQLIMHYRELARRIEA

HVAGRETSSDDSEYIRHAVYNHNWFDADMSYRKMLIFIMARAQKPSKLQATTFMTVSMS
TMTDVSNSKYIVSYNIFHMYSKI*
>BminOR85d2 [gene=OR85d2] [protein=odorant receptor 85d2] [organism=Bactrocera minax] [moltype=protein] [country=China] [ID=Bmi001691] [locus=Contig1207:1498210:1500581:- len:1263 odorant receptor 85d2]
MSTQIVEFDRFIKTANFWYSFNGIVAYDDIYRQPGDEPKRRSVQTRLATVLRQIFSFVSLAN
LTWVLIIETLFVVVNFVENADFLEAARNFTFMGFVIVAIIKYLSNLKQRSRLSVLMQKLYEI
YPKQSTDQRPYDLQSHLRHYRRIGFIYAFLYAFTVWAYNSLPMVNYLLLAPLLQQTVYER
VLPYSCWVPFEWRDNWLYYPLYVSQALAGHEALAAYLASDLLLCAAMVQLIMHFRKLA
RDIQAYQAGSSCTKQDVMAQQAKRDLSFLSTAVYYHNRTLALCQLINEIFGLPVLINFISTS
FVMCFLAFQFTVGVSFDDLIMLVFYMICSLVQIYMICSYGQELVTASENIGHVVYNHNWL
VTDIRYKKLLIMIIERAQKPAILRATSFLNVSMGTITDLLQLSYKFYALIRTMYAR*
>BminOR85e [gene=OR85e] [protein=odorant receptor 85e] [organism=Bactrocera minax] [moltype=protein] [country=China] [ID=Bmi007558] [locus=Contig2333:492873:496795:+ len:984 odorant receptor 85e]
MDFSEQVESTLLYSHEDKPRIANLFVAQVTSFKVTGQIPFNVGYRLGYIYCFFVILQTLHM
GVLFLKTSYDMLLSGKLEQITDALTMSIMFWSGVYATCYWFLRSQRLLAFLQRINEHYWH
HSLPGLSFVSWHRTFVLTKRLTTVWVLTCVVSTVSYGLAPLVMGVHALPLKCWYPFDPL
NEQATLPETTDDDELNQYMYCREHLTNLSTLQHLYSQQPAVTFPEALHLAIGERSMVRILN
LVQYLTLTLIELLMFTYFGELLRRHSVRSGEAFWRSQWWTHAVAIRQDIFILLENSKRAVR
LTAGKFYAMDVDRLRSLISQQPIVG*
>BminOR88a [gene=OR88a] [protein=odorant receptor 88a] [organism=Bactrocera minax] [moltype=protein] [country=China] [ID=Bmi007186] [locus=Contig2177:57657:59015:+ len:1206 odorant receptor 88a]
MALQQERNGAAKVCAIDDLCAILHPAQRYLGLNYLNFRRVNGRFAIPRSKLLNIALFLAV
VDCTGNVIKCGKAINARDVTKAQEIFAVFGMGLVMTMRGLQLALNRDKLSKFYNDIDRIF
PRSENLQQHMEVEKVHNYIKRRFFLLHTTLTVTVSAFLTMPAMKFLLFYDFESNDIVADEF
HVNPSWLPFGVKDKVTTYPYIYIYEVILASAAVNMIVTWDEVFVVLISQLCMYYEYLGKL
LEEMKVQDAMDAKKINAFYKQLHDYIYMHQYLNNLAVDLNDLFNFSILFSDAGIAISICF
NLVLITDATDYLQMITYASPLFVEVWLIYDAAKWGTLLETVTARINEILYEQKWYDSSARF
GKYTMMWIQSTNEPFRLTAFNMFYVNMKHFQDVCSKCFL*
>BminOR94ab [gene=OR94ab] [protein=odorant receptor 94ab] [organism=Bactrocera minax] [moltype=protein] [country=China] [ID=Bmi011849] [locus=Contig953:283315:284727:+ len:1143 odorant receptor 94ab]
MRETSASVLGTRIGIARVLMRLLQILGLWPMWQARQQSSTSTRCRIWLTRYYRYLLHAPL
TFTYNTLMWIEALTRWERADHILYISITEVGMMALTLNFWRLDKRAWHFMHELSYSDRL
ALRNKAERQWWRKQQRFFARVVVCYIGGGAGVLVTAFGATLHMSGYYLPYDYWLPFE
WHNAQNYWYAYGYEVIAMSLTCISNVTMDMILCYYLFHVALLYKLIGMRLMALQQLHEP
LAVQQLMNIIELHKRVKRLTAQCESLVSVPILVQIVLSAFILCLCAYRLQSMQISENPGQFLA
MLQFASVLTLQIFLPCYFANEITINSDALTTCVYNSSWEEFSPSTRKQMNLYMELLKRPAHI
KAGNFFLVGLPVFTKVAAMI*
>BminORCO [gene=ORCO] [protein=odorant receptor coreceptor] [organism=Bactrocera minax] [moltype=protein] [country=China] [ID=Bmi011214] [locus=Contig900:6030904:6047567:+ len:1554 odorant receptor coreceptor]

MQPSKYVGLVADLMPNIRLMKYSGLFMHNFTGGSGLFKKIYSSIHLVLVLVQFLLILVNLA
MNAEEVNELSGNTITVLFFTHCITKFVYLAVSQKNFYRTLNIWNQANSHPLFAESDARYH
AIALAKMRKLFTLVMLTTVASAVAWTTITFFGESVKFALDKETNSTITVEIPRLPIKSFYPWN
AGAGMFYMISFVFQCYYLLFSMVHSNLCDVLFCSWLIFACEQLQHLKGIMKPLMELSASL
DTYRPNSAALFRSLSANSKSELINNEEKEHTDLDISGVYSSKADWGAQFRAPSTLQTFNG
MNGMNGTNPNGLTRKQEMMVRSAIKYWVERHKHVVRLVAAIGDTYGGALLLHMLTSTI
MLTLLAYQATKITGVNVYAFTTVGYLGYALAQVFHFCIFGNRLIEESSSVMEAAYSCHWY
DGSEEAKTFVQIVCQQCQKAMSISGAKFFTVSLDLFASFAIEVCGFALFVAIMSGEGCARR
VAIWLVWSVHSGHSPLSQNLFVFALIYITNTVLL*
>BminOR1-1 [gene=OR1-1] [protein=odorant receptor OR1-1] [organism=Bactrocera minax] [moltype=protein] [country=China] [ID=Bmi010399] [locus=Contig760:1261200:1262805:- len:957 odorant receptor OR1-1]
MGKLKVLSSRIFPSDPSKGKIGSIQYNVWLAQLFGVPVVGLKAESPCLKIVLGIYGILITLV
VTFIYTGFEIYDMILCWPNLDSLTQNICLSLTHIAGVLKLMAFYHGEFENKIPLTIYASLVGS
TGVLGIMYLLHSQIFPYRVKLPDWMPLGTQLAYMGISVLVFALQIVAIDYLNVTMINQIRF
QLKILNLAFEELKLDCVNKKEHINLNKRLQTIVEHHCLLRDLRNDVEDLFRLPVLIQFFTS
LVIFAMTGFQAIVKAENSNGAALIYCYCGCIFCELFVYCWFGNEVSEQSKTLTSSGYSSHC
LNRERLSQQA*
>BminOR2-1 [gene=OR2-1] [protein=odorant receptor OR2-1] [organism=Bactrocera minax] [moltype=protein] [country=China] [ID=Bmi012274] [locus=Contig982:348766:357197:+ len:1101 odorant receptor OR2-1]
MHPTRRHRWPYYIYSACINISLGIFLPATMIAKLFFIENLSQLIGLLYLGVTLTMATTKQWSL
WLHRSQLLAVHYYLGKLDARCMRHAVDRQHILTAIRICHLYYAAYMFLYELSSSGFAYIGF
KLRQLVYDGWFPQFYADAAQNLTVTLIYQNFAVMTFFVLQNVNNDMYPQCYLAILIGHL
RALTARISRIGKDGVLNVDENIEELTNCVEDHKNLLGYFNCIRPVISRTIFIQFAITAFVLCLT
AVNYVAFERDAAQKLIAATYIFAVLIEALPCCWYVNSLMEECGELTTALYNCQWYDQNRK
FRKMLIIFMQRSQHTMVLMAGDLVPITLQTFLNLFCLCVQSIYALCSDIYSIIYLILLVA
>BminOR3-1 [gene=OR3-1] [protein=odorant receptor OR3-1] [organism=Bactrocera minax] [moltype=protein] [country=China] [ID=Bmi001029] [locus=Contig1067:2655921:2657619:+ len:858 odorant receptor OR3-1]
MKRVGYFDQHRLASCLLTVPIFICVSSAYRTYIIRNDFDEVTISLFKISGAYVTTARSLIVMY
KAQEFLNFFDGIDRWYQELQCDGNEITLKKVHEFMRIVKKASKTILILTALTLFYITVIQLL
ATADYEHKSISFGTFLIKDLQYKLESTTDMTELEALKCIKKCVKDHVMIIKYHNDLEVLFS
SGSSVSVSIFGITPCVIIVFSTMVSSNRSNLFLCYIFKFKFTIILHLQDHDMSLLIADIQLSLLV
MISTFIFFWVANDFCCEVIWDYFPHIYNICYYNL*
>BminOR3-2 [gene=OR3-2] [protein=odorant receptor OR3-2] [organism=Bactrocera minax] [moltype=protein] [country=China] [ID=Bmi003768] [locus=Contig1403:591622:609321:+ len:1128 odorant receptor OR3-2]
MTSYQNMPLYSVNIKIFLKLGLIGSGARTMRVLLGLVLILTYSGQLINFCKTWNEDIGESG
MNFHVVALIMHSVVRFFVVLKKAKKFERFFQRTEQWFTDIEQNSDPGVVRMLQDVITHA
RKVTRIGFYTSIIGALCAYIYPFSFEERKFILDIDYFFFDAKQTPFYEFFFLLQALILVPVFIFV
YLPFANFLLMSLKFGEVILMDLCTKLRNISNQDEATQLRELKECISYHEKIITFRNDLEYLV
SIDGFFHIALFSLMLCMLLFFLSLVHDIRLILTALAFISFHIYIIGITYYYADNFSNESLKVAYA
AYDTPWYEGNSELRKCVQVMIARSHRPLEIKSGGLYPMTLENFQAILRISYSYFSMLQGFN

QQ*
>BminOR4-1 [gene=OR4-1] [protein=odorant receptor OR4-1] [organism=Bactrocera minax] [moltype=protein] [country=China] [ID=Bmi006901] [locus=Contig2083:263072:268337:-len:1068 odorant receptor OR4-1]
MMLFIVYDNLETLLDITEFILFWGFTLNAMMKGSTMILMRCEIESILKGLIVRHPKTEEERV
AFQLVPYFKTINASNKYLSMWHLSITSIFALHPMLASIYGYISREDENESFEFTLPFMMGYY
YDTNRPLPYVITYFTQCCGAFYMSLLFLSGDLLLVSLVHLVNMHFGYLIYRIESFQPTGTD
ADMKVLGPLMEYHNEILDYAERIDSTFSLATLLNYVGTCLVLCLIGLQIALGSEILNVFKFL
AFLISTIVQVFFVSYFGNNLKDLSTGISDAFYNHPWHDANYKYMRMLVIPIARAQRYAHLT
AFKFFEISMDSFKSYKELFGIEESGTSIATEDKTQNRKMFSFTMVNT*
>BminOR5-1 [gene=OR5-1] [protein=odorant receptor OR5-1] [organism=Bactrocera minax] [moltype=protein] [country=China] [ID=Bmi005394] [locus=Contig1697:2592421:2593658:+len:1158 odorant receptor OR5-1]
MSFEEKPPGVKELFRTHWTVWKWLGQVTHPEYPKLHIAYTILLNISFSIGYPLHLLLGQLN
LKTMQDVLLNLTISVPVAVCTLKYFNIWRNLAKVRHLEQMFNTLYARIDHPEEWIYYRKVI
IPYALKVLHLFYFICVGTAITSELTLLIMGFAYEWRLMYPAYFPFDPYATKAGYVTAHVFQII
GLMVQLAENLVSDTYGGMCLTLLAGHANLLGQRVASIGYDERKTQEENNRELVDCIIDHN
VLFDCHRTLGDIIGFGLFVQITSASLIMGVVIIYVIFFVGNAFEFVYYALFLFACIMEVFPTC
YYATYFEIEFEKLTYQMFSCNWMDQNREFKRNLIVCVEQSLKTRYFRVGGMFRINLQIFFA
TCKGAYSVLAVALRLK*
>BminOR5-2 [gene=OR5-2] [protein=odorant receptor OR5-2] [organism=Bactrocera minax] [moltype=protein] [country=China] [ID=Bmi007381] [locus=Contig2236:327376:328608:-len:1173 odorant receptor OR5-2]
MPATETTISGVSVFHFHDVTLQYLGLMPPTKKLYRYLYYVYSLVLNIIITVGYPTHLMIGLI
KSENKSDVFKNMSINFTCSACSIKIFAFWWRLAEVQKIYAIISKLDKHIVRSTDYELYKIYAL
RRAQHVLYFILVIGLGAAISSEVATIIGGFLGEWRLMYPAYFPFDIERSLWGYPIAHIYQCFG
VTAQIFQNLINDTLPPMALAMLAGHVRLLNVRVARIGHDTSVVSVRKQACNTEFLLCVED
YKALLEFRVAIQRLCSLGTFVQILVTAINMGVVIFYLIFFVNGIFTYIYYVVFLIAMPLEIFPL
CYYGTSVQMEFEELTYGIFSCNWMDQNAAFKKNLRIFAEQSLRTQIVIAGGMFAVNLDTF
FGTLKGAYSLFTVVVQMK*
>BminOR6-1 [gene=OR6-1] [protein=odorant receptor OR6-1] [organism=Bactrocera minax] [moltype=protein] [country=China] [ID=Bmi007806] [locus=Contig2464:168072:169281:-len:1098 odorant receptor OR6-1]
MPERRKWLYALYSLIPNVLVTIWLPVSFVFSYATMTTEDLVPSSLLTSIQVAINVIGCSVKIV
VMAFLLPKLRTANVFMDRLDARCRAEEEIAELRKIVQQGNRFVVLFAMSYWSYASSTFLG
SVIFGRPPYALYNPFIDWRKSRLEFIAASLMEFALMDVACFQQVVDDSYAVIYVCILRTHM
NILLMRLGRLATNTETKLEENLEELKLCIIDHKNLLGLYDIVAPIISVTIFIQFMITASILSATLI
NIFIFADQLSAQIACCFYILAVVVEIFPLCYYAQCLMDDSERLSQQIFHSNWIAQDVRFRKM
LVFFMQRTQRVMELNAGKIFPITLGSFLNIAKFSFSLYTLIKKMGIRERLGLE*
>BminOR7-1 [gene=OR7-1] [protein=odorant receptor OR7-1] [organism=Bactrocera minax] [moltype=protein] [country=China] [ID=Bmi011393] [locus=Contig914:106338:107659:-len:1152 odorant receptor OR7-1]
MSKILRVSRAKIYKSRDAVSYLFNIFTFLGTNPLEHRSRRYYFLYYFYSFTLNFISLLYCPLSF
HIGYIKLTHVLTNSQLLTAIQNAIQVSGIPIKVVAITWYMKRLQHAVEILDELDVNYTQRED

LAKIRECVRRCRKVILLFCLPYYSFGISTIALGVLQNQAPLTVWVPFLDGKRAAWEYWTIV
LWDTLVMFILLCHQLGNDTYPPIFIKVISTHMQLLVTRVNRLGRPGALTADKHYEELLASKI
VAPVISVTLFTQFATTATTLLNWFGDMEYPENIISLAFFCCQLVQILPCCLSASQLIADCERL
PDAIFHCNWMDQDRRFRRAILFFLQRTQTPIRFSCLKLFDANLETSVAIGKFAFSLYTLIHEA
EDGGKTDN*

>BminOR8-1-1 [gene=OR8-1-1] [protein=odorant receptor OR8-1-1] [organism=Bactrocera minax] [moltype=protein] [country=China] [ID=Bmi002251] [locus=Contig1291:3119669:3122246:+ len:1038 odorant receptor OR8-1-1]

MFQDQVRLPLQRRITSPENTRNMRKLTDLLYGRGAAKFESNESFQLIFQCWSLFGIKPLKQ
YRSGRLLHMCFCWFCLILCPFSFYMGYLQTLQTAPVMVQLSLLQATVNVLGLPLKAIVITI
FQTHLRSAEPIFVRLDERYQSTESREQIKNCVALSTRLFTIVGFMYHLYGGITYFQALVTNN
YPLRTWLPFTDYIPQPTIRYWAHFMFEVFHMAFLLSVQFTMDVFPAIYIRNLRTHLNLLTER
VSQLGGNPDFTDEQNYDELVDCIIMPTCYQASMIEEYSTKLPDAIFHCNWLAMDKRCRKL
TIYFMHRAQENVTFVALKLFKINLTTNLSVSKSCCKEQQ*

>BminOR8-1-2 [gene=OR8-1-2] [protein=odorant receptor OR8-1-2] [organism=Bactrocera minax] [moltype=protein] [country=China] [ID=Bmi001974] [locus=Contig1263:54980:56532:- len:978 odorant receptor OR8-1-2]

MTVILTTTLQATLNVQALPLKATVASIYLNRLRSVESIFKSLDARYQSPQGRFAIKDSVMKSA
HLFFIVSVSYFTYGTISWLSSVFTHTQPLNIWLPFVDWIPQPTIRFWMHFIFEVLYVHFLLII
QFTNDVYSVIYLKALRTHITLLAERVSKLGENPEFNDDDNYEELIDCVRSHQELLHLVGSV
LSLTIFLQFTVAAVILCVCMLNIFIFADASHQAITIVYYVCVMLQTLPACYQASMLKADSTN
LPNAIFHCNWLAFDKRSRRLLIYFLHRAQEEISFLAAKLFEINLGTNLSRKGIIPAKRPYISM
GYSQVIIFEMLRSV*

>BminOR8-1-3 [gene=OR8-1-3] [protein=odorant receptor OR8-1-3] [organism=Bactrocera minax] [moltype=protein] [country=China] [ID=Bmi012276] [locus=Contig982:386075:389553:+ len:1167 odorant receptor OR8-1-3]

MQKFSDVLYGRVESDCDTNKPFKTLLHLYGLIGIKPKPKGFLPTLHMVIVWMAFGFTPLLS
IVGFIRFQKTATITESLTRLQAVINAIFIVVKSLVVLVNLKRLENVEPVMKSLDERYNTTQER
QQISDCVAACTRLYASMGCLYYSYGTLSILSALISHKQPFGVWYPFLDLISNPTIYFYTCLLL
EACYGYFLLAAQYLHDIYPTLYMRTLRTQIQLLRARISRLGEDPDMSDKENHKELVECIDT
HQKILQVVDMVGSVCSPTIFIQFSVVAIVHCICMVNLFIFADTINKVITILYYATVGMQILPT
CYEASTLEMESSKLPDSIFHCNWLALDKRGRRLITFFIQRAQVEVSFVAIQMFEINLRTYVA
IFEEAFTYGKSFTVH*

>BminOR8-2-1 [gene=OR8-2-1] [protein=odorant receptor OR8-2-1] [organism=Bactrocera minax] [moltype=protein] [country=China] [ID=Bmi009485] [locus=Contig4893:4740:6866:- len:1188 odorant receptor OR8-2-1]

MRNVLNLFYRRGKDDFETNESFVLLFRSWSSVGFIPKKPKRIADIIHQLICWTCVIICPYIYF
SGVIETMSYLPITIVLANLGAAINCIAFPLKAFYIKANIDRLHDVGTIFKDLDGRYQRPQDQ
MQIRDLVTNSRRIFAVSFILCWFYATLSGLVALFAHEYPHGNNLPFIDWLPESNFKFWLHFT
FEVMFLQYLVQVNLTNDSFPAIYIRAIRTHVSLLTDRVSRLGSNPDLNDQENFEELVDCIVS
HQKLLQISDTVGTILSLTTFFQFTIYAAIICVCMLNMFIFGNATTKVVTVVYLIPVFWQTIPT
CYQASMLEGDCAKLPLAIFHSNWLALDKRCHKLIIYFMQRTQKEISFTAIKLFQINLRTNLS
IAKFSFTLYTFINAMDFGKTR*

>BminOR9 [gene=OR9] [protein=odorant receptor OR9] [organism=Bactrocera minax]

[moltype=protein] [country=China] [ID=Bmi007416] [locus=Contig2256:29896:32987:+ len:1212 odorant receptor OR9]

MVHREHDNISGGRGVITVLKLLGLWHYEGAMRIPYILFSGLLHSICTIPYTIMMCLDVLQA
TDLKKFTNTMYMTLTELCLVVKLVNVWCYSRLLVDFFAAFEHDKLYQLQDVEERLNWR
RPQRNFARVVFIYITVSFSAMVSAFIGVLYREDYELPYPYAPPFDWRTPRGYWYAYFYELL
AMPITGLSNCAFDMIQSYMLLQLSLCFKLISARLACMGALQEDGASSGFCEVKFHREFVDI
VNLIMTLQIFLPCYCGNEIIQHSGSLNNAIYSTEWFRCSPRMRKYLIIYMEMLQRPVCVRA
GNFFEISLDTVAIYPACVRAGTEVRNVQRVACATNHGQTIKATMTYELPGFPCIFAQPLLTL
DHLMTVLARDRVGPLRLHTGANVLHSRHGIINALRKVIN*

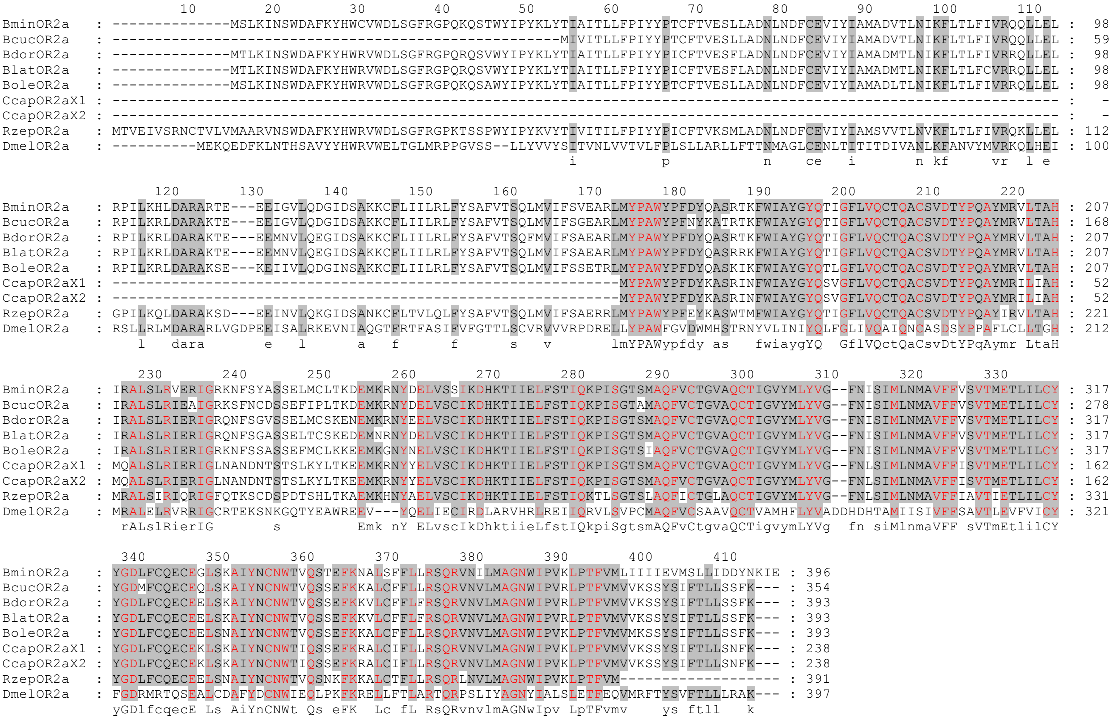

Figure S6-1 The alignment of OR2a

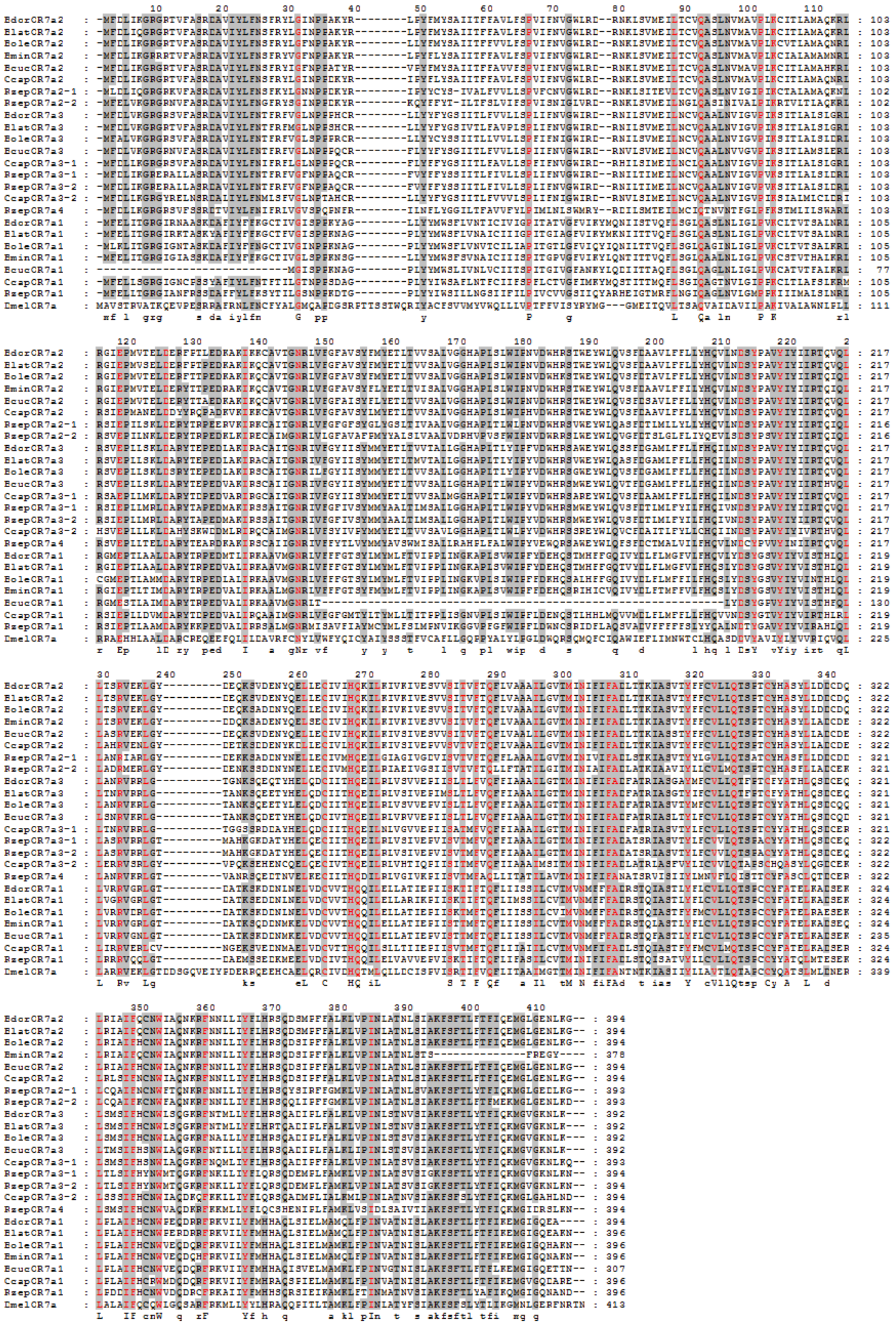

Figure S6-2　The alignment of OR7a

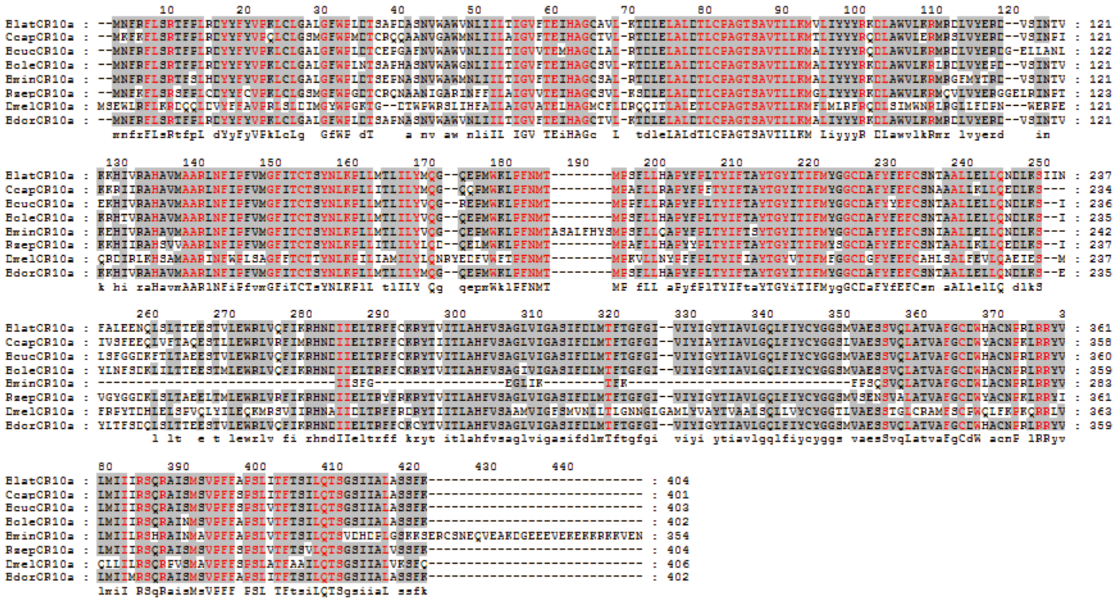

Figure S6-3　The alignment of OR10a

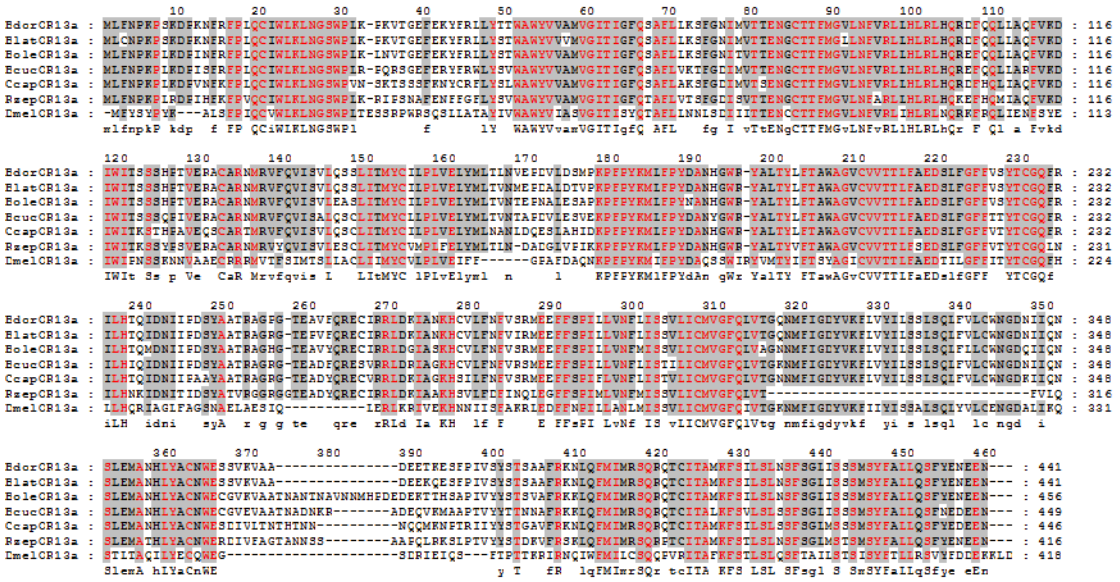

Figure S6-4　The alignment of OR13a

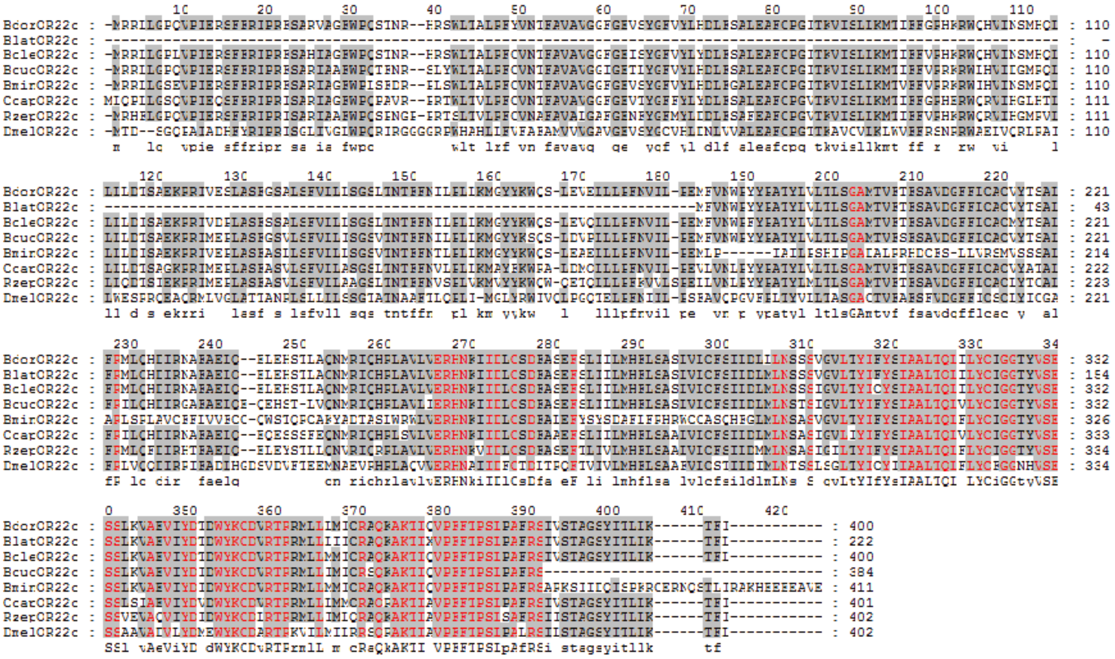

Figure S6-5　The alignment of OR22c

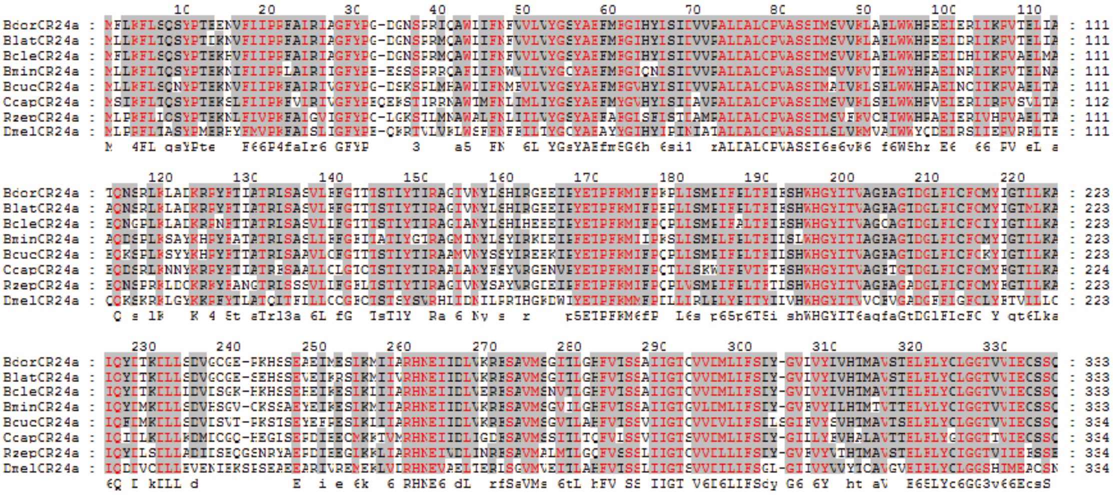

Figure S6-6　The alignment of OR24a

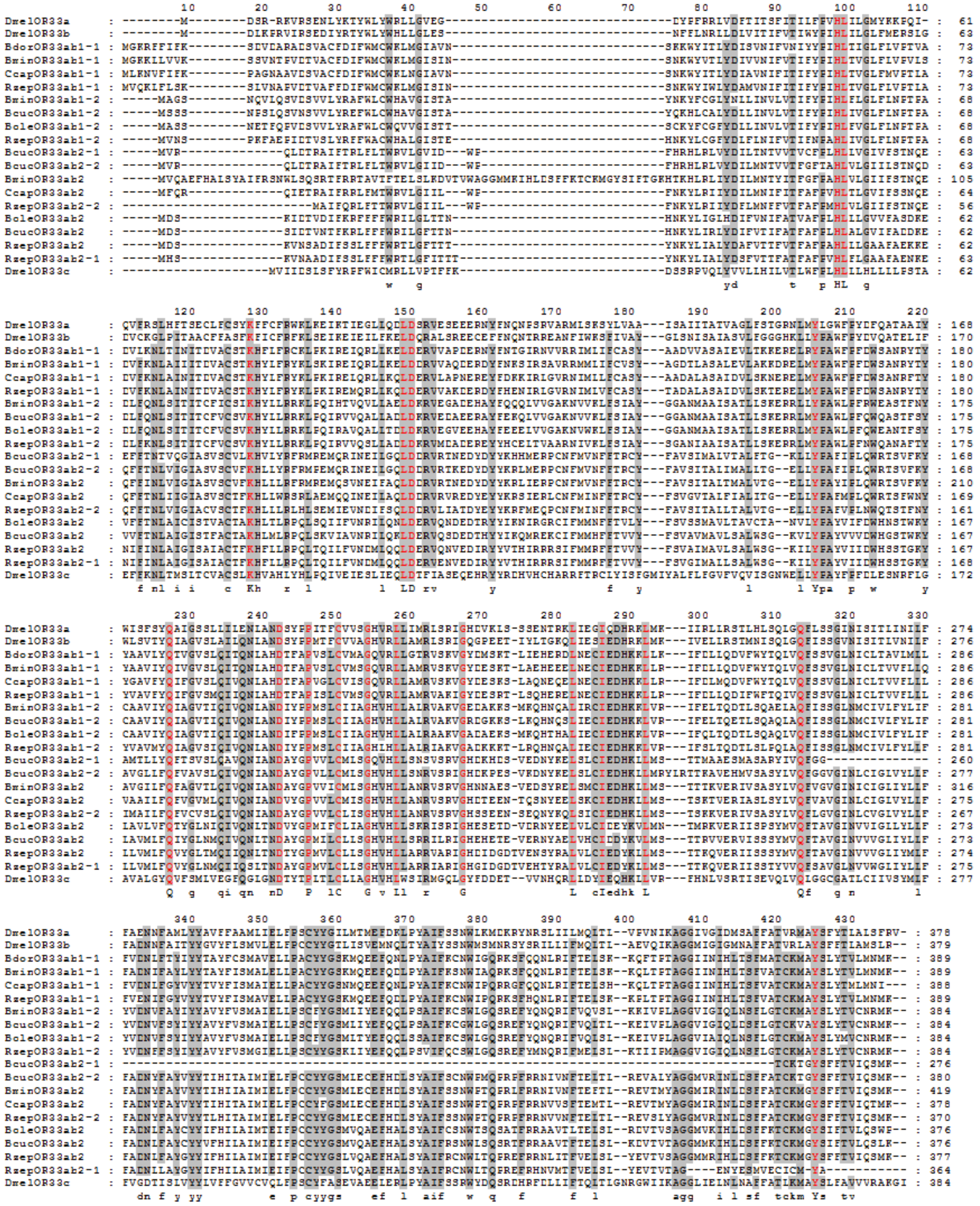

Figure S6-7 The alignment of OR33abc

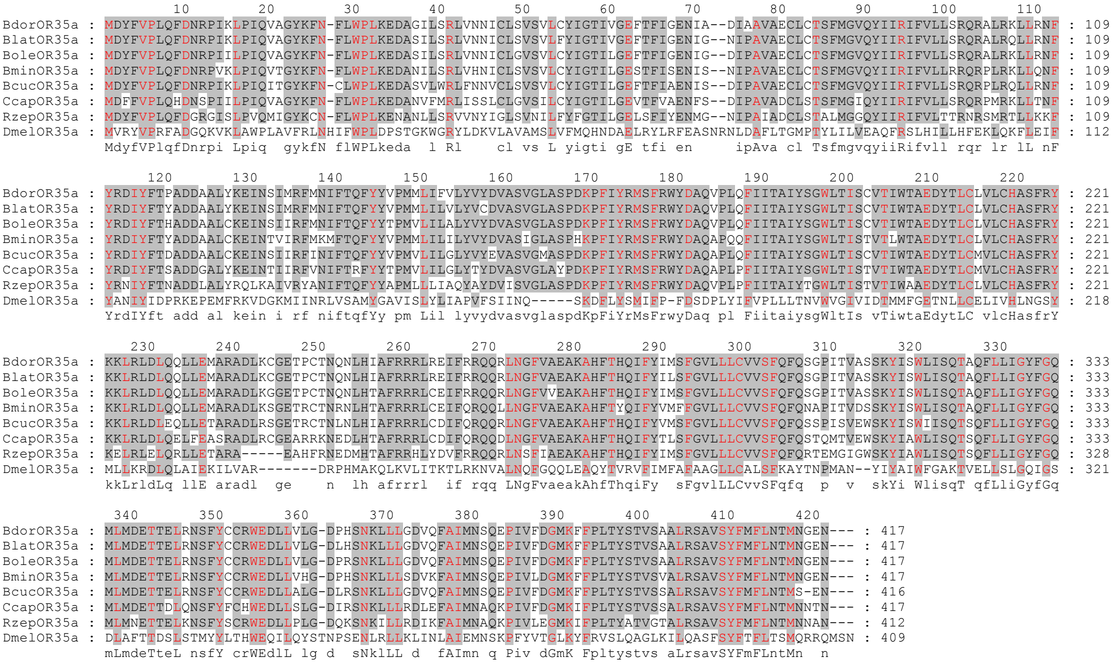

Figure S6-8 The alignment of OR35a

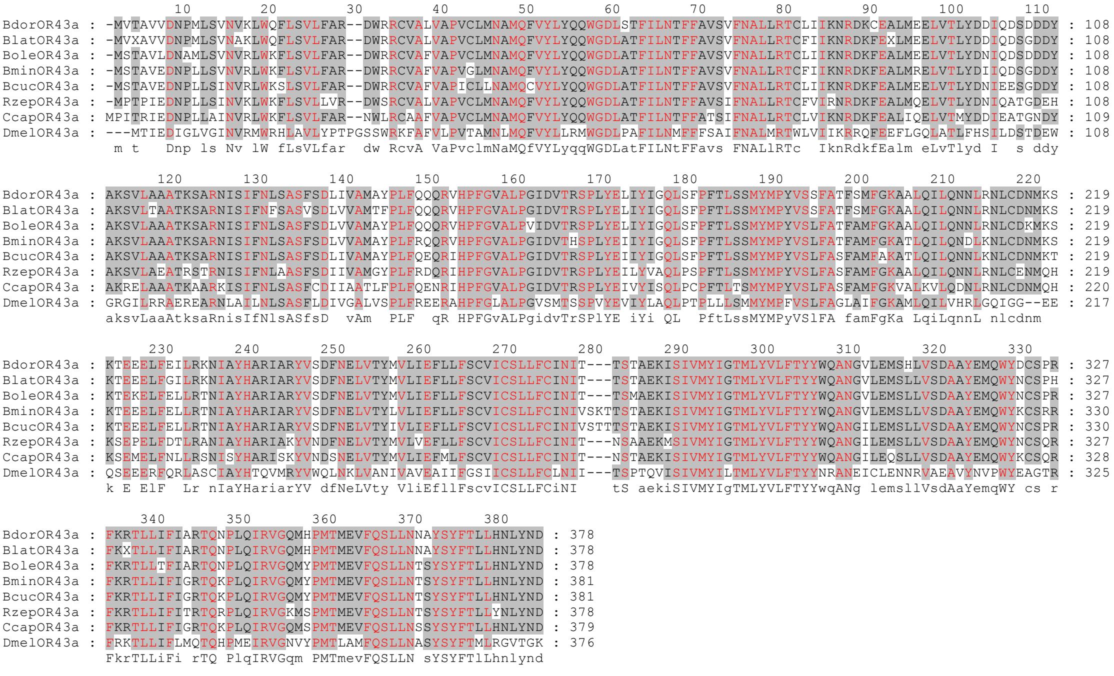

Figure S6-9 The alignment of OR43a

```
                       10        20        30        40        50        60        70        80        90       100       110
BdorOR45a2   : ----------------------------------------------------------------------------------MENFADVDKIVAAMTINVQLFTTSGKNF :  28
BlatOR45a2   : -------------------------------------------------------------------------------------------------------------- :   -
BcucOR45a2   : --------------------------MRITILKDIKPITDAFVRIGLSIELGGSKGIFSHPERYATVMIGVIVWAVALFTYTMEYLADVDKIVAAMTINVQLCLTTSKNF :  84
CcapOR45a2   : --------------------------MRIIVLKDITPVVDAFACIGFNIDLNKAKGSFSQPVRYFVLLIGVIAWTAALAAYTMQYLTDVDKMVAAMTINVQLFLTTSKNL :  84
BoleOR45a2   : ----------------------------------------------------------------------------------MENLTDVDKILAAVTINSQLFTSSSKHF :  28
RzepOR45a2   : -------------------------------------------------------------------------------------------------------------- :   -
BminOR45a3   : ------------------------------MYKYLRIPYFSFRVIGINLWAQQDQRITSAPCRYYSWTLATAIIIILMGFY--IYTSDQDKAIQVLTVFLQGVLCIIKSG :  78
BoleOR45a3   : ------------------------------MYEYLQIQHFSFRVIGINLWAQRDQRITSAPCRYLTWTLATAIITLLMGCY--IYTSEQDKAIQVLTVFLQGVLSVIKSG :  78
BdorOR45a3   : ------------------------------MYEYLRIQQFSFRVIGINLWAQRDQRIASAPCRYYSWTLATAIITLFMGFY--IYTSEQDKAIQVLTVFLQGVLSVFKSG :  78
RzepOR45a3-1 : ------------------------------MHKYLRIQQISFLLLGIDMFATRDRHILRAPYRYGCWTLATATITILMGVY--IYNSEQDQAVKVLTVFLQGVLSVLKSF :  78
RzepOR45a3-2 : -------------------------------------------------------------------------------------------------------------- :   -
CcapOR45a3   : -------------------------------MDGLGIGGDGNS--GIRAGEIGEKTDANSDSDVGANVTSSTMDNALMQ--------KPDQAITVLTVFLQGVLSVFKSG :  69
BdorOR45a1   : --------------------------------------------------MTTRSARVIKIYFLTLQIIALATILIPIAVYSWQHIQEIVEVTNAMAPFMQATISLWKIW :  60
BlatOR45a1   : -----------------------------MVMVFAQSCTSKTRQLPHLTDMTTRSARVTKIYFLTLQIIALATILIPIAVYSWQHIQEIVEVTNAMAPFMQATISLWKIW :  81
BoleOR45a1   : --------------------------------------------------MTNRSARVTQIYFLTLQIIALSTILIPFAVYSWQHMQEIVEVTNAMAPFMQATISLWKIW :  60
BminOR45a1   : -------------------------MFKSGLNIKGYFHLQKFTFHRLGIDITTRSAHVTRIYLLILQIVALATILISMASYSWQHIQDIGEVTNAMTPFMQATITLWKIW :  85
BcucOR45a1   : --------------------------------------------------MTTREARVTQICFLIIQIFALASILIPIAVYSWQHIDDIAEVSNAMAPFMQATISLWKVW :  60
CcapOR45a1   : MLVHSHYKSHSICSLCVRRSTGSQKMFHSDLVINGYFQLQKHTFQRLGIDMTTRNASITHIYFLALQIVALVTITMPMVIYSCQHLQEIAEVTNAMAPFMQATITLWKIW : 110
DmelOR45a    : -------------------------------MDASYFAVQRRALEIVGFDPSTPQLSLKHPIWAGILILSLISHNWPMVVYALQDLSDLTRLTDNFAVFMQGSQSTFKFL :  79
                                                                                                                   q      k

                      120       130       140       150       160       170       180       190       200       210       220
BdorOR45a2   : IFLARRKRFLRLNEALERLALKGNKYERELWNATNRPVLP-------------ITTAYSISCQLTVNICLLLPIFKLLFYYIWYNEVVLTLPLPGIFPYDYTLPFYYILT : 125
BlatOR45a2   : -------------------------------------------------------------------------------------------------------------- :   -
BcucOR45a2   : IFLARRERFLRLNEALERLALTGNNIERELWNTSNRRVLP-------------ITMAYSISCQMTVSICVLLPILKLLYYYIWHNEVVLTLPLPGIFPYDYTVPFYFILT : 181
CcapOR45a2   : IFLARRKRFLHLNEALERLALNGNESERILWNTTNRFVLP-------------ITRAYRISSELTVSFCVLLPILKLLYYYIFHSEVVLTLPLPGIFPYNYTLPFYFILT : 181
BoleOR45a2   : IFLARRKRLLRLNEALERLALTGNKFERDLWNTTNRTVLP-------------IVTAYSISCQLTVNICVLLPILKLLYYYIWYNEVVLTLPLPGSFPYDYTSPFYFILT : 125
RzepOR45a2   : ------------------------------------------------------------------------------------------------FPYDYTIPFYYILT :  14
BminOR45a3   : MFVAKGRRFIKLIRNLDALADAATVKEWSEWRHENDWQQC-------------IVRVYYICCTSTGTLYCTVPAVILLYSQCFREHTVFILPFDAAFPLDTGHLFFYTVS : 175
BoleOR45a3   : MFVAKGRRFIKLIRSLDMLADEANVKEGREWRHENDWQQR-------------IVRVYYICCMSTGTLYCTVPAIILLYSRCFNDHTMFILPFNAAFPYDTGHPFFYTVS : 175
BdorOR45a3   : MFVAKGRRFIKLIRSLDMLAAEANVKEGKEWKHENDWQQR-------------IARVYYSCCMSTGTLYCAVPAIILLYSQCFNGHATFILPFDAAFPFDTAHPFFYPIS : 175
RzepOR45a3-1 : MFVTKGRRFIALIENLESLAEGANVKEHAEWTHENDWQQRXAEWTHENDWQQRIARVYYSCCTATGTLYCTVPVLILLYSVCFNEHAVLILPFDAAFPFNVQQPFFYIIA : 188
RzepOR45a3-2 : ------------------------------------------------------------------------------------------------FPFNVQQPFFYIIA :  14
CcapOR45a3   : MFVLKSGRFIELIRNLDMLAE----KEHSAWRLENDWQQR-------------IVSVYYFCCTSTGILYCTVPALVLLYTRYFREQTVFILPFEASFPYDTNQPLFYILS : 162
BdorOR45a1   : RVIYRRKEMAQMAENIYLISTRASAK--ELTHLIQENNRE-----------RLMNTAYYYSVLNTGMLALAAPVLVSFIQYLRLGEFSYIVVLKATYPIEYARPLNYFLI : 157
BlatOR45a1   : LVIYRRKEMAQMVENIYLISYKASAK--ELTHLIQENNRE-----------RLMNTAYYYSVLNTGMLALAAPVLVSFIQYLRLGEFSYIVVLKATYPIDYARPLNYFLI : 178
BoleOR45a1   : RVIYRRKQMAEMVENIYMISAKASTK--ELTHLIQENNRE-----------RLMNTAYYYSVLITGMLALTAPVLVSFIQYLRFGEFSYIVVLKGTYPIDYARPLNYFLI : 157
BminOR45a1   : LVIYRRREMAEIVENIYLISAKASTK--ELTHLIQENNRE-----------RLMNTAYYYSVLNTGMLALTAPVLVSFIQYLRLGEFSYILVLKATYPIDYARPLNYFLI : 182
BcucOR45a1   : RVIYRRKEMAELCENIYLISAKASKL--ELAHLIQENNRE-----------RIMNTAYYYSVLNTGVMALTAPVVVSFIQYLRLGEFSYITALKATYPIDYARPLNYFLI : 157
CcapOR45a1   : RVIYRRKMMAELVEDIYSVSTKATKQ--ELTHLRRENNRE-----------RIMNTFYYYSVFNTGVLALIAPVLVSFIQYLRLGEFSYIVVLKATYPIELARPLNYVLI : 207
DmelOR45a    : VMMAKRRRIGSLIHRLHKLNQAASATPNHLEKIERENQLD-----------RYVARSFRNAAYGVICASAIAPMLLGLWGYVETGVFTPTTPMEFNFWLDERKPHFYWPI : 178
                                                                        y      t       p                        p     p  y

                      230       240       250       260       270       280       290       300       310       320       330
BdorOR45a2   : TILTVLLVQLCANTITVVDGLFGWFVYNISAHLQIMRLKLEQLLQLHVDDPN---FHRDFVALINYHREIINLALELDAVYAPIIFLESRLXSLPICFLAYQLSYLSDPA : 232
BlatOR45a2   : -----------------VDGLFGWFVYNISAHLQIMRLKLEQLLQLHVDDPS---FHRDFVALINYHRETIDLALELDAVYAPIIFLEVTSSCLPICFLAYQLSYLSDPA :  90
BcucOR45a2   : TILSVLLVYFCVYTICAVDGLFGWFVYNISAHLQIMRLKLEQLLQLHVDDPN---FQRDLVALVNYHRQIIDLSLELDALYAPIIFLEVTSSSLPICFLAYQLSYLSDPA : 288
CcapOR45a2   : TILTILLVYLCAYTIVAIDGLFGWFIYNISAHLQIMSLRLEQILQLPIEDPR---FHRHFVDLVNYHKEIIRLSLELDAVYAPIIFLEVTSSSLPICFLAYQLSYLSDPA : 288
BoleOR45a2   : LILTVLLVQFCSNSIIIVDGLFGWFVYNISAHLQIMRLKLEQLLQLHVEDPN---FNRDFVAFVNYHREIIDLTLELDAVYAPIIFLEVTSSSLPICFFAYQLSYLSDPA : 232
RzepOR45a2   : VILSVLLVFFCMNAICIVDGLFGWFVYNISAHLQIMRLKLEHLVQLQMADPQ---FQADFAAFISYHREIISLTLELDAVYAPIIFLEVTLSSLPICFLAYQFTYITDPA : 121
BminOR45a3   : YLWCISFIIYAIHAITAMDSLFCWFIFNISAHFRTLQHELQNVAKTSADVEDSACLHSKITETLHYHRRIIELSAEFDELYAPIVFIEISVSYLKLCFSAYNLINLGDIS : 285
BoleOR45a3   : YIWCISFIVYAIHAIAAMDSLFCWFIFNISAHFRALQRELETVVAASAGAEDDSSLHSRITQTLHYHRRIMELSAEFDELYAPIVFIEISVSYLKLCFSAYNLINLSDIS : 285
BdorOR45a3   : YIWCISFIIYAVHAIAAMDSLFCWFIFNISAHFRALQRAVETVGAAMTGAEDYASLHGRITRTLHYHRRIIELSAEFDELYAPIVFIEISVSYLKLCFSAYNLINLAAAA : 285
RzepOR45a3-1 : YIWCISFIVYAIHAVAAMDSLFCWFIFNISAHFRILQHKLEAVAAT-LAEEDYVSFQQNITEALRYHRHIIELAAEFDDLYAPIVFIEIAVSYLKLCFSAYNLINLADIS : 297
RzepOR45a3-2 : YIWCISFIVYAIHAVAAMDSLFCWFIFNISAHFRILQHKLEAVAAT-LAEEDYVSFQQNITEALRYHRHIIELAAEFDDLYAPIVFIEIAVSYLKLCFSAYNLINLADIS : 123
CcapOR45a3   : YIWCISFIIYAIHAIVAMDSLFCWFIFNISAHFRILQQKLTDVSVSPNASTDHAIFQQDITQTLFYHQRIIELSAEFDELYAPIVFVEISVSYLKLCFSAYNLINLDDIS : 272
BdorOR45a1   : WLWTAVAIYGVIYGSVSVDSLYSWYIHNLVGNFKILQSKLVTAESASE----LSERRELIYYCIAYHQRIIAMTEQLNIIYQPIVFVQFSLNALQICFLAYQIGSGVVDT : 263
BlatOR45a1   : WLWTAVAIYGVTYGSVSVDSLYSWYIHNLVGNFKILQSKVLTAELANE----PSQRCELIYYCIAYHQRIIAMTEQLNIIYQPIVFVQFSLNALQICFLAYQIGSGVVDT : 284
BoleOR45a1   : WLWSAAAIYGVIYGSVSVDSLYSWYIHNLVGNFKILQSKLVTAEWATD----LSERREEIYYCIAYHQRIIAMTEQFNIIYQPIVFVQFSLNALQICFLAYQIGSGVVDT : 263
BminOR45a1   : WLWSAVSIYGVIYGSVSVDSLYSWYIHNLVGNFKILQSKLVSAESTAD----LSERRTHIYYCIAYHQRIIAMTEQLNLIYQPIVFVQFSLNALQICFLAYQIGSGVVAT : 288
BcucOR45a1   : WLWTAVAIYGVIYVSVPVDSLYSWYIHNLVGNFKILQSKLVSAESIAESTADLGKRRELIYYCVAYHQRLITMSEQLNIIYQPIVFVQFSLNGLQICFLAYQIGSGVVAM : 267
CcapOR45a1   : WLWSAIAIYGVIYGSVSVDSLYSWYIHNLVGNFKILQTKFVTAELIAE----VSERRASIYYCLGYHQRLITMSEQLNIIYQPIVLVQFSLNALQICFLAYQIGSGDVAV : 313
DmelOR45a    : YVWGVLGVAAAAWLAIATDTLFSWLTHNVVIQFQLLELVLEEKDLNGG--------DSRLTGFVSRHRIALDLAKELSSIFGEIVFVKYMLSYLQLCMLAFRFSRS-GWS : 279
                                 D L  W   N      i    l                         yH  ii         y pI f       L  Cf Ay

                      340       350       360       370       380       390       400       410       420       430
BdorOR45a2   : N-VPFICLLLSSIVLQLMIYCFGGEKVQNECDELSQNIYLLIP-WHKLPPKHCRLLIPFIRSQRVLVLTGYFFTANRSLLVWIFRTAGSFTAMLFALKEKEV--- : 333
BlatOR45a2   : N-VPFICLLLSSIVIQLMIYCFGGEKVQSECDELSENIYLLIP-WQNLPPKHCRLLIPFIRSQRVLVLTGYFFTANRSLLVWIFRTAGSFTAMLFALKEKEL--- : 191
BcucOR45a2   : S-VPFMCLLMSSIVIQLMIYCFGGEKVQNECDQLCENIYLLIP-WHKLPPKHCRLLNPFIRSQRVLVLTGYFFTANRSLLVWIFRTAGSFTALLFALKEKEV--- : 389
CcapOR45a2   : N-VPFMCLLMSSIVIQLMIYCFGGEKVQSECDQLCENIYLLIP-WQNLPQKHCRLLNPLIRSQRVLVLTGYFFTANRSLLVWIFRTAGSFTAMLFALKEKDV--- : 389
BoleOR45a2   : N-VPFICLLLASIVLQLMIYCFGGEKVKNECEQLSENIYLLIP-WQNLPPKYCRLLTPFIHSQRVLVLTGYFFTANRSLLVWIFKTAGSITAMLFAWKEKKVV-- : 334
RzepOR45a2   : N-VPFMCLLLSSIVLQLLIYCFGGEKVQNECEKLCESIYLLIP-WHKLPPKHSRLYLTPLIRSQRALVLTGYFFTANRSLLVW---------------------- : 202
BminOR45a3   : G-LPVIAVGLVTITFQLCNYCFSGEKLKSVSKVVSDRIYLAFS-WERVPPPLRRLLILPLMRAQRATNLTGFLFVVDHSLLVWIFKTTGSIIGFLSATKSENTTS- : 388
BoleOR45a3   : G-LPVLAVGLVTITFQLCIYCFSGEKIKNVSEEVSDRIYLAFP-WERVPPSVRRLLLLPLMRAQRATNLTGFLFIVDHSLLVWIFKTTGSIIGFLSATKSENTTS- : 388
BdorOR45a3   : A-NTFQTSPSVS---QATDYLKSLNLLNISCVAASECIGAAKRNLQQVAEPIK------LNANARHLAITGQSAVLACE-------------------------- : 354
RzepOR45a3-1 : S-FPVIVVGLVTITFQLCIYCFSGEKIKNASLLISDHIYLTFP-WARMPSSMRRLLLLPLMRAQRSTNLTGYLFIVDHSLLVWIFKTTGSIIGFLSATKEKTVDVN : 401
RzepOR45a3-2 : S-FPVIVVGLVTITFQLCIYCFSGEKIKNASLLISDHIYLTFP-WARMPSSMRRLLLLPLMRAQRSTNLTGYLFIVDHSLLVWIFKTTGSIIGFLSATKEKTVDVN : 227
CcapOR45a3   : S-LPVIAVGLVTITFQLCIYCFSGEKIKNASEQFANHIYLSFP-WERVPPSLRRLLFVPIMRAQRPMHLTGFLFIVDHSLLVWIFKTTGSIIGFLSATKKENTNI- : 375
BdorOR45a1   : VDLPFLFLFMISVGIQLMIYCYGGQHLQNESVNVSKSIYQTIN-SSSWPNELRKVLLISMMRAQKPSKLTGIFFDVDLPLFLWVWRTAGSYVTLLRSVDQKTM--- : 365
BlatOR45a1   : MDLPFLFLFMISVGIQLMIYCYGGQHLQNESVNVSKSIYQTIN-SATWPNKLRKVLLISMMRAQKPSKLTGIFFDVDLPLFLWVWRTAGSYVTLLRSMDQKTM--- : 386
BoleOR45a1   : VDLPFLFLFMISVGIQLMIYCYGGQHLQNESVNVSKSIYQTIN-SSSWPNELRKVLLISMMRAQKPSKLTGIFFDVDLPLFLWVWRTAGSYVTLLRSVEQKTM--- : 365
BminOR45a1   : VDLPFLFLFMISVGIQLMIYSYGGQHLQNESVNVSKSIYQTIN-SSAWPIDLRKVLLISMMRAQKPSKLTGIFFDVDLPLFLWVWRTAGSYVTLLRSVDQKNL--- : 390
BcucOR45a1   : VDLPFLLLFLISVGIQLMIYCYGGQHLQNESVNVSKSIYQTIN-SSPWPNELRKVLLISLMRAQKPCKLTGIFFDVDLPLFLAVWRTAGSYVTLLRSVDQKTM--- : 369
CcapOR45a1   : VDLPFLFLFMTSVGIQLMIYCYGGQYLQNESENVAKFISQTIN-STIWPIDLRKVLLFSMARAQRPCKLTGIFFDVDLRLFLWVWRTAGSYVTLLRSVDQQSS--- : 415
DmelOR45a    : AQVPFRATFLVAIIIQLSSYCYGGEYIKQQSLAIAQAVYGQIN-WPEMTPKKRRLWQMVIMRAQRPAKIFGFMFVVDLPLLLWVIRTAGSFLAMLRTFER------ : 378
                  p          Ql iYc  g              iy          p      ll    r q    ltG  f       l  w   t gs     l
```

Figure S6-10 The alignment of OR45a

```
                     10        20        30        40        50        60        70        80        90       100       110
RzepOR46a  : -----------------------------------------------------------MYFWYVAIFWIFLFDASMWIK--IISNITDLNEIIKVFYVCSMAIAVMAKFV :  50
CcapOR46a  : MITPRKASSIPIIKYTRITMEEPKKIVNCFYERQFKFFKFLGLFGLPPNYSRFCQILFKLYFWHVTVVWMLLFDISMWVK--VIGNITDLNEIVNVFYICSMAIAVMAKFV : 109
BdorOR46a  : ---------------------------MGFH------QKLLGLFGLPAHYPSLLQYLYKLYFWHVAIFWMLLFDISMWIK--IIGNISNLNEIIKVFYLCSMAIAVMAKFV :  76
BlatOR46a  : -------------------MEDAKRIVHSFYNIQLLFFKLLGLFGLSAHYPSHLEYLYKLYFWHVAIFWMLLFDISMWIK--IIGNISNLNEIIKVFYLCSMAIAVMAKFV :  90
BoleOR46a  : -------------------MDDAKGIVYSFYKVQYLFFKLLGLFDLPAHYSSFWHYMYKLYFWHVAIFWMLLFDISMWIK--IVGNISNLNEIIKVFYLCSMAIAVMAKFV :  90
BminOR46a  : ------------------------MVSPQQSTTPEIDEYLLGLFGLPKHYSSFWHYMYKLYFWHVAIFWMLIFDISMWIK--IIGNISNLNEIIKVFYLCSMAIAVMAKFM :  85
DmelOR46aB : ----------------------MVT--EDFYKYQVWYFQILGVWQLPTWA---ADHQRRFQSMRFGFILVILFIMLLLFSFEMLNNISQVREILKVFFMFATEISCMAKLL :  84
DmelOR46aA : ----------------------MSKGVEIFYKGQKAFLNILSLWPQIERR---WRIIHQVNYVHVIVFWVLLFDLLLVL--HVMANLSYMSEVVKAIFILATSAGHTTKLL :  84
                                          f          lgl  l              yfwhv  fw llFd smw k     Nis lnEi kvfy csmaiavmaKf

                    120       130       140       150       160       170       180       190       200       210       220
RzepOR46a  : HIRIKTSSFLALFARMHDDDLLPVNKFEMETFTQSLQLSCKVRNCYMYLSLTSLSLVFVRQLVSDPGELPLSIYIPMNVEN-IWFFILAYLFQFIGASLCCFINISFDSLS : 160
CcapOR46a  : HIRKKNSRYVAFFARMHNDDLLPANPSELKKFIKSVHLSCAVRNCYMGLSLTSLALVFVPKLISDPGELPLSIYIPLNVEH-TLCFLVAYIFQFVGLSLCCFLNIAFDSLS : 219
BdorOR46a  : RIRLKNSSYVALFARIHDEDLLPVNVSELEKFTQSSHLSCRVRNSYMYLSLTSLSLIFVTQLISEPGELPLSIYVPISVEN-FWCYLIAYLFQFIGLSLCCLLNISFDSLS : 186
BlatOR46a  : RIRLKNRSYVALFARIQDEDLLPVNVSELEKFTQSSHLSCRVRNSYIYLSLTSLSLIFVTQLISEPGELPLSIYIPISVEN-FWCYLIAYMFQFIGLSLCCLLNISFDSLS : 200
BoleOR46a  : RIRLKNSSYVALFARIHDDDLLPVNVSELEKYTQSSHLSCRVRNSYTYLSLTSLSLIFITQIISEPSELPLSIYIPISVEN-FWCYLIAYLFQFIGLSLCCLLNISFDSLS : 200
BminOR46a  : RIHLKNNSYVEIFARMHDDDLLPVNVSELEKLAQSSHLSCRVRNYYTYLSLTSLTLIFVTKLISEPDELPLSIYIPISVES-FWRYLIAYLFQFIGLSLCCLLNISFDSLS : 195
DmelOR46aB : HLKLKSRKLAGLVDAMLSPEFGVKSEQEMQMLELDRVAVVRMRNSYGIMSLGAASLILIVPCFDNFGELPLAML-EVCSIEGWICYWSQYLFHSICLLPTCVLNITYDSVA : 194
DmelOR46aA : SIKANNVQMEELFRRLDNEEFRPRGANEELIFAACERSRKLRDFYGALSFAALSMILIPQFALDWSHLPLKTYNPLGENTGSPAYWLLYCYQCLALSVSCITNIGFDSLC : 195
              i  kn     lfar    dllp n  E      s  lsc vRn Y  lSltslslif     s p eLPLsiy p  ve      y  aY fqfiglslcC lNI fDSls

                  230       240       250       260       270       280       290       300       310       320       330
RzepOR46a  : ASFFIYLRGQLNILSNRLENIPTDKDTTQD-VINMQLKDCIRYYNKLLDITEIMEDLLSTPMSVQVISSVFVLVANFYAMTFLTDPN-DYATFMKFLVYQLCMLTQIFILC : 269
CcapOR46a  : ASFFIYLKGQLDILSNRLENIGKFQNITQD-VITLQLKECIRYYAKLRYITDIMEDLLCIPMSVQVISSVLVLVANFYAMTFLTDPS-DYGTFMKFLVYQLCMLSQIFMLC : 328
BdorOR46a  : ASFFIYLKGQLDILANRLENIGKDLYVDDN-IINLQLRDCIQHYVKLRKIAEIMEDLLSIPMSVQMISSVLVLVANFYAMTFLTDPS-DYVTFIKFLVYQLCMLSQIFMLC : 295
BlatOR46a  : ASFFIYLKGQLDILANRLENIGKDLYVDDS-IINLQLRDCIQHYVKLRKIAEIMEDLLSIPMSVQMISSVLVLVANFYAMTFLTDPS-DYVTFTKFLVYQLCMLFQIFMLC : 309
BoleOR46a  : SSFFIYLKGQLDILANRLENIGTNLYVDDN-MINLQLRDCIQQYVKLRNITEIMEELLSIPMSVQMISSVLVLVANFYAMTFLTDPS-DYVTFIKFLVYQLCMLAQIFMLC : 309
BminOR46a  : ASFFIYLKGQLDILAYRLENIGTNLDVDDN-MINLQLRYCIQHYVKVRKITENMEDLLSIPMFVQMISSVLVLVANFYAMTFLTDTS-DYVTFIKFLVYQLSMLSQIFMLC : 304
DmelOR46aB : YSLLCFLKVQLQMLVLRLEKLGPVIEPQDNEKIAMELRECAAYYNRIVRFKDLVELFIKGPGSVQLMCSVLVLVSNLYDMSTMSIANGDAIFMLKTCIYQLVMLWQIFIIC : 305
DmelOR46aA : SSLFIFLKCQLDILAVRLDKIGRLITTSGG-TVEQQLKENIRYHMTIVELSKTVERLLCKPISVQIFCSVLVLTANFYAIAVLSDER---LELFKYVTYQACMLIQIFILC : 302
              SffiyLkgQLdiL  RLenig          i  qL  ci  y k   i   mE ll  PmsVQ  isSVlVLvaNfYamtfltd   dy tf KflvYQlcML QIF lC

                340       350       360       370       380       390       400       410       420       430       440
RzepOR46a  : YFANEVSIRSSELPFSLYSSEWTHCNRINRRLMLLMMAQFDIPIRIKTINRCYSFNLSAFTS---------------------------------------------- : 331
CcapOR46a  : YFANEVSLRSAQLSYALYSSEWTHCNQINRRLMMLMMAQFDVPIRIKTINRCYSFNLPAFTSIINSSYSYYALLKNMKD----------------------------- : 407
BdorOR46a  : YFANEVSLRSAELSYSLYSSEWTRCSKINRRLMLLMMAQMDVPIRIKTINRCYSFNLPAFTSIINSSYSYYALLKKMKD----------------------------- : 374
BlatOR46a  : YFANEVSLRSAELSYSLYSSEWTRCSKINQRLMLLMMAQLDVPIRIKTINRCYSFNLPAFTSIINSSYSYYALLKKMKD----------------------------- : 388
BoleOR46a  : YFANEVSLRSAELSYSLYSSEWTRCSQTNRRMMLLMMAQFDVPIRIKTINRCYSFNLPAFTSIVNSSYSYYALLKKMKD----------------------------- : 388
BminOR46a  : YLANEVSLRSAELSYSLYSSEWTRCSKMNQRLMLLMMAQFGVPIRIKTVNRCYSFNLPAFTSKKKPKESSSKEQEDSTEHQSRNVINSTELETRKICSSDLTGIEFVK : 412
DmelOR46aB : YASNEVTVQSSRLCHSIYSSQWTGWNRANRRIVLLMMQRFNSPMLLSTFNPTFAFSLEAFGSIVNCSYSYFALLKRVNS----------------------------- : 384
DmelOR46aA : YYAGEVTQRSLDLPHELYKTSWVDWDYRSRRIALLFMQRLHSTLRIRTLNPSLGFDLMLFSSIVNCSYSYFALLKRVNS----------------------------- : 381
             Y anEVs rS  L  slYsseWt c   nrR mlLmMaq   pirikT NrcysFnL aFtSi n sysy allk
```

Figure S6-11 The alignment of OR46a

```
                      10        20        30        40        50        60        70        80        90       100       110
BcucOR47bX1 : -MISLSTQATINNTISTHNSYLSNDSNTHLKHTASKLRTILAPYR-VLKEMLRSGEAVHPPHTCLFYFRAYIRLLGLWPAERAVENPLYYAFNVLIMLLFGFFT---------- : 102
BcucOR46bX2 : -MISLSTQATINNTISTHNSYLSNDSNTHLKHTASKLRTILAPYR-VLKEMLRSGEAVHPPHTCLFYFRAYIRLLGLWPAERAVENPLYYAFN--------------------- :  91
BdorOR47b   : -MISLSSKATISNTIASHNSYLTNHSYTHLKHTASKLQTILAPYR-VLKEMLRCGEAVQPPHTCLFYFRSYIRLLGLWPAKRAAENQLYYFYNLLIMVLFSFFM---------- : 102
BlatOR47b   : -MISLSSKATTSNTIASHNSYLTNHSYTHLKHTVSKLQTILAPYR-VLKEMLRRGEAVQPPHTCLYYFRSYIRLLGLWPAKRDAENQLYYFYNLLIMVLFSFFM---------- : 102
BoleOR47b   : -MISLLSKATISNTISSHNSYLINNSYTHLQHTSSKLQTILAPYR-VLKEMLRFGEAVHPPHTCLFYFRSYMRLLGLWPAARATENQLYYFYNLLLMMLFSFFM---------- : 102
BminOR47b   : -MISLSSKATIDNSISSHNSYLTNKSYTHLKHTASKLQTILAPYR-VLKEMLRYGEAVHPPHTCLFYFRAYIRLLGLWPTERTAENPLYYFYNVLIMILFSFFM---------- : 102
CcapOR47b   : MLHECSDKSTISNTIFNHQSYRRRSR----KYSSSKLETILAPIR-TLKEMLLSGDAAHPSHTCLYYVRAYIRLLGLWPSQRGVEQPMYYAYNVLIMTVFSFFVATIIADLYVA : 109
RzepOR47b   : -MIPILRESTISNTISEHN-FNLNNGNIFLQNTIGMAKTILEPYR-VAREVLRRGDDGHPSHTCLYYFRAYMRLLGLWPLQRRVEQPLYYGYNGLLMTLFGFFV---------- : 101
DmelOR47b   : -----MNDSGYQSNLSLLRVFLDEFRSVLRQESPGLIPRLAFYYVRAFLSLLCQYPNKKLASLPLYRWINLFIMCNVMTIFWTMFVALPESKNVIEMGDDLVWISG-------- : 101
               mi      ti nti  hnsyl n      l  t skl tilapyr vlkemLr g a   p htcL yfr y rllglwp   r  e   lyy  N l m   f ff

                     120       130       140       150       160       170       180       190       200       210       220       2
BcucOR47bX1 : ----------------LCLIFFKMILFRLGNADTDIIINEFDALHVKHFNESH-DSPRNRRTRQWQRSFFFGEMCFFSGFYILSLFLFAAMSLQPLLSQQILPFRCKFPFGLDD : 199
BcucOR46bX2 : ----------------LCLIFFKMILFRLGNADTDIIINEFDALHVKHFNESH-DSPRNRRTRQWQRSFFFGEMCFFSGFYILSLFLFAAMSLQPLLSQQILPFRCKFPFGLDD : 188
BdorOR47b   : ----------------LYLIFFKMILFRMGNVDTDIIINEFDALHIKHARGLS-GGPRNRRILQWQRSFFFGEMCFFSGFYILSLLLFAAMSLQPLLSQQTLPFRCKFPFGLND : 199
BlatOR47b   : ----------------LYLIFFKMILFRMGNVDTDIIINEFDALHIKHGRGLS-ESPRNRRILQWRRSFFFGEICFFSGFYILSLLLFAAMSLQPLLSQQTLPFRCKFPFGLND : 199
BoleOR47b   : ----------------LYLIFFKMILFRMGNIDTDIIINEFEALHIKHASGLR-DGPRNRRILQWQRSFFIGDMCLFSGFYILSLFLFAAMSLQPLLSQQTLPFRCKFPFGLNN : 199
BminOR47b   : ----------------LYLIFFKMALFRMRNGETDIIISEFDALHVQHANLLR-DGPRNQRILQWQRRFFFGEICFFSGFYILSLFLFAAMSLQPLLSQQTLPFRCKFPFGLDD : 199
CcapOR47b   : SSDFVLLGEDLVVALGLYLILFKMILFRMSTADVDVIVDEFDALHMKFARDTS-DSPHIRRIRQLQRSFFLGEASFFCGFFFLSLFLFAAMSLQPLLTHQALPFRCVFPFGLHD : 222
RzepOR47b   : ----------------LYLILFKMFLYRSSIAEIDSIVNEFDTLHAKYAHALLPKSQSNRRIRQWQRNFFIGEIVLTTGFFILSFLLFAAMSLQPLFSHQTLPFRSKYPFGLDN : 199
DmelOR47b   : ----------------MALVFTKIFYMHLRCDEIDELISDFEYYNRELR-----PHNIDEEVLGWQRLCYVIESGLYINCFCLVNFFSAAIFLQPLLGEGKLPFHSVYPFQWHR : 194
                              l LiffKm lfr      D ii eFdalh k          p nrr  qwqR ff ge    f gf iLsl lfAAmsLQPLls q LPFrckfPFgl

                30       240       250       260       270       280       290       300       310       320       330       340
BcucOR47bX1 : PDEHPMGFVCVYFFQCFCTLYMLVAIVVMDSLGGNSFNQTTLNLRILCENMRNLGN---------GSTSELVVWRKLKETVEFHQQIIKLMNRINQTFYWNYVSQMGASTFMIC : 304
BcucOR46bX2 : PDEHPMGFVCVYFFQCFCTLYMLVAIVVMDSLGGNSFNQTTLNLRILCENMRNLGN---------GSTSELVVWRKLKETVEFHQQIIKLMNRINQTFYWNYVSQMGASTFMIC : 293
BdorOR47b   : PDEHPIAFVCVYFFQCFCTLYMLVAIVVMDSLGGNSFNQTTLNLRILCENIRHLGIVAAG---ASSSTSEAVAWRELREAVEFHQKIIGLMNRINQTFYWNYVSQMGASTFMIC : 310
BlatOR47b   : PDEHPIAFVCVYFFQCFCTLYMLVAIVVMDSLGGNSFNQTTLNLRILCENIRHLGIVAAG---ASSSTSEAVAWRELREAVLFHQKIIGLMNRINQTFYWNYVSQMGASTFMIC : 310
BoleOR47b   : PDEHPMAFVCVYFFQCFCTLYMLVAVVVMDSLGGNSFNQTTLNLRILCENIRHLGNGATG---DCSSTSEVVVWRELRETVEFHQKIIGIMNRINQTFYWNYVSQMGASTFMIC : 310
BminOR47b   : PDEHPMAFACVYFFQCFCTLYMLVAIVVMDSLGGNSFNQTTLNLRILCENIRHLGESAAGTAGACSITSELVVWRELREAVEFHQKIIGLMNRINQTFYWNYVSQMGASTFMIC : 313
CcapOR47b   : PDKHHITFVCVYAFQCFCTLYMLVSIVVMDSLGGNSFNQTTLNLQILCESIRHIGYAGGR----STTITEAVLWRELRENVEFHVKIIELVDGINHTFYWNYVSQMGASTFMIC : 332
RzepOR47b   : RDEHPIAFACIYAFQCFCVLYMLVSIVVMDSLGGNCFNQTTLNLRILCENIRNIRG----------GATEEVIWHELRVTVEFHQQIIGLIDHLNDVFYWNYVSQMGASTFMIC : 303
DmelOR47b   : LDLHPYTFWFLYIWQSLTSQHNLMSILMVDMVGISTFLQTALNLKLLCIEIRKLGD---------MEVSDKRFHEEFCRVVRFHQHIIKLVGKANRAFNGAFNAQLMASFSLIS : 299
              pDeHp  F cvY fQcfctlymLv ivvmDslGgnsFnQTtLNLriLCeniR lg             se v wrel e VeFHq II l   iN tFywnyvsQmgAStfmIc

                       350       360       370       380       390       400       410       420       430       440       450
BcucOR47bX1 : LTAFEALLAQDK-PMVALKFQTYMFSAFMQLLYWCWMGNRTYYDSMEVATAAYEVRTWYRHSPLLQRQLIFIIKRAQKPLEFRAKPLFGFTFASFTSILSTSYSYFTLLRTMSD : 417
BcucOR46bX2 : LTAFEALLAQDK-PMVALKFQTYMFSAFMQLLYWCWMGNRTYYDSMEVATAAYEVRTWYRHSPLLQRQLIFIIKRAQKPLEFRAKPLFGFTFASFTSILSTSYSYFTLLRTMSD : 406
BdorOR47b   : LTAFEALLAQDK-PMVAMKFQTYMFSAFMQLLYWCWMGNRTYYDSMEVATAAYEIRAWYEHSPLLQRQLMFIIKRAQKPLEFRAKPLFGFTFASFTSILSTSYSYFALLRTMSD : 423
BlatOR47b   : LTAFEALLAQDK-PMVAMKFQTYMFSAFMQLLYWCWMGNRTYYDSMEVATVAYEIRAWYEHSPLLQRQLMFIIKRAQKPLEFRAKPLFGFTFASFTSILSTSYSYFALLRTMSD : 423
BoleOR47b   : LTAFEALLAQDK-PMVAMKFQTYMFSAFMQLLYWCWMGNRTYYDSMEVATAAYEVRTWYEHSPLLQRQLMFIIKRAQRPLEFRAKPLFGFTFASFTSILSTSYSYFALLRTMSD : 423
BminOR47b   : LTAFEALLAQDK-PMVAMKFQTYMFSAFMQLLYWCWMGNRTYYDSMEVATAAYDVHAWYEHSPLLQRQIMFIIKRAQKPLEFRAKPLFGFTFASFTSILSTSYSYFALLRTMSD : 426
CcapOR47b   : LTAFEALLAKDQ-PMVAMKFQTYMFSAFMQLFYWCFMGNRTYYDSMEVATAAYEVYAWYEHSPRLQRNLLFMIKRAQKPLEFRSKPFFGFTFASFNSILSTSYSYFALLRTMND : 445
RzepOR47b   : LTAFEALLAKDQ-PMVAMKFQMYMFSAFMQLFYWCSMGNRTYYESMEVATAAFQVHTWYNHSPRLQRNLMFMIKRAQRPLEFRAKPFFGFTFASFTSILSTSYSYFALLRTMSD : 416
DmelOR47b   : ISTFETMAAAAVDPKMAAKFVLLMLVAFIQLSLWCVSGTLVYTQSVEVAQAAFDINDWHTKSPGIQRDISFVILRAQKPLMYVAEPFLPFTLGTYMLVLKNCYRLLALMQESM- : 412
              ltaFEallA d  PmvA KFqtyMfsAFmQL yWC mGnrtYydSmEVAtaAy    Wy hSP lQR l F IkRAQkPLefrakP fgFTfasftsiLstsYsyfaLlrtmsd
```

Figure S6-12 The alignment of OR47b

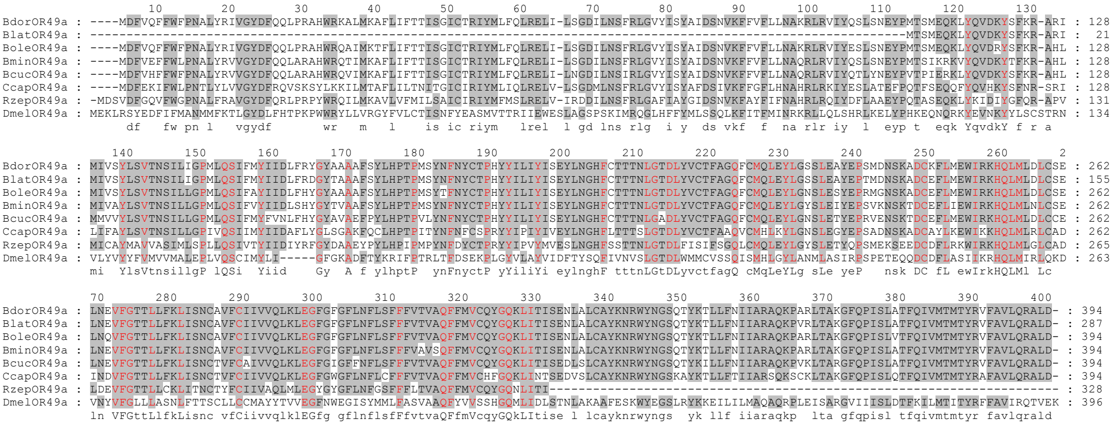

Figure S6-13 The alignment of OR49a

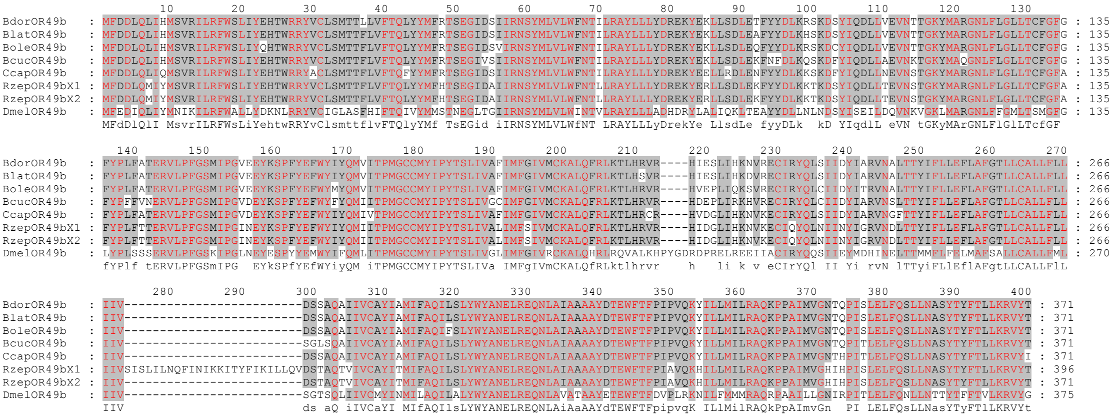

Figure S6-14 The alignment of OR49b

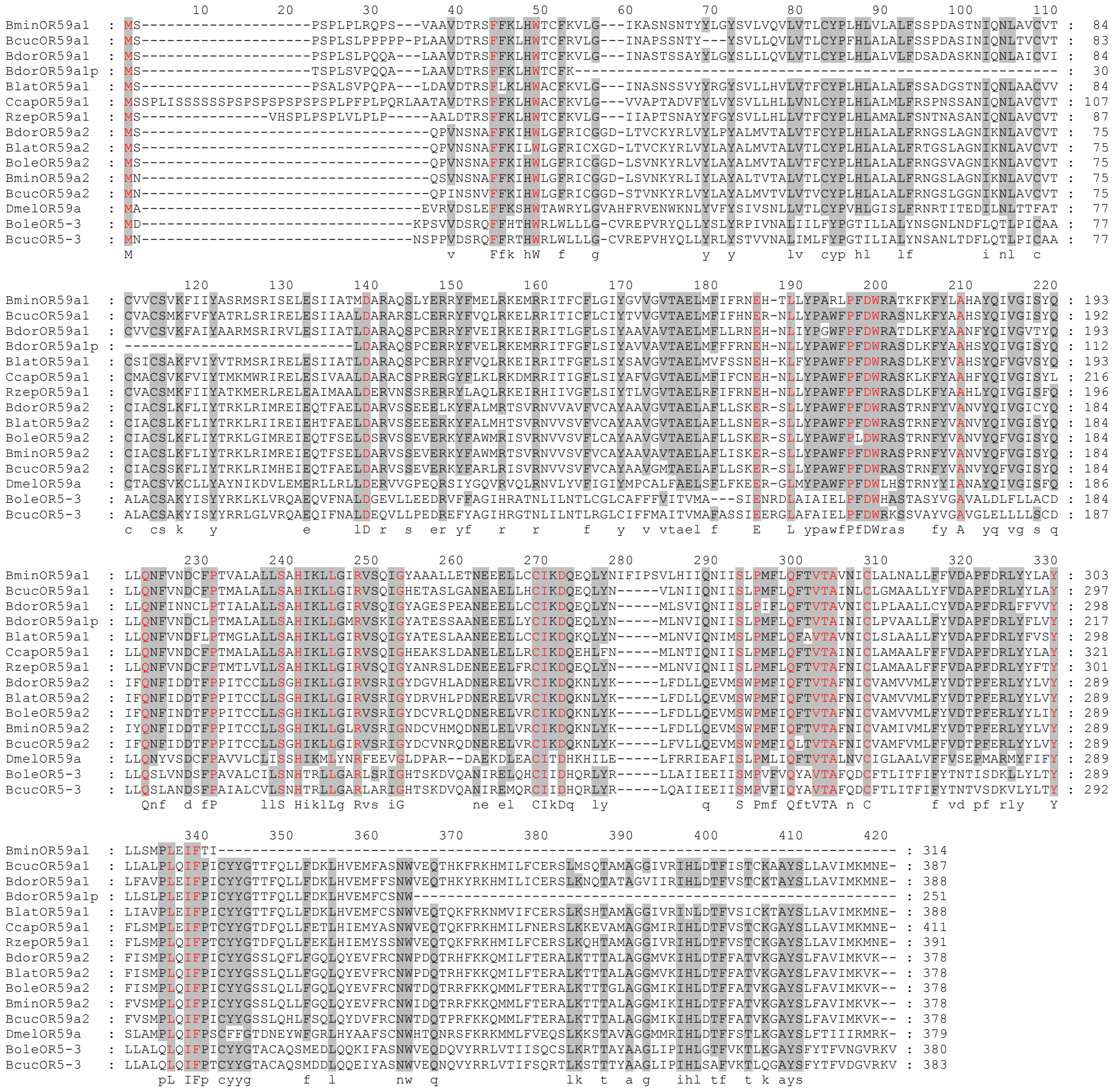

Figure S6-15 The alignment of OR59a

```
                       10        20        30        40        50        60        70        80        90       100       11
BdorOR63a1-1 : -MMSESVEEIYKRNYNSIKVLIGVSFSLGVNLTAPSKIKDALKLFNVIWVVASLLSLYAHWSYFIRHIDNIP--LLAETVCTALQTLISAVKMVYYLFTQRTFYRLLEQ : 106
BoleOR63a1-1 : --MLESVEEIYKRNYNSIKVLIGVSFGLGVNLTAPSKIKDALKLFNVILVVTSLLSLYAHWCYFLRYIDNIP--LLAETVCTALQTLISAVKMVYYLFTQRTFYRLLDQ : 105
BcucOR63a1   : -MVFEDVEEIYRRNYNSIKVLFQVSFGLGVNLTAPSKIKDALKLFNVILVVASLLSMYAHWCYLIRHFENIP--LLAETVCTALQTLISAFKMIYFLFRQHNFYRLLDQ : 106
CcapOR63a1   : -MILEEVEDVYKRNYNSIKVLIRVSFGLGVNLTAPTRFKDSLRIFNVILVTTSILSLYAHWCYLIRHFDNIP--LLAETVCTALQTLISAVKMVYFLFTQRTFYRLIDQ : 106
RzepOR63a1   : -MILDKLAEVRERNFNSIKVLFGVSYGLGVNLTAPN---------------------FPVKYQLKQEIDNIMTDIWRTTRRQILFYFSCCLAIVCNYFFAALFVNLYNQ :  87
BlatOR63a1-2 : -MILENKEELYKRNYNSIKVLFRVSYTLGVNLTAPDKFKDSLKII--------------------------P--LIAETVFTALQIGMAAIKMIYFFFTHRTFYRLLDQ :  80
BoleOR63a1-2 : -MTSENKEELHKRNYNSIKVLFRVSYSLGVNLTAPNKIKDALK-----------------------------------TVFTALQIGMAAIKMIYFFFTHRTFYRLLDQ :  73
BdorOR63a1-2 : -MTLENKEELYKRNYNSIKVLFRVSYTLGVNLTAPDKFKDSLKVIQIILIASSLLSLFAHWWYLKRHIDNIP--LIAEAVFTALQIGMAAIKLIYFFFTHRTFYRLLDQ : 106
BminOR63a1-1 : -MMMENAEEIYKRNYNYIKVLIRVSFSLGVNLTAPSKIKDALKLISVILSVTSLLSMYGHCCYLMRHLDRMP--LIAEAVFTALQILMAAIKLIYFFFTHRTFYRLLDQ : 106
BminOR63a1-2 : -MILENEEEIYKRNYNSSKVLFRVSFALGVNLTAPSKAKNTLKIVHVILIVSSILSLYAHWCYLMRHFDSIP--LIAEAVFTALQILMAAIKLIYFFFTHRTFYRLLDQ : 106
DmelOR63a    : MYSPEEAAELKRRNYRSIREMIRLSYTVGFNLLDPSRCGQVLRIWTIVLSVSSLASLYGHWQMLARYIHDIP--RIGETAGTALQFLTSIAKMWYFLFAHRQIYELLRK : 107
BdorOR63a2-1 : MYSISEIKELRTRNHWRIRELKRISYIIGINLNAQTKCKRWWRIVNILFIIASCIALYPHWLMIKQAEGDIP--LIAETSTTALQTTTGLIKMAYMLFTQHRFHRLLRK : 107
BlatOR63a2-1 : ------------------------------NLNAQTKCKRWWRIINILFIIASCIALYPHWLMIKQAQGDIP--LIAETSTTALQTTTGLIKMAYMLFTQHRFHKLLRK :  77
BoleOR63a2-1 : MYSITEIKTLRVRNHLLIRELKRISFIIGINLNAQTNLKEWWRIINILFIITSCIALYPHWLMIKQAKGDIP--LIAETSTTALQTTTGLIKMAYLLFSQHILYKLLQK : 107
BminOR63a2   : MYSIIERKELRVHNIWRIRELKRISYIIGINLNAQTKLKRSLRIINVLIIIFSCIALYPHWLMIKQAQGDIP--LIAETSTTALQTTTALIKMAYMLFNQHKFHKLLQK : 107
CcapOR63a2   : MYSQREINDLKVRNHWLIRELKRSSYLIGVNLSTQTFLKGWLKIINVLLIIASCIALYPHWLMIEHAEGNLS--LIAETSTTALQTTTALVKMAFILFKQHRLHELLYK : 107
RzepOR63a2   : MYSAGEIQALKRRNRHYIRELIWTSFSIGVNITTQTKLKDYLRIVSVLLVIASCIALYPHWLMIQQAQGHIP--LIAETVTTTLQTTTAIVKLIYYIFMQHRFSAILHK : 107
BlatOR63a2-2 : MYNAAEFAELKNSNRFKIRELRKVSYILGINYGSETSLKKCLRVLNLFLIITCGISLYPRWLMLERADGNVP--LIAETITTMLQTTTTMVKMIFCLFMQSQCRALLMK : 107
BdorOR63a2-2 : MYNAAEFAELKNNNRFKIRELRNVSYILGINYGSETSLKKFLRVLNLFLIIICAISLYPRWLMLERADGNVP--LIAETITTMLQTTTSMVKMTFCLFMQGQCRALLKK : 107
BoleOR63a2-2 : MYNTEEFVELKKYNRFKIRELKAVSYILGINYGSETSLKKFLRVLNLLLIIICGISLYPRWLMLERADGDVP--LIAETITTILQTTTSMVKMTFCLFMQGQCCALLKK : 107
                            n   i  l   s   g Nl      k  l               y  w          p  l aet  t Lq      k     F       ll

               0       120       130       140       150       160       170       180       190       200       210       2
BdorOR63a1-1 : TLTHEIIRKIEIFER--DFPINRQLKQEVDDIMNGVWRSARRQLLFYFCCCVGIVCNYFFGALFVNLYHQLKQTP-DYEHILPFPALYPIWEDQGMTFPYYPLQMYLSG : 212
BoleOR63a1-1 : TLKHEIIRKIEIFQH--DFPINRQLKQEIDDIMNGVWLSAKRQILFYFCCCVAIVCNYFFGSFFVNLYHQLKQTP-DYKHILPYPALYPFWEEKGMTFPYYPLQMYMTG : 211
BcucOR63a1   : ALKHVIIREIEIFKH--DFPINQQLKREIDEIMNGVWQNARRQILFYFCCCVGIVCNYFFGAFLVNLYHQLKKTP-DYKYVLPFPELYPFWEDKGMTFPYYPIQMFISS : 212
CcapOR63a1   : ALTHEVIRKIEIFKY-------------------------------------------------------------------AFPALYPFWEAKGMTFPYYHLQMYMTG : 148
RzepOR63a1   : ------LKQTPNYKH-----------------------------------------------------------------ILPFPALYSFWENKGMTFPYYHLEMFLSG : 125
BlatOR63a1-2 : TLTHEIIRKIEILT---DFPIDRQLRQEIDDIMNRVWRNTRHLFLFYFCCCVGIIANYFFTAFFVNLYHQLKQTP-DYEFFLPVPALYPFWEKKGMAFPYYPIQMYMTG : 185
BoleOR63a1-2 : ALTHEIIRKIEILT---RFPINRQLKQEIDDIMKRVWP------------------------FFVNLYHQLRQTP-DYEYFLPVPALYPFWEKKGMAFPYYHIQMYMTF : 154
BdorOR63a1-2 : TLTHEIIRKIEILT---DFPIDRQLRQEIDDIMNRVWLNTRRLFLFYFCCCVGIIANYFFTAFFVNLYHQLKQTP-DYEFFLPVPALYPFWEKKGMTFPYYPIQMYMTG : 211
BminOR63a1-1 : TLTHEIIRKMEILQQ--DFPINRQLKKEIDDIMNAVWRNIRRILLFYFFCCVGIIANYFFTALFQNLYHHLKQTP-NYEFILPVPSQYPFWEKKGMAFPYYHLQMYVTG : 212
BminOR63a1-2 : TLTHEIIRKIEILQQ--DFPINRQLKKEIDDIMNAVWRNIRRIFLFYFFCCVGIIANYFFTALFQNLYHHLKQTP-NYEFILPVPALYPFWEKKGMAFPYYHLQMYMTG : 212
DmelOR63a    : ARCHELLQKCELFERMSDLPVIKEIRQQVESTMNRYWASTRRQILIYLYSCICITTNYFINSFVINLYRYFTKPKGSYDIMLPLPSLYPAWEHKGLEFPYYHIQMYLET : 216
BdorOR63a2-1 : AETHELLQGIEIFQT--DMPIKTSLKKEINAVMEINWKQARGQLLFTLGTCICIMSNYFFYAFFKNLYNHLQGTP-NYVYILPFTG-YPMFLHKGMASPYYAMDMFFGA : 212
BlatOR63a2-1 : AETHELLQGIEIFQT--DMPIKTTLKKEINAIMEINWRQARGQLLFTLGTCICIMSNYFFYAFFKNLYNHLQGTP-NYVYILPFTG-YPMFLHKGMASPYYAMDMFFGA : 182
BoleOR63a2-1 : AETHELLQRIEIFQT--GMPIKTTLKKEINAIMDTNWKQTRRQLLFTLGTCICILSNYFFYALFKNLYNYLQGTP-NYVYILPFTG-YPMFLDKVMGSPYYALDMFFGA : 212
BminOR63a2   : VETHDLLQRIEIFLT--GMPIKPNLKREINAIMETNWKQTRRQLLFTIGTCICIMCNYFFYALFKNLYNHLQGTP-NYVYILPFTG-YPMFLDKGMASPYYAMDMFFGA : 212
CcapOR63a2   : AEYHELLQGIQIFMT--DMPIRISLKNEAIRIMDTTWQEARGQLLFSLISCICIQANYFFYAFFKNLYHHLQGTP-NYVYILPFTG-YPMFHHKGMSSFYYIMDMFFGA : 212
RzepOR63a2   : AETHELLQCLEIFKT--DMPVKMKLKQEINAIMVTTWLESRRQLIYCVILIVCILSNYFFYAIFINLYHQIQGTP-NYVYILPFTG-YPMFLDKGMNSFYYALDMFLGA : 212
BlatOR63a2-2 : AENYELLQGIKIFET--DMDIKAELKVEINAIMATIWKESRRQLLSCLITCSCILSNYFLYAFFTNLYHQIKRTP-NYVHILPFTG-YPMFLEKGMASPYYAVEMFIGG : 212
BdorOR63a2-2 : AENYELLQGIKIFLT--DMDIKAELKVEINAIMATIWKESRRQLLSCLITCSCILSNYFLYAFFTNLYHQIKKTP-NYVHILPFTG-YPMFLEKGMASPYYAVEMFIGG : 212
BoleOR63a2-2 : AENYELLQGMKIFST--DMCIKTELKNEINSIMETIWMESRRQLLSCLITCSCILSNYFLYAFFTNLYHQIKKTP-NYVHILPFTG-FPMFLDKGMTSPYYAVEMFIGG : 212
                  he      i        i   l  e   im   w   r   l     c  i  nyf    f nly     tp  y   lp    yp    kgm  pYY   M

               20       230       240       250       260       270       280       290       300       310       320
BdorOR63a1-1 : SAVYIAGMCAVSFEGVFIVLCQHAVGLVKVHNLLVLRSTSRLIPAERRIEYLRYTIITYQRINIFAQQIQTIFKHVSLSQFVLSLIVFGFVLFEMSFGLES----SIVI : 317
BoleOR63a1-1 : SAVYISGICAVSFEGVFIVLCQHAVGLVRVHNLLVLRSTSPLIPAERRVEYLRYTIITYQRINFYVQQIQNSFKHVSLSQFVLSLIVFGFVLFEMSFGLES----SIVI : 316
BcucOR63a1   : SAVYIAGMCAVSFDGVFIVLCQHAVGLVRVHNLLVLRSTSPLIPVERRVEYLRYTIFTYQRIYTYVQQIQTSFKHISLSQFVLSLIVFGFVLFEMSFGLVSNCESSIII : 321
CcapOR63a1   : SAVYIAGICAVSFDGVFIVLCQHAVGLVKVHNLLVLRATSPLIPPERRVDYLRYVIFTYQRVRVYVQQVQTIYKHVSLSQFVLSLIVFGFVLFEMSFGLES----SITI : 253
RzepOR63a1   : SAVYIAGMCAVSFDGVFIVLSQHAVGLIKVLNLLVLRSTSPLIPPERRIEYLRYTIITYQRTAIYVQQIQTIFKQISLSQFVFSLIIYGFVLFELSFGLES----SITI : 230
BlatOR63a1-2 : AALYVSGLGAVSFEGVFMVLCQHAVALVKVHNLLVQHATSPQIPTERRLEYLRYLIITYRRISKYLQEIKTIFRHISLVQFLLSLFVMGFVLFEISYGL----EASIVI : 290
BoleOR63a1-2 : AALYVSGLGAVSFEGVFMVLCQHAVALVKVHNLLVLHATSSQIPAERRLEYLRYLIITYRRISKYLQEIKTIFRHISLVQFLLSLIVMGFVLFEISYGL----ESSIVI : 259
BdorOR63a1-2 : AALYVSGLGAVSFEGVFMVLCQHAVALVKVHNLLVQHATSPQIPAERRLEYLRYLIITYRRVSK--------------------------------------------- : 275
BminOR63a1-1 : SALYVSGLGAVSFEGVFMVLCQHAVGLVKVHNLLVLRATSAQIPVERRLEYLRYTIFNYQRINKYMHEIQTIFRHISLSQFILSLIVIGFVLFEINYGL----GSNIII : 317
BminOR63a1-2 : SALYVSGLGAVSFEGVFMVLCQHAVGLVKVHNLLVLRATSAQIPVERRLEYLRYTIFTYQRINKYMQEIQTIFRHISLSQFLLSLIVLGFVLFEINYGLQIFKGSNIII : 321
DmelOR63a    : CSLYICGMCAVSFDGVFIVLCLHSVGLMRSLNQMVEQATSELVPPDRRVEYLRCCIYQYQRVANFATEVNNCFRHITFTQFLLSLFNWGLALFQMSVGLGN---NSSIT : 322
BdorOR63a2-1 : CSLLVAGMSAISFQGCFLVLCKHSCGLVQVLCLLLKRSTSSLVPKPQRVEYLRYCIVQHQRTLEFINEVNQPFRHICLSQFLHSLAIYGFVLFEMNFGLES----NKIT : 317
BlatOR63a2-1 : CSLLVAGMSAITFQGCFLVLCKHSCGLVQVLCLLLKRSTSTLVPKPQRVEYLRYCIVQHQRTLGFIDEVNQLFRHICLSQFLHSLAIYGFVLFEMNFGLES----NKIT : 287
BoleOR63a2-1 : CSLLVAGMSAISFQGCFMVLCKHCCGLVQVLCLMLLRSTSTLVPKSQHVEYLRYCIVQHQRTLEFINDVNRFFRHICLSQFLHSLAIYGFVLFEMNFGLES----NKVT : 317
BminOR63a2   : CSLFAAGMSALSFQGSFMVLCKHSCGLVQVVCLLLLRSTSAMVPKPQRVEYLRYCIVQHQRTLEFINDVNRLYRHICLSQFLHSLAIYGFVLFEMNFGLES----NKVT : 317
CcapOR63a2   : CSLHCAAMCAICVQCTFMVLCKHCCGLVQVQCLMLLRSTSPLVPKVRRVEYLRYCVIQHQQILRFMEGINQLFRHICLSHFLHSLAIYGFVLFEMSFGLES----NKVI : 317
RzepOR63a2   : CSLHVAGMCAISFNCAFMVFCKHACGIVRVLNLMLQHSTSPLVPAERRVEYLRYCIIQHQRIVNFLDEVNQLFKHIILSQFFHSMAIYGLVLYEINFSLES----SKVT : 317
BlatOR63a2-2 : CSLLTCGMCSVSFHCIFMILSKHACGLVKVLCAMLLQSTSPVVPAHRREEYLRYCVIQHQQTLLFINDINELFKHITLSHFLHSLAIYGLVLFEMNFGLET----NKTT : 317
BdorOR63a2-2 : CSLLTCGMCSVSFHCIFMILCKHACGLVKVLCVLLMRSTSLQVPAHRRDEYLRYCVIQHQQTLRFINDINDLFKHITLSHFLHSLAIYGLVLFEMNFGLET----DKTT : 317
BoleOR63a2-2 : SSLLTCGMCSVSFHCIFMILCKHACGLVKVLCAILLQSTSPHVPAHRRDEYLRYCVIQHQETLRFINDINELFKHITLSHFLHSLAIYGLVLFEMNFGLET----NKTT : 317
                     g  a sf   F vlc H  glv v  l     TS   P  rr eYLRy i   qr           f hi ls f  sl   g vlfe   gl

               330       340       350       360       370       380       390       400       410       420
BdorOR63a1-1 : VIRMIMYFAAGGTQIILYCYNGQQLTSVSEEIPLAFYNCNWYEESGKFKQLLRMMIMRTNRPFNLEVSSFTLMNLATLIALFRMSGSYFLLLRNLQEK-- : 415
BoleOR63a1-1 : VIRMIMYFAAGGTQIILYCYNGQQLTTVSEEIPSAYYSCNWYEESEKFKQFLRMMIMRTNRYFYMEVSSFTLMNLATLIALFRMSGSYFLLLRNLQEK-- : 414
BcucOR63a1   : FIRMIMYISAGGTQIIIYCYNGQALTSVSEEIPMAFYNCNWYEECEKFKQLLRMMIMRTHRYFYLEVSWFTLMNLATLIALFRMSGSYFLLLRNLQES-- : 419
CcapOR63a1   : FIRMIMYISAGGTQIIIYCYNGQQLTSVSEEMPLAFYSCGWYEESKKFKQLLRMMIMRTNRHFYLEVSWFTLMNLATLIALFRMSGSYFLLLRNLQES-- : 351
RzepOR63a1   : FIRMIMYIAAGGTQIVIYCYNGQQLTTVSEQIPFSFYTSKWYEESEKFKQLIRMMILRTHRNFHLEVSWFTLMNLATLIALFRMSGSYFLLLRNLQEK-- : 328
BlatOR63a1-2 : LIRMIMYISASISQITIYCYHGQALTSACEEIPLAYYNCDWYGENKTFKNLILMMIIRTNEEFNMEVSWFTLMNLTTLISLLRASGSYFLLLQNLQED-- : 388
BoleOR63a1-2 : LIRMIMYISASISQITIYCYHGQALTSACEKIPMAYYNCDWYGENKAFKNLILMMIMRTNKQFYMEVSWFTLMNLTTLISLLRASGSYFLLLQNLQED-- : 357
BdorOR63a1-2 : ---------------------------------------------------------------------------------------------------- :   -
BminOR63a1-1 : FIRLIMYISASITQITIYCYHGQALTTVNEKIPLAYYNCNWYGENKTFKQLIMMMIMRTNKEFYLEVSWFTLMNLATLISLIKASVSYYLLLQHFQEN-- : 415
BminOR63a1-2 : FIRLIMYISASITQITIYCYHGQALTTVNEKIPLAYYNCNWYGENKTFKQLIMMMIMRTNKEFYLEVSWFTLMNLATLISLIRASVSYYLLLQNFQEN-- : 419
DmelOR63a    : MIRMTMYLVAAGYQIVVYCYNGQRFATASEEIANAFYQVRWYGESREFRHLIRMMLMRTNRGFRLDVSWFMQMSLPTLMAMVRTSGQYFLLLQNVNQK-- : 420
BdorOR63a2-1 : FIRMLMYLCAATTGDCTHYVNGQFLANELEKVPLACYNCEWYHETDAFKKTLRMIIMRSNKKFCFQISWFTVMSLATLMGIFKASGSYFVLLRDIDET-- : 415
BlatOR63a2-1 : FIRTLMY--------------------------------------------------------------------------------------------- : 294
BoleOR63a2-1 : FIRMIMYLCAALTGDCMHYVNGQFLANELEKIPLACFNCEWYHETDDFKKKLKMIIMRSNKKFCFQISWFTVMSLATLMGIFKASGSYFVLLRDIDEP-- : 415
BminOR63a2   : FIRMIMYLCAALTCDCLQHVNGQFLANELEKIPLACYNCEWYHETDDFKKTLKMIIMRSNKKFCFQISWFTVMSLATLMGVS------------------ : 399
CcapOR63a2   : FVRMMMYLCAALTCDCMFYVNGQFLSTELEKIPLACYSCEWFHESREFKMILKMIIMRSNKPFYFQISWFTVMSLATLMGIFKASGSYFVLLRDIDEA-- : 415
RzepOR63a2   : LIRMIFYICAAFSGDCMLYVNGQFLVTELEAIPLSCYCCHWYNESAEFKKTLQMIIMRSNKEFCFQISWFGVLSLVTLMGIFKASASYFVLIRDIADADA : 417
BlatOR63a2-2 : FIRMVMYIGAALTVDSMYYVNGQFLATELEKIPFVCYSCDWFNESKDFKKTLNMIIMRSNKDFQFQISWFGVMSLTTLMGILKASFSYFLILRDMTDEVN : 417
BdorOR63a2-2 : FVRMLMYIGAALTVDSMYYVNGQFLATELENIPFVCYSCDWFNESEDFKRTLKMIIMRSNKDFCFQISWFGIMSLTTLMGILKASFSYFLILRDMTDETN : 417
BoleOR63a2-2 : FVRMIMYIGAALTVDSMYYVNGQFLVTELEKIPLVCYSCDWFNESEGFKKTLNMIIMRSNKDFCFQISWFGVMSLTTLMGILKASFSYFLILRDITDEIN : 417
                irm my  a           gq l    e ip   y c w  e   fk    m imr n  f    swf  m l tl      s sy   l
```

Figure S6-16 The alignment of OR63a

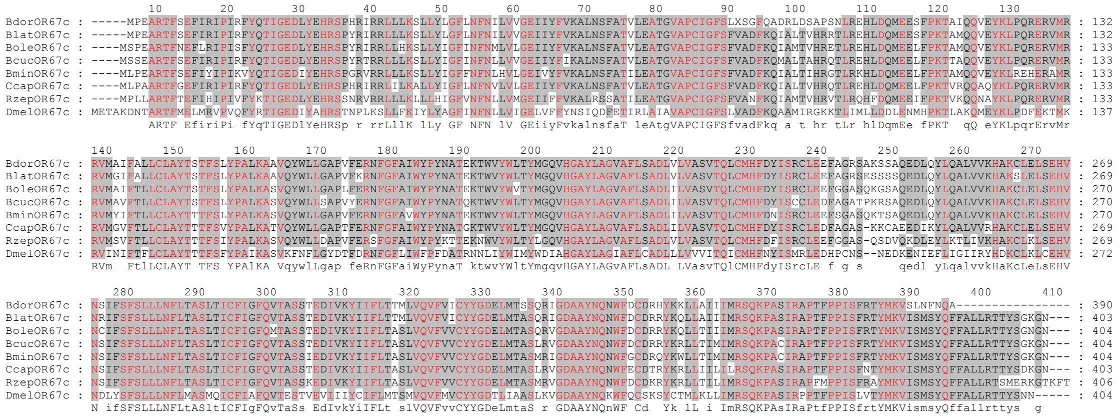

Figure S6-17 The alignment of OR67c

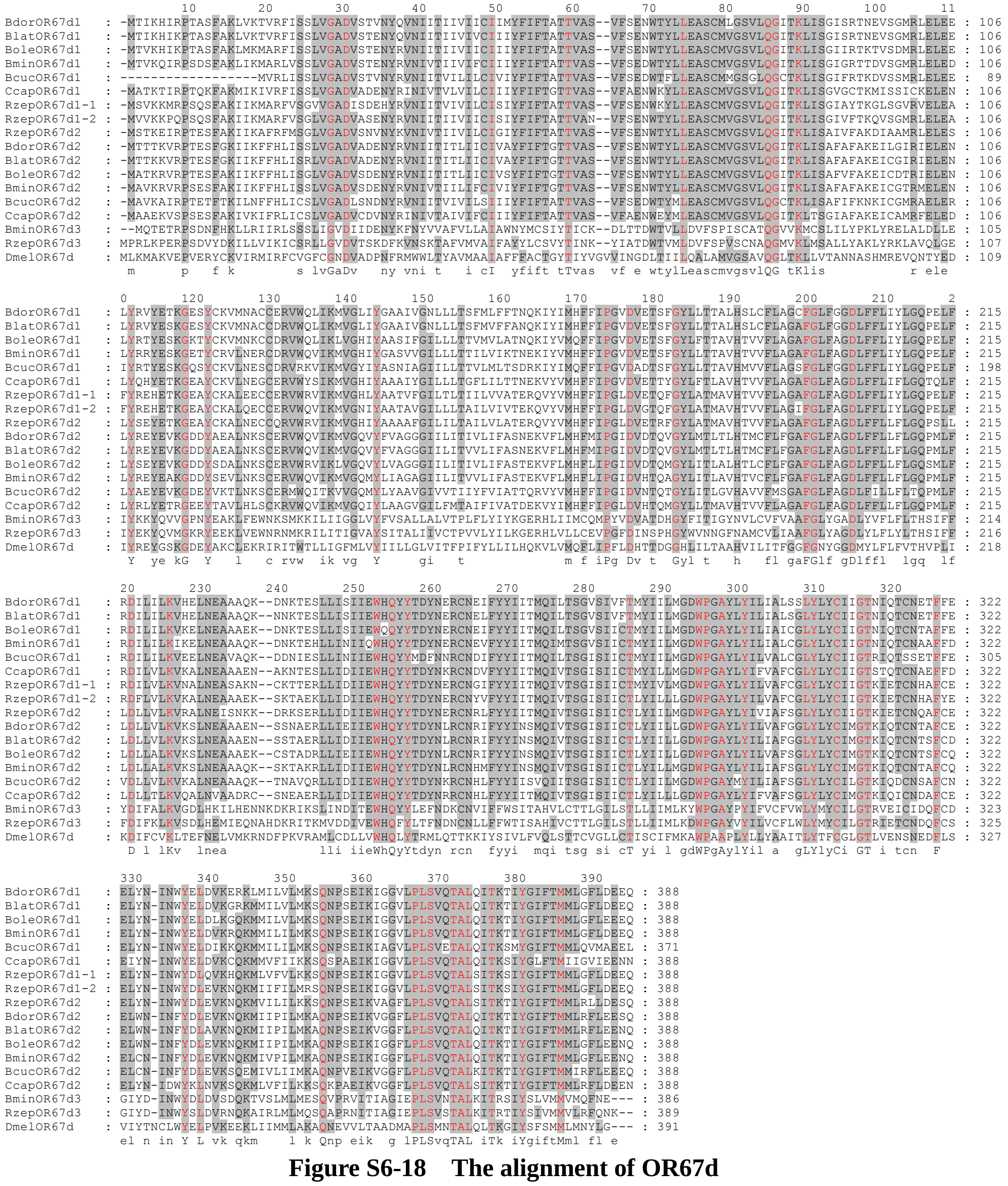

Figure S6-18 The alignment of OR67d

```
                    10        20        30        40        50        60        70        80        90       100       110
DmelOR69aB : -----MQLEDFMRYPDLVCQAAQLPRYTWNG--RRSLEVKRNLAKRIIFWLGAVNLVYHNIGCVMYGYFGDGRTKDPI--AYLAELASVASMLGFTIVGTLNLWKMLSLKTH : 103
DmelOR69aA : -----MQLHDHMKYIDLGCKMACIPRYQWKG--RPTERQFYASEQRIVFLLGTICQIFQITGVLIYWYCNGRLATETG--TFVAQLSEMCSSFCLTFVGFCNVYAISTNRNQ : 103
BdorOR69a1 : -MSEIYSMKHCLKYPYFTLDLAATEPFTWSG--ARTYSYRRIWLRRALFTFGAINLVYQNIGMLIYLFMPHESSAQ----STIVQVTETGGIMGLTMVGTSNMLVMFWYGDR : 105
BlatOR69a1 : -MSELYSMKHCLKYPYFTLDLAATEPFTWSG--ARTYSHRRIWLRRALFTFGAINLVYQNIGMLIYLFMPHESSAQ----STIVQVTETGGIMGLTMVGTSNMVVMFWYGDR : 105
BoleOR69a1 : -MSEIFTIKHCLKYPYFTLDLASTEPFTWSG--ARTYSYRRLWFRRMLFTLGAINLVYQNIGMLIYLFMPHESSAQ----STIVQVTETGGIMGLTLVGTSNMLVMFWYGDR : 105
BcucOR69a1 : -MSETYTLNDCLKYPYFTLDLASTEPFTWSG--APTHSYRKLWLRRALFIFGALNLVYQNVGMLIYLFMPHESSAE----TTIAQITETGGIMGLTMVGTSNMLVMFWYADR : 105
CcapOR69a1 : -MSGVFSMNHFLKYPNFTLDLAWSNPFAWSG--ARQYGYHMLWIRRLLFTFGAANLVYQNFGMMIYLCMPHEVSNE----STIGQITETGGIMGLTMVGASNMFVMYWHADR : 105
RzepOR69a1 : -MPRIYTLQHFLHYPQLTLNLAWSDPFAWSG--SRKYSSHAIWLRRALFIFGALNLVYQNFGMLIYLFMPHEISTE----SVIGQITETGGIMGLTMVGACNMFVMYWYGGR : 105
BcucOR69a2 : ---------------------------------------------------------------------------------------------------------------- :   -
CcapOR69a2 : ---------------------------------------------------------------------------------------------------------------- :   -
BdorOR69a2 : ------------------------------------------------------------------------------------------------------MWTLFWRRHD :  10
RzepOR69a2 : ------------------------------------------------------------------------------------------------------MWNIFWRRKD :  10
BoleOR69a2 : MSLRTYNLKTFLEYPQFAFKLVCFESFIWCDNEHRHESALWYRIKQLIFIFSTIILIVHVSALVAGVFVPRATPEAELYSSSDSKVFEALATISYYSCGIYKVWNLLYRRHD : 112

                  120       130       140       150       160       170       180       190       200       210       220
DmelOR69aB : FENLLNEFEELFQLIKHR-------------------AYRIHHYQEKYTRHIRNTFIFHTSAVVYYNSLPILLMIREHFSNSQQLGYRIQSNTWYPWQVQG---SIPGFFAA : 193
DmelOR69aA : IETLLEELHQIYPRYRKN-------------------HYRCQHYFDMAMTIMRIEFLFYMILYVYYNSAPLWVLLWEHLHEEYDLSFKTQTNTWFPWKVHG---SALGFGMA : 193
BdorOR69a1 : IAMLLEKFQQLFPTARLQRKAKFTKQS----LRGVEFPHRIEHFVLKSNKLMKLATTLYMFAFAYYNSLPIVEFLYEWTTPGIVWKYRYQSNTWYPWQNERNAKSFASFTLA : 213
BlatOR69a1 : IAMLLEKFQQLFPTAGLQRKAKFTKRS----LRGVEFPHRIEHFVLKSNKLMKLATTLYMFAFAYYNSLPIVEFLYEWTTPGVVWKYRYQSNTWYPWQNERNAKSFASFTLA : 213
BoleOR69a1 : IAMLLENFQQLFPTERVQIKAKSTKRR----LRGVKFPHRIEHFALKSNKLMRLATILYLFAFAYYNFLPIVEFLYEWWTPGVVWKYRYQSNTWYPWQNERNAKSFVSFVVA : 213
BcucOR69a1 : IAILLEKFQQLFPTERVQRQVK------------LEFPHRIEYFAVKSNKLMKLATTLYMYAFAYYNSLPIVEYLYEWSTPGIVWKYRYQSNTWYPWQNERNSKSFLSFAVA : 205
CcapOR69a1 : IALLLEKFQRLFPTKQLQWRAKHANGR----LRGVKFPHSVEYFALKSNKLMKIATTAYLFAFSYYNSLPIVEYLYESLTPGVELKYHYQSNTWYPWQNAHNRKSFIAFLAA : 213
RzepOR69a1 : ISALLEELQNLFPTARAQWRAN-CGRR----LRGVRIPYRVEYFAVKSNRLMKHTTTAYIFAFSYYNSLPIIEYLYEWLKPEEELKFRYQSNTWYPWQNSHNSHSFISFLAA : 212
BcucOR69a2 : ----MEELKALFPSIAKQKRLAEINESKEGI-IGSGGIYRLEYYEEKSRTIMQFITRYFMFAYVAYNSIPVMQLCFAVITQQEHITYRAQANAWYPWHNHNDHSSFMGFMLS : 107
CcapOR69a2 : ---------------------------------------------------------------------------------------------------------------- :   -
BdorOR69a2 : ILRVLEELKDLFPSVAKQRRIAELNESEKGQ-IGSVGIYRLRYYEEKSRASMRRLTRFIINCYIYYNSVPILQLCYAIVTHQEKIMYRAQANTWYPWHNHNDHSSFIGFMAA : 121
RzepOR69a2 : IGPLLEKFNELFPSVVIQKKLAEANDYKTELKRGSGGYYRFAYFEEKSRARMRFMTKYFVFAYFYYNIIPILQLIYEILSPNQTVTYKTQSNAWFPWHNHNNHSTFVGFVFN : 122
BoleOR69a2 : IARLLKELKDIFPSVAKQRRLAELNESKKDQ-LGSGSIYRLGYYEEKSRANMRRLTHFFKFAYCAYNAIPILQLCFAIMTHQEKITYKAQANAWYPWHNHNNHSSFIGFMVS : 223
             i  lle    lfp    q                     r      ks   m   t     a  yyn  pi               y  q n wypw n     sf  f

                230       240       250       260       270       280       290       300       310       320       330
DmelOR69aB : VACQIFSCQTNMCVNMFIQFLINFFGIQLEIHFDGLARQLETIDARNPHAKDQLKYLIVYHTKLLNLADRVNRSFNFTFLISLSVSMISNCFLAFSMTMFDFGTSLKHLLGL : 305
DmelOR69aA : VLSITVGSFVGVGFSIVTQNLICLLTFQLKLHYDGISSQLVSLDCRRPGAHKELSILIAHHSRILQLGDQVNDIMNFVFGSSLVGATIAICMSSVSIMLLDLASAFKYASGL : 305
BdorOR69a1 : YVCQVQSSLTGVAFIMAAEFMLCFFTTQLQIHFDYLANALETIDAAGANANEDLKYLINYHSQLLSYSKETNAIFNVSFMVNLCTSAIAICLMGFSMVMISLAHAFKYSIGL : 325
BlatOR69a1 : YVCQVQSSLTGVAFIMAAEFMLCFFTTQLQIHFDYLANALETIDAAGANANEDLKYLINYHSQLLNYSKETNAIFNVSFMVNLCTSAIAICLMGFSMVMISLAHAFKYSIGL : 325
BoleOR69a1 : YLCQVQSSLTGVAFIMAAEFMLCFFTTQLQIHFDYLANALETIDAAGANANEELKYLLNYHSQLLSYSKETNAIFNVSFMVNLCTSAIAICLMGFSMVMISLAHACKYSIGL : 325
BcucOR69a1 : YICQVQSSLTGVAFIMAAEFMLCFFTTQLQIHFDYLANALETIDAAAPNANEDLKYLINYHSQLLSYSKETNAIFNVSFMVNLCTSSIAICLMGFSMVMISLAHAFKYSIGL : 317
CcapOR69a1 : YICQVQSSLTGVAFIMAAEFMLCFFITQLQMHFDYLANALETIDAAGENANEELKFLIHHHGRLLSYSKEINAIFNISFLVNIFTSSIAICLMGFSMVMISVIHACKYCIGL : 325
RzepOR69a1 : YICQMQSSLTGVAFILSAKFMLYFFTTQLQMHFDYLAGALEAIDASGELANDELKFLINYHSKLLSFSKEINDIFNISFLVNFFTSAVAMCLMGFSMVMISLAHAFKYSVGL : 324
BcucOR69a2 : YLTQAIVEYTSIAFVMSGEFIFCFFTTQMLMHYNYLCSALSALDASAPDAVRQLKALISYHTHLLRLSKLINSIFNLTFALDLIITTFAISLMGLAIVLVNFADALMFSAGF : 219
CcapOR69a2 : ------------------------------MFYN------------------------------FSLSKLINSIFNFTFALDLITTTFAISLMGLTMVMVRFGQAVMFSAGF :  52
BdorOR69a2 : FLTQAIAEYVCIVFIVCSENLFCFFTTQMLMHFN-------------------------------YLSSALS-------ALAASAPDALLQLKALISYHNQLLRAVMFSAGF : 195
RzepOR69a2 : YLVQASAEFAGINFIMCGEYLFGFFNTQMQLHFDYLAIALETLDATAPDSMKQLKTLITYHANLLRLFNEINSVFNITFALDLINATFAISLMGLAMVMIEFGRAVMFSAGF : 234
BoleOR69a2 : FLTQASAEYVSVAFIISGEFLFCFFTTQMLMHFN-------------------------------YLSSAFN-------SLDASAPDALLQLKALISYHNHLLR-------- : 289
                q         f         ff tq   hf      l   d         l  l   h   l  s   n  fn  f         a  l            a     g

              340       350       360       370       380       390       400       410       420       430       440
DmelOR69aB : LLFITYNFSMCRSGTHLILTSGKVLPAAFYNNWYEGDLVYRRMLLILMMRATKPYMWKTYKLAPVSITTYMA----------------------TLKFSYQMFTCVRSLK- : 393
DmelOR69aA : VAFVLYNFVICYMGTEVTLASGKVLPAAFYNNWYEGDLVYRRMLLILMMRATKPYMWKTYKLAPVSITTYMA----------------------TLKFSYQMFTCVRSLK- : 393
BdorOR69a1 : TSFIVFTFFICYTGKELTETSDKLLNAAFYGNWYDGNLAYRKMILFFIMRCRIPTELRAYKFTTVSMPTFTA----------------------ILRSSYSLFTFFQAMGQ : 414
BlatOR69a1 : TSFIVFTFFICYTGKELTETSDKLLNAAFYGNWYDGNLAYRKMILFFIMRCRIPTELRAYKFTTVSMPTFTA----------------------ILRSSYSLFTFFQAMGQ : 414
BoleOR69a1 : TSFIVFTFFICYTGKELTETSDKLLNAAFYGNWYDGNLAYRKMILFFIMRCRIPTELRAYKFATVSMPTFTA----------------------ILRSSYSLFTFFQAMGQ : 414
BcucOR69a1 : TAFIVFTFFICYTGKELTETSDKLLHAAFYGNWYDGNLAYRKMILFFIMRCRIPTELRAYKFT-VSMPTFTA----------------------ILRSSYSLFTFFQAMGK : 405
CcapOR69a1 : LSFIVFTFFICYTGKELTDASDKLLYAAFYGNWYEGDLAYRKMILFLIMRCRIPTVLRAYKFTTVSMPTFTA----------------------ILRSSYSLFTFFQAMGK : 414
RzepOR69a1 : LSFIVFTLFICYTGSELTESSNKVPAAAFYSNWYEGDLAYRKMILFFIMRSRKPAVLSAYKFKTVSMPTFTT----------------------ILRASYSLFTFFQALGK : 413
BcucOR69a2 : SFFLLLGYLFCNNGDEILRETMQINSAIFYSNWYEGSPEYRRLIIFFIMRTKTPCQYQAYGYTPLSMETYMR----------------------ILKLSYQMFTSVRAIE- : 307
CcapOR69a2 : SFFLLLGFLFCNNGDELINATKNLGAAIFYSNWYEGSSEYRRMIIFFIMRTKTPCEYRAFGYMSLSMETYMRVKILIFVLIYFSSFSFLRFHFQILKLSYQLFTSFRAIE- : 162
BdorOR69a2 : SFFLILGYLFCKNGDELIRETMKISPAIFYSNWYEGSYEYRRLIIFFIMRTKAPCEFQAYGYTSLSMETYMR----------------------ILKLSYQLFTSFRAIE- : 283
RzepOR69a2 : SFFLILSYVFCKNGDEFTDVSNKLSSALFYSNWYEGSLEYRRMIIFFIMRTNKPCEYQAYGYTAISMVTYMR----------------------MLKLSYQLFTSFRAIE- : 322
BoleOR69a2 : --------------------------------------------------------------------------------------------------------------- :   -
               f       c  g e      k   a fy nwy g   yr mi f imr   p    ay     sm t                          l  sy  ft   a
```

Figure S6-19 The alignment of OR69a

```
                      10        20        30        40        50        60        70        80        90       100       110
BoleOR71a    : ------------------------------------------------MTFTFTLLLWMEIILSGDVDYVFQWMRLAITETCLIVKVLNIWYHAQTANELLQEWDKSDMF :  62
RzepOR71a1-2 : ------------------------------------------------------------------MEYASEVLKIALSETVVVLKVLNIWHFAPNAAKLLHEWEQSEFF :  44
BdorOR71a    : MAINKLANFRTLEPVLTFLGLW----EGGDSSWFKRQYRYYQLFMHTTITFTFACLMILEFIYSESLDYAIDVLKYMLVEMAIISKVLNAWYYEQQTAELVNELANSAIF : 106
BlatOR71a    : MAIKKLANFRTLEPILTVLGLW----QGGDSSWFKRHYRYYQLFMHTTITFTFASLMVLEVIYSESLDYAIDVLKYMLVEMAIICKVLNAWYYAQPTAELVNELEDSAIF : 106
BminOR71a    : MAFDSMANFRTLTRLLIILGLW----HGGNGSWFERHYRYYQLFLHTTLTFTFTLLMCLEVIYSESLNYAIDVLKYMLCEMALVFKVLNAWYYARKISELMNEWQVSEIF : 106
BcucOR71a    : MAIDTMVNFRTIVRILTLS-----------------------------VTFGFILLLSLEIIYSDGLDHAIDVLKYLLVVMALGIKVLNAWYYTRQITEVMYEWENSELF :  81
CcapOR71a    : MTFDNIANSRFLTRALVLLGLWPVITVHG--SWLQRNYCFYQLFLQITFTFSFTFLMLLEVICSESLDHATEVLKFLLTEMALVFKILNTWYYARKAAAFLHEWETGEMF : 108
RzepOR71a1-1 : MSFDTIANSRFLIRALTFLGLWPVVIGNGGGSWFKRYYRCYQLFLHITLTFTLSLLMWLDVIFSDNLDHATQVLKFLLTEMSLVLKIFNIWHYSLKAAGLLHEWQMSELF : 110
DmelOR71aX1  : MDYDRIRPVRFLTGVLKWWRLWP----RKESVSTPDWTNWQAYALHVPFTFLFVLLLWLEAIKSRDIQHTADVLLICLTTTALGGKVINIWKYAHVAQGILSEWSTWDLF : 106
DmelOR71aX2  : MDYDRIRPVRFLTGVLKWWRLWP----RKESVSTPDWTNWQAYALHVPFTFLFVLLLWLEAIKSRDIQHTADVLLICLTTTALGGKVINIWKYAHVAQGILSEWSTWDLF : 106
               m        r     l                                 tf f  l  le i s        vl   l       Kv N W y         Ew     F

                      120       130       140       150       160       170       180       190       200       210       220
BoleOR71a    : ALKTLEEQKMWQGAQRYFRKVVAVYSLLSICSVFMALFSVFFMDTLALPIPYWMPAS-WRGSNAWPLFLYEDIVVPFSCLCNTQIEFFLCYLMFHLTLCLRMIGIRMERL : 171
RzepOR71a1-2 : VPRTPQEQVMWRRAQNAYKKVLAFYRIWSIICVLLAFLSVVFMETPQLPVPYWIPVKKWREGNGWPLYLYDFVAVIFTCCCNIEIDSLQCYYLLHLTLCLRLIGLRMARL : 154
BdorOR71a    : ELRTSAEEQMWQKSQKNFRKLTMIYMGTGLNSAFCALLAAALMGAKELPYALWLPYD-WRDTYFWGIYCYECIAMPFTCLCNITIDLFQAYLLLHLTLCFRVISMRLERL : 215
BlatOR71a    : ELHTSAEEQMWQKSQKNFNKLTXIYMGTGLNSAFCALLAAALMDAKELPYALWLPYD-WRDTYFWGIYCYECIAMPFTCLCNITIDLFQAYLLLHLTLCFRVISMRLERL : 215
BminOR71a    : ALRTSDEKQMWQKTQKNFHKLTMTYICTGFNSAMCALIGVLLMGASELPYALWMPKN-WRETYFWGMYCYEFLAMPFTCVCNITIDLFQAYLLLHLTLCYRVISMRLERL : 215
BcucOR71a    : VLRNDDEKQMWAKTQKTFRKLGMTSFGLGFNSAMCALLGVLLMGATEQPYALWMPTN-WRDKYYWQMYIYQCLSMPFICFSNVTNDVFQAYLLLHLTLCFRVISMRLERL : 190
CcapOR71a    : VLRSNAEKNMWAKKQSTFRKIMLGYIYWSISSAVCALLSCLFINDQALPFPYWTPNH-WLEDYYWLMYFYELLTMPFTCLCNIEIDVFQCYLLLHLALCLRVVGMRLERL : 217
RzepOR71a1-1 : VLQTVGEQEMWRCAQRAFHKVVIFYVGCSFNAAICAFVAVPFMNTRELPFPFWLPRK-WREDYYWAMYFYEFIAMPFTCLCNIQIDLFLSYFLLHLTLCLRVIGKRLERL : 219
DmelOR71aX1  : ELRSKQEVDMWRFEHRRFNRVFMFYCLCSAGVIPFIVIQPLFDIPNRLPFWMWTPFDWQQPVLFWYAFIYQATTIPIACACNVTMDAVNWYLMLHLSLCLRMLGQRLSKL : 216
DmelOR71aX2  : ELRSKQEVDMWRFEHRRFNRVFMFYCLCSAGVIPFIVIQPLFDIPNRLPFWMWTPFDWQQPVLFWYAFIYQATTIPIACACNVTMDAVNWYLMLHLSLCLRMLGQRLSKL : 216
                l    E  MW   q  f k    y          a           lP   W P   w     W    Y     pf C cN   d    Yl lHL LC R    Rl rL

                      230       240       250       260       270       280       290       300       310       320       330
BoleOR71a    : GDQVNDVEITTELENIIKIHQRIEGMAKSCQKIIMWPVLAQIIFSSLIICCSIYSIQR-------ISFSENPSNFLGFIQYVVAMALEIFLPCYYANEITVESENLSNHL : 274
RzepOR71a1-2 : EDAGDNVEITSELLKLIQMQQRVKDMVKRCEEIVSLPVLVQIIFSSMIICFTAYYLQS-------LSFSNNPADFVAMVQYGFVMSLQIFLPCYYANELTAESLNLSKQL : 257
BdorOR71a    : EDAGKEDAITTELLNNIKMHQRVKELALKCEQVISIALLSQIMLTFLILCFIIYNMQNVKTENDIAQFSENPAHFLAMLQYALIISMEMFLPCYYGNELTVESEKLGFHL : 325
BlatOR71a    : ESAGTEDAITTELLNNIKMHQRVKELALKCEQVISLALLSQIMLTFLILCFIIYNMQNVETANDIAHFSENPAHFLAMLQYALIISMEMFLPCYYGNELTVESEKLGIHL : 325
BminOR71a    : DNAGTDEAITKEFLNNIKMQQRVKD--------------------------------------------ENPVHCLAMLQYVLIVSLQMFLPCYYANELTVESEKLGIHL : 281
BcucOR71a    : ANAGADGAITAELMNDIKMHQRVKEMAISCEHIISLSMLTQITLTFLIICFIIYNLAN-------ANFREDPVHCLAMLQYALIVSLQMFLPCYYGNELTLESEKLSINL : 293
CcapOR71a    : ANAGDENAITREFLKTIKMHRRINEW---------------IMLSALIICFIIYRMQS-------VHFSDNPTEYLAMFQYVVAMSMQIFLPCYYANELTVQSQNLSKSL : 305
RzepOR71a1-1 : GNATEDDAVTKEFVKIMKMHQRVIDMSKRCVQMVSLPVLMQIMLSSFIICFIIYRLQS-------VSFRENPAEYLAMLQYVVAMSMEIFLPCYYANELTVQSEKLSYHL : 322
DmelOR71aX1  : QHD--DKDLREKFLELIHLHQRLKQQALSIEIFISKSTFTQILVSSLIICFTIYSMQM-------SPVLQDLPGFAAMMQYLVAMIMQVMLPTIYGNAVIDSANMLTDSM : 317
DmelOR71aX2  : QHD--DKDLREKFLELIHLHQRLKQQALSIEIFISKSTFTQILS----------------------PVLQDLPGFAAMMQYLVAMIMQVMLPTIYGNAVIDSANMLTDSM : 302
                        t e    i  hqR                  qi     i c   y                 p   am QY         fLPcyY Ne t  s  L   l

                      340       350       360       370       380       390
BoleOR71a    : YNCDWTV--MSLYNRRQIFLYMEHLKQPITLYAGNYFQIGLPVFSKTMNNAYSLLALLANVNEEE--- : 337
RzepOR71a1-2 : YSCNWTD--MSAYNRRLIYLYMQDLKQPIALRAGSYFEIGLQVFSKTMNNAYTLLALLANVADEDE-- : 321
BdorOR71a    : YSCDWT--AMSAVNRRLIYVYMESLKKPVVLCAGRFFEIGIPIFSKAMNNAYSVLALLLNVNDDEQH- : 390
BlatOR71a    : YSCDWT--AMSAVNRRLIYVYMESLKKPVVLCAGRFFEIGIPIFSKAMNNAYSVLALLLNVNDDEEH- : 390
BminOR71a    : YSCDWTGFLVDFSPAAPNRSTAPSQRRSTFAIQRSAFEVQHAVG-QIVHTTYNHMNFGMQFDLWSLQY : 348
BcucOR71a    : YSSDWTG--MSAYNRRFIFHYMESLKKPLVLHAGSFFEIGIPIFAKAMNNAYSLLALLLNVNDDEQ-- : 357
CcapOR71a    : YNADWTG--MSAYNRRLMLLYMQYLKLPL-----------------TMNNAYSLLALLLNVSDDKDKK : 354
RzepOR71a1-1 : YCCDWTG--MSVYNRRMIFLYMEYLNQPLVLHAGQFFKIGLPIFSKTMNNAYSLLALLLRVSDDNKN- : 387
DmelOR71aX1  : YNSDWPD--MNCRMRRLVLMFMVYLNRPVTLKAGGFFHIGLPLFTKTMNQAYSLLALLLNMNQ----- : 378
DmelOR71aX2  : YNSDWPD--MNCRMRRLVLMFMVYLNRPVTLKAGGFFHIGLPLFTKTMNQAYSLLALLLNMNQ----- : 363
               Y  dWt   m    rr     m  l  p  l ag  f ig   f k mn aYs lall n
```

Figure S6-20 The alignment of OR71a

```
                     10        20        30        40        50        60        70        80        90       100       110
BdorOR74a1 : ------MLYRPRLKNGKLIPLSWPVAAYRLLNHICWPLRDDASYLLRLYDRFFWAFGFFIFMQHNDAELRYIIVNNNNLDEMLICGPTYLVLVEIHLRAFQLGLKKEPFKR : 105
BlatOR74a1 : ------MLYRPRLKNGKLIPLSWPVAAYRLLNHVCWPLRDDASYLLRLYDRFFWAFGFCIFMQHNDAELRYIIVNNNNLDEMLICGPTYLILVEIHLRAFQLGLKKDAFKR : 105
BoleOR74a1 : ------MLYRPRLENGKLIPLSWPVAAYRLLNHVCWPLQDDASWLQRLFDRFFWAFGFFIFMQHNDAELRYIIRNNNNLDEMLICGPTYLILVEIHLRAFQLGLKKEAFKR : 105
BminOR74a1 : ------MLYRPRLENGKLISLSWPIAAYRLLNNICWPLQDDATQLERLFDRFLWAFGFLIFMQHNDAELRYIILNNNNLDEMLICGPTYLILVEIHLRAFQLGLKKNAFKR : 105
BcucOR74a1 : ------MLYRPRLNNGKLIPLSWPIVAYRLLNNICWPLRDHASLLERLFDRFFWSLGFFIFVQHNDAELRYILVNNNNLDEMLICGPTYLILVEIHLRAFQLGLNKEAFKR : 105
CcapOR74a1 : ------MLYRPRLPNGKLIPLSWPLAAYRLLNNICWPLRDNANRLERLFDRFCWALGFFIFIQHNDAELRYILSNNNNLDQMLICGPTYLILVEAHLRAFQLGLKKNNFKN : 105
RzepOR74a1 : ------MLYRPNVSNKELIPLSWPLAAYRLLNNICWPLRDGANLPERLFDRFCWWLCFIIFIQHNDAELRYIVVNRDDLDQMLTCVPEYLILIEIHIRAFQLGLKKDDFKT : 105
DmelOR74a  : ---MSFHRYRPRLPGGELAPMPWPVSLYRVLNHVAWPLEAESGRWTVFLDRLMIFLGFLVFCEHNEVDFHYLIANRQDMDNMLTGLPTYLILVEMQIRCFQLAWHKDRFRA : 108
BdorOR74a2 : MRYLPISYHKPLLPNGLHPPIDWQLYGFFCAN--GWPLAAHITKTRYIADIMVTIMQFMSEGMVLIGEAVVMHDNLDNISFVCTVLAPNLILFEMMLRAYNIIYRRNSFRT : 109
BlatOR74a2 : ------------------------------------PLAAHITRTRYIADIMVTIMQFMSESMVLIGEAVVMQNNLDNISFVCTVLAPNLILFEMMLRAYNIIYRRNSFRT :  75
BoleOR74a2 : MRYLPISYHKPLLPNGRHPPIDWQIYGFLCAN--CWPLAALITKTRYIVDIMVIIMQFMSESMVLIGEAVVMHDNLDNISFVCTVLAPNLILFEMMLRVYNIIYSRNSFRT : 109
BcucOR74a2 : ---------------------------------------------------MVTIAQFMSESMVLIGEGIVMHDNLDNISFVCTVLAPNLILIEMMLRAYNIIYRRSSFRK :  60
BminOR74a2 : MRYLPISYHKPLLPNGRHPPIDWQLYGFFCSN--GWPLAAHITKTRYIVDILVTMMQFMSESMVLIGEAVVMYDNLDNISFVCTVLAPNLILFEMMLRAYNIIYKRNSFRT : 109
CcapOR74a2 : MRYLPSSYHKPLLPNGRHPPIDWQLYGFVCSN--CWPLARDITKTRRIIDMVITAMQFMSESMVLSGETIAMRNNLDDISFVCMVLAPYLILIELMLRAYNIIYKRNSFRT : 109
RzepOR74a2 : MRYLPSSYHRPLLPNGQYPPIDWQLYGYMGAC--CWPPAINISKWRYIVDAVMIMCQFISECSVLFGEMQLMCASLDDISFVCAILAPFLILFEMMLRAYNIIYKRASFRV : 109
                       p l ng   p  w        n   wpl           d       F         e      n              LiL E   lRa        F

                    120       130       140       150       160       170       180       190       200       210       220
BdorOR74a1 : FLQKFYAEIYIDESSHPKLYANIQKSLRPIWFYSLLYFSTLSSYVIMPVVNYLNNVKAPLYKMYYPFDITP-NPIYVAIVLSNIWVGFTVITMVSGEDNVLSEVMLHLNGR : 215
BlatOR74a1 : FLQKFYATIYIDESSHPKLYANIQKSLRPIWFYSLLYFSTLSSYIIIPIINYLNNVKVPLYKMYYPFDITS-NAIYVAIVLSNIWVGFTVITMVTGEDNVLSEVMLHLNGR : 215
BoleOR74a1 : FLQNFYAEIYIDQSSHSKLYANIQKRLRPIWFNSLLYFSTLSSYVIMPLINYLNNVKVPLYKMYYPFDITP-NPIYVAIVLSNIWVGFTVITMVSGEDNILSEVLLHLNGR : 215
BminOR74a1 : FLQKFYAEIYIDEVSHPKLHGNMRKRLRPIWFYSFLYFGTLSSYVIMPLINYLKNVKAPLYKMYYPIDIMP-NPIYVAVVLSNIWVGFTVITMVSGEDNVLSEVMIHLNGR : 215
BcucOR74a1 : FLQKFYAEIYIDEPAHPKLYASIQKRLRPIWFYSLLYFSTLSSYVIMPFVNYLNNVKAPLYKMYYPFDITP-NPIYVAVVLSNIWVGFTVITMVSGEDNILSEVMLHLNGR : 215
CcapOR74a1 : FLKRYYAEIYIDKPTHPKLYANIQKRLRPIWFYSFLYYSTLFSYVITPLTNYLKNVKAPLFKMYYPFDITP-NPIYVAVVLSNIWVGFTVISLVAGEDNILSEVMLHLNGR : 215
RzepOR74a1 : FLKKFYAEVYVDKSSHPKLYANIQQRLRPIWFFSALYYMTLSSFFLTLLNNNLNNAKEPLYKMYYPFDITP-HAIYVAIVFSNIWVGLIAITTISGEDNLLSEVMLHLNGR : 215
DmelOR74a  : LLQRFYAEIYVSEEMEPHLFASIQRQMLATRVNSTVYLLALLNFFLVPVTNVIYHRREMLYKQVYPFDNTQ-LHFFIPLLVLNFWVGFIITSMLFGELNVMGELMMHLNAR : 218
BdorOR74a2 : HIEEFYKKIYIQRTWNPELFEKIRRQQLPTKYSTFTYIITLVTYVYVPVSGLIKNERLVPFPINFGFDYTVPWPRYLVFLTMSMWTGFAVVGPLVAEANILAMQILHLNGR : 220
BlatOR74a2 : HIEEFYKKIYIQRTWTPELFEKIRRQQLPTKYSTFTYIITLVTYVYVPVSGLIKKERLVPFPINFGFDYTVPWPRYLVFLTMSMWTGFAVVGPLVAEANILAMQILHLNGR : 186
BoleOR74a2 : HIEEFYNKIYIQRSWKPEVFEKIRRQQLPTKYSTFTFIITLVTYVYVPVSGLFKNERLLPFPINFGFDYTVPWPRYLVFLAMSMWTGFAVVGPLIAEANILAMQILHLNGR : 220
BcucOR74a2 : HIEEFYKKIYVQRTWNPDLFEQIRRQQLPTKYSTCTYIITLVTYVYVPISGLIKNERLVPFPIRFSFDYTVPWPRYLVFLAMSIWTGFAVVGPLVAEPNLLAMQILHLNGR : 171
BminOR74a2 : HVEEFYKKIYIQRTWNPELFEKIRRQHLPTKYSTFTYIITLVTYVYVPISGLVKNERLLPFPIKFGFDYTVPWPRYVVFLAMSIWTGFAVVGPLIGEANMLAMQILHLNGR : 220
CcapOR74a2 : HIEEFYKKIYVQRTWNPELFEQIRRQHLPTKYSTFTYIITLVTYVYVPISGLVKNERLVPFPIQFSFDFTVPWPRYVVFLIMSIWTGFAVVGPLVSEANMLAMQILHLNGR : 220
RzepOR74a2 : HLEEFYKKIYIERSWNPELFEQIRRQQLPTKYSTSTYIITLVTYFYVPIMGLIHQQRLLPFPVIYKYDYKATFAGYLLFLAMALWTGFAVVGPLIAEANMLAMQILHLNAR : 220
                 fY  iY      p l   i     p       y  tL  y   p      n           fD t     y        W Gf v      E N l    lHLNgR

                   230       240       250       260       270       280       290       300       310       320       330
BdorOR74a1 : FLLLQQKLRHDAERLLHVVDDRNI-------ADGLRRRIIEAIEENVRLYKFAEDFEREFSFRIFVNLSFSAGLLCVLGFKVYT--------------------------- : 292
BlatOR74a1 : FLLLQQKLRHDAERLLHVVDDRNI-------ADGLRRRVIEAIEENVRLYKFAEDFEREFSFRIFVNLSFSAGLLCILGFKVYT--------------------------- : 292
BoleOR74a1 : FLLLQQKLRQDADRLLHIVDERNI-------ADELRRQIIEAVEENVRLYKFAEDFEREFSFRLFVSLSFSAGLLCVLGFKVYTNPMA--SFGFIFWICAKIMEMILVGQL : 317
BminOR74a1 : FLLLQQKLRQDAERLLHAADDRNI-------ADELRRRIVEAIKENVRLYKFAEDFKLEFSFRLFVNLSFSAGLICVLGFKVYT--------------------------- : 292
BcucOR74a1 : FLLLQQKLGQDAERLLHAGDDRNI-------ADDLRQRIVEAIEENVRLYKFAEDFEREFSFRLFVSLSFSAGLICVLGFKVYTNPMA--SFGFMFWICAKVMEMLLIGQL : 317
CcapOR74a1 : FLLLQQKLRQNADRLLHNTDGRYI-------ADALQDQIIEAIEENVRLYEFAKGFEREFSFRIFVNLSFSAGLLCVLCFKVYTNPMA--SYGFMFWICAKIMEMILVGQL : 317
RzepOR74a1 : FLLLQQKLRDDTKRLLDEANGKNVD------ADDLSRQIVEAIRENERLYKFAKEFEREFSFRIFTNLSFSAMLLCALGFKVYTNPMS--SFGFMFWICAKVMEMLLIGQL : 318
DmelOR74a  : YIQLGQDLRRSAQMLLKKSSSLNV-------AIAYRLNLTHILRRNAALRDFGQRVEKEFTLRIFVMFAFSAGLLCALFFKAFTNPWG--NVAYIVWFLAKFMELLALGML : 320
BdorOR74a2 : YSLLLEDLRNISRESIAEHEKCKRK-DNMLVTQRFRYRLYDIIRRNVELNDFAKSMQEQYSFRVFVMLALSATLLCVLGFLTATLGITAQNIRFVSWIIGKVVELLIFGRL : 330
BlatOR74a2 : YSLLLEDLRNIAKESIAEHEKCKWK-DNMLITQRFRYRLYDIIRRNVDMNDFAKSMQEQYSFRVFVMLALSATLLCVLGFLTATLGITAQNIRFVSWIIGKVVELLIFGRL : 296
BoleOR74a2 : YSLLLDDLRNISRESIVEHKNCKGK-DNMLITQRFRFRLYEIIRRNVELNDFAKSMQKQYSFRVFVMLALSATLLCVLGFLTATLGLTLKNIRFVSWIIGKVVELLIFGRL : 330
BcucOR74a2 : YSLLLQDLRKISKESIVEHERLKGK-DTLLVTQRFRYRLFEIIRRNVELNEFAKSLQEQYSFRVFVMMAMSATLLCVLGFLTATLGLTAQNIRFVSWIIGKVVELLIFGRL : 281
BminOR74a2 : YSLLLQDLRKISRDSIAEHERCKDK-DNMLVTQRFRCRLFEIIRRNVELNDFAKSLQEQYSFRVFVMMALSATLLCVLGFLTA---------------------------- : 302
CcapOR74a2 : YSLLLRDLRKIAREAIEEHEKCKGR-DKISVTQRFRYSLFDIIRRNVELNEFAKSLQDQYSFRVFVMMALSATLLCVLGFLTATLGLTAENIRFVSWIIGKVVELLIFGRL : 330
RzepOR74a2 : YTLLLRDLRKIAQNAIEWHAMHKGEGDNVSVTSRFRYGLIDVVRRNVELNKFAESLQEQYSFRVFVMMALSATLLCVLGFLTATIGLSTDNIRFVTWIIGKVIELLIFGRL : 331
                lL  Lr                          r      i  Nv l  Fa       sfR Fv    SA LlCvLgF   t

                  340       350       360       370       380       390       400       410
BdorOR74a1 : -------VSRLS------------------------------------------------------------------------- : 297
BlatOR74a1 : -------TNEMSYTFYECNWELVLLKSGDSKANVRLMKTLLLAISTIQKPFVLTGFKYFSVSLTAVLKILQGAGSYFTFLTSMR- : 369
BoleOR74a1 : GSTVIYTTNEMSSTFYECNWELVLLKSGDTKSNVRLMKTLLLAISTSQKPFVLTGFKYFSISLAAVLKILQGAGSYFTFLTSMRK : 402
BminOR74a1 : -------TNEMSSTFYESNWELVILRSRDTKANVRLMKTLLLAISTNQKPFVLTGFNYFSVSLTAVLKQQLASTNNNIPMADL-- : 368
BcucOR74a1 : GSSLIYTTNEISSTFYKSHWELVIQKSSDTNANVRLMKTLLLAITTSQKPFVLTGFNYFSVSLTAVLKILQGAGSYFTFLTSMRK : 402
CcapOR74a1 : GSTMIYTTNEVSSTFYECNWELVLMKSTDTKANVRLLKTLALAISTSGKPFVLTGFNYFSVSLTAVLKILQGAGSYFTFLTSMR- : 401
RzepOR74a1 : SSTLIATTNQISSAFYESSWELVIHQSKDSNANVRLMKLLVLAIGTSQKPFTLSGLNYFSVSLTAVLKILQGAGSYFTFLTSLR- : 402
DmelOR74a  : GSILLKTTDELGMMYYTADWEQVIHQSDNVGENVKLMKLVTLAIQLNSRPFFITGLNYFRVSLTAVLKIIQGAFSYFTFLNSMR- : 404
BdorOR74a2 : GTTLSTTTDKLSTSYYCCDWEDIILHSTNAEENKKLMKLIALAIHLNSNPFRLTGLNFSVVNYETVVAIFRGAGSYFTVIYAYR- : 414
BlatOR74a2 : GTTLSTTTDKLSTSYYCCDWEDVINHSTNAEENKKLMKLIALAIHLNSNPFRLTGLNFSVVNYETVVTILRGAGSYFTVIYAYR- : 380
BoleOR74a2 : GTTLSTTTDELSTSYYCCDWEDIILYSTNTEENKKLMKLIALAIHLNSKPFRLTGLNFSVVNYETVVAILRGAGSYFTVIYAYR- : 414
BcucOR74a2 : GTTLSTTTDELSTSYYCCDWEDVILHSTDAEENKKLMKLIALAVHLNSNPFRLTGLNFSVVNYETVVSILRGAGSYFTVIYAYR- : 365
BminOR74a2 : ------TTDELSTSYYCCDWEEVIFHSRDAEENKKIMKLIALAIHLNSKPFRLTGLNFSVVNYETVVSLAFC------------- : 368
CcapOR74a2 : GTTLSTTTNDLSTSYYCCDWEEIIFHSADAEENRKTMKLIALAIHLNSNPFQLTGLNFFVVNYETVVSILRGAGSYFTVIYAYR- : 414
RzepOR74a2 : GTTLSTTTNELSTSYYSCGWEDVILHSTDAVENAKLMKLMALAIHLNSNPFRLSGLAFFYVNYEVVVSILRGAGSYFTVIYAYR- : 415
                   tt   s   y   we     s     n  lmk   lai     pf ltg     v     v i  ga syft     r
```

Figure S6-21 The alignment of OR74a

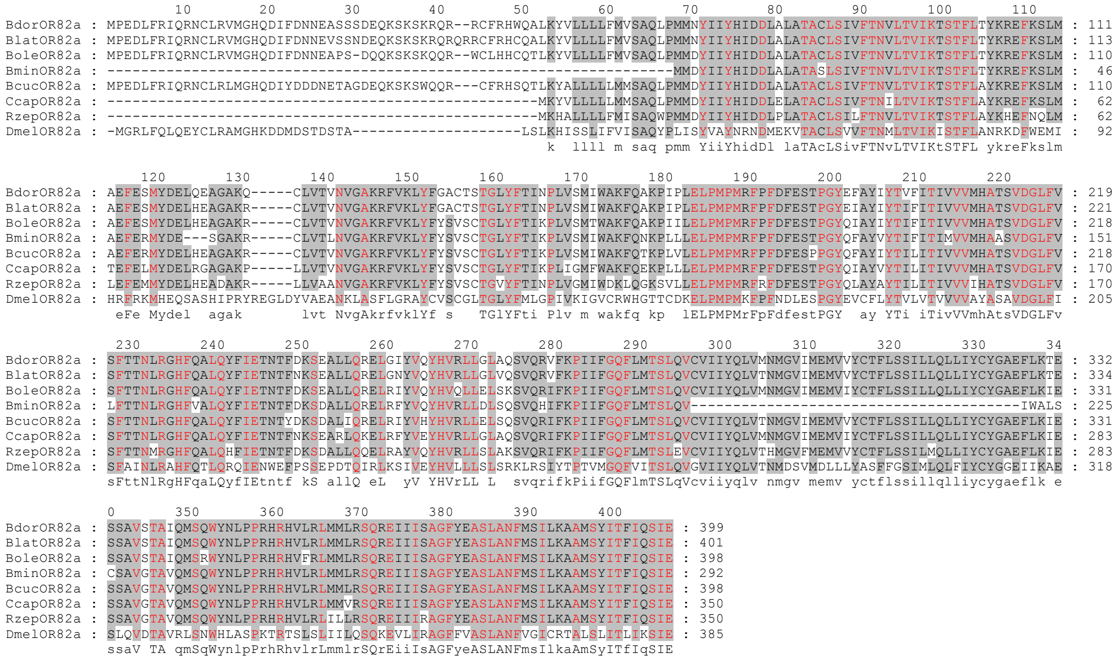

Figure S6-22 The alignment of OR82a

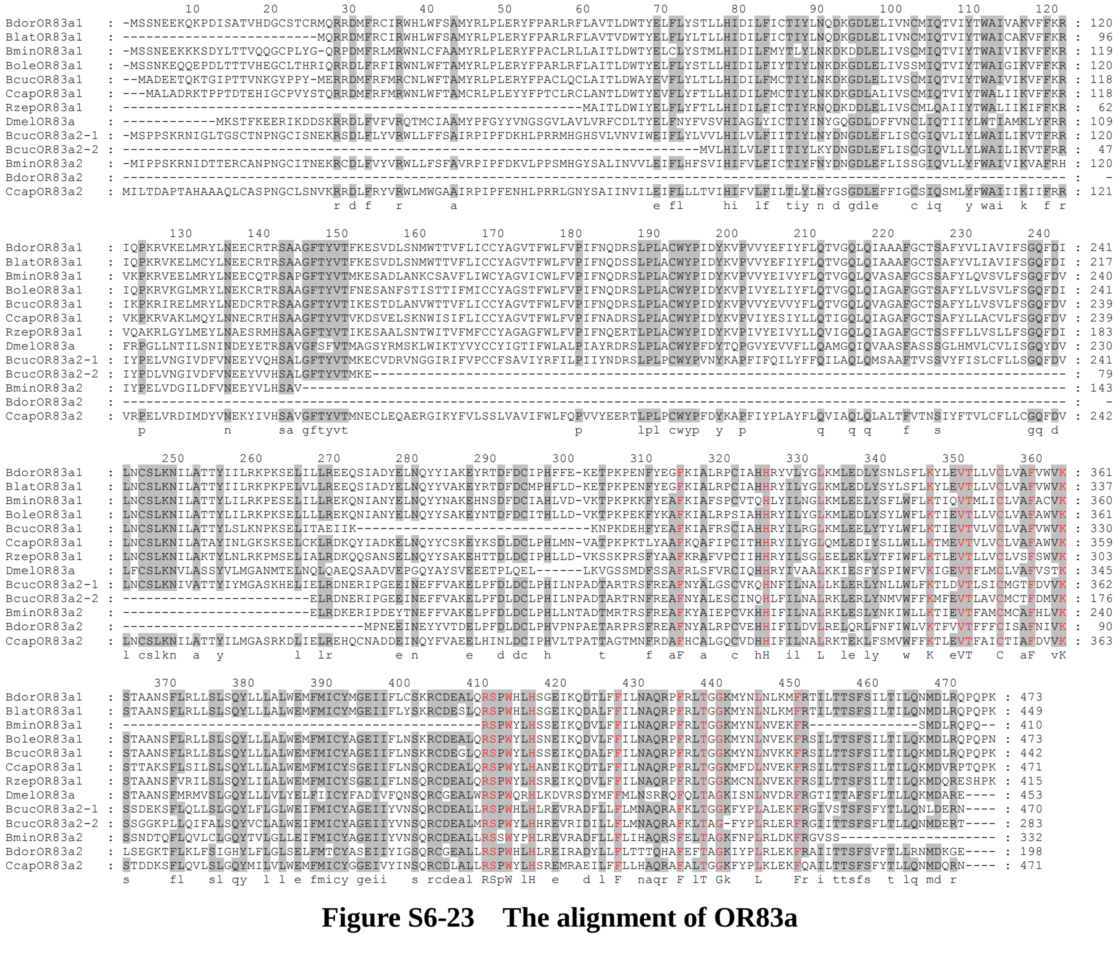

Figure S6-23 The alignment of OR83a

```
                        10        20        30        40        50        60        70        80        90       100       110
DmelOR85c     : -----------------MKFMKYAVFFYTSVGIEPYTIDSRSKKASLWSHLLFWANVINLSVIVFGEILYLGVAYSDGKFIDAVTVLSYIGFVIVGMSKMFFIWWKKTDLS :  94
DmelOR85b     : ----------------MEKLMKYASFFYTAVGIRPYTNGEESKMNKLIFHIVFWSNVINLSFVGLFESIYVYSAFMDNKFLEAVTALSYIGFVTVGMSKMFFIRWKKTAIT :  95
BdorOR85bc1   : ----------MSNIIRFEAFLRIPSFFYRSVGVDLWNTNGGP-----LQNAVFYISLFNVNVWLLSELIFAVLMLTKN-FIQATMTLSYAGFVLVGSIKMYFMWRKKAEMT :  95
BlatOR85bc1   : ----------MSNIIRFEAFLRIPSFFYRSVGIDLWNTNGGS-----LQSAVFYISLFNVNVWLLSELIFAVLMFTKN-FIQATMTLSYAGFVLVGSIKMYFMWRKKAEMT :  95
BoleOR85bc1   : ----------MPNVISFEAFLRIPSFFYRSVGVDLWNTNGGP-----LQNAVFYFGLINLNIWLLSELIFSVLMVSKN-FIQATMTLSYAGFVLVGTIKMFYMRRKKAEMT :  95
BcucOR85bc1   : MAPLRVCPSIMSKIIRFEAFLRIPNFFYRSVGVDLWNTDGGP-----LQDAVFYFGLLNVNVWLLSELVFAVLMVSKN-FIQATMTLSYAGFVLVGSIKMYFMWRKKAEMK : 105
CcapOR85bc1   : ---------MCENIESFEAFLRIPSFFYRSVGVDLWNTNGGS-----IQRFIFYFGFLNVNLWLLSELIFAIITVSEN-FIQATMTLSYAGFVLVGSIKMYFMWRKKTEMT :  96
RzepOR85bc1   : ----------MPDTIRFESFLRLPNFLYRSVGIDLWGTHGGL-----LQQALFYLCIINVNIWLISEVILALMIFGKN-IVQATMTLSYAGFVLVGFIKLYYMWRKKQELT :  95
BlatOR85bc2X1 : ----------MSTIIKFEQFLGLSSFFCYNIGIKLRGPNDGF-----WLNFWLYLASINLFLTVFAECIYIIMTIRSD-FIVAIMTLSYVSFIVVAYVKWYYLYNYQTERN :  95
BlatOR85bc2X2 : ------------------------------------------------------------------------MTIRSD-FIVAIMTLSYVSFIVVAYVKWYYLYNYQTERN :  38
BdorOR85bc2   : ----------MSTIIKFEKFLELASFFCYNIGIKLWGPNDGF-----WLNFWLYLTSINLFLTVFAECIYIIMTIRSD-FIVAIMTLSYVSFIVVAYVKWYYLYNYQTERN :  95
BoleOR85bc2   : ------------------------------------------------------------------------------------MTLSYVSFIVVAFVKWYYLINYHTERT :  27
BminOR85bc2   : -------------MENLEKFVRLANFFGNHIGMKPWGRLDGF-----QRRFLFYFSSINLLITFIAESSYIIVTITSD-FILAVMTLSYVTFIVVAYAKWYYLYTYQTERT :  92
Ccap85bc2     : ----------MSIIIRFEEFLRLPSFFSRNIGIILWGQRGKL-----FDRFMFYFSSINLFLTLLAELWYIISTISTD-FITAIMGLSYVSFVVLAEVKFYYLIKYDMKVS :  95
                                 e f     ff    g                          n       e            fi a mtLSY  F  v   K y       e

                      120       130       140       150       160       170       180       190       200       210       220
DmelOR85c     : DLVKELEHIYPNGKAEEEMYRLDRYLRSCSRISITYALLYSVLIWTFNLFSIMQFLVYEKLLKIRVVGQTLPYLMYFPWNWHENWTYYV-------LLFCQNFAGHTSASG : 198
DmelOR85b     : ELINELKEIYPNGLIREERYNLPMYLGTCSRISLIYSLLYSVLIWTFNLFCVMEYWVYDKWLNIRVVGKQLPYLMYIPWKWQDNWSYYP-------LLFSQNFAGYTSAAG : 199
BdorOR85bc1   : RFLQLMNTIFPRTETQQKKMNLRSHLRQCTIVMTVFAMIFMILIWTYNLYPYMQRQIYDCWLHMRSINKTLPYESYIPWNWHDHWTFYL-------YYTLQSIAGYHSASG : 199
BlatOR85bc1   : RFLQLMNAIFPRTEPQQKMMNLRSHLRQCTIVMSAFAMIFMILIWTYNLYPYMQRQIYDCWLHMRSVNKTLPYESYIPWNWHDHWTFYL-------YYTSQSIAGYHSASG : 199
BoleOR85bc1   : RFLQLMHTIFPHTEPQQKMMNLRRHLCQCTIVMSGFATIFMLLIWTYNLYPYMQRQIYDCWLQVRSVNKTLPYESYIPWNWHDHWSFYLTILYKLSYYTLQSIAGYHSASG : 206
BcucOR85bc1   : RFLQLMNAIFPRTEQQQKIMHLRRHLRQSTIVMSCFAMMFMVLIWTYNLYPYMQRQIYDRLLDVRSINKTLPYESYIPWNWHEHWTFYL-------YYTLQSIAGYHAASA : 209
CcapOR85bc1   : QFLKLMDEIFPRTAEQQKMMNLRRHLRQSTIVMSGFALIFMILIWTYNLYPFMQRQIYDCWLDTRSINKTLPYESYIPWNWHNHWSFYL-------YYVLQSIAGYHSAAG : 200
RzepOR85bc1   : RFLIQMDAIFPRKASQQQSMDVSRHLRQSSIVMSIFSLIVMMLICTYNLYPWLHRELYDCWLHMRSIEKSLPYESYIPWNWHDHWSYYL-------LYVSQSFAAYHAAAG : 199
BlatOR85bc2X1 : AFFQRLEALFPHTKSEQENIKLXEYFRLNKLATRGYTITFMVVIWLYNLYTISQRFIYTKLLHVH-IERVLPYQALYPWDWRDNWTYYV-------IYVTQDFAGFHATCA : 198
BlatOR85bc2X2 : AFFQRLEALFPHTKSEQENIKLXEYFRLNKLATRGYTITFMVVIWLYNLYTISQRFIYTKLLHVH-IERVLPYQALYPWDWRDNWTYYV-------IYVTQDFAGFHATCA : 141
BdorOR85bc2   : AFFQRLDALFPHTKSEQESIKLSEYFRLNKLATRGYTITFMVVIWIYNLYTISQRFIYTQLLHVH-IERVLPYQAMYPWDWRDNWTYYV-------IYVTQGFAGFHATCA : 198
BoleOR85bc2   : VFFQRLEALFPHTKSEQETIKLSKYLRLNKLATMSYTITFMVVIWIYNLYTISQRFIYTNLLHVH-IERVLPYQAMYPWDWRDNWTYYV-------IYVTQSFAAWHSTCA : 130
BminOR85bc2   : AFFQRLETLFPCTKSQQEAIKLSEYIRMNKLSTISYTISFLMCISTYTFYTIARQFIYTNLLHVPGTERNLPYQAVYPWDWRDNWTYYV-------CYVSQGFAGWHSTCA : 196
Ccap85bc2     : TVLKRLNALFPHTKEEQENIQLIKYLKMSKFYTLFYTVTFMLVIWTYNLYTVSQRFIYTKILQVREIERELPYPAIYFWNWQDNWSYFM-------LYISQSLAGWHATCA : 199
                 f       fP t   q    l    r            fm  Iw ynly   qr iY   L        LPY    pW W d W  y         y  Q  Ag h

                     230       240       250       260       270       280       290       300       310       320       330
DmelOR85c     : QISTDLLLCAVATQVVMHFDYLARVVE---------------------------KQVLDRDWSENSRFLAKTVQYHQRILR-LMDVLNDIFGIPLLLNFMVSTFVICFVGF : 281
DmelOR85b     : QISTDVLLCAVATQLVMHFDFLSNSME---------------------------RHELSGDWKKDSRFLVDIVRYHERILR-LSDAVNDIFGIPLLLNFMVSSFVICFVGF : 282
BdorOR85bc1   : QIASDLVLCAMATQIIMHYEYVAQRIT-EYQPQAL--------------RAPRHQVKESESYRKDMEFLCDIIAYHANILS-LSDIMNEVLGVPLLVNFMTSSFVICFVGF : 294
BlatOR85bc1   : QIASDLVLCAMATQIIMHYEYVAQRIT-EYQPQAL--------------KGAVRDVKESELYRKDMRFLCDIXEYHANVLS-LSDIMNEVLGVPLLVNFMTSSFVICFVGF : 294
BoleOR85bc1   : QIASDLVLCAMATQIIMHYEYVAQTIT-EYRPQLVDCKIS---------RAPRLDXESERSLCKDMKFLCDIIAYHANILRXLSDIMNEVLGVPLLVNFMTSSFVICFVGF : 307
BcucOR85bc1   : QIASDLVLCAMATQMIMHYEYVAQKIT-EYQPQVG-----------------ADSGSKNEAYCRDMKFLCDIIAYHANVLS-LSDIMNEVLGVPLLVNFMTSSFVLCFVGF : 301
CcapOR85bc1   : QIASDLVLCAMATQMIMHYEYVSHKIRSRYRGERKCVDSKS----VSCLNALNHWTEEQVATHKDMRWLCETIAYHSNLLS-LSDVMNDVLGVPLLVNFMTSSFVICFVGF : 306
RzepOR85bc1   : QIASDLVLCAMATQMVMHYEYVALKIE-QYQPKWGTSCCVNRQQQVKLVQASSQWEMKRAAYIEDMRFLGDLIAYHSNILS-LSNFMNEVFGVPLLVNFMASSFVICFIGF : 308
BlatOR85bc2X1 : QIAYDLLLCILSIQLIMHYDHISRSLE-GYQTKFAEVHGVD---INNGLPQLACAAVELRAVKEDIQFISNIVSYHNELLS-LSKSLNKLFGMPLFVNFFTSSAIICFLSF : 304
BlatOR85bc2X2 : QIAYDLLLCILSIQLIMHYDHISRSLE-GYQTKFAEVHGVD---INNGLPQLACAAVELRAVKEDIQFISNIVSYHNELLS-LSKSLNKLFGMPLFVNFFTSSAIICFLSF : 247
BdorOR85bc2   : QIAYDLLLCILSIQLIMHYDHISRSLE-EYQTKFAEVHGID---INNGLPPLMCAAVELRAVKEDIKFISNIVSYHNELLS-LSMSLNKLFGMPLFVNFFTSSAIICFLSF : 304
BoleOR85bc2   : QIAYDLLLCILSMHLIMHYDHISRSLE-GYQTKFAEVHGVE---TSNSLQQLARVAVELKAVKEDIKFISNIVAYHNELLR-FVVL------------------------- : 211
BminOR85bc2   : QMAFDLMLCTLSTHLIMHYDHISRSLE-GYQTKFAEIHG------KNGLTQQARAAMELKAVKDDIKFISSIVAYHTELLR-F---------------------------- : 271
Ccap85bc2     : QILTDLLICILISHLIMHYDHIARSLL-NYQSKFAELYGKES--TMKCMPKLARVMMEERAVRADMKFLADIIAYHTELLS-LTESLNDVFGVPLFMKFMSSSAIICFLGF : 306
                Qia Dl lC    q iMHy          y                                  d  f   i  YH   L  l    n   g pl  nf  ss  icf  f

                    340       350       360       370       380       390       400       410       420       430       440
DmelOR85c     : QMTVGVPPDIMIKLFLFLFSSLSQVYLICHYGQLIADASSSLSISAYKQNWQNADIRYRRALVFFIARPQRTTYLKATIFMNITRATMTDLLQVSYKFFALLRTMYIK : 389
DmelOR85b     : QMTVGVPPDIVVKLFLFLVSSMSQVYLICHYGQLVADASYGFSVATYNQKWYKADVRYKRALVIIIARSQKVTFLKATIFLDITRSTMTDLLQISYKFFALLRTMYTQ : 390
BdorOR85bc1   : QMTMDAEPDYMVKLFFFLFSSLIQIYLICHYGQQLIDASSNVSRAVYNHDWIHSHVHYQRMLVLVTARAQKPAMLKATSFVRISRGTLTDIMQISYKFFTLVRTMYSN : 402
BlatOR85bc1   : QMTMDAEPDYMVKLFLFLFSSLFQIYLICHYGQQLIDASNNVSRAVYNHDWVHSHVHYQRMLVLVTARAQKPAMLKATSFVRISRGTLTDIMQISYKFFTLVRTMYNN : 402
BoleOR85bc1   : QMTMDAEPDYMVXLFLFLFSSLFQIYLICHYGQQLIDASSNVARAIYNHDWIHSHVHYQRMLVLVAARAQKPAMLKATSFVHISRGTLTDIMQISYKFFTLIRTMYSN : 415
BcucOR85bc1   : QMTMDAEPDYMVKLFLFLFSSLVQICLICQYGQLLIDSSSNIAHAVYNHDWVHSHVHYQRMLVLVAVRAQKPAMLQATSFVRISRGTITDIMQISYKFFTLIRTMYSN : 409
CcapOR85bc1   : QMTMDAEPDYMVKLFLFLFSSLAQIYLICHYGQLLIDASINVAAAVYDQDWFDLNVRYQHMLVLVVARAQKPAMLKATNFVRISRGTLTDIMQISYKFFTLIRTMYSD : 414
RzepOR85bc1   : QMTMDTEPDYKVKLLLFFFSSLTQIYLICHYGQLLLDASINVAQAVFNNDWTNAHTHYQQMLVLVAARAQKPAILKATSFVHVSRGTMNDIMQISYKFFTLIRTMYSN : 416
BlatOR85bc2X1 : QMSVTREVDLLMKLAVFLFFSVMQVYLICHFGQLLSDASTNVASAAYFQDWSYADIRFQKMTILVAERAQEAAALKATNFITISLDTMTVIMQMSYKFFTLLRTMYAD : 412
BlatOR85bc2X2 : QMSVTREVDLLMKLAVFLFFSVMQVYLICHFGQLLSDASTNVASAAYFQDWSYADIRFQKMTILVAERAQEAAALKATNFITISLDTMTVIMQMSYKFFTLLRTMYAD : 355
BdorOR85bc2   : QMSVTREVDLLMKLAVFLFFSVMQVYLICHFGQLLSDASTNVASAAYFQDWSYADVRFQKMTILVAQRAQEAAALKATNFITISLDTMTVIMQISYKFFTVLRTMYAD : 412
BoleOR85bc2   : ------------------------------------------------------------------------------------------------------------ :   -
BminOR85bc2   : -------------------------------------------------DWSHADIRFQKMTILVAERAQDPAALKATKLITISRDTMTVIMQMSYKFFTVLRTMYGD : 330
Ccap85bc2     : QMTVNRGFDLLTKLALFFILSVLQVYLICHFGQLLIDASTNVSTALYSQDWTNADVRYQKMLVLIIKRAQRSATLKATNFIIISRATMTEIMQMSYKFFALIRTMYND : 414
                qm      d   kl  f   s  q ylich gq   das     a y  dw       q m  l   raq  a lkat f  is  t t imq sykff l rtmy
```

Figure S6-24 The alignment of OR85bc

```
                     10        20        30        40        50        60        70        80        90       100       110       120
BdorOR85d1 : -------------MSDQIIHFESFNTLANIFYTSIGLDAYQKAGQRTNN----------IRRQLLS-----IFFIITIANMNITLLSELLYIFMAFAKNNNFVEATMLSSFVGFVIVGDFK :  93
BlatOR85d1 : -------------MSNQIIHFESFNTLANIFYTSIGLDAYQKAGQRTNN----------IRRQLLS-----IFFIITIANMNITLLSELLYIFMAFANNNNFVEATMLSSFVGFVIVGDFK :  93
BcucOR85d1 : -------------MSNQPIQFERFTILANAYYTSIGLDAYEKTGQRTSN----------IRRRLLS-----IFFIFTIANMNITLLSELLYILMAFVSNNNFVEATMLLSFVGFVIVGDVK :  93
BminOR85d1 : -------------MSNQPIHFESFNYLANIFYTSIGLYAYEQAGERTKDR------AAYIRRQLLS-----IFSIIVIVNMNIALLSQLMYIFLAFANNNNFVETTMLSSFVVFVIVSDFK :  97
RzepOR85d1 : -------------MPNEPIHFESFNHLANIFYSSIGLDAYEKIGEQRTGR------LAQLRRQLQR-----IFFFITMANMNVTLLSEIIFIFLAFANGNNFVEATMLSSFVGFVIVGDIK :  97
DmelOR85d  : MLTKKDTQSAKEQEKLKAIPLHSFLKYANVFYLSIGMMAYDHKYSQKWK------------EVLLH-----WTFIAQMVNLNTVLISELIYVFLAIGKGSNFLEATMNLSFIGFVIVGDFK : 104
BdorOR85d2 : -----------------MIEFGAFMSTANFWYSFNGIVAYDDIYRQPGDA-PKQKSFAARFTTPLR----QIYSLIGLVNLIWVLIIEASFVVVNFIENSDFLQAARNFTFMGFVIVSILK :  99
BlatOR85d2 : -----------------MIEFGAFMSTANFWYSFNGIVAYDDIYRXPGDA-PKQKSIAARFTTVLR----QTYCLIGLVNLVWVLIIEASFVVVNFIENSNFLQAARNFTFMGFVIVSILK :  99
BoleOR85d2 : -------------MSTKIVEFDAFIKKANFWYGFNGIVAYDNFHRQAGDE-PKQKNIAARLATVLR----QIYCFIGLVNLFWVLIIEACFVVVNFIENSDFLQAARNLTFMGFVTVSIIK : 103
BcucOR85d2 : -------------MPTKIVEFDAFIKTANFWYSSNGVVAYDDIYRQAGNE-PNQRSITARLATLLR----QIYCFISLINLNWVLIIEASFVVVNFMENADFLQAARNLSFMGFVVVAILK : 103
BminOR85d2 : -------------MSTQIVEFDRFIKTANFWYSFNGIVAYDDIYRQPGDE-PKRRSVQTRLATVLR----QIFSFVSLANLTWVLIIETLFVVVNFVENADFLEAARNFTFMGFVIVAIIK : 103
RzepOR85d2 : -------------MPSNIITFSAFIRKANFWYNSNGIEPYDDVYDAAAEKSAVQESIAANLTRALRHYLSQFFSLIILINMNFVLLTEAAFVVVNFLENADFMQAAKNLTFLGFVFVAVFK : 108
                                 f  F   AN  Y   G  aY                        l              N    L  e       f  n  F  a     F gFV V   K

                   130       140       150       160       170       180       190       200       210       220       230       240
BdorOR85d1 : IYSIWRQRARITAMMQALHALYPRTLAEQIKYEVQRALQRYQRFAYAFVLLHELLVWSYNLFPLLNYFIYEVWLAARVVGKTLPYNCWTPFDWHVNDWRYYPMYLTQIAAGQACLSGQLAN : 214
BlatOR85d1 : IYSIWRLRARITAMMQALHALYPQTLAEQRQYEVQRVLKRYQRFAYAFVMLHELLVWSYNLFPLLXYFIYEXWLALRVVGKTLPYNCWTPFDWHVNBWRYYLMYLTQIAAGQACLSGQLAN : 214
BcucOR85d1 : IYSIWRQRERISAMIQALHALYPQTLAEQAKYAVERELQRYKRFAYAFVLLHELLVWSYNLFPLLNYFIYEVWLTWRVVGKTLPYNCWTPFDWHNSDWRYYTMYLTQIAAGQACLSGQLAN : 214
BminOR85d1 : IYNICRQRARISAMMQALHALYPQTLAEQTKYKVQWQLQRYNRLAYAFAVFYEMLVWGYNLFPLLNYLIYEVWLALRVVEKTLPYNCWTPFDWHNNDWIYYHMYLMLSASGQACISGQLAN : 218
RzepOR85d1 : IFSIWRQHSRISSMMQDLYALYPISVREQLFYDVKGQLKCYNRFAYAFVLLHELLVWSYNLFPLFNYLIYELWLEVRTVGKTIPYNCWTPFNWHDN-WLYYPMYLTQCVGGQTCLSGQLAN : 217
DmelOR85d  : IWNISRQRKRLTQVVSRLEELHPQGLAQQEPYNIGHHLSGYSRYSKFYFGMHMVLIWTYNLYWAVYYLVCDFWLGMRQFERMLPYYCWVPWDWSTG-YSYYFMYISQNIGGQACLSGQLAA : 224
BdorOR85d2 : ILSNLRQRSRISILMRKLYEIYPKQSTDQPPYELQSHLSHYRRIGFMHAFTHAFTVGTYNFLPMINYLFLAPLLQHTDVVRELPYYCWVPFEWRDN-WLYYPLYVSQVCASLTGLGGYLAS : 219
BlatOR85d2 : ILSNLRQRSRISILMRKLYEIYPKQSTDQPPYELQSHLLRYRLFGFMHAFTHAFTVGTYNCLPMINYLFLAPLLQHTDVVREVPFFCWVPFEWRDN-WLYYPLYVSQVYASLTGLGGYLAS : 219
BoleOR85d2 : ILSNLKKRSQLSILMRKLYEIYPKQSTDQPPYDLQSHLLHYRRIGFMYGFTHGFTVCAYNCLPMVNYLFLAPLLQLTDVERELPYFCWVPFEWRDN-WLYYPLYVSQVFASLTGLGGYLAN : 223
BcucOR85d2 : ILSNLKQRSRLSVLMQKLYEMYPKQAADQSPYELQRHLQHYRRIGFIYAFTHTFTVWAYNSLPMINYLFLAPLLQQTVVERVLPYSCWVPFEWRDN-WLYYPLYVSQVFAGHTSLAAYLAN : 223
BminOR85d2 : YLSNLKQRSRLSVLMQKLYEIYPKQSTDQRPYDLQSHLRHYRRIGFIYAFLYAFTVWAYNSLPMVNYLLLAPLLQQTVYERVLPYSCWVPFEWRDN-WLYYPLYVSQALAGHEALAAYLAS : 223
RzepOR85d2 : TWHDKRQRVRITWLMRQLYDIYPKLTVDQPQYELQQHLDHYQRITFAYVLTIGFTAWCYNFLPLLNYLIFEQWLQRGTWQPTLPYYVWVPFEWRNN-WLYYPLYVSQALATHNCLASYVAT : 228
             i     qr r    m  L   yP     Q  Y     L  Y r            v  YN  p  nY      L        lPy cW Pf W  n w YY  Y  q       l   lA

                  250       260       270       280       290       300       310       320       330       340       350       360
BdorOR85d1 : DLLLSAVAVQLIMHYRQLARRIELHVAGGGG-----GSGSKWRTAATNVCREQDLRFLRSVIAYHQQILN-------LSQALNDVFGISLFISFASTALIICFVLFQITIGANIDAIIMLA : 323
BlatOR85d1 : DLLLSAVSVQLIMHYRQLARRIELHVAGGGGDGDGGGSGSKWRXAATNVSRZHDLCFLRSIIAYHQQILNYFYTPSRLSQALNDVFGNSLFISFASTALIICFVLFQITIGANIDAIVMLA : 335
BcucOR85d1 : DLLLSAVAVQLIMHYRQLARRIEAHVADSRD-----DSASKRRGESANECAAHDLHFLRTIIAYHQQILN-------ISQVLNDVFGISLFINFTSTALIICFVLFQITIGANIDSIIMLA : 323
BminOR85d1 : DLLLSALAVQLIMHYRELARRIEAHVAG--------------RETSS-------------------------------------------------------------------------- : 251
RzepOR85d1 : DLLLSAVAVQLIMHYRQLAKRIESHIAGNGD----GTATRERQRISSLERSEVDLRFLRGIIAYHQQILK-------LSQAMNDVFGISLFISFVSTSLIICFVLFQITIGAAVDSLIMLA : 327
DmelOR85d  : DMLMCALVTLVVMHFIRLSAHIESHVAGIGS-------------------FQHDLEFLQATVAYHQSLIH-------LCQDINEIFGVSLLSNFVSSSFIICFVGFQMTIGSKIDNLVMLV : 319
BdorOR85d2 : DLLFCAATVQLIIHFRKLARDIEAYQAGCSC----------ATADVCTQQAQRDLDFLSAAVYYHSHTLA-------LCQLINEIFGLPVLINFISTSFVICFLAFQFSIGVPLDSMVALV : 323
BlatOR85d2 : DLLFCAATVQLIIHFRKLARDIEAYQAGCSC----------ATADVMAQQSQRDLDFLSAAVYYHSHTLA-------LCQLINEIFGLPVLINFISTSFVICFLAFQFSIGVPLDSMVALA : 323
BoleOR85d2 : DLLLCAATVQLIMHFRKLARDIEAYQAGSSC----------ATEEVIAQQAQRDLSFLSAAVYYHSRTLA-------LCELINDIFGLPVLINFISTSFVICFLAFQFTVGVPLSAMVALA : 327
BcucOR85d2 : DLLLCAATVQLIMHFRKLARDIEAYQAGSTN----------ATQFVNAEKAKRDLSFLSTAVYYHNHTLA--------------------------------------------------- : 283
BminOR85d2 : DLLLCAAMVQLIMHFRKLARDIQAYQAGSSC----------TKQDVMAQQAKRDLSFLSTAVYYHNRTLA-------LCQLINEIFGLPVLINFISTSFVMCFLAFQFTVGVSFDDLIMLV : 327
RzepOR85d2 : DLLLCAAAVQLIMHFRQLSRNIEQYEP---------------SVESEWSSVSKDLKFLCAIVSQHYHTLD-------LCQLINEIFGLPMLINFISISFVICFLAFQFTVGVPLGAMIMLI : 327
             DlL  A  vqlimH r Lar Ie   ag                         dl fl     yh   l             n  fg      f s     cf  fq   g        l

                 370       380       390       400       410       420       430       440       450       460
BdorOR85d1 : FFLFCSLVQIFLICYYAQQILEASEYISYAVYNHNWFDSDLRYRKMLIYIMARAQKPSKLQATALVIVSMPTMTDLLQLSYKGFAVIRTMYAREPKNFTK : 423
BlatOR85d1 : FFLFCSLVQIFLICFYAQQILEASEYISFAVYNHNWFDSDLRYRKMLVYIMARAQKPSKLQATSLVTVSMPTMTNTHGLFDK-------VVQNDP----- : 423
BcucOR85d1 : FFLFCSLVQIFLICYYAQQLLEASEYISFAVYNHNWFVADLRYRKMLIFIMARAQKPSKLQATSLVTVSMSTMTDLLQLSYKGFAVIRTMYAREPKSVSN : 423
BminOR85d1 : ---------------------DDSEYIRHAVYNHNWFDADMSYRKMLIFIMARAQKPSKLQATTFMTVSMSTMTDVSNSKYIVSYNIFHMYSKI------ : 324
RzepOR85d1 : FFLFCSLVQIFLICYYAHGLLEASEYIGSAVYNHNWYDADLRYKKMLLVIMQRAQKAAKLRATSFVIVSMPTMTDVSTK--------------------- : 406
DmelOR85d  : LFLFCAMVQVFMIATHAQRLVDASEQIGQAVYNHDWFRADLRYRKMLILIIKRAQQPSRLKATMFLNISLVTVSDLLQLSYKFFALLRTMYVN------- : 412
BdorOR85d2 : SYMICCLVQFYMICSYGQELITTSENIGHAVYNHNWLVADIRYKKMLIMIIRRAQKPAILKATTFVNISMGTLTDLLQLSYKFFALIRTMYAR------- : 416
BlatOR85d2 : SYMICCLVQFYMICSYGQELITTSENIGHVVYNHNWLVADIRYKKMLIMIIRRAQKPAMLKATTFVNISMGTLTDLLQLSYKFFALIRTMYAR------- : 416
BoleOR85d2 : CYMICSFVQIYMICSYGQQLITTSENIGHVVYNHNWLVADIRYKKMLIMIIRRAQKPAMLKATSFVNISMGTLADLLQLSYKFFALIRTMYAR------- : 420
BcucOR85d2 : ---------------------------------------------------------------------------------------------------- :   -
BminOR85d2 : FYMICSLVQIYMICSYGQELVTASENIGHVVYNHNWLVTDIRYKKLLIMIIERAQKPAILRATSFLNVSMGTITDLLQLSYKFYALIRTMYAR------- : 420
RzepOR85d2 : SYINCSLIQIYMICWYGQELITTSENVGHAVYNHNWLAANIRYKKMLTIIIQRAQKSATLRATSFVNVSMGTLTDLLQLSYKFFAIIRTMYAR------- : 420
                 c   q   i          se i   vynhnw   d ry kml  i  raqk   l at     sm t  d
```

Figure S6-25　The alignment of OR85d

```
                       10        20        30        40        50        60        70        80        90       100       110       120
BcucOR85e1-3 : MKERAERNTR-------------ELESNKLLYSN-EDKPRIPDLFVAQVICLKATGQIP---MNLGYG---------LGYIYCFIMIIQSLHMAVLFLKTAHDKLLHGELAEISDALTMTII :  96
BcucOR85e1-2 : ------MDTR-------------ALGDTTLLYSH-QDKPRIPDLFVAQIIFLKATGQIP---MNFGYG---------LGYGFCFIIIIQSLHMAVLFLKTAYDKLLDGELEEITDALTMSII :  90
BcucOR85e1-1 : MKDKTARDVR-------------ELESSTLLYSD-EDKPRIADLFVAQVICFKATGQIP---FNLGYG---------LGYVYCFFVITQTLHMAVLFLKTSYEMLLNGKLEEITDALTMTII :  96
BdorOR85e    : ----KNLSYYGRLRARCDVDKLLDLGGTSLLYSP-EDKPRIADLFGAHVIFFKATGQIP---FNVGYG---------LGYIYCCFFVAQSVHMAVLFWKTSYDMLLSGKLEEITDALTMTII : 105
BlatOR85e    : ------MDYR------------------DLTYSH-YDEARIADLFVAQVIFFKATGQIP---FNVGYG---------LGYIYCCFFITQSLHMAVLFLKTSYDMLLSGKLEEITDALTMTII :  85
BminOR85e    : ------MDFS-------------EQVESTLLYSH-EDKPRIANLFVAQVTSFKVTGQIP---FNVGYR---------LGYIYCFFVILQTLHMGVLFLKTSYDMLLSGKLEQITDALTMSIM :  90
BoleOR85e    : ------MDTR-------------ELGDMSLLYSH-DDKQRIADLFVAQVIAFKATGQIP---FDVHYG---------LGYTYCCFVITQSLYLGVLFLKTSYDMLLNGKLEQITDALTMTII :  90
CcapOR85e    : ------MQNT-------------AGACSIILYSE-KDKPRVCDLFMAQVLAFKATGQIP---FNWRWR---------LGYIYCFIVIAQTFLLAVLFLKSSYIMLLSGKLEEITDALTMTII :  90
RzepOR85e    : ------MLVK------------EFQQGASPRYSH-ADPARIGNLFAAQVAAFKATGQIP---FNIRYPPPLHLVNQLLGYIYCCFVISQTLCLGVLYLKCSYDMLLGGTLEEITDALTMTII : 100
DmelOR85e    : ---MASLQFHG-------------NVDADIRYDISLDPARESNLFRLLMGLQLANGTKPSPRLPKWWPKRLEMIGKVLPKAYCSMVIFTSLHLGVLFTKTTLDVLPTGELQAITDALTMTII : 106
                                              Ys   D  Ri  LF aqv   katGqiP    n  y          Lgy yC   i q l   VLflKt yd Ll G Le ItDALTMtIi

                    130       140       150       160       170       180       190       200       210       220       230       240
BcucOR85e1-3 : FWFSVYAATYCFLRSQRILAFLQRINHHYWQHSLPGLSFVCWHRTFVLAKRITVTWTLSCVAATILYGSTPLVMGVRALPLKCWYPFDPLQPYVYELVYALQLSTQVIMGVTLGNCSALFVS : 218
BcucOR85e1-2 : FSFSVYAGCYWLLRWQRLLAFLQCISHHYWHHSLPGLSFVCWHRTFVLAKRVTIAWTLACVAGTVLYGLGPLVMGAHVLPLKCWYPFDPLQPYVYELVYALQLSAQIIMGATLGNCSALFVS : 212
BcucOR85e1-1 : FWFSVYAACYWLLRWQRLLVFLRRINHHYWHHSLPGLSFVSSHRTFVLANRMSIVWTVACVAGTLLYGFAPLVMGVHVLPLKCWYPFDPLQPYVYELLYVLQLSAQMIMGATFGNGSALFVS : 218
BdorOR85e    : FSFSVYAAFYWLLRLKRLLAFLQRINQYYWHHSLPGLSFVSAYRPFILAKRITIVWVLICVASTALYGLPPLIMGVRALPLKCWYPFDPLQPYVYELVYVLQFTAQLIMGATLSNGSALFVS : 227
BlatOR85e    : FWFSVYAASYWLLRWKRLLAFLQRINQYYWHHSLPGLSFVSAHRTFILAKRMTITWTLTCMAGTALYGLAPLVMGVRTLPLKCWYPFDPLQPYVYELVYALQFTAQIIMGATFGNGSALYVS : 207
BminOR85e    : FWSGVYATCYWFLRSQRLLAFLQRINEHYWHHSLPGLSFVSWHRTFVLTKRLTTVWVLTCVVSTVSYGLAPLVMGVHALPLKCWYPFDPLN------------------------------- : 181
BoleOR85e    : FWFSVYAACYWLLRWQRLLAFLQHINHHYWHHSLPGLSFVSSHRTFILAKRITVVWALTCMAGTALYGLAPLVMGVHALPLKCWYPFDPLQPYVYKFVYALQLSAQIIMGATFGNGSALFVS : 212
CcapOR85e    : FWFSVYAACYWLLRWRRLMAFLELINQQYWHHSLPGLSFVSWQRTYLLAKRMTIVWTVACVLGTVLYGLAPLVMGVRALPLKAWYPFDPLQPYVYELVYVMQLSAQIIMGATFGNGSALYVS : 212
RzepOR85e    : FWFSVYAACYWLLRSRRLMTFLQYINQHYWHHSLPGLSFVSWHHSFVLAKRLTIVWTSVCVAGTVLYGLAPLVMGVRLLPLNTWYPFDPLQPYIYQLLYVQQLLAQIIMGATFGNGSALFVS : 222
DmelOR85e    : YFFTGYGTIYWCLRSRRLLAYMEHMNREYRHHSLAGVTFVSSHAAFRMSRNFTVVWIMSCLLGVISWGVSPLMLGIRMLPLQCWYPFDALGPGTYTAVYATQLFGQIMVGMTFGFGGSLFVT : 228
               f fsvYa  Yw LR  Rllafl  in  YwhHSLpGlsFVs hr f lakr t  W   C   t lyG  PLvmGv  LPLkcWYPFDpLqpy y   y  q   q img t gn sal vs

                  250       260       270       280       290       300       310       320       330       340       350       360
BcucOR85e1-3 : LVILMCGQFDVLYCSLKNLSYYGHLRAFCEVEKLRKEQSALPNTADD-EVNQYMYCQEHLANLSTVQHLYTQEPAVSLPEALHLGVVQCVQLHRFILDACKELEELFNPYCLVKSIQVTFQL : 339
BcucOR85e1-2 : LIILMCGQFDVLYCSLKNLNYYGRLRACCEVEKLRNEQAALPKISDD-ELNQYMYCREHLTNLSTLQHLYTQEPAVTLPKALHMAVVQCVQLHRFILDVCKEFEELFNPFCLVKSMQVTLQL : 333
BcucOR85e1-1 : LVILMCGQFDVLYCSLKNLSYYARLRSSFEVEKLRNEQAALPITSDD-ELNQYMYCQEHLTNLSTLQLLYTQQSAVTLPEALHMAVVQCVQLHRFILDACKELEELFNPYCLVKSIQVTFQL : 339
BdorOR85e    : LVILMCGQFDVLYCSLKNLSYYGRLRARCDVDKLRKEQAALPKPSDD-ELNQYMYCEEHLTNLSTLQHLYTQKPAATLPEALHLAVVQCVQLHRFILDVCKEMEELFNPFCLVKSIQVTLQL : 348
BlatOR85e    : LVILMCGQFDVLYCSLKNLTYYGRLRARCDMEKLRNEQAALPRTSDD-ELNQYMYCEEHLTNLSTLQHLYTQKPAVTLSEALHLAVVQCVQLHRFILDVCXEMEELFNPFCLVKSIQVTLQL : 328
BminOR85e    : ------------------------------------EQATLPETTDDDELNQYMYCREHLTNLSTLQHLYSQQPAVTFPEALHLAI------------------------------------ : 231
BoleOR85e    : LVILMCGQFDVLYCSLKNLSYYGRLRACCDVEKLRNEQAALPKTSDD-ELNQYMYCREHLTNLSTLQRLYSQQPAATLPEALHLAVVQCVQLHRFILGACKELEELFNPYCLVKSIQVTLQL : 333
CcapOR85e    : LVILMCGQFDVLYCSLKNLSHSARLRCCSGVEILRKEQAALPKSPDD-ELNQYMYCREHLTNLSILQHLYTQQPALTLPEALHLGVVQCVQLHRFILDACKELEELFNPYCLVKSIQVTLQL : 333
RzepOR85e    : LVMLMCGQFDVLYCSLKNLSYYGRLCAGYESEKLRNEQAALPTNQDD-ELNQYIYCNEHLTDLSALQHLHTQQPTATLPEAVRSAVVQCVQLHRFILDACKELEELFNPYCLVKSLQVTFQL : 343
DmelOR85e    : LSLLLLGQFDVLYCSLKNLDAHTKLLGGESVNGLSSLQEELLLGDSKRELNQYVLLQEHPTDLLRLS---AGRKCPDQGNAFHNALVECIRLHRFILHCSQELENLFSPYCLVKSLQITFQL : 347
               l  lmcgqfdvlycslknl     l        lr eQaaLp   dd ElNQYmyc EHltnLs lq ly q pa tlpeAlh avvqcvqlhrfil  c e eelfnp clvks qvt ql

                370       380       390       400       410       420       430       440       450       460       470       480
BcucOR85e1-3 : CLLIFIGVVGERQIVRILNLAQYAFLTFVEMLMLTYFGELLRGHSVRCGEAFWRSQWWTHSIVIRKDILILLANSKRAVRLTAGKFFAMDVERLRAVVTQAFSFLTLLLKLAAKNPK--- : 456
BcucOR85e1-2 : CLLVFIGVAGERSTVRILNLAQYAILTFIEVLMFTYFGELLRGHSVRCDEAFGRSQWWTHSVAIRKDILILLANSKRAVRLTAGKFYAMDVERLRSVVAQAFSYLTLLQNLAAKNPK--- : 450
BcucOR85e1-1 : CLLVFVGVAGERSMVRILNLVQYVTLTFIELLMFTYFGELLRGHSVRCGEAFWRSQWWTHSVAIRKDIIILLANSKRAVRLTAGKFYAMDVERLRSVVTQAFSFLTLLQKLAAKNQK--- : 456
BdorOR85e    : CLLVFVGVAGERSIVRILNLAQYVTLTFIELLMFTYFGELLRGHSVRCGEAFWRSQWWTHTAAIRQDILILLANSKRAVRLTAGKFYAMDVERLRSVVTQAFSFLTLLQKLAAKNPK--- : 465
BlatOR85e    : CLLVFVGVAGERSVVRILNLVQYVTLTFIELLMFTYFGELLRGHSVRCGEAFWRSQWWTHTAAIRQDILILLANSKRAVRLTAGKFYAMDVERLRSVVTQAFSFLTLLQKLAAKNPK--- : 445
BminOR85e    : ---------GERSMVRILNLVQYLTLTLIELLMFTYFGELLRRHSVRSGEAFWRSQWWTHAVAIRQDIFILLENSKRAVRLTAGKFYAMDVDRLRSLISQ--------QPIVG------- : 327
BoleOR85e    : CLLVFVGVAGERSMVRILNLVQYVTLTFIELLMFTYFGELLRGHSVRSGEAFWRSQWWTHTAAIRQDVFILLANSKRAVQLTAGKFYAMDVERLRSVVTQAFSFLTLLQKLAAKNPK--- : 450
CcapOR85e    : CLLVFVGVAGERSMVRIVNLAQYVTLTLVELLMFTYFGELLRGHSVRCGEAFWRXQWWTHTIPIRQDILILLANSKRAVRLTAGKFYAMDIERLRSVVTQAFSFLTLLQKLAAKNQK--- : 450
RzepOR85e    : CLLVFVGVAGERSMVRILNLAQYLTLTFVELLMFTYFGELLRGHSVRCGEAFWRSQWWTHTGAIRQDILILLVNSKRAVRLTAGKFYAMDIERLRSVVTQAFSFLTLLQKLAAKNP---- : 459
DmelOR85e    : CLLVFVGVSGTREVLRIVNQLQYLGLTIFELLMFTYCGELLSRHSIRSGDAFWRGAWWKHAHFIRQDILIFLVNSRRAVHVTAGKFYVMDVNRLRSVITQAFSFLTLLQKLAAKKTESEL : 467
               cllvf gv GeRs vRIlNl QY  LT  ElLMfTYfGELLrgHSvR geAFwRsqWWtH   IR Di IlL NSkRAVrlTAGKFyaMDveRLRsvvtQafsfltllqklaakn
```

Figure S6-26　The alignment of OR85e

```
                         10        20        30        40        50        60        70        80        90       100       11
BcucOR88a     : -MALRQEKY-GTAK---LDDLCDILHPVQRYLRLNYLDFRRVNGRFAIPNSKLLNICLILAVFDCIGNCIKCVKAINAGEITKAQEIFAVFGMGFVMTMRGLMLALNRV : 104
BminOR88a     : -MALQQERN-GAAKVCAIDDLCAILHPAQRYLGLNYLNFRRVNGRFAIPRSKLLNIALFLAVVDCTGNVIKCGKAINARDVTKAQEIFAVFGMGLVMTMRGLQLALNRD : 107
BoleOR88a1-2 : -MAQQQEWN-GVWKLCTIDDLCATQHPYQRYLGLKFLEFKRVNGRFVIPKSNILNVVLTLAVVDCAGNIIKIVKAINDRDVTQAQEVFAVCGMCLVMMMRGFLLALSRG : 107
BoleOR88a1-3 : -MALQQKRN-GVSNLCTFDDLCAIQHPYQRYLGLKFLEFKRVNGRFVIPKSNILNVILLLALMDCAGNIIKIGKAINDRDVTQAQEIFAVFGMGLVMTMRGSMIGLNRG : 107
BoleOR88a1-1 : -MAVQKKRT-FAAKLCAIDDLCAIQHPYQRYLGLKFLDFKRVNGRFAIPKSMLLNVAVFLAIIDCICNVINIAKAINERDVTKAQEAFAIFGMGLVLTMRGFMLAQNRD : 107
BdorOR88a     : -MAPQQEVFGAKSKLCAIEDLCAIEHPYQRYLGLKYVEFKRVNGRLVIPKSNILNFLLFLAVVDCTGNVIKTAIAINDRDVTKAQEVFAVFGMGLVMTMRGFMLGLNRG : 108
BlatOR88a     : -MAPQQEAFGARSKLCAIDDLCAIEHPYQRYLGLKYVEFKRVNGRFVIPKSNILNFWLFLAVVDCTGNVIKTAIAINNRDITKAQEVFAVFGMGLVMTMRGFMLGLNRG : 108
CcapOR88a     : -MALQAGRG-GEPKLRSIDDLCAILHPIQRYLSINFLDFTRINGRFAIPSSMLLNVGIVLSVLDCMGNITKVCMAINDRDLTKAQETFAVLGMAFVMTMRGMMLARSRV : 107
RzepOR88a     : -MGLTSGSTKTDKKLCTIDDLCAIYHPFQSYLRLNFMDYKRVNGRFAIPSSKLLQVGLVLAAYDCIGNAIKCVAAIASGNVNLAQEIFAVFGMAVVMTSRGFSLEFNRN : 108
DmelOR88a     : MKPTEIKKPYRMEEFLRPQMFQEVAQMVHFQWRRNPVDNSMVNASMVP---FCLSAFLNVLFFGCNGWDIIGHFWLG----HPANQNPPVLSITIYFSIRGLMLYLKRK : 102
                 ma         k    ddlcai hp qryl l    f rvNgrf ip s  Ln   l la  dC gn ik   ain     t Aqe fav gm  vmtmRG  l   R

                0        120       130       140       150       160       170       180       190       200       210       2
BcucOR88a     : QLSNFYNKIDCIFPRSAHLQQHMAVEKVHSYIKRRFYIMHTLMTVTVAAFLTTPGVKFMVFHDFDSDDSVADEYHVNPSWLPFGLKDKVSTYPYVYIYESVLAAAAVNM : 213
BminOR88a     : KLSKFYNDIDRIFPRSENLQQHMEVEKVHNYIKRRFFLLHTTLTVTVSAFLTMPAMKFLLFYDFESNDIVADEFHVNPSWLPFGVKDKVTTYPYIYIYEVILASAAVNM : 216
BoleOR88a1-2 : KLLKMFNAIDSIFPRSQRLQQHMEVEKMHKNIKRRFFLLHTSLTVSVSAFSALPLAKFMVFYDFKSDNRVIEEFHVNSSWLPFGIKDKVSTYPYIYVYEIMLATAGVHI : 216
BoleOR88a1-3 : NLSKMYNAIDRIFPRSQRLQQHMEVEKMHKNIKRRFFLLHTSLTVTVSAFIALPLAKFMVFYDFKSDNRVTEEFHVNASWLPFGIKDKVSTYPYIYVYEMMLATAGAHM : 216
BoleOR88a1-1 : KVLKMYNAIDRIFPRSEHLQQHMEVEKMHKNIKRRLLILHRSITALGVIFFSVPSVRFVLIYDFESDDLVAEEFHVNASWLPFGIKDKVSTYPYIYMYEIILALAAIQM : 216
BdorOR88a     : KLLKMYNAIDRIFPRSEHLQQHMEVEKVHNYIKKRFFYLHWFLTVSVCGFIFMPFVKFMAFHGFKSDAPVSEEFHVYASWLPFGVKDKVSTYPYIYVYELFLATAASHM : 217
BlatOR88a     : KLLKMYNAIDRIFPRSEHLQQHMEVEKMHNYIKKRFFYLHTFLTISVCGFISMPFVKFMAFHGFKSDAPVGEEFHVNASWLPFGVKDKVSTYPYIYLYELLLATAASHM : 217
CcapOR88a     : RLSELYNSIDRIFPNSSELQTHMEVAKTHDYIKRRFFLLHQGLSFALVLFCTMPAVKLVFFYDFEAQEPVADEFHVNPSWVPFQVKETISSYGYIYVYEVILALVAVNM : 216
RzepOR88a     : KLSKLYNDLDRIFPRSAFLQERMEVEKCHRYIKRRFFFFHNFMCAEVAPFCVMPLIKFLLAYDFEEKGPVPDEFHLNASWMPFGVKENLSVYPFIYAYETILALIAVNM : 217
DmelOR88a     : EIVEFVNDLDRECPRDLVSQLDMQMDETYRNFWQRYRFIRIYSHLGGPMFCVVPLALFLLTHEGK-DTPVAQHEQLLGGWLPCGVRKDPNFYLLVWSFDLMCTTCGVSF : 210
                 l   yN iDrifPrs  lQ hMevek h  ik Rf   h             F   P  kf     f     V  efhvn sWlPfg k   s Ypyiy ye  la      m

                20        230       240       250       260       270       280       290       300       310       320
BcucOR88a     : IITWDELFVVLISQLCMYYEYLGRLLEEMNVQDALDPTKLDAFYEQLHEYIYMHQYLNKLAVEFNDLFNFSILFSDAGIATSICFNIVLITDATDYLQIVTYTSPLFVE : 322
BminOR88a     : IVTWDEVFVVLISQLCMYYEYLGKLLEEMKVQDAMDAKKINAFYKQLHDYIYMHQYLNNLAVDLNDLFNFSILFSDAGIAISICFNLVLITDATDYLQMITYASPLFVE : 325
BoleOR88a1-2 : IATWDQVFVILISHLCMYYEYLGKLLAEMNEQEAMDPTKADAFYKQLHDYINIHQYLNNLAVELNDLFNLSILASDVGIAISICFNLVLITEATNYLQVINYATPLFIE : 325
BoleOR88a1-3 : LVTWDHVFVILISQLCMYYEYLGKLLAEMNVQDAMDPTKADAFYKQLHDYINIHQYLNNLAVEFNDLFNFSILSSDAGIAISICFNLVLISEATNYLQILNYTTPLFVE : 325
BoleOR88a1-1 : LIKWDQIFVILISHLCMYYEYLGKLLAEMNVQDAMDPTKADAVYKQLHDYIYIQQHLNNLAVELNDLFNLSILSSDAGIALSICFNLVLITEATNYLQMTLHTTPLFVE : 325
BdorOR88a     : LVVWDQIFVILISQLCMYYEYLGKLLAEMNVPDAMDPTKSDAVFKQLHDYIYMHQYLNNLAVQLNDLFNFSILSSDAGIAISICFNVVLITEAKNNLQIINYTIPLFVE : 326
BlatOR88a     : LVIWDQVFVILISQLCMYYEYLGKLLAEMNVQDAMDPAKADGVYKQLHDYIYMHQYLNSLATELNDLFNFSILSSDAGIAISICFNVVLITEAKNNLQIINYTIPLFVE : 326
CcapOR88a     : IITWDEVFVVLISQLSV---------------------------------LFFLHFVSSLADELNDLFNLSILVSDMGTAMSICFNLFLVTGAKDYLQIPSYLTPCFVE : 292
RzepOR88a     : IITWDQVFAVTMSHLCMYYQYLAKLLEEIDVREANDPQQRQAFFKQLHHYIYTNQCLNKIASDLNDIFNLSILISDMCIAASICFHLFLVSDASDYLAVATYIWPCLTE : 326
DmelOR88a     : FVTFDNLFNVMQGHLVMHLGHLARQFSAIDPRQSLTDEKR--FFVDLRLLVQRQQLLNGLCRKYNDIFKVAFLVSNFVGAGSLCFYLFMLSETSDVLIIAQYILPTLVL : 317
                   wD  Fv lis Lcmyy yl  ll e     a d  k       qlh yi   q ln la   NDlFn siL Sd giA SiCFn  l   a   Lq   y  P fve

                330       340       350       360       370       380       390       400       410       420
BcucOR88a     : VWLIYDAAKWGTMLETVTGRINEILYEQKWYESSVRFGKYTMMWMQSTNEPFRLTAFNMFYVNMKHFQDMMMLAYQMLTFLKSKS----------- : 407
BminOR88a     : VWLIYDAAKWGTLLETVTARINEILYEQKWYDSSARFGKYTMMWIQSTNEPFRLTAFNMFYVNMKHFQDVCSKCFL------------------- : 401
BoleOR88a1-2 : VWLIYDAAKWGTMLETVTARINEIIYEQKWYDCSVRFGKYTLMLLQSTNEPLRLTAFNMLYVNMKHFQDMMMLAYQLLTFLKSKG----------- : 410
BoleOR88a1-3 : IWLIYDAAKWGTMLETVTSRVNEIIYELKWYDCSVRFGKYTLMLLQSTNEPFRLTVFNIFYFNMKHFQDMMMLAYQLLX---------------- : 404
BoleOR88a1-1 : IWLIYDAAKWGTMLETVTARINEIIYEQKWYDCSVRFGKYTLMLLQSTNEPLRLTAFNMLYVNMKHFQDMMMLAY-------------------- : 400
BdorOR88a     : VWLIYDASKWGQMLETVTARINERIYEQQWYDSSIRFGKYTLMWIQSTNVPFRLTVFNLFYVNMKHFQDMMILAYQLLTFLKAKGYTK-------- : 414
BlatOR88a     : VWLIYDASKWGTMLETVTARINERIYEQQWYDSSVRFGKYTLMWIQSTNEPFRLTVFNMFYVNMKHFQDMMMLAYQLLTFLKAKGXTKLWLVGITK : 422
CcapOR88a     : TWLIYDVSKWGTMLETVTARINEVLYEQKWYDSSVH------------------------------------------------------------ : 328
RzepOR88a     : MWLLYDVAKWGTLLETVTSRINEVLYEQPWYESSLQLQKYTMMWMQGTNEPMRLTAFNIFNVNMKHFQDMMMLAYQMLTFMKSKS----------- : 411
DmelOR88a     : VGFTFEICLRGTQLEKASEGLESSLRSQEWYLGSRRYRKFYLLWTQYCQRTQQLGAFGLIQVNMVHFTEIMQLAYRLFTFLKSH------------ : 401
                 wliyd  kwGt LEtvt rine  yeq WY  S r  kyt m   q tn p rlt fn   vnmkhfqd m lay
```

Figure S6-27　The alignment of OR88a

```
                        10        20        30        40        50        60        70        80        90       100       110
DmelOR94a    : ----------------MDKHKDRIESMRLILQVMQLFGLWPWSLKSEEEW----TFTGFVKRNYRFLLHLPITFTFIGLMWLEAFISSNLEQAGQVLYMSITEMALVVKI :  90
DmelOR94b    : ----------------MES-TNRLSAIQTLLVIQRWIGLLKWENEGED------GVLTWLKRIYPFVLHLPLTFTYIALMWYEAITSSDFEEAGQVLYMSITELALVTKL :  87
BdorOR94ab1  : MWAQHIIKLELKMTPSAAEQQERIGVARVLMHFLQILGAWPILPEHHHQNASSTQCRTWLARNYRYLLHLPLTFTYNTLMWVEALTR--WERADHILYISITEVGMMALT : 108
BlatOR94ab1  : ------------MTPSAERQRERIGVARVLMHFLQILGAWPILPERYQQNARSTQRRTWLARNYRYLLHMPLTFTYNTLMWVEALTR--WERADHILYISITEVGMMALT :  96
BoleOR94ab1  : ------------MTPTATAQRERIGVARVLMRLLQILGLWPIWKESHQKNGSSTQCRNWLTHYYRYLLHVPLTFTYNTLMWIEALTR--WERADHILYISITEVGMMALT :  96
BminOR94ab1  : -----------MRETSASVLGTRIGIARVLMRLLQILGLWPMWQAR-QQSSTSTRCRIWLTRYYRYLLHAPLTFTYNTLMWIEALTR--WERADHILYISITEVGMMALT :  96
CcapOR94ab1  : -------------MATAIELQERIGAARVLMRMLQYLGLWPISQTG-LQRTRHTQCRARLAHYYRYLLHLPLTFTYNTFMWIEALTR--WERADHILYISITEVGMMALT :  94
BdorOR94ab2  : -------MAVKKWSPRNTSSMSRTASANIIIAVLKSLGYWQWTRDPRQ------PYIEKVERAYRIVLHTTLPFTFIALMLTGVLLSRDLDEIGSILHVLLTEFSLIVKT :  97
BlatOR94ab2  : -------MAVKKWSTRNTPSMSRTAAANTIIAVLKPLGYWQWTRDPRR------PYIEKVERVYRIVLHITVSFSFISLMLAGVLLSQDLEEISSILHILLTEFALVVKA :  97
BoleOR94ab2  : -------MAVKKWSSRSPSSMSRIAATLFIVHVLKAIGLWQWTRDSHL------PYFEKLEHAYRIILHIPFTFIFITLMFTAVLLSQDLEEISSVLHILLTELALVVKI :  97
                                       R        l  G w                         Yr  LH p tFt   lM    l      e     L     TE

                       120       130       140       150       160       170       180       190       200       210       220
DmelOR94a    : LSIWHYRTEAWRLMYELQHAPDYQLHNQEEVDFWRREQRFFKWFFYIYILISLGVVYSGCTGVLFLEG--YELPFAYYVPFEWQNERRYWFAYGYDMAGMTLTCISNITL : 198
DmelOR94b    : LNIWYRRHEAASLIHELQHDPAFNLRNSEEIKFWQQNQRNFKRIFYWYIWGSLFVAVMGYISVFFQED--YELPFGYYVPFEWRTERRYFYAWGYNVVAMTLCCLSNILL : 195
BdorOR94ab1  : LNFWRLDQRAYHFMHELCYSDHLALRNQAERQWWRAKQRSFTRIAVCYIGGGAGVLCTAFGATLLVNG--YSLPYDYWLPFEWHNAQNYWYAYGYELVAMSLTCIANVTM : 216
BlatOR94ab1  : LNFWRLEQHAYHFMHELSYSDHLALHNQEERQWWRAKQRSFTRIVVCYIGGGAGVLCTAFGATLLVNG--YSLPYDYWLPFEWHNAQNYWYAYGYELVAMSLTCIANVTM : 204
BoleOR94ab1  : LNFWRLEQRAYHFMHELSYSDNLALRNQAERQWWRGEQRFFARIAVCYIGGGVCVLFTAFGATLLVNG--YSLPYDYWLPFEWHNAQNYWYAYGYELIAMSLTCISNVTM : 204
BminOR94ab1  : LNFWRLDKRAWHFMHELSYSDRLALRNKAERQWWRKQQRFFARVVVCYIGGGAGVLVTAFGATLHMSG--YYLPYDYWLPFEWHNAQNYWYAYGYEVIAMSLTCISNVTM : 204
CcapOR94ab1  : LNFWRMERRAWQFMQEISHDSGLTLCNEVERIWWRKQQRTFTLIVICYIGGGAGVLFTAFGATLLMSG--YELPYDYWLPFEWHNARNYWYAYGYELVAMSLTCIANVTM : 202
BdorOR94ab2  : LHIWRKGGVAWRFMHEVANDPIYDLRQQSEWTKWQQAQRSFAIVSNTYFVAATTVVVFACIGAMMTPADVYVLPMNIYVPFDWHHPRRYWYAWTYNTIASLMTATANAML : 207
BlatOR94ab2  : LNIWRNCGAAWRFMDEAANDPIYALRQQSEWTKWQRAQRSFAIVTYIYCLASVAALVFACIGVMMTPADVYVLPCNIYVPFEWHHPRRYWYVWTYNTIAALMTCISNTTL : 207
BoleOR94ab2  : LYFWRQGNTAWRYMDELANDPMYALRQQSEWTKWQSAQRSFAIVAYTYILCSVAAVVFACIGGIMTPTDVYVLPVNIYVPFEWHHPHKYWYAWTYITLGILLTCVSNIML : 207
               L  Wr    A   m E         L     E    W    QR F       Yi        v    a                 Y LP       PFeWh     Ywya  Y    a  ltc  N

                       230       240       250       260       270       280       290       300       310       320       330
DmelOR94a    : DTLGCYFLFHISLLYRLLGLRLRETKN-------MKNDTIFGQQLRAIFIMHQRIRSLTLTCQRIVSPYILSQIILSALIICFSGYRLQHVGIRDNPGQFISMLQFVSVM : 301
DmelOR94b    : DTLGCYFMFHIASLFRLLGMRLEALKN-------AAEEKAR-PELRRIFQLHTKVRRLTRECEVLVSPYVLSQVVFSAFIICFSAYRLVHMGFKQRPGLFVTTVQFVAVM : 297
BdorOR94ab1  : DMMLCYYLFHVALLYKLIGMRLMALQH-------LSERLAV-QQLINIIELHKRVKRLTAQCEVLVSLPILVQIVLSVFILCLSAYRLQSMQINENPGQFFAMLQFASVL : 318
BlatOR94ab1  : DMMLCYYLFHVALLYKLIGMRLTALQQ-------LSEPLAV-QQLINIIELHKRVKRLTAQCEVLVSLPILVQIVLSVFILCLSAYRLQSMQISENPGQFFAMLQFASVL : 306
BoleOR94ab1  : DMLLCYYLFHVALLYKLIGMRLMALQH-------LSERLAV-QQLINIIELHKRVKRLTTQCEVLVSVPILVQIVLSAFILCLCAYRLQTMQISENPGQFLAMLQFASVL : 306
BminOR94ab1  : DMILCYYLFHVALLYKLIGMRLMALQQ-------LHEPLAV-QQLMNIIELHKRVKRLTAQCESLVSVPILVQIVLSAFILCLCAYRLQSMQISENPGQFLAMLQFASVL : 306
CcapOR94ab1  : DMMMCYYLFHVALLYKLIGMRLIALQY-------LSEPFAV-QKLRNIIELHKKVKRLTVQCESLVSLPILVQILLSAFILCLSAYRLQNMQINENPGQFLAMLQFASVL : 304
BdorOR94ab2  : DLVNCYFMFHLSLLYKLIGWRLSALRR-------SANEPPVIEQMSEIFQMHMKVRRLTTECETLVSIPVFSQIILSSFILCFCGYRLQQMEIMENLSMLFSTVEFATVM : 310
BlatOR94ab2  : DMIYCYFMFHLSLLYKLIGWRLRALRR-------RADEPPVIKQMSEIFQMHVNVRRLTTECETLVSMPVFGQIILSAFILCFSGYRLQQMGNMENLGMLFSTVQFATVM : 310
BoleOR94ab2  : DMIYCYFMFHLSLLYKLIGWRLSDLRRLKNTVGVKVDEPEVIDQLSEIFQMHMNVRRLTTQCETLVSVPILGEIVFSAFILCFSGYRLQQMNNLENIGMLFSTIQFASVM : 317
               D   CY   FH   lLykLiG RL al                 v   ql   I    H  v rLT   Ce lVS p l qi lS fIlC    YRLq m    en g       qFa V

                       340       350       360       370       380       390       400       410
DmelOR94a    : ILQIYLPCYYGNEITVYANQLTNEVYHTNWLECRPPIRKLLNAYMEHLKKPVTIRAGNFFAVGLPIFVKTINNAYSFLALLLNVSN : 387
DmelOR94b    : IVQIFLPCYYGNELTFHANALTNSVFGTNWLEYSVGTRKLLNCYMEFLKRPVKVRAGVFFEIGLPIFVKTINNAYSFFALLLKISK : 383
BdorOR94ab1  : TLQIFLPCYFANEITINSDALTTCVYNSNWEEFSPPTRKLMNLYMELMKRPEQIKAGNFFLVGLPVFTKTMNNAYSLLALLLNMSK : 404
BlatOR94ab1  : TLQIFLPCYFSNEITINSNALTTCVYNSNWEEFSPPTRKLMNLYMELLKRPEQIKAGNFFLVGLPVFTKTMNNAYSLLALLLNMNK : 392
BoleOR94ab1  : TLQIFLPCYFGNEITINSDALTTCVYNSNWEEFSPPTRKLMNLYMELLKRPAQIKAGNFFLVGLPVFTKTMNNAYSLLALLLNMSK : 392
BminOR94ab1  : TLQIFLPCYFANEITINSDALTTCVYNSSWEEFSPSTRKQMNLYMELLKRPAHIKAGNFFLVGLPVFTKVA-------AMI----- : 380
CcapOR94ab1  : TLQIFLPCYFANEITINSDALMTCVYSSNWEGFSPATRKQMYLYMELLKKPICIKAGNFFMVGLPVFTKTMNNAYSFFALLLNMNN : 390
BdorOR94ab2  : AVQIFLPCYFGNKVTESSDALTDEIFNSDWTTFDVPTRRFMILYMELLKKPANLMSVNYFIIGVDIFAKTMKNAYSIFALVLNMNN : 396
BlatOR94ab2  : AVQIFLPCYFGNKVTESSNALTDDIFNSDWTTFDVPTRKFMILYMELLKRPANLMSANYFKIGLPIFAKTMNNAYSIFALILNMNN : 396
BoleOR94ab2  : TVQIFLPCYFGDAVTEHSNALTNDIFNSDWTTFDMPARKFMILYMELLKRPAYLKSGNFFKIGLPIFAKTMNNAYSIFALLLNMNN : 403
                 QIfLPCYf n   T  s aLt      s W   f   tRk m lYMEllK P     gnfF  Glp F Kt nnays  Al ln
```

Figure S6-28　The alignment of OR94ab

```
                    10        20        30        40        50        60        70        80        90       100       110
BminOR1-1 : ----MGKLKVLSSRIFPSDPSKGKIGSIQYNVWLAQLFGVPVVGLKAESPCLKIVLGIYGILITLVVTFIYTGFEIYDMILCWPNLDSLTQNICLSLTHIAGVLK------- : 101
BoleOR1-1 : ----MRKLEVLSSRIFPSDPSKGKIGSIQYNVWLAQLFGVPVVGLKTESPRLRIALGVYGVLITLVVTFIYTAFEIYDMILCXPNLDSLTQNICLSLTHVAGVLKVINIIYR : 108
BdorOR1-1 : ----MDKLEELSSRIFPSDASKGKIGSIQYNVWLAQLFGVPVVGLKAESTRLRIALGIYGLLMTLVVTFFYTGFEVYDMILCWPNLDSLTQNICLSLTHVAGVFKVINIIYR : 108
BlatOR1-1 : ----MDKLEELSSRIFPSDAYKGKIGSIQYNVWLAQLFGVPVVGLKAESPRLKIALGIYGVLMTLVVTFFYTGFEIYDMILCWPNLDSLTQNICLSLTHIAGVFKVINIIYR : 108
BcucOR1-1 : ----MDKLAVLSSRIFPSDPSKGKIGSIEYNVWLAQLFGVPVVGLKAESPLMRIALGIYGVLLTLLVTFIYTGFEIYDMILCWPNLDDLSQNICLSLTHIAGVLKVINILYR : 108
CcapOR1-1 : ----MDKLEALSSRIFPSDPSIGKIGSIEYNVWLAQLFGVPVLGLKKETPRMRIALAVYGVVATLVVTFLYTGFEIYDMIFCWPNLDKLTQNICLSLTHVAGALKVINIIYR : 108
BdorOR1-2 : ---------------------------------------------------------------------------------------------------------------- :   -
BlatOR1-2 : MAAFQDILKEVYFRVFPSEAGKGQLGSIEFNIWLSQISGVPLS----------------------------------------PWFVPLHR--------------------- :  51
BcucOR1-2 : ---------------------------------------------------------------------------------------------------------------- :   -
CcapOR1-2 : MHRLLCKFEQVAMRVFPSDVRRGQIGSIEFNIWLSQMTGVPLP----------------------------------------PSFVPLRRRFRQ----------------- :  55
                         r fps    g  gsi  n wl q  gvp                                          p    l

                 120       130       140       150       160       170       180       190       200       210       220
BminOR1-1 : ------------------------LMAFYHGEFENKIPLTIYASLVGSTGVLGIMYLLHS------QIFPYRVKLPDWMPLGTQLAYMGISVLVFALQIVAIDYLNVTMINQ : 183
BoleOR1-1 : LDEVANVVRRIEYSAKTYVVSKSQLVAFYRGEFENKIPLTIYAALVGFTGVLGLIYLLYNPIGVAGQIFPYRVKLPDWMPFGTQLAYMGISVLVFALQIVAIDYLNVTMINQ : 220
BdorOR1-1 : LDEVAFVVRRIEYAAKTYVISKSQLVAFYRGEFENKIPLTIYASLVGFTGVLGLIYLFYNPIGVAGQIFPYRVKLPEWMPFGIQLAYMGMSVLVFALQIVAIDYLNVTMINQ : 220
BlatOR1-1 : LDEVGFVIRRIEYSAKTYVISKSQLVAFYRGEFENKIPLTIYASLVGFTGVLGLIYLFYNPIGVAGQIFPYRVKLPEWMPFGTQLAYMGMSVLVFALQIVAIDYLNVTMINQ : 220
BcucOR1-1 : LDEVAHVVRRIEYSAKTYVISKRQLVAFYRGEFENKIPLTIYASLVGFTGVLGIIYLFYNPVGVAGQIFPYRVKLPDWMPFGLQLAYMGISVLVFALQIVSIDYLNVTMINQ : 220
CcapOR1-1 : LKEVAGVVRKIEYAARYYVISKNQLKAFYRGEFENKIPLTIYASLVGFTGILGIAYLLHNPTGVAGEIFPYRVKLPHWMPFGLQLAYMGFSVLVFALQIVAIDYLNVTMINQ : 220
BdorOR1-2 : ---------------------------------------------------------------------------------------------MFAVQIVTVDYLNISMMNL :  19
BlatOR1-2 : ------------------------------TRLVNGLLLAY--------EYISSLLFFPDP-DMAGQIFPYRVTMPAWLPFFLQVVYIGVTDFMFAVQIVTVDYLNISMMNL : 124
BcucOR1-2 : ---------------------------------------------------------------MAGKIFPFRITLPAWLPFYMQVAYIGVTDFMFAVQIVTVDYLNISMINL :  49
CcapOR1-2 : --------------------------IFDRAEVENKLFLVIYATVIGFTGFTAMILVFINP-DMAGKIFPYRVALPAWLPLPVRVAYIGTTDFMFAVQIVTVDYLNIGMMNL : 140
                                              n   l                         ag ifpyrv lp w p   q ay g     FA QIV  DYLN  M N

               230       240       250       260       270       280       290       300       310       320       330
BminOR1-1 : IRFQLKILNLAFEELKLDCV---NKK-EHINLNKRLQTIVEHHCLLRDLRNDVEDLFRLPVLIQFFTSLVIFAMTGFQAIVKAENSNGAALIYCYCGCIFCELFVYCWFGNE : 291
BoleOR1-1 : IRFQLKILNLAFEELKLDCV---NKG-EQREFNKRLQTIIEHHCLLRDLRNDVEDIFRLPVLLQFFTSLIIFAMTGFQAIVKAENSNGAALIYCYCGCIFCELFVYCWFGNE : 328
BdorOR1-1 : IRFQLKILNLAFEELKLDCV---NAR-ELEEVNKRLHTIVEHHCLLHDLRNDVEDIFRLPVLLQFFTSLIIFAMTGFQAIVKVENSNGAALIYCYCGCIFCELFVYCWFGNE : 328
BlatOR1-1 : IRFQLKILNLAFEELKMDCV---NAR-EQEEANKRLQTIVEHHCLLRDLRNNVEDIFRLPVLLQFFTSLIIFAMTGFQAIVKVENSDGAALIYCYCGCIFCELFVYCWFGNE : 328
BcucOR1-1 : IRFQLKILNLAFEELKLDCG---SKKKAHFNHDKRLQTIVEHHCLLRDLRIDVEEIFRMPVLLQFFTSLIIFAMTGFQAIVKTENSNGAALIYCYCGCIFCELFVYCWFGHE : 329
CcapOR1-1 : IRFQLKILNLAFEELKFVSG---QAA-HELSLDRRLRTIVDHHNLLRNLRNEVEEIFRLPVLVQFFTSLIIFAMTGFQAIVKSENSNGASLIYCYCGCIFCELFVYCWFGNE : 328
BdorOR1-2 : LRCHLNIIKSTFDDLILDECHVRRDMKRIRDPNARMADIVEHHCILKSVRDDVEHIFRLSILLQFFTSLVISAVTGFQATMNSSNSNSEIIIYFYCFCIFTQLFGYCWFGNE : 131
BlatOR1-2 : LRCHLNIIKSTFDDLILDECHMRRDMRRIRDPNARMTDIVEHHCILKSVRDDVEHIFRLSILLQFFTSLVISAVTGFQATMNSSNSNSEMIIYFYCFCIFTQLFGYCWFGNE : 236
BcucOR1-2 : LRCHLNIIKSTFDELILDEQNARRDMK--RDPNARMADIVEHHCILKSVRDDVEYIFNLAVLLQFFTSLIVSAVTGFQATMHSSNSNSMMIMYFYCFCIFTELFGYCWFGQE : 159
CcapOR1-2 : LRCHLNVMKSSFDELNFNVKCMKSDIKRIRDPNERLADIVRHHCVLKSVRDDVEQIFRLPVLLQFFTSLVISAVTGFQATIYSSNFKSELIIYFYCFCIFTQLFGYCWFGNE : 252
             R  L i    F eL  d              n R   IveHHc L   R dVE iFrl vLlQFFTSL i A TGFQA     Nsn    iY YC CIF  LF YCWFGnE

             340       350       360       370       380       390       400       410
BminOR1-1 : VSEQSKTLTSSGYSSHCLNRERLSQQA----------------------------------------------- : 318
BoleOR1-1 : VSEQSKTLTASGYASHWYEFDQRFKKSLIIFMCNSQTPFVFTAGGFMSLSLPSFTGILSKSYTVIALLRQVYSR : 402
BdorOR1-1 : VSEQSKTLSASGYGSHWYAFDQRFKKSLLIFMCNSQTPFVFTAGGFMSLSLPSFTGILSKS------------- : 389
BlatOR1-1 : VSEQSKTLAASGYGSHWYAFDQRFKKSLLIFMCNSHIPFVFTAGGFMSLSLPSFTGILSKSYTVIALLRQVYSR : 402
BcucOR1-1 : VSEQSKTLTTSGYSSHWFEFDQRFKKSLLIFMCNSQTPFVFTAGGFMSLSLPSFTGILSKSYTVIALLRQVYSR : 403
CcapOR1-1 : VSEQSKTLTTSGYNCHWYQFGPRYKKSLLIFMFNSQKPIVFTAGGFMALSLPSFTGILSKSYTVIALLRQFYGR : 402
BdorOR1-2 : VNEQNKTLAAHGYGSSWYHFDQRFRKSLAIFLLNAQQPFNFTGGGFVDLSLPSFTNVLSKAYSFIAVLRQMYER : 205
BlatOR1-2 : VNEQNKTLATHGYGSSWYHFDQRFRKSLAIFQLNAQQPFNFTGGGFVDLSLPSFTNVLSKAYSFIAVLRQMYER : 310
BcucOR1-2 : VNEQNNTLAARGYGSSWYHFDQRFRKSLAIFLLNAQQPFNFTGGGFIDLSLPSFTNVMSKAYSFIAVLHRMYGR : 233
CcapOR1-2 : VNEQNKTLAARGYSSSWYYFDNRFRKSLAIFLVNAQQPFDFTGGGFVALSLPSFTGIMSKAYSFIAVLRQMYDR : 326
            V EQ kTL   GY s wy fd rf ksl if  n q pf ft ggf  lslpsft   sk y  ia l   y r
```

Figure S6-29 The alignment of OR1

```
                    10        20        30        40        50        60        70        80        90       100       110
BdorOR2-1 : --------------------MSVQPQQQPQQQQ----LDSAHAFRYLWLNWRLIGMHPTRRHRLPYYIYSACINISLGLFLPATMIAKLFFIENLSQLIGLLYLGVTLTMAT :  88
BlatOR2-1 : --------------------MSVQLQQQSQQQQ----LDSAHAFRYLWLNWRLIGMHPTRRHRLPYYIYSAFINISLGVFLPATMIAKLFFIENLSQLIGLLYLGVTLTMAT :  88
BoleOR2-1 : --------------------MSVQLQEQ-QKQQ----IDSAHAFRYLWLNWRLIGMHPTRRHRLPYYIYSACINISLGIFLPATMIAKLFFIENLSQLIGLLYLGVTLTMAT :  87
BcucOR2-1 : -------------------MSAVQPQQQQQQQHAAASLHSSHAFRYLWLNWRLIGMHPTRRHRLPYYIYSGCINISLGVFLPATMIAKLFFIENLSQLIGLLYLGVTLTMAT :  93
BminOR2-1 : -------------------------------------------------------MHPTRRHRWPYYIYSACINISLGIFLPATMIAKLFFIENLSQLIGLLYLGVTLTMAT :  57
RzepOR2-1 : --------------------MSALLQVTDTGAP----LNSARAFRYLWLNWRLIGMHPTRRHRCLYLAYSASINSVFGILLPATMIAKLCFIENLPQLIGLLYLGVTLTMAT :  88
BcucOR2-2 : --------------------------MNNK-QDAVGRLDSSDALRYVWLFWRITGIHPTAKYRGIYWLYSLLLNISSSVLFIAFYVVTFFISTDLLETLTNLSVMVPLIYTS :  85
BoleOR2-2 : --------------------------MANQ-PHSATRLDSSDALRYIWLFWRITGIHPVKKHRYIYYIYSLLLNFTSTVLFISFYVVTLFISNGLLEILTNLSVMVPLIYSS :  85
CcapOR2-2 : --------------------------MTKQEQATPTRLDSGDATRYVWLFWSIIGIHPFKKHRTLYWLYSVLLNFCCSAFFIAFYAVTFFVVSDLLEILANLSVMVPLIYNT :  86
BlatOR2-3 : --MNMIN-------------------KHKPLHATT-TLDTTEAFKYIWFCWRLFGMHRDLYERRFNWVYLIVLNLYCGVIYPMLYVASFFASIELSQKLANISVAVPIIYTF :  90
BdorOR2-3 : ----MTN-------------------KHKPLHATT-TLDTTEAFKYIWSCWRLFGMHRDLYERRLNWIYLILLNLYCGVIYPMLYICSFFTPMDLSQKLANISVAVPIIYTF :  88
BcucOR2-3 : NLQAIVNNFTIFQLTILHPGENTSKMSHKPLLTAT-TLDTKEAFSYIWYCWGFFGMYPDLYERRLNWIYLILLNLYCGVIYPLLYIGSLLTPMDLNQKLANISVAVPILYTL : 111
RzepOR2-3 : --MSIKQISLD--------DQRLMSSSQTTLSSPSPKLDTHDALRYIWLCWRYLGMLPFMQSSYIYYAYSVLIIFYGAILFPLFYVISLFVHIDFADRLANLSVALPLLYTT : 102
                                                  ld   a  y w  wr  gmhp    r  y  Ys   n       p       f    l      l   v l

                 120       130       140       150       160       170       180       190       200       210       220
BdorOR2-1 : AKQWSLWLHR-PKLLAVNYYLAKLDVRCMRHAVDRQHIRTAIRICHLYYAGYMFVYELSSSGFAYIGFSLRQLVYDGWFPQLYADAAQNLTVTLIYQNFAVMTFFVLQNVNN : 199
BlatOR2-1 : AKQWSLWLHR-PKLLAVNYYLAKLDVRCMRHAVDRQHILTAIRICHLYYAGYMFVYELSSSGFAYIGFSLRQLVYDGWFPQLYADAAQNLTLTLIYQNFAVMTFFVLQNVNN : 199
BoleOR2-1 : AKQWSLWLHR-SKLLAVNYYLSKLDVRCMTHAVDRQHILTAIRICHLYYAGYMFVYELSSSGFAYIGFSLRQLVYDGWFPQLYADAAQNLTVTLIYQNFAVMTFFVLQNVNN : 198
BcucOR2-1 : AKQWSLWLHR-SKLLAVNQYLDKLDARCMPHAVDRQHIRTAIRICHLYYAGYMFVYELCSSGFAYIGFTLRQLVYDGWFPQFYADEATNLTVTLIYQNFAVMSFFVLQNVNN : 204
BminOR2-1 : TKQWSLWLHR-SQLLAVHYYLGKLDARCMRHAVDRQHILTAIRICHLYYAAYMFLYELSSSGFAYIGFKLRQLVYDGWFPQFYADAAQNLTVTLIYQNFAVMTFFVLQNVNN : 168
RzepOR2-1 : AKQFSLWLHR-SKLLDVNLYLAKLDARCNHHAVDRQHLLWAIRICHFYYVAYMFVYELSSSGFAYIGFSLRQLVYDAWFPKLYEDADKNLTITLIYQNFAVMTFFVLQNVNN : 199
BcucOR2-2 : TKHLVVFYHIRGKLPQAAFHLQALDRRVELEPAACEHLRRLVQRCHRIFLAALAGIGVCLALYALVGIARHKLPFEGWLPFDWEHSLNAYILACAYQLFCLS-VQSIYALCS : 196
BoleOR2-2 : TKHFVVFYHIRGELPKAALHLQALDRRVELEPVACTHLKRLVQRCHKIYLAALAGIVVCLALYALVGILRHKLPFEGWLPFDWEHSLNAYILACAFQLFCLS-VQSIYALCN : 196
CcapOR2-2 : AKQLVIFYHIRRTLPQAALHLHALDRRAEQEPAAREQLKRLVQLSHRIFLTALTGIGICLTLYAMGGILRHRLPFDGWLPLDWEHSVGAYVAACAYQLFCLI-VQCIAALCN : 197
BlatOR2-3 : GKHVVIVYYTREDLPKALAQLKALDRLAESRPEDCAYMQKMVKNCHLVFFVSFVSFWFALLSYGVLEVLRHKLPFEGWVPFDWTSSEAAYVGACVIQLIGLI-IETTNAICC : 201
BdorOR2-3 : GKHVVIVYYIREDLPKALAQLKALDRLAESRPEDRAYMQKMVKNCHLVFFVSFVSFWFALLSYGVLEIFRHKLPFEGWVPFDWTRSEAAYVGACAIQLIGLG-IETTTAICC : 199
BcucOR2-3 : GKHVIIVYYLRKDLPKALRQLQALDRLAERRPKDREYMKRMVKNCHWIFLASSVSFWFALLSYGVLEMFRHKLPFEGWVPFDWQRTEFAYVCACALQLFGLG-IETTNAICC : 222
RzepOR2-3 : AKQAIIFYYIQGDLQKAAHHLHAMDRRAEGHIEDCAYLKRTVRYCHRIFIVNYAGFWLALLVYGLLGVMSHKLPFEGWLPFDWQHSRGAYTLACTLQMFCFC-SQVTNAVCI : 213
             K           L      L  lD r      d          cH                    g     L   gW P                Q f

               230       240       250       260       270       280       290       300       310       320       330
BdorOR2-1 : DMYPQCYLAVMIGHLRALTARISRIGK--DGVLSAEENISELINCIEDHKNLLGYFACIRPVISRTIFMQFGITAFVLCLTAVNYVAFERDAAQMLIAATYIFAVLIEALPC : 309
BlatOR2-1 : DMYPQCYLAVMIGHLRALTARISRIGK--DGVLSAEENISELVNCIEDHKNLLSYFACIRPVISRTIFMQFGITAFVLCLTAVNYVAFERDAAQMLIAATYIFAVLIEALPC : 309
BoleOR2-1 : DMYPQCYLAVMIGHLRALTARISRIGK--DGVLSVEQNIAELTNCIEDHKNLLGYFACIRPVISRTIFMQFGITAFVLCLTAVNYVAFERDAAQMLIAATYIFAILIEALPC : 308
BcucOR2-1 : DMYPQCYLAMMIGHLRALTARISRIGK--DGVLSEDENIAELTNCIEDHKNLLGYFACIRPVISRTIFMQFGITAFVLCLTAVNYVAFERDAAQMLIAATYIFAVLIEALPC : 314
BminOR2-1 : DMYPQCYLAILIGHLRALTARISRIGK--DGVLNVDENIEELTNCVEDHKNLLGYFNCIRPVISRTIFIQFAITAFVLCLTAVNYVAFERDAAQKLIAATYIFAVLIEALPC : 278
RzepOR2-1 : DMYPQCYLAVMIGHMRALSARISRVGK--DGALSVEDNIEELKNCIEDHKNLLSYFACIRPVISRTIFMQFGITAFVLCLTAVKYVAFGVDTAQMLIAITYIFAVLIEALPC : 309
BcucOR2-2 : DTYSIIYLLLLVAHLRILNARIARIGGACAAQCGEVANYQQLADCVRDHWECM---KCISPTIAATIFVQFLSTAFALCTAAVAFVNADSSVEQLMKFLPYLLVVLCEIAPC : 305
BoleOR2-2 : DIYSIVYLLLLVAHLRILNARIARIGGVCAEQHSELANYQQLAACVRDHWECV---KCISPAIAATIFIQFISTALALCTAAVAFVNADSIGEQLIKFLPYILVVLCEIAPC : 305
CcapOR2-2 : DTYTVIYLLLLATHLRILNARIASLG---HGECTEVENYRQLAACVRDHWACMNFYNSIRPAIAATLFIQFFSTAITLCTSAVAFVNAEDSVAQLFKFLPHLLVVVCEILPC : 306
BlatOR2-3 : DTYPVTYLILLVAHLRVLNGRIERVGS--AGAASDAESYRELVACVEYHKECMSYYNSLRPTLSGIYFIQFLSTGLGLSMPAIAFVGGNFSFSHVIKFLIIFGVILIEVAPC : 311
BdorOR2-3 : DTYAVTYLILLVAHLRVLNGRIERVGS--AGATSDAESYRELVACVEYHKECMSYYNSLRPTLSGIYFIQFLSTGLGLSMPAIAFVGGNFSFSHVIKFLIIFGAIIIEVAPC : 309
BcucOR2-3 : DTYAVTYLVLLVAHLRILNRRIARAGN--TGDGSDAENYRELVACVEYHKECISYYNSIRPTLSGTCFIQFLSTGLGLSMPAIAFVGGSFSFSHVIKFLIIFGAIIIEVAPC : 332
RzepOR2-3 : DTYSVTYLALLVAHLRVLNARIERIGN--CNTRTETDLYGELIACVQDHRECMCYYNCIRPAISGTLFVQFLSTALGLCAPAIAFVGGDFDLFQLLKFIIFFFAIIIEVAPC : 323
            D Y   YL     HlR L aRI r G    g       n eL  C  dH     y   irP is t F QF  Ta  Lc  A   V       q           iE  PC

              340       350       360       370       380       390       400       410       420
BdorOR2-1 : CWYVNSLMEECAQLTTALYNCQWYDQNRRFRKMLIIFMQRSQRTMVLMAGDLVPITLQTFLNIIKFSFS--------MYTILKG----- : 385
BlatOR2-1 : CWYVNSLMEECGQLTTALYNCQWYDQNRKFRKMLIIFMQRSQRTVLLMAGDLVPITLQTFLNIIKFSFS--------MYTIMKG----- : 385
BoleOR2-1 : CWYVNSLMEECSQLTTALYNCQWYDQDRKFRKMLIIFMQRSQRTMVLMAGDLVPITLQTFLNIIKFSFS--------MYTILKG----- : 384
BcucOR2-1 : CWYVNSLMEECGELTTALYNCQWYDQNRKFRKMLIIFMQRSQRTMLLMAGDLVPITLQTFLNIIKFSFS--------MYTILKG----- : 390
BminOR2-1 : CWYVNSLMEECGELTTALYNCQWYDQNRKFRKMLIIFMQRSQHTMVLMAGDLVPITLQTFLNLFCLCVQSIYALCSDIYSIIYLILLVA : 367
RzepOR2-1 : CWYVNALLEECGQLTLAIYSCQWFEQSKEFRKILIVFMQRSQRATVLMAGNLVPITLQTFLNIIKFSFS--------MYTILKG----- : 385
BcucOR2-2 : CWLMDEAALEMLKLTNALFSCCCSDGN-------------------------------------------------------------- : 332
BoleOR2-2 : CWLMDEAALEMFKLTNALFSCCWYEQNLRFRRSLIIFMQRSQKVEQILAGKIFPVSLVTFIKILKLAFS--------LFTLLNQLKS-- : 384
CcapOR2-2 : CWLMDKAALEMQDLTKSLFACRWYEQNQKFRRSLLIFMQRSQKVEKILAGDLVPVSLETFVNIIKFAFS--------LFTLLNQIKSK- : 386
BlatOR2-3 : CWFMDEVLFEMRRLTNSMFSCRWYEQNLKFRKALIIFMQRSQIAQPLLAGNLIPVSLDTFTNIIKFAVS--------LFTLLNKLNS-- : 390
BdorOR2-3 : CWFMDEVLVEMRRLTNAMFSCRWYDQNLKFRKALIIFMQRSQIAQPILAGNLIPVSLETFTNIIKFAFS--------LFTLLNQLNS-- : 388
BcucOR2-3 : CWFMDEVMAEMHKLTNSMFSCRWYDQNLKYRKALIIFMQRSQIAHPVLAGNIIPVSLETFTNIIKFAFS--------LFTLLNQLSHN- : 412
RzepOR2-3 : CWFMDEVMAEMRQLTTALFSCSWYEQSPRFRRDLIIFMQRSQKEDKIIAGNITAVSLETFRSVSENLC--------------------- : 391
            CW       E   LT a   C wy qn  fr  liifmqrsq      ag   p  l tf ni k   s          t
```

Figure S6-30　The alignment of OR2

```
                      10        20        30        40        50        60        70        80        90       100       110
BdorOR3-2-5 : -----MTSYENLPLYAVNVKVFVKVGLINSTDWTKGLLFFLILIVAYVGQIINLCKSWNEDIGETTMNFHCFLFVTHCLIRLWIVVKKKNKFERFFQCVEQWHR------ :  99
BdorOR3-2-6 : -----MTNYENLPLYAENVKVFVKAGFIDSNGLAKGILFCLMLIITYVGAIINLCKTWNEDIGETTMNFHCFLLVTHCLIRFCIVVKKKNKFERFFQCVEQWHR------ :  99
BdorOR3-2-2 : -----MTNYENLPLYAVNVKVFVKVGLIDSTGWTKGLLFCLILIITYVGAIINMCKTWYEDIGETTMNFHRLLLITHCLIRFCIIVKKKNKFERFFQCIEQWHR------ :  99
BdorOR3-2-4 : -----MTSYENLPLYAVNVKVFVKVGLIDSIGWTKRFLFCLIPIITYVGQIIHIFKSWNEDMGETSMNLHILLLKTHCLIRLWLMVKKPKDFERFFQCVEQWYR------ :  99
BlatOR3-2-4 : -----MTNYENLPLYGVNVKIFVKLGLIDSIGWTKRFLVCLIPIITYVGQIIHIFKSWDDDIGEMSMNLHILLFTTHSIVRLWLMVTKPKDFERFFLCVEQWYT------ :  99
BdorOR3-2-3 : -----MTSYENLPLYLMNVKVFVKMGLIDSSGWIKRFLYGLILITSFVGQMINVSKTWSEDIGDTSMNFYCLLLVTHCLIRFSIVVKKAEKFERFFQCIKQWYT------ :  99
BlatOR3-2-3 : -----MTSYENLPLYLMNVKVFVKMGLIDSSGWIKRFLYGLILIISFVGQMINFCKTWSEDIGDTSMNFYCLLLVTHCLIRFFIVVKKAHKFEWFFLCIKQWYT------ :  99
BoleOR3-2-2 : -----MTNYENLPLYAMNVKVFVKLGLIDSSDWTKRFLFSLILVITLVGLVVNMFKTWDEDIGETSMNIHKLLALTHCSIRCCIMVKKAKKFERLFQSIEQWYR------ :  99
BcucOR3-2X1 : -----MASYQNLPLYSVNIKAFVKLGLIESNNSTRRFLLGIIIIVTYIGQLTNMFRTWDVDIGETGMNFHVLALVTHYLLRFIIIVRKEKKFERLFQGIEPWYT------ :  99
BcucOR3-2X2 : -----MASYQNLPLYSVNIKAFVKLGLIESNNSTRRFLLGIIIIVTYIGQLTNMFRTWDVDIGETGMNFHVLALVTHYLLRFIIIVRKEKKFERLFQGIEPWYT------ :  99
BdorOR3-2-1 : -----MQNYKNLPLYSVNVKIFLQLGLIGRSTQTKQILLAFVPVATYLGQIINLYKTWGGVIGETGMNFYMLAHITHCLVRLLMVVRNNERFMCFLQSTDRWYE------ :  99
BoleOR3-2-1 : -----MQNYKNLPLYSINVKIFLQLGLIGPSTRAKQILLAFVPVTTYLGQIINLYKTWSDDIGETGLNFYMLALVTHCLL------------------------------ :  75
BminOR3-2   : -----MTSYQNMPLYSVNIKIFLKLGLIGSGARTMRVLLGLVLILTYSGQLINFCKTWNEDIGESGMNFHVVALIMHSVVRFFVVLKKAKKFERFFQRTEQWFT------ :  99
RzepOR3-2   : -----MLDYQNLELFSVNAKIFLKLRIIGTRSKKRQLLVALEPVLSIFGQILHLFMTRNESISETGLNFFFMAVVLNLVVRLFIVIHNGDKFVRLLRLVANWYH------ :  99
BdorOR3-1-1 : ---------------------MRRIGYFDQHR--LAWLYLISPIFLCLTAYYRTYVIRN-DFDEVIVNLFKISGATTTTVRAFIVMYKAKKFLSFFESIDEWYQGLQVRF :  86
BlatOR3-1   : -------------------------------------------------------------------------------------MYKAKKFLSFFESIDEWYQ------ :  19
BdorOR3-1-2 : ----------------------------------------------------------------------------MTIGRAFIVMCKSKKFLNFFESVDEWYQELHVRF :  34
BoleOR3-1   : -------MHYCGELFNWSLAFMRRVGYFDQHR--LAGLFLISPIFLCLTAYYRTYVIRN-DFDEVIVNLFKISGATVTTARTFIVMYKAQKFLNFFESIDKWYQELQVLF : 100
BcucOR3-1   : -------------------------------------------------------------------------------------MYKSQAFLDFFVFVEKWYE------ :  19
BminOR3-1   : ---------------------MKRVGYFDQHR--LASCLLTVPIFICVSSAYRTYIIRN-DFDEVTISLFKISGAYVTTARSLIVMYKAQEFLNFFDGIDRWYQ------ :  80
RzepOR3-1   : -------MYFCEQLYNINLVYMKRVGSLGDPK--RALFMLAMPTFCCFATIYRIYQIWH-NFDEVVANFFKVSGMITITIRTFMVLSKQKKFLDFFNDIDNWYH------ :  94
CcapOR3-1-1 : -------MLYSEEIYNWNLVFMRSIGYLGNRR--RAYFLLSLPVLICFGAIYGTYKMWS-DFDKVIINLFKTSGLLTVTLRSFVIIQKEKKLYDFFDYISQLYR------ :  94
CcapOR3-1-2 : MATERKSMPNVERLFNWNLEAWTRLGYLDKRKRLLACTIISAPIVVSMFVMPTFFMGVN-TFEQHIYNFYMIIVTSSSVARAILIIIKQRKILDLLNDMENWFV------ : 103
CcapOR3-1-3 : -------------------------------------------MIPPIFCN--LYLGHD-DLGEAIYDFFTAMIDITGLIRSIVILRKQRKFLNLFGVVESWYE------ :  58
                                       g                                         n            r      k   f   f     w

                     120       130       140       150       160       170       180       190       200       210       220
BdorOR3-2-5 : --------------------EIERNDDPQMVGILQEITKRTQLLSKMTIYVAAGGTLAAIFYPLSFD----GRKHMITMQFPHFDVLQTPFYEIFFLIGVTWLTPAFLVI : 185
BdorOR3-2-6 : --------------------EIERNDDPQMVGILKEITKRTQLWSKMTIYVAAGGTFAAIFYPLSFD----GRRHMITMQFPHFDVLQTPFYEIFFLLQVTWLTPATLAL : 185
BdorOR3-2-2 : --------------------EIERNDDPQMVGILQEITKRTQLLSKMTIYVAAGGTLAAIVYPLSFD----ERRHMITMQFPHFDVLQTPFYEIFFLMQVTWFTPAVLVL : 185
BdorOR3-2-4 : --------------------DIERNGDPQMVGTLKEITKRTQLLSKMTIYVAAGGTIAAFFYPLSFD----RRKHMITVQYPFVDALQTPFFEFLFLLQVLCLAPIILVL : 185
BlatOR3-2-4 : --------------------NIKRNDDPQMVGTLQKIIKRTQLVIKIAICVAAGGTIAAFFYPLSFD----GHKHMVSVQYPFFDALQTPFYEFLFLIQVLCLAPINVIL : 185
BdorOR3-2-3 : --------------------DIEREGDPQMVGTIQEITRKTQKLSKVTIYVAALATLAAFCYPLSFD----ERKHMIEVQYLFFDILQTPYYELFYLMEVVLVTPTILVL : 185
BlatOR3-2-3 : --------------------NIELKGDPQMVGTLQEITIKTQKLSKITIYVAALATISAFLYPVSFD----ECKHMIEVQYLFFDTLQTPFYELFYLMQVVLVTPTILVL : 185
BoleOR3-2-2 : --------------------DIERNGDPEILSTLQEINRKTDKLSKMTIYLAAMAALAGLLYPLSYN----TRTHMVTVQYPFIDALQTPFFEFFFLIQVLWLTPTVLVI : 185
BcucOR3-2X1 : --------------------DIERHGDPHIVSILQKITQKTQRLTRLSFYASVVATLATFIYSLSFD----ERRLLVTVQYPFFDVLQTPFFEFFFLIQMVWLVPTSLLV : 185
BcucOR3-2X2 : --------------------DIERHGDPHIVSILQKITQKTQRLTRLSFYASVVATLATFIYSLSFD----ERRLLVTVQYPFFDVLQTPFFEFFFLIQMVWLVPTSLLV : 185
BdorOR3-2-1 : --------------------DIELNSDAEVVLMLQDVTTHTHKLTRIGFYTITIGALFSYIYPFSFE----ERKFILHIHYIFFDAKQTPFYEFFFLLQALVLVPTFVFV : 185
BoleOR3-2-1 : ------------------------NSDPEVVRMLQDVTTHTQKLTRIGFYTILTGGLCSYIFPFLFE----ERKFILDIHYIFFDAKQTPFYEFFLVLQIVVLVPVFIVI : 157
BminOR3-2   : --------------------DIEQNSDPGVVRMLQDVITHARKVTRIGFYTSIIGALCAYIYPFSFE----ERKFILDIDYFFFDAKQTPFYEFFFLLQALILVPVFIFV : 185
RzepOR3-2   : --------------------EIERDANPEVLRKLQDVNKHARKLTRISIFAACTGMLFAYTFPFSFD----DRKLIVEVSYSFFDTKQSPFYEFFFLLQALVLVPIFCCV : 185
BdorOR3-1-1 : AGVSSLFLFICYSLTFL---HFQREDDDVTLKKAHEFTKKIKKTSKTVLILAGITLFYVIFIQLLATAGVGYKKLLVDVAFPGVDLYESPLWEMMSILQSLWIAPIVLFS : 193
BlatOR3-1   : --------------------ELQREDDDVTLKKAHEYTQKIKKTSKTVLILAGITLFYIAFIQLLATVGVGYKKLLVDVAFPGVDLYESPLWEMMSILQSLWIAPIVLFS : 109
BdorOR3-1-2 : AGGSSLFLFIYYLLTFL---HFQREGDDVTLKKAHEYTKKIKKTSKTVLILTGITIFYIMFVQLLSTAGVGYKKLILDVAFPGVDFYESPLWEMMSILQGLWTAPIVIVS : 141
BoleOR3-1   : ANRKSNFMFIYYLLTFLFNLYFQCEGNEITLNKAHEYTRKIKKATKTVLILTSITLFYVIFIQLLATVGVGYKKLLVDVAFPGIDLYKSPFWEIMSIMQSLCIAPITLFS : 210
BcucOR3-1   : --------------------EQQRDGEEVTLRKTHEFTQKIRKAAKTLLIVTMIILSYMVLIQLLATVGIGYRKLIVDVAFPGIDFYISPNWEMMSFLQCLYVAPFTFVS : 109
BminOR3-1   : --------------------ELQCDGNEITLKKVHEFMRIVKKASKTILILTALTLFYITVIQLLATADYEHK------------------------------------- : 133
RzepOR3-1   : --------------------RLRSENDEVTMKNLEEYTRKTRRASKTIFGIMIVCIFYISAIQLLTSISSTNKKYIIEVRLPFVDLYTSPYYEIISVLQALWLAPIVLLS : 184
CcapOR3-1-1 : --------------------ELQAEGDETTLKRMHEFARKTKRYTKGLFILMIACTFYISLIQVMSTFGIGLKKFLIEMELPFISVNENPYWDIFSTLQAVWLAPSILLS : 184
CcapOR3-1-2 : --------------------EVQEQNDNNALETLNKLTQKVRRYSKYTLWWVIIFGAFLSFQPICT----GYGKFVYDTQIPGIDLHQSPLYEIMYGFQSLWVIPMACVS : 189
CcapOR3-1-3 : --------------------DLKQPNEHKALETLNQIVAKVQLYSKCCLYSLLMVDVTYAFEPIIT----HYGKLVVELQLPSIDLHQSPVYEMVYLIQALWLVPLTSVN : 144
                                        d                                                         d    p  e     q     p

                     230       240       250       260       270       280       290       300       310       320       330
BdorOR3-2-5 : SLPFTNIFLISLMFGELVLKDLCVKLRNIRSE-NEETMLQEFKECIAYHQKIIDLCDDLQDLLSIDGFFHLALFGMMLCMLLFFLSMI---------------------- : 272
BdorOR3-2-6 : SLPFTNIFLISLMF------------------------------------------------------------------------------------------------ : 199
BdorOR3-2-2 : SLPFTNIFLISLMFGELVLKDLCVKLRNIRSE-NEETMLQEFKKCIAYHRKIVDWCAD---------------------------------------------------- : 242
BdorOR3-2-4 : TLPFTNIYLISLMFGELVLKDLCVKLRNIRSE-NEETMLQEFKKCIAYHQKIIALCDDLQDLLSMDGFFHVALFGMMLCMLLFFLSMI---------------------- : 272
BlatOR3-2-4 : TLPLTNIYLISLMFGELVLKDLCVKLRNIRSE-NEEIMLQEFKQCIAYHRKIIDWCDDLQDLLSMDGFFHVALFGMMLCMLLFFLSMI---------------------- : 272
BdorOR3-2-3 : YLPFTNILLISLMFGELALKDLCVKLRNIRSE-NEETMLQEFKECIEYHGKVVDMCDDLEDLISIDGFFHLALFGMILCMLLFVLSVV---------------------- : 272
BlatOR3-2-3 : YLPFTNILLISLMFGELVLKDLCVKLRNIGSE-NEETMLQDFKECLGYHGKVVDLCDDLEDLISVDGFFHVALFGMILCMLLFVLSVV---------------------- : 272
BoleOR3-2-2 : SLPFMNIFLITFTFGVLALKDLCGKLRNIRSE-NEETMLQEFKECIVYHRKVIKLCDDLEDLISIDGFFNLALFGMMLCILLFFLSMI---------------------- : 272
BcucOR3-2X1 : YLSFTNIFLTSLMFGELILKDLCLKLRNIRSE-NEMTMLKEFKDCIAYHNKIIDLCGDIEDLISMDAFFHVTSFGMMLCMLLFFLSMI---------------------- : 272
BcucOR3-2X2 : YLSFTNIFLTSLMFGELILKDLCLKLRNIRSE-NEMTMLKEFKDCIAYHNKIIDLCGDIEDLISMDAFFHVTSFGMMLCMLLFFLSMI---------------------- : 272
BdorOR3-2-1 : YLPFTNILLTSLKFG-------------------EVHDLR----------------------------------LILSALAFIS-------------------------- : 216
BoleOR3-2-1 : YLPFTNILLTSLKFGELILMDMCTKLKNINNQ-DETTQLREFKECISYHEKIITFRDDLEYLVSIDGFFHVTLFGLMLCMLLFFL------------------------- : 241
BminOR3-2   : YLPFANFLLMSLKFGEVILMDLCTKLRNISNQ-DEATQLRELKECISYHEKIITFRNDLEYLVSIDGFFHIALFSLMLCMLLFFLSLV---------------------- : 272
RzepOR3-2   : YLAFTNLFLAFLMFGEVVLLDLRVRLDSISSSGNKAAMLHELKECIAYHRKIISYRNDLDNLVKVANFFDVALFGLMFCMMLFFMSLV---------------------- : 273
BdorOR3-1-1 : YVSYLCLTLIAIAFGIFLMKNLQSKLEGMNEM-TDEEALKCIKKCVKDHVMIIKYHRDLEVLFSVGSFADVCIFAIIPCVIIVISTM----------------------- : 279
BlatOR3-1   : YVSYLCLTLIAIAFGIFLMKNLQTKLEGINEK-TDEEALKFIKKCVKDHVMIIKYHQDLEVLFSVGSFADVCIFAIIPCVIIVISTM----------------------- : 195
BdorOR3-1-2 : YVSYLCLTLIAIAFGIFLMKNLQSKLEGMNEM-TDEEALKCIKKCVKDHVMIIKYHRDLEVLFSVNSFADVCIFAVIPCVIIIISTM----------------------- : 227
BoleOR3-1   : YVSYFCLFLIAIAFGIFLMKDLQSKLESMNDM-TDVEALECINKCVKDHIMIIKYHNDLEVLFSVGSSADVCIFGIIPCVIIVFSTM----------------------- : 296
BcucOR3-1   : YVSYLCLTLIAISFGIFLMKDLQFKLENMNDM-TDLEALNCIKKCVKAHVMIIKYHNHLEALFSVGSFADVCIFGIIPCVIIVLSTM----------------------- : 195
BminOR3-1   : ----------SISFGTFLIKDLQYKLESTTDM-TELEALKCIKKCVKDHVMIIKYHNDLEVLFSSGSSVSVSIFGITPCVIIVFSTMVSSNRSNLFLCYIFKFKFTIILH : 232
RzepOR3-1   : YVSYLCVILISISFGIFLMKDLQQKLGNMHEL-NELEGLECIKKCIQQHVLIIKFHRDLEVLFSAGNFVDVSIFCIIPCVIIVYANM----------------------- : 270
CcapOR3-1-1 : YISYLCIIFTTISFGILLMKDLQFKLGNMNEK-NDLEAYEYIKNCVKQHVMIIKYHRQMEVLFSLGSCAEVCNFCIIPCVIIVYSTM----------------------- : 270
CcapOR3-1-2 : SISYADTLLIFISFGIFATKQLQRKLKDISQM-DEQQGLENIKKCVQYHWKIIKFGEDLENAYSLMCLLDFSLYCVTLCMLLFYSVM----------------------- : 275
CcapOR3-1-3 : YVSYSNSLLIFTIFGVFATRHLQQKLMEISQM-EDDEALANLKQCVVYHSKIIKFGENLEELYSLMSLLDISLYCISVCLMLVYLTM----------------------- : 230
                      l    Fg      l  kl            l   k c   h  ii    d   l s         f    c

                     340       350       360       370       380       390       400       410       420       430
BdorOR3-2-5 : --HDLRLILAALSFVSFTTYMLFITYYYANKLVTE-SLEVANAAYDTPWYRGNLEMRKCVITMIARCQKPLQMTAGGIYPMTMETFQAILRVSYSYFSLLQGLNQQ--- : 375
BdorOR3-2-6 : ---------------------------------------VANAAYDTPWYRGNLEMRRCVITMIARCQKPLQMTAGGIYPMTMETFQAILRVSYSYFSLLQGLNQQ--- : 266
BdorOR3-2-2 : --------------------------------------LQDLLSMDGFFHLALFGMMLCILLFFLSLIHDLRLFLAALSFVSFTTYIISITYYYANKLATEVLHAT--- : 310
BdorOR3-2-4 : --HDLRLILTILTFVSYTTYMLFTTYYYANNLATE-SLEVANAAYDTPWYRGNLEMRKCVITMIARCQKPLQMKAGGLYPMTMETFQAILRVSYSYFSLLQGLSQQ--- : 375
BlatOR3-2-4 : --HDLRLIVTILTFVSYTTYMLFSTYYYANKLATA-SLEVANAAYDTPWYRGNLEMRKCVITMIARCQKPLQMNAGGLYPMTMETFQAVLRVSYSYFSLLQGLSQQ--- : 375
BdorOR3-2-3 : --HNLRLILMVFVFVSFNIYMIGITYYYANNLATE-SLEVANAIYDTPWYRGNLEMRKSVITMIARCQKPLQMKAGGLYPMNMETFQAILRVSYSYFSLLQGLNQ---- : 374
BlatOR3-2-3 : --HNLRLILMVFVFVSFNIYMIGITYYYANNLATE-SLEVANAIYNTPWYRGNLEMRKCVITMIAKCQKPLQMKAGGLYPMNMETFQAILRVSYSYFSLLQGLNQ---- : 374
BoleOR3-2-2 : --DDLRLILVGLVFVSLNTYMIGITYYYANNLATE-SVEVANAAYDTPWYMGNLEMRKCVTIMIARCQKPIQITAGGLYPMNMENFQAILRISYSYFSLLQGLSQQ--- : 375
BcucOR3-2X1 : --DDLELIPAVLVMMGFDMYLIGFSYYYANNLATE-SLEVANAAYDTPWYRGNLEMRKCVLIMIARSQNPLQITAGGLYPLTMENFQAILRISYSYFSLLQGVSQQ--- : 375
BcucOR3-2X2 : --DDLELIPAVLVMMGFDMYLIGFSYYYANNLATE-SLEVANAAYDTPWYRGNLEMRKCVLIMIARSQNPLQITAGGLYPLTMENFQAILRISYSYFSLLQGVSQQ--- : 375
BdorOR3-2-1 : ----------------FNFYVIGITHYYANNFSNETTLKRAYAAYDTPWYEGNMELRKCVQIMIARSHKPLEIKSSGLYPMTLENFQAILRISYSYFSMLXGFNQQ--- : 306
BoleOR3-2-1 : ----------------SLMFFIFVSAYTLYILLLMQSLKVAYAAYDTPWYEGNPELRKCVQIMIARSHKPLEIKAGGLLPMTLENFQAILRISYSYFSMLQGFSQK--- : 331
BminOR3-2   : --HDIRLILTALAFISFHIYIIGITYYYADNFSNE-SLKVAYAAYDTPWYEGNSELRKCVQVMIARSHRPLEIKSGGLYPMTLENFQAILRISYSYFSMLQGFNQQ--- : 375
RzepOR3-2   : --KDIQLVLSAVVFISFPVYVIAISYYYANKFTNE-SIQICNAAYNTPWYEGNLEMRKCVLIMIARSQRPLYITVMGMYPMTLETFQAIIRISYSYFSLLQGLSQQ--- : 376
BdorOR3-1-1 : --DHDMSLLIADIQLSLMVMISTFIIFWVANNFCYESANIAKAAYNCNWENRNKEFRKYIPLIIITSQRPLQLTAGGLKPINMEFFLTMVRCTYSLFTVLFTMKTEGDS : 386
BlatOR3-1   : --DHDMSLLIADIQLSLIVMISTFIIFWVXNNFCYESENIAKAAYNCNWESRNKEFRKYIPLIIMASQRQLQLTAGGLKPLNMEFFLTMVRCTYSLFTVLFTMKTEGDS : 302
BdorOR3-1-2 : --DHDMSMLIGDIQLSIMVMISTFLVFWVGNNFCYENENIAKAAYNCNWENRNKEFRKYIPLIIITSQRPLQLTAGGLKPINMEFFLTIVRCTYSFFTVLFTMTTEGDS : 334
BoleOR3-1   : --DHDMSLLIADIQLSLMVMISTFIIFWVANNFCCESEKIANAAYNCNWEDRNKEFRKYIPLIIIISQRPLQLTAGGLKPINMEFFLTMVRCTYSLFTVLITMKTEGDS : 403
BcucOR3-1   : --DHDISMLIADIQMAAMVMTSTFIFFWVANNFVIESENIANAAYNCNWVDRDKEFRKYIPLIIGNSQKPLQLTAGGIKPINMEFFLTIVRCTYSLFTVLFTMKTGGDS : 302
BminOR3-1   : LQDHDMSLLIADIQLSLLVMISTFIFFWVANDFCCEV------------------IWDYFPHIYN-------------------------ICYYNL------------- : 285
RzepOR3-1   : --EYNLAFMITDVQLVFVVIFSTYIIFWLANSFYIEGLNIAHAAYNCNWVDRGKDFRKYIVVIMTVGQKPLELTAGGLKPVNMQFFLAIVRVSYSIFTVLQGTKKDY-- : 375
CcapOR3-1-1 : --DYDLAFLMTDIQLAVIAVSSTFFNFWLANNFCVESLNIAYAAYNSNWIDRNKEFRKYIVLIMTMSQKPLQLTAAGLKPINMEFFLAILRAAYSLFTVLQ-------- : 369
CcapOR3-1-2 : --DFTWALMFQAVVVELILTLLIFLTTFLADIFTQESLNVAQTAYDMNWLQRDKAFRVAVLLIILRSQRPLILTAGGIQPLNLETFLAIMRSSYSFFSVLRGVM----- : 377
CcapOR3-1-3 : --DFTWPLLFKGVIVILFLTTLIFLTYHVADVLTHESMNIAELAYNTNWMDRDKEFRSCIQVIIVRSQRPLMLTAGGFQPMNMKTFLAIMRASYSFFSVLRSTV----- : 332
                                                      a aay   w     e rk     i   q pl   agg  p   e f    r  Ys f  l
```

Figure S6-31 The alignment of OR3

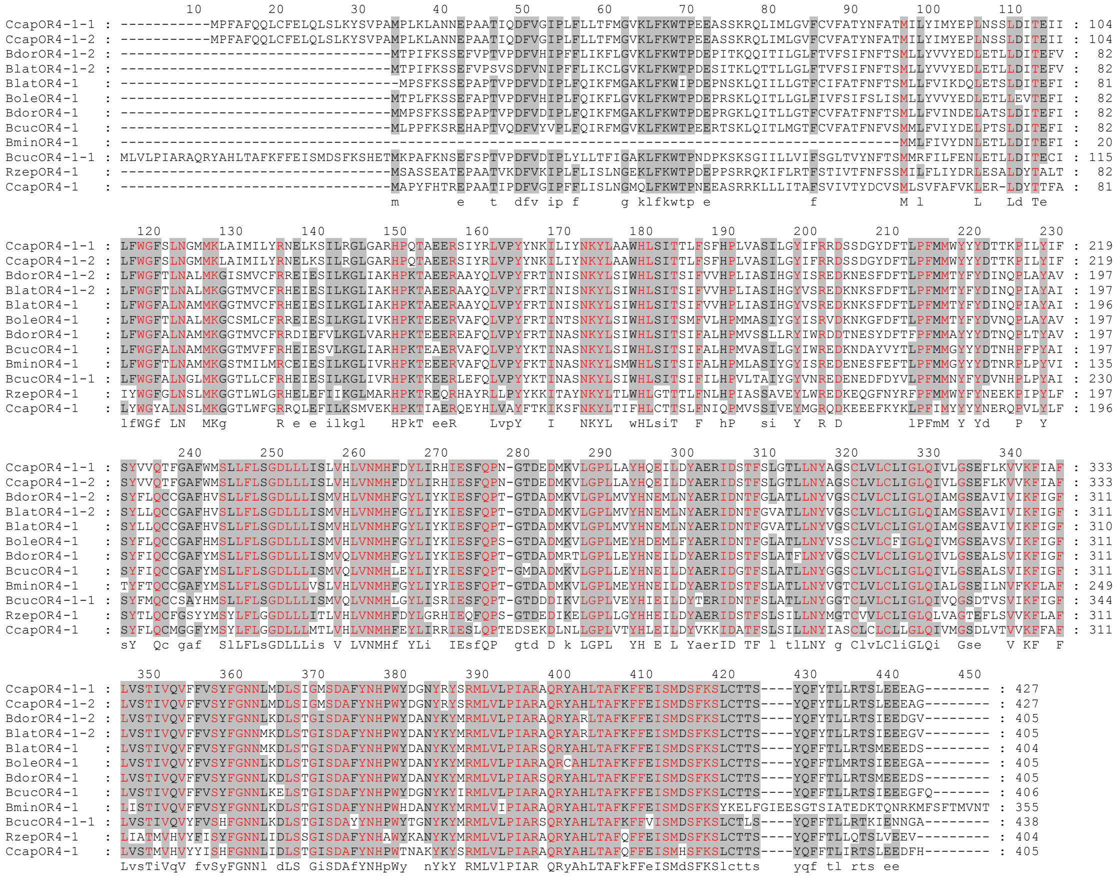

Figure S6-32 The alignment of OR4

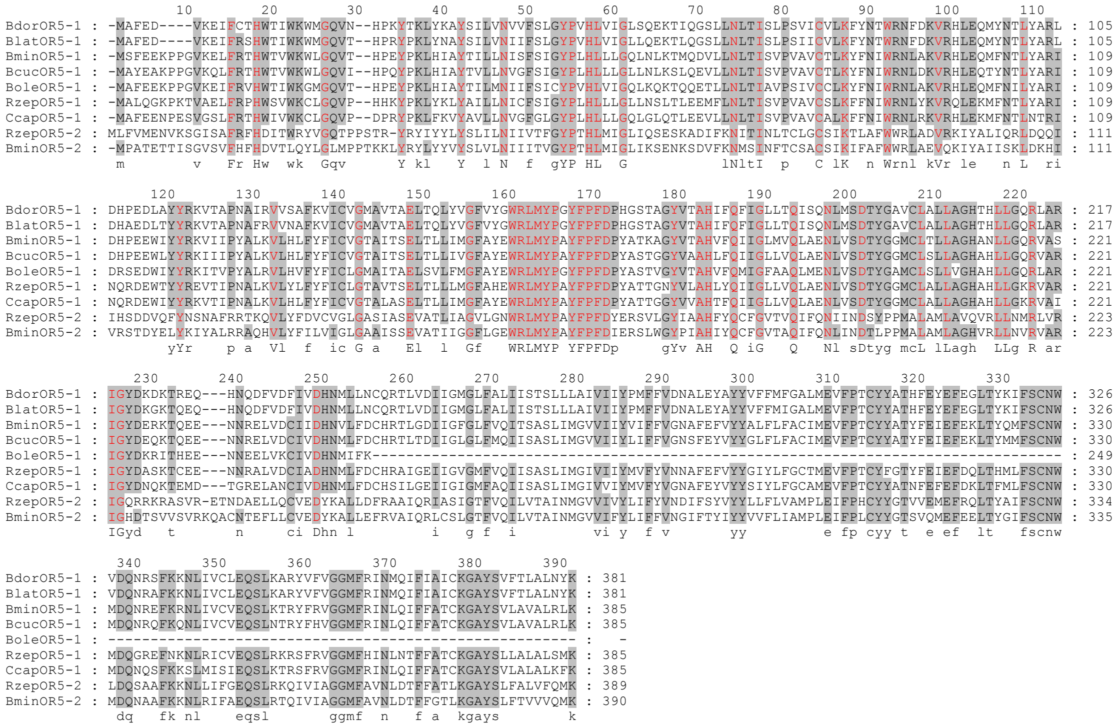

Figure S6-33 The alignment of OR5

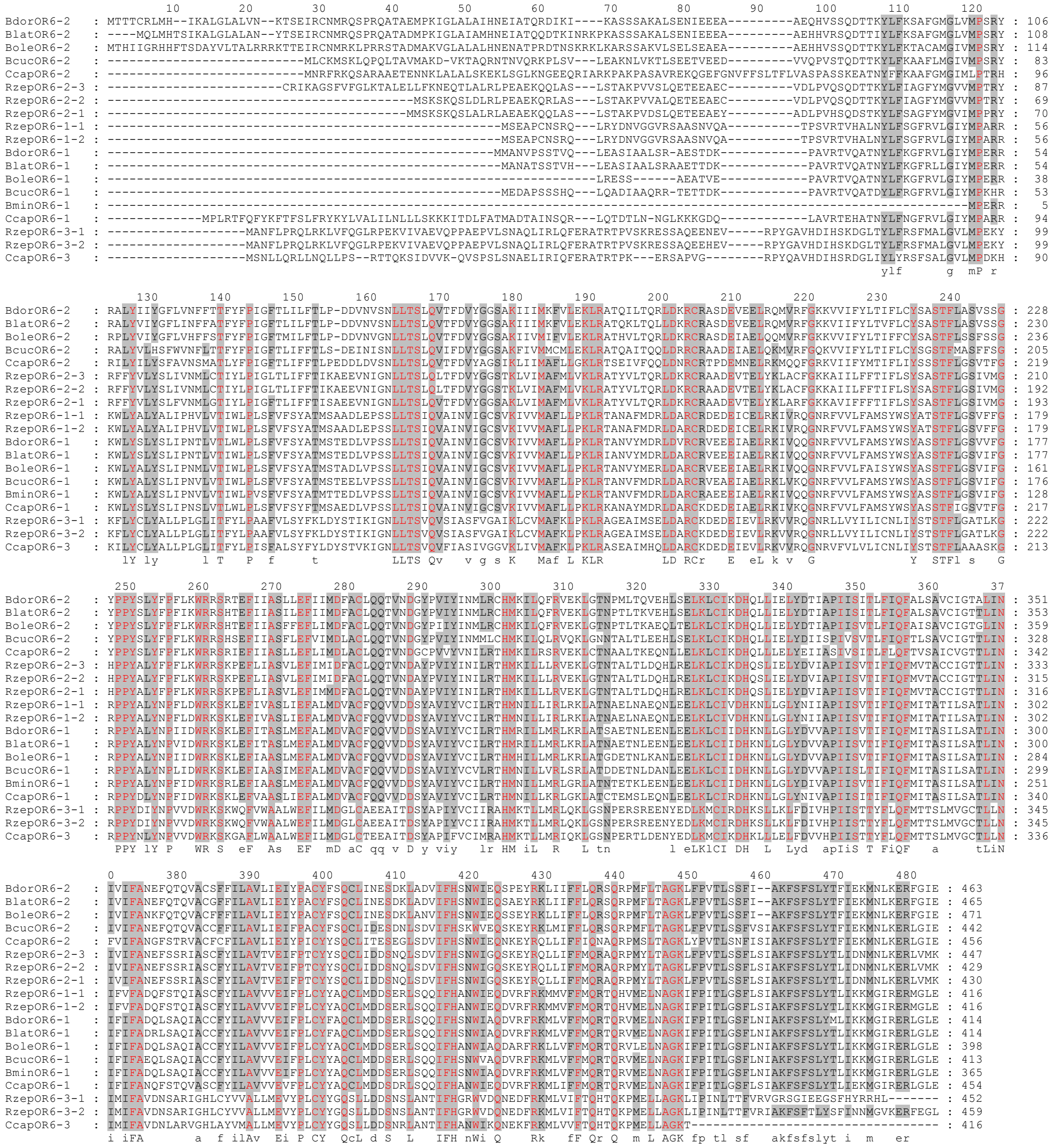

Figure S6-34   The alignment of OR6

```
                      10        20        30        40        50        60        70        80        90       100       110
BdorOR7-1   : MSKILLVRSATVYKSRDALTYLFNVFTFMGTNPLENRSQRYYRLYHFYSFTVNFICCLFCPLSFHIGYIKLRHVLTNSQLLAAIQNAVQVSGIPIKILVITWYMKRLRHA : 110
BlatOR7-1   : MSKILRFRSAIVYKSRDALTYLFNIFTFVGTNPLENRSQRYYRLYYFYSFTVNFICCLFCPLSFHIGYIKLRHVLTNSQLLAAIQNAVQVSGIPIKILVITWYMKRLRHA : 110
BminOR7-1   : MSKILRVSRAKIYKSRDAVSYLFNIFTFLGTNPLEHRSRRYYFLYYFYSFTLNFISLLYCPLSFHIGYIKLTHVLTNSQLLTAIQNAIQVSGIPIKVVAITWYMKRLQHA : 110
BcucOR7-1   : MPNLIRVGGACIYKSRDSLTYLFKIFTFVGINPSEQQSRKYYWLYYSYSLTVNFICCLFSPLSFHIGYIKLWHVLNNNQLLAAIQNAVQVTGIPIKILVITWYMKRLRQA : 110
CcapOR7-1-1 : MLDLLRGRGRRVYKSRHALTYLFNVFTFVGTNPGKTRTHKYYTLYYTYSLTVNFICCLFCPLSFHIGYIKSWHLLNTTELLAAIQNAVQVTGIPIKIFFITWNMRRLQSV : 110
RzepOR7-1   : MLDLLRGRGLEAYKSRDALTYLFNIFTLVGTNPQGQQSNKYYYLYYLYSVTVNFICCFFSPLSFHIGYIKYLNVLSTTELLAAIQNAVQVTGIPVKIIVITWHMKSLLSA : 110
CcapOR7-1-2 : MSDLLFGRGQIVYKSRHALTYLFNIFTFVGTNPLGGQSYAYYSLYYLYSLLVNFVCCIFCPISFHIGYIKLLNVLNTNELLSAIQNAIQVSGIPIKIIVIAWYMKRLQSV : 110
              M   l        YKSR altYLFn FTf GtNP    s  YY LYy YS tvNFicc f PlSFHIGYIK   vL    LL AIQNA QV GIPiKi  ItW MkrL

                     120       130       140       150       160       170       180       190       200       210       220
BdorOR7-1   : FEILDELDVNYTRREDLAKIRECVRRCKKIVLIFCFPYYSFELTTIALGVAQNRAPLAAWVPFLDGQR-AAWEYWTIVLWDAFVMFFLLCHQLGSDTYPPIFINIIRTHV : 219
BlatOR7-1   : FDILDELDVNYTRREDLAKIRECVRRCRKIVLIFCFPYYSFELSTIALGVVQNRAPLAAWVPFLNGQR-AAWEYWTIVLWDAFVMFFLLCHQLGSDTYPPIFINIIRTHI : 219
BminOR7-1   : VEILDELDVNYTQREDLAKIRECVRRCRKVILLFCLPYYSFGISTIALGVLQNQAPLTVWVPFLDGKR-AAWEYWTIVLWDTLVMFILLCHQLGNDTYPPIFIKVISTHM : 219
BcucOR7-1   : FKILDQLDVNYTQHEDLAKIRECVRRCRKIVLIFCLPYYSFELSTIALGLLQKRAPLAAWVPFLDGQR-AAWEYWTIVLWDTFVMFILLSYQLGGDTYPLIFLNIIRTHV : 219
CcapOR7-1-1 : IPILDELDENYKSAKDLLKIRKCVRGCCKMIGFFCLPYYSYEITTIALGVWQNRAPLAAWVPYLDGQR-AAWEYWTIVVWDIFVMFFLLSHQLGSDTYPPIYINIIRTHV : 219
RzepOR7-1   : REILDELDANYRRADDLLRIRQCVRRCCKIIVIFCVPYYSFEITTIAFGVLQHRVPLAAWVPYLDAQGGAEWQYWTIVVWDTFVMFILLTHQLASDTYPPVYISIIRTHI : 220
CcapOR7-1-2 : CEILDKLDENYKRAEDLNSIRRCVRSCCKIIAAFCVPYYGFELSTIAFGVYQNRAPLTIWLPYFDATR-TTWEYWTHVGWDVFIMLFLLAHQLGSDTYPPVFISVIRLHM : 219
                ILD LD NY    DL  IR CVR C K    FC PYYsfe  TIA Gv Q raPL  WvP ld  r a WeYWTiV WD fvMf LL hQLg DTYPp  i  IrtH

                     230       240       250       260       270       280       290       300       310       320       330
BdorOR7-1   : QLLIARVNRLGRTGALTADEHYEELLGCIRTHVQIVSIAKIVAPVISVTLFTQFATTATTLLNWLGNVEYPENIISLAFFSCQLLQILPCCSSASQLIADCERLP----- : 324
BlatOR7-1   : QLLITRVNRLGRAGALTADEHYEELLGCIRTHVQIVSIAKIVAPVISVTLFTQFATTATTLLNWLGNVEYPENIISLAFFSCQLLQILPCCSSASQLIADCERLP----- : 324
BminOR7-1   : QLLVTRVNRLGRPGALTADKHYEELL-----------ASKIVAPVISVTLFTQFATTATTLLNWFGDMEYPENIISLAFFCCQLVQILPCCLSASQLIADCERLP----- : 313
BcucOR7-1   : QLLVTRVSRLGRDGALSADEHYAELLACIRTHVQIVSIANIVAPVISVTLFTQFATTATTLLTWFGNVEYPENIISFAFFSCQLLQIFPCCSSASQLIADCERLP----- : 324
CcapOR7-1-1 : QLLVERVELLGSDKTKSAEEHYAELLGCIRTHGQIKRIVNLIAPVISVTLFTQFATSATTLLNWFGDVEFPENIISFAYFTCLTLQILPCCSSASYLISDCELLP----- : 324
RzepOR7-1   : QLLVERAKRLGGDRMLNADENYAELLACIRTHCQILRLARIVAPVISITLFTQFATTAITVLNWFGNVEFPENIISFAFFSCQLMQILPCCYCASLLIDDCDQLP----- : 325
CcapOR7-1-2 : QLLVERVKRLGTNKALCREKRYAELLVCINTYGQILSIANIVAPVISITLFTQFATTATTVLNWFGNMKFPDNIIPMAFFSCQIMQILPCCYYASQLIADWCFLMTHHSL : 329
              QLL  Rv rLG    l a   Y ELL ci t  qi     ivAPVIS TLFTQFATtAtT LnW G  e PeNIIs AfF Cq  QIlPCC  AS LI Dc  Lp

                     340       350       360       370       380       390       400       410       420       430
BdorOR7-1   : --------------------DAIFHCNWVDQDRRFRRAMLFFLQRAQNPIRFSCLKLFNVKLETS------VAIGKFAFSLYT--LIEETKVGTDTEN----- : 394
BlatOR7-1   : --------------------DAIFHCNWVDLDRRFRRAILFFLQRTQNPIRFSCLKLFDVKLETS------VAIGKFAFSLYT--LIEETTVGTNTDN----- : 394
BminOR7-1   : --------------------DAIFHCNWMDQDRRFRRAILFFLQRTQTPIRFSCLKLFDANLETS------VAIGKFAFSLYT--LIHEAEDGGKTDN----- : 383
BcucOR7-1   : --------------------DAIFHCNWVDQDRRFRRAILFFLQRTQKPMRFWCLKLFVVKLETS------VAIGKFAFSLYT--FIQESDVGRKSNN----- : 394
CcapOR7-1-1 : --------------------NAIFHCNWIERDRRFRKTLLFFLQRTQTPLRFSCLKLFVVKLETS------VAIGKFAFSLYT--LIQGTEVGGKTEN----- : 394
RzepOR7-1   : --------------------NAIFHSNWMDQNRRYRKTILFFIQRTQNPIRFSCLKLFGVNLATG------MAIGKFAFSLYT--FIKESKVGDKLDK----- : 395
CcapOR7-1-2 : RPIFEQHYRPAVGSVIRATIDQLYTVLTLLDNARPPSAVIATVEQNIKENPNEYIHYREAQLEPCSSTFCKILLKNLGLRTYKTQLVQESKQNDQLERCTSAK : 432
                                   aifh nw    rR r   lff qr q p rf clklf   Let        aigkfafslYt   i e   g
```

Figure S6-35 The alignment of OR7

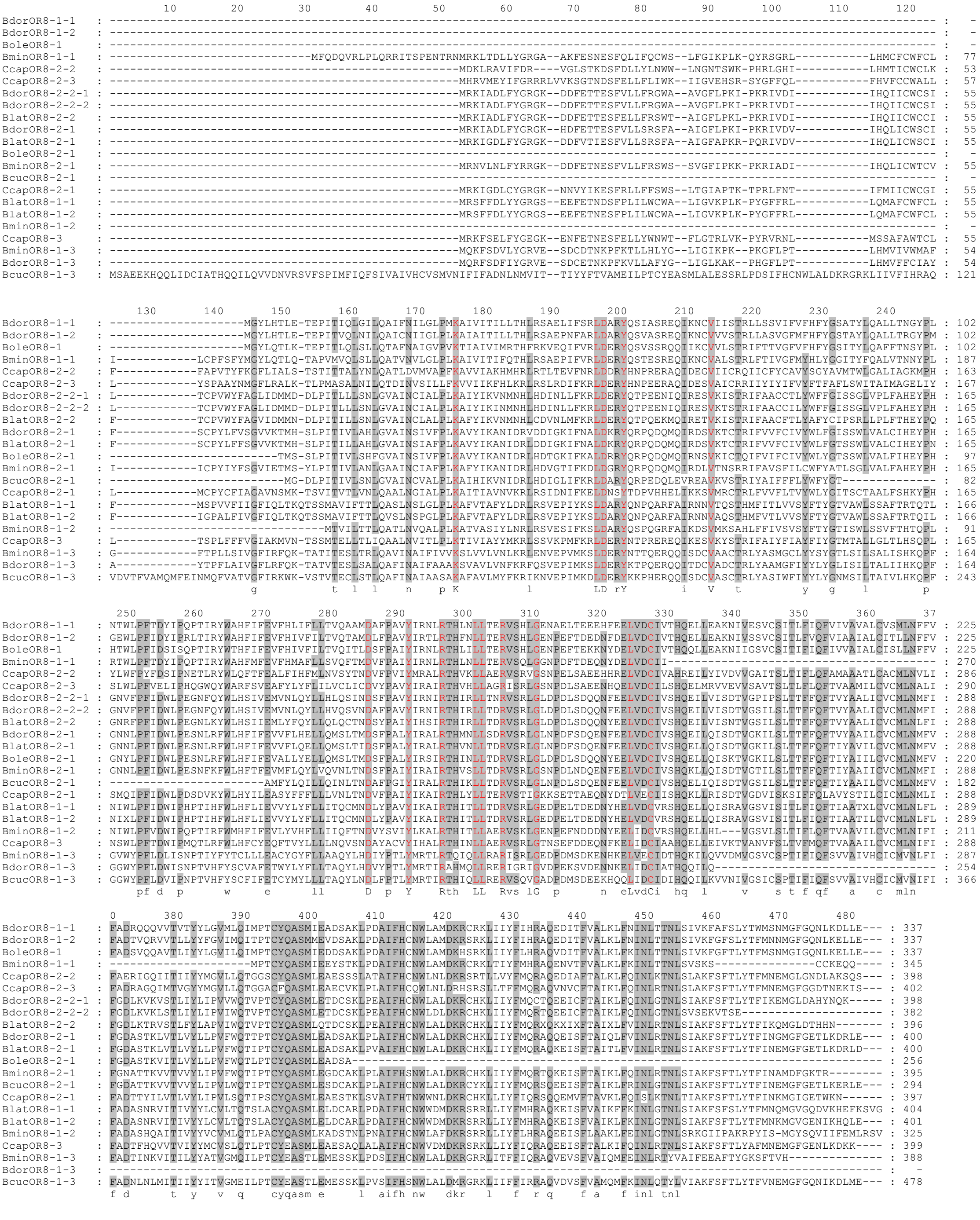

Figure S6-36 The alignment of OR8

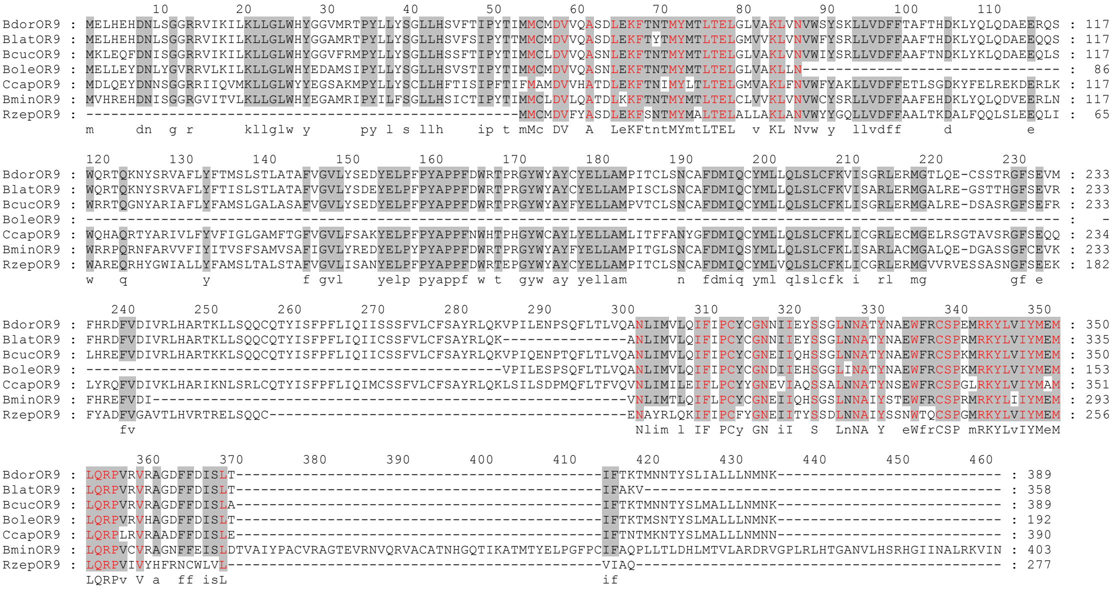

Figure S6-37　The alignment of OR9

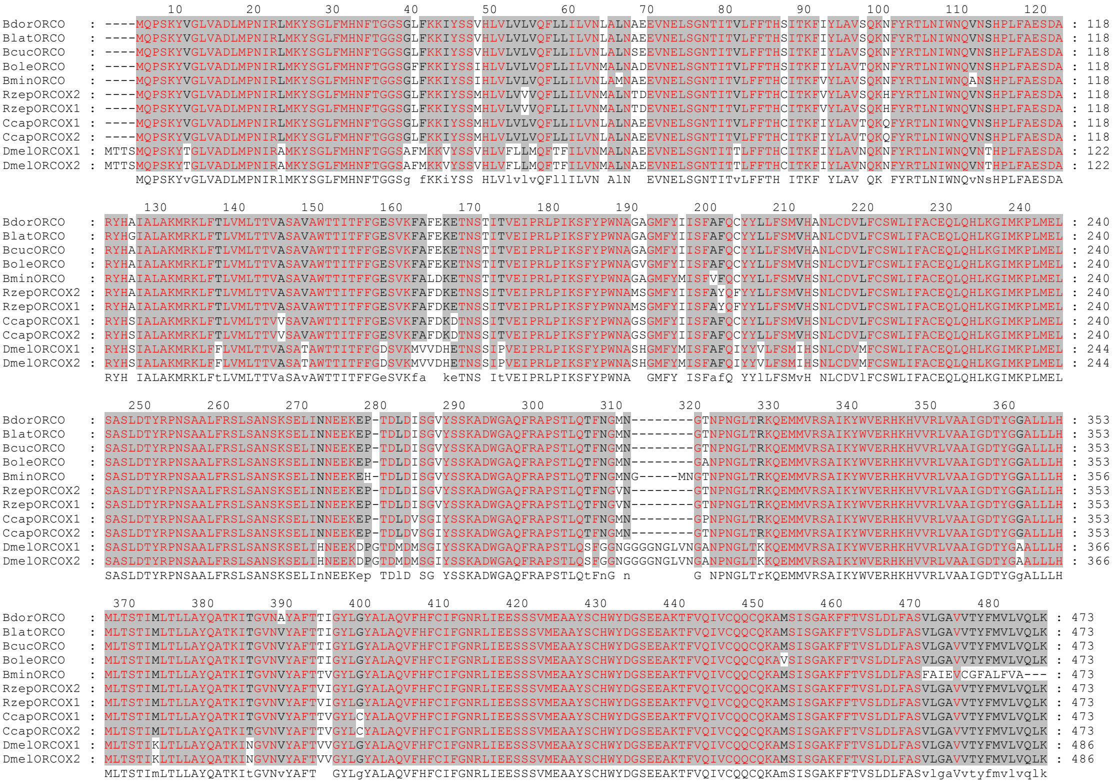

Figure S6-38　The alignment of ORCO
