# Supplementary material for: Comparative genomic and transcriptomic analyses of chemosensory genes in the citrus fruit fly Bactrocera (Tetradacus) minax
Source: Sci Rep. 2020 Oct 22;10:18068. doi: 10.1038/s41598-020-74803-5 (PMC7583261; doi:10.1038/s41598-020-74803-5)
Supplement: Supplementary file 8 — Supplementary Information 8. [file 41598_2020_74803_MOESM8_ESM.pdf]

Supplementary file 7

Table S7-1 The sequence similarity of CSP1

	BlatCSP1X3	BlatCSP1X1	BdorCSP1X1	BdorCSP1X2	BoleCSP1X2	BoleCSP1X3	BoleCSP1X1	BcucCSP1	BminCSP1	RzepCSP1X2	RzepCSP1X3	RzepCSP1X1	CcapCSP1	DmelCSP1
BlatCSP1X2	100%	100%	97%	97%	92%	92%	92%	94%	91%	85%	85%	85%	87%	64%
BlatCSP1X3		100%	97%	97%	92%	92%	92%	94%	91%	85%	85%	85%	87%	64%
BlatCSP1X1			97%	97%	92%	92%	92%	94%	91%	85%	85%	85%	87%	64%
BdorCSP1X1				100%	93%	93%	93%	96%	91%	87%	87%	87%	89%	65%
BdorCSP1X2					93%	93%	93%	96%	91%	87%	87%	87%	89%	65%
BoleCSP1X2							100%	96%	92%	87%	87%	87%	85%	65%
BoleCSP1X3							100%	96%	92%	87%	87%	87%	85%	65%
BoleCSP1X1								96%	92%	87%	87%	87%	85%	65%
BcucCSP1									93%	87%	87%	87%	87%	67%
BminCSP1										84%	84%	84%	84%	63%
RzepCSP1X2											100%	100%	85%	63%
RzepCSP1X3												100%	85%	63%
RzepCSP1X1													85%	63%
CcapCSP1														63%

Table S7-2 The sequence similarity of CSP2

	CcapCSP2X2	BcucCSP2	BminCSP2	BoleCSP2	BdorCSP2	BlatCSP2	RzepCSP2X1	RzepCSP2X2	DmelCSP2
CcapCSP2X1	100%	92%	93%	92%	92%	89%	89%	89%	73%
CcapCSP2X2		92%	93%	92%	92%	89%	89%	89%	73%
BcucCSP2			92%	91%	92%	89%	87%	87%	74%
BminCSP2				97%	96%	94%	87%	87%	74%
BoleCSP2					95%	93%	87%	87%	74%
BdorCSP2						96%	88%	88%	74%
BlatCSP2							88%	88%	71%
RzepCSP2X1								100%	73%
RzepCSP2X2									73%

Table S7-3 The sequence similarity of CSP3

	BlatCSP3X1	BoleCSP3X1	BcucCSP3X1	BminCSP3X1	CcapCSP3X1	RzepCSP3X1	DmelCSP3	BdorCSP3X2	BlatCSP3X2	BoleCSP3X2	BcucCSP3X2	BminCSP3X2	RzepCSP3X2	CcapCSP3X2
BdorCSP3X1	93%	91%	89%	87%	75%	76%	52%	53%	50%	48%	48%	46%	43%	41%
BlatCSP3X1		90%	89%	87%	76%	77%	51%	50%	53%	47%	49%	47%	45%	41%
BoleCSP3X1			87%	90%	76%	77%	50%	48%	47%	53%	47%	48%	43%	41%
BcucCSP3X1				88%	76%	75%	52%	48%	48%	47%	53%	47%	41%	41%
BminCSP3X1					72%	74%	51%	45%	46%	47%	46%	52%	41%	37%
CcapCSP3X1						70%	48%	40%	41%	40%	40%	38%	39%	51%
RzepCSP3X1							47%	41%	43%	42%	40%	41%	54%	38%
DmelCSP3								29%	29%	28%	29%	29%	28%	27%
BdorCSP3X2									94%	91%	89%	87%	70%	76%
BlatCSP3X2										91%	89%	87%	72%	76%
BoleCSP3X2											87%	88%	71%	74%
BcucCSP3X2												88%	68%	75%
BminCSP3X2													69%	73%
RzepCSP3X2														68%

Table S7-4 The sequence similarity of CSP4

	BlatCSP4	BoleCSP4	BminCSP4	RzepCSP4	CcapCSP4	DmelCSP4
BdorCSP4	100%	98%	95%	84%	84%	65%
BlatCSP4		98%	95%	84%	84%	65%
BoleCSP4			95%	84%	84%	65%
BminCSP4				82%	84%	66%
RzepCSP4					81%	64%
CcapCSP4						68%
