# Supplementary material for: Comparative genomic and transcriptomic analyses of chemosensory genes in the citrus fruit fly Bactrocera (Tetradacus) minax
Source: Sci Rep. 2020 Oct 22;10:18068. doi: 10.1038/s41598-020-74803-5 (PMC7583261; doi:10.1038/s41598-020-74803-5)
Supplement: Supplementary file 9 — Supplementary Information 9. [file 41598_2020_74803_MOESM9_ESM.pdf]

Supplementary file 8

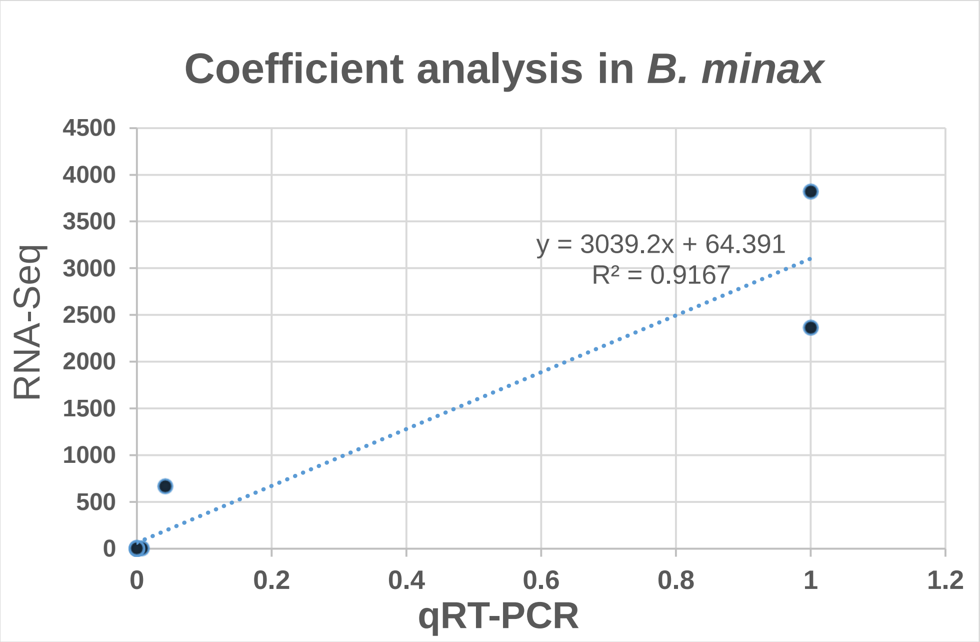

Figure S8-1 Coefficient analysis of gene expression levels obtained from RNA-seq and qRT-PCR in *B. minax*.

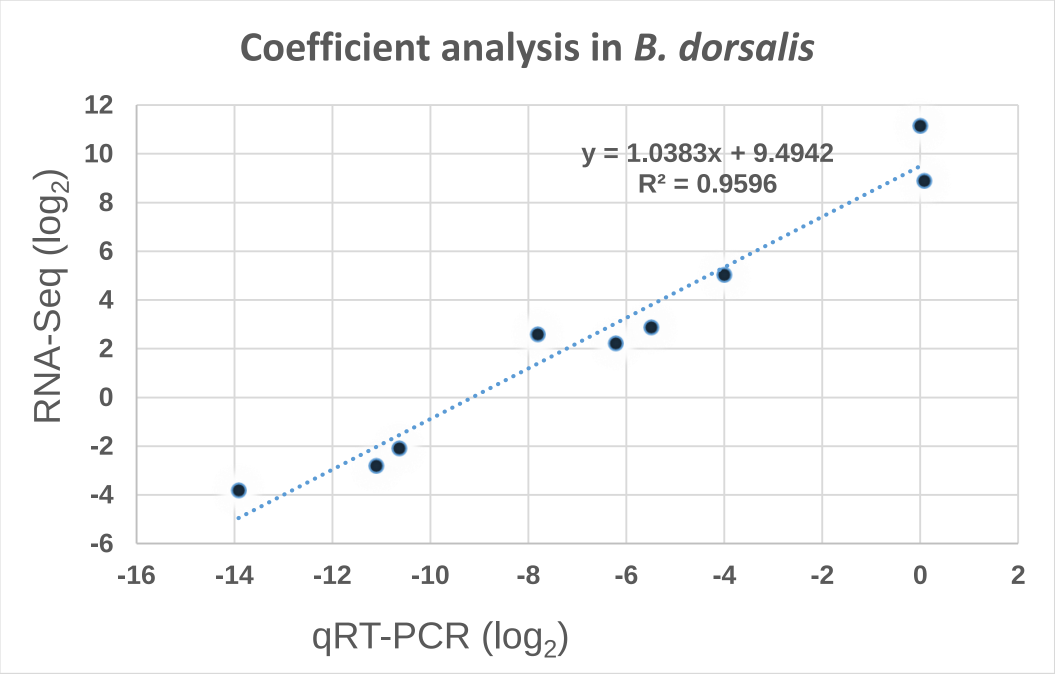

Figure S8-2 Coefficient analysis of gene expression levels obtained from RNA-seq and qRT-PCR in *B. dorsalis*. The gene expression at log_2 values.
